# Nickel-catalyzed enantioselective allylic alkylation of lactones and lactams with unactivated allylic alcohols†
†Electronic supplementary information (ESI) available: Experimental procedures, ^1^H NMR, ^13^C NMR, and IR spectra, SFC traces of racemic and chiral compounds. CCDC 1815141. For ESI and crystallographic data in CIF or other electronic format see DOI: 10.1039/c7sc05216b


**DOI:** 10.1039/c7sc05216b

**Published:** 2018-01-24

**Authors:** Aurapat Ngamnithiporn, Carina I. Jette, Shoshana Bachman, Scott C. Virgil, Brian M. Stoltz

**Affiliations:** a Warren and Katharine Schlinger Laboratory for Chemistry and Chemical Engineering , Division of Chemistry and Chemical Engineering , California Institute of Technology , Pasadena , CA 91125 , USA . Email: stoltz@caltech.edu

## Abstract

The first nickel-catalyzed enantioselective allylic alkylation of lactone and lactam substrates to deliver products bearing an all-carbon quaternary stereocenter is reported. The reaction, which utilizes a commercially available chiral bisphosphine ligand, proceeds in good yield with a high level of enantioselectivity (up to 90% ee) on a range of unactivated allylic alcohols for both lactone and lactam nucleophiles. The utility of this method is further highlighted *via* a number of synthetically useful product transformations.

## Abstract 2

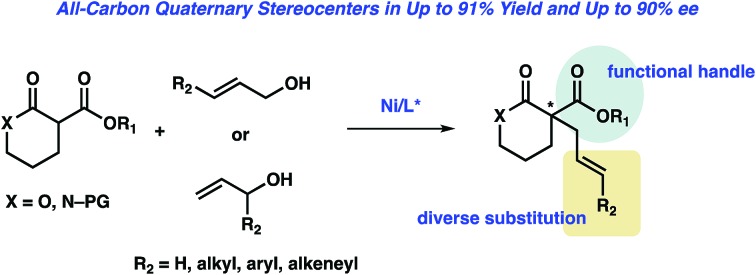
Reported is the first nickel-catalyzed enantioselective allylic alkylation of lactones and lactams to provide products bearing an all-carbon quaternary stereocenter.

## Introduction

Since the seminal report in 1965 by Tsuji,^1^ transition metal-catalyzed allylic alkylation has emerged as one of the most powerful methods for the construction of stereocenters.^2^ In particular, with the use of prochiral nucleophiles that proceed through tetrasubstituted enolates, the transition metal-catalyzed enantioselective allylic alkylation has proven to be a formidable strategy for accessing chiral quaternary stereocenters in catalytic enantioselective fashion.^3^ Although this transformation has been studied for more than 50 years,^4^ the use of α-substituted lactones or lactams as prochiral nucleophiles remains significantly under-developed.^5^,^6^

As part of our ongoing research program directed at the development of new strategies for constructing quaternary stereocenters,^7^ we were drawn to the α-acyl lactones and lactams, as we envisioned the α-acyl substituent would provide an additional functional handle for further synthetic manipulations. In addition, lactone products could also provide access to acyclic quaternary stereocenters *via* ring-opening reactions^8^ and reduction of the lactam products would enable direct access to functionalized piperidine rings, the most prevalent nitrogenous heterocycle in drug molecules.^9^ However, to the best of our knowledge, there has been only one report of a transition metal-catalyzed enantioselective allylic alkylation of monocyclic α-acyl lactone or lactam prochiral nucleophiles to furnish products bearing a quaternary stereocenter.5*d*

**Scheme 1:**
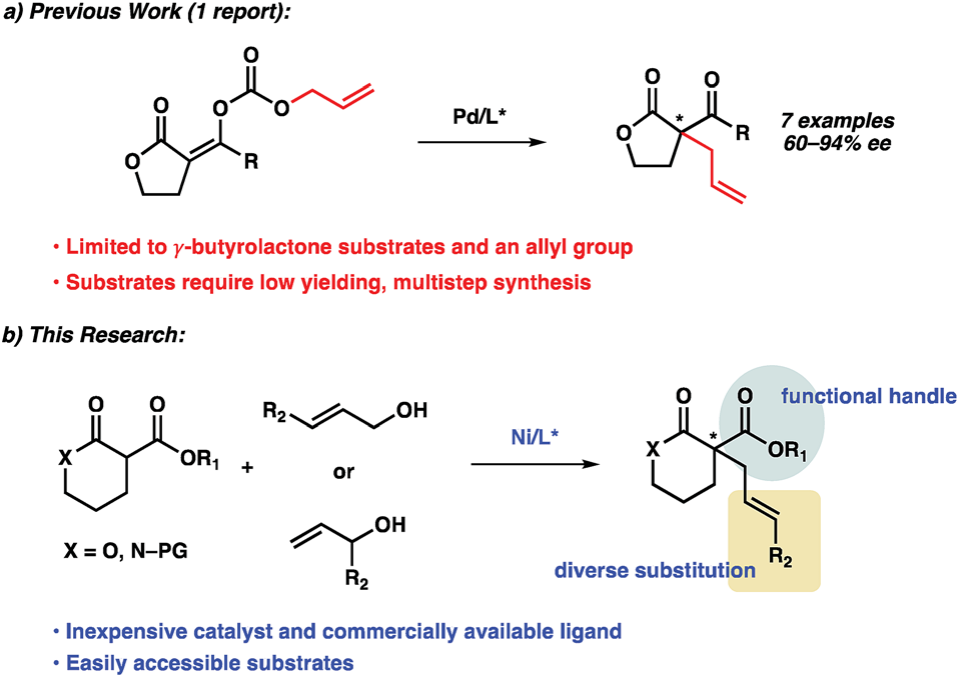
Metal-catalyzed enantioselective allylic alkylations (AA) of α-acyl lactone and lactam prochiral nucleophiles.

Recently, Cossy disclosed a palladium-catalyzed decarboxylative enantioselective allylic alkylation of enol carbonates derived from γ-butyrolactones (Scheme 1a). Various enol carbonates can be used to obtain diverse α-acyl quaternary butyrolactones in moderate to high levels of enantioselectivity. Nonetheless, the limited electrophile scope and challenging nucleophile synthesis limits the practicality of this transformation.

To address this limitation, we chose to investigate the enantioselective allylic alkylation of α-acyl lactones and lactams by using an inexpensive transition metal catalyst and easily accessible prochiral nucleophiles. Mashima's recent report on nickel-catalyzed enantioselective allylic alkylation of β-keto esters with allyl alcohol prompted us to probe nickel in our system.^10^,^11^ Furthermore, we anticipated that an intermolecular allylic alkylation would simplify the substrate synthesis and provide a more convergent approach to these α-quaternary products.^12^ Herein, we report the first example of nickel-catalyzed intermolecular enantioselective allylic alkylation using easily accessible α-acyl lactones and lactams as prochiral nucleophiles in conjunction with allylic alcohols as electrophilic coupling partners (Scheme 1b).

## Results and discussion

Our studies commenced with an investigation of the enantioselective allylic alkylation between α-ethoxycarbonyl lactone **1a** and allyl alcohol (**2a**) using Ni(COD)_2_ and (*R*)-BINAP in diethyl ether at 0 °C. Although the α-quaternary lactone product **3aa** was obtained in good yield, only moderate enantioselectivity was achieved.^13^ Seeking to improve the enantioselectivity, we elected to survey a wide variety of commercially available ligand scaffolds. Chiral bisphosphine ligands were discovered to exhibit superior enantioselectivity to other classes of ligands, including those commonly used in asymmetric allylic alkylations such as phosphinooxazolines (PHOX) or C2-asymmetric ligands pioneered by the Trost group.^14^ In the presence of Ni(COD)_2_ (10 mol%) and chiral bisphosphine ligands **L1–L4** (12 mol%) in Et_2_O, the reaction proceeds with moderate levels of enantioselectivity (Table 1, entries 1–4). Using toluene or other ethereal solvents results in lower ee.^15^ The highest enantiomeric excess (ee) was achieved with (*R*)-P-phos (**L4**), which delivers α-quaternary lactone **3aa** in 82% yield and 82% ee (entry 4). Decreasing the catalyst loading to 5 mol% requires exceedingly long reaction time (entry 5). An examination of different temperatures revealed that decreasing the temperature improves ee (entries 6–7), albeit with slightly diminished yields. Prolonged reaction time (48 h) at –10 °C affords product **3aa** in 80% yield and 85% ee (entry 8). Importantly, a control experiment performed in the absence of the chiral ligand shows no background reaction (entry 9).

**Table 1:** Optimization of reaction parameters^*a*^

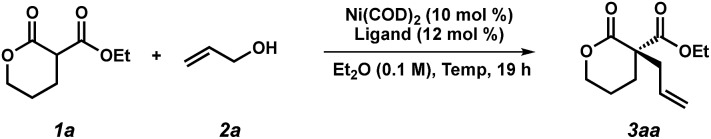				
Entry	Ligand	Temp (°C)	% Yield^*b*^	% ee^*c*^
1	**L1**	0	53	75
2	**L2**	0	76	78
3	**L3**	0	93	79
4	**L4**	0	82	82
5^*d*^	**L4**	0	62	81
6	**L4**	23	86	74
7	**L4**	–10	69	84
8^*e*^	**L4**	–10	80	85
9	—	0	0	—
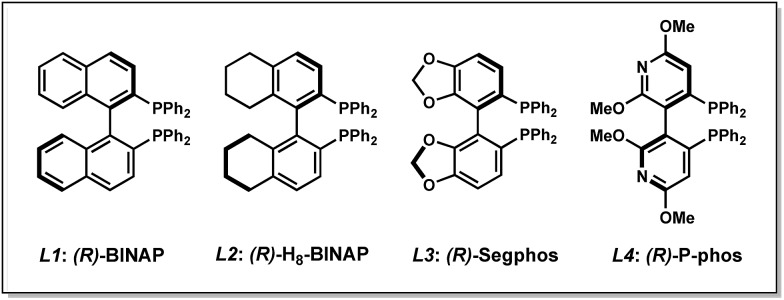				

^*a*^Conditions: **1a** (0.1 mmol), **2a** (0.1 mmol), Ni(COD)_2_ (10 mol%), ligand (12 mol%) in Et_2_O (1.0 mL).

^*b*^Yields determined by ^1^H NMR of crude reaction mixture using 1,3,5-trimethoxybenzene as a standard.

^*c*^Determined by chiral SFC analysis of the isolated product.

^*d*^Ni(COD)_2_ (5 mol%) and **L4** (6 mol%) were used.

^*e*^Reaction time = 48 h.

With the optimized reaction conditions in hand, we examined the scope of this asymmetric transformation (Table 2). The reaction of α-methoxycarbonyl lactone **1b**, possessing a smaller alkyl group at the ester fragment, with allyl alcohol (**2a**) provides α-quaternary lactone **3ba** in comparable yield and ee to the allylated product **3aa**. Bicyclic lactone **1c** could also be used to furnish product **3ca** in slightly diminished yield and enantioselectivity. With respect to the electrophile scope, reactions between lactone **1a** with various substituted allyl alcohols proceed with good ee (78–90% ee) at increased temperature (10 °C). Although a trend in enantioselectivity was not observed, we found that the electronic nature of the aryl substituent does affect the reactivity. Electrophiles containing electron rich aryl substituents provide the corresponding products in greater yields than their electron-deficient counterparts (**3ac–3ag**). Furthermore, we found that *para*- and *meta*-substituted aryl rings exhibit higher reactivity as compared to the *ortho*-substituted aryl ring (**3ac**, **3ah–ai***vs.***3aj**). Apart from the aryl-substituted electrophiles, we were pleased to find that heteroaryl substitution is also well-tolerated (**3ak–3al**). The reaction with an aliphatic electrophile affords product **3am** in slightly diminished yield and ee. In addition, an alkenyl-substituted electrophile fares well under our reaction conditions, delivering product **3an** in an excellent 91% yield and 88% ee.

**Table 2:** Nucleophile and electrophile scope^*a*^

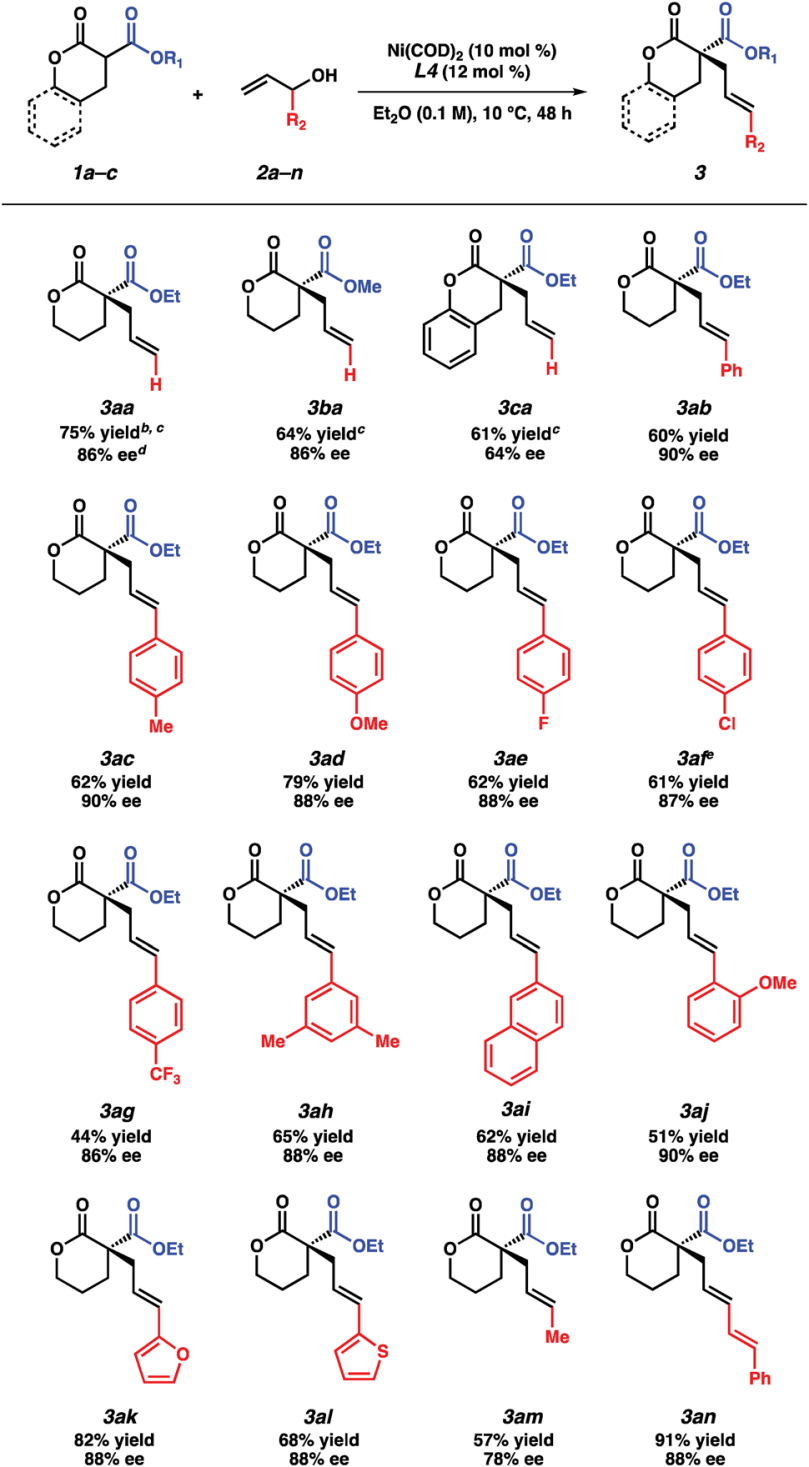

^*a*^Reactions performed on 0.2 mmol scale.

^*b*^Yield of isolated product.

^*c*^Reaction performed at –10 °C.

^*d*^Determined by chiral SFC analysis.

^*e*^Absolute configuration determined *via* single crystal X-ray analysis.

At this stage, we questioned whether we could leverage this transformation to include nitrogen-containing lactam nucleophiles. To our delight, under slightly modified reaction conditions using the same chiral bisphosphine ligand **L4**,^16^ α-ester lactams **4a–4b** furnish products **5aa–5ba** in good yields and with even higher enantioselectivity as compared to their lactone counterparts (Table 3). Examination of different protecting groups revealed that the benzoyl-protecting group is optimal.^17^ Reaction of α-ethoxycarbonyl benzoyl-protected lactam **4a** with branched cinnamyl alcohol affords linear product **5ab** in 74% yield and 90% ee.

**Table 3:** α-Acyl lactam prochiral nucleophiles^*a*^

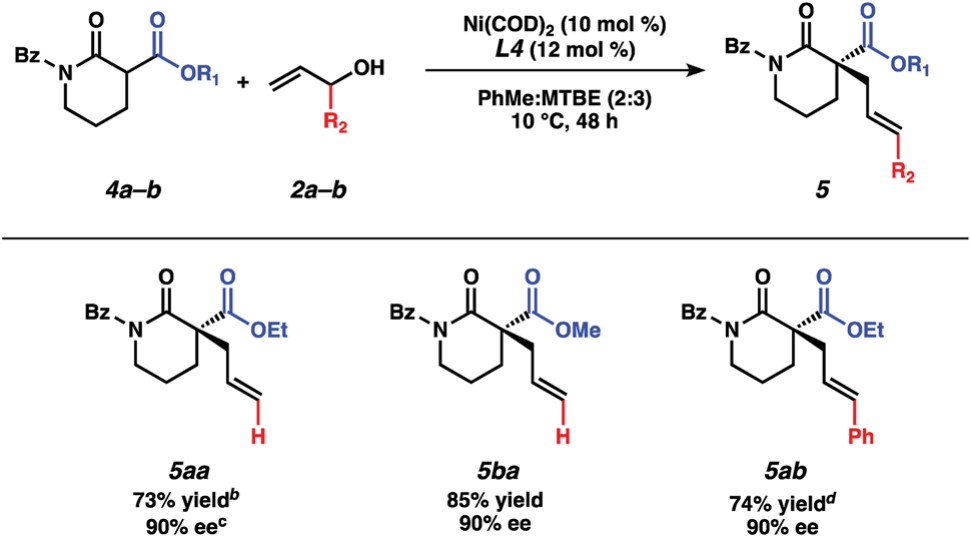

^*a*^Reactions performed on 0.2 mmol scale.

^*b*^Yield of isolated product.

^*c*^Determined by chiral SFC analysis.

^*d*^Reaction performed at 30 °C.

In order to gain mechanistic insights into this transformation, we compared the results from reactions using linear and branched cinnamyl alcohols (Table 4). Only the linear product was detected, indicating that a nickel π-allyl is likely an intermediate in the catalytic cycle.^18^ While additional studies are needed to establish the full reaction mechanism and stereocontrolling factors in the process, the ability of this catalyst combination to access a single product from two electrophilic coupling partners highlights its flexibility in potential synthetic applications.

**Table 4:** Linear *vs.* branched cinnamyl alcohol^*a*^

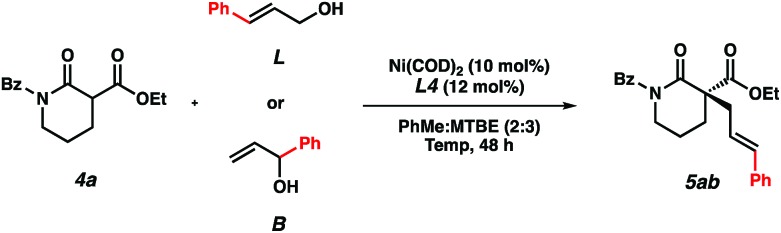					
Entry	Elec.	Temp (°C)	% Conversion^*b*^	% Yield^*b*^	% ee^*c*^
1	**L**	10	60	59	92
2	**B**	10	58	55	92
3	**L**	30	>95	86	91
4	**B**	30	90	83	91

^*a*^Reactions performed on 0.1 mmol scale.

^*b*^Yields determined by ^1^H NMR of crude reaction mixture using benzyl ether as a standard.

^*c*^Determined by chiral SFC analysis of the isolated product.

**Scheme 2:**
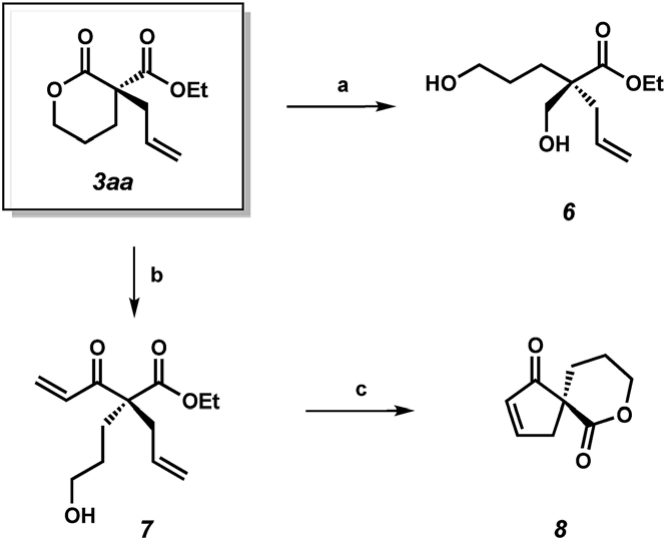
(a) NaBH_4_, CeCl_3_·7H_2_O, THF/MeOH, 0 °C, 88% yield; (b) vinyl-magnesium bromide, THF, –78 °C, 67% yield, 86% ee; (c) Grubbs' II (5 mol%), toluene, 40 °C; DBU, MeCN, 23 °C, 53% yield.

**Scheme 3:**
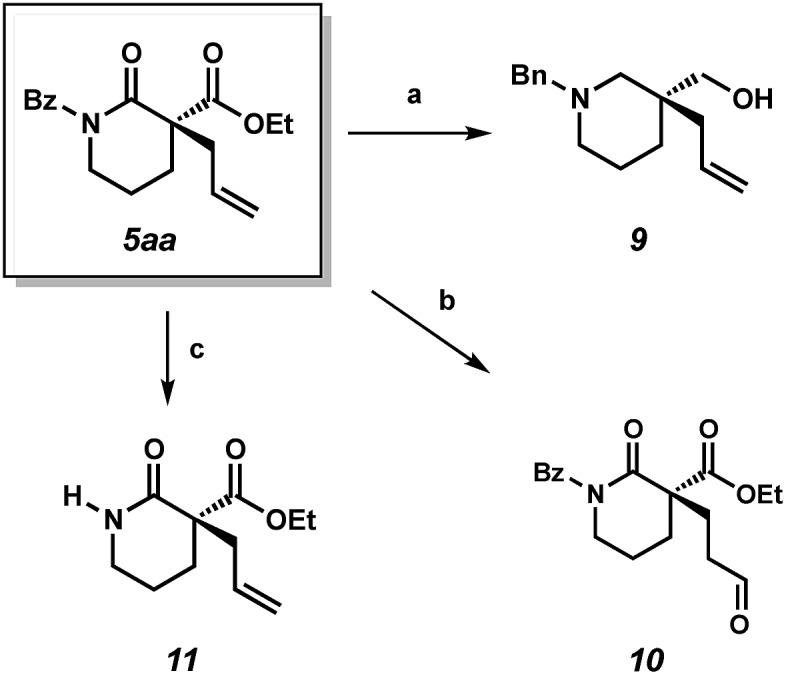
(a) LAH, ether, 65 °C, 80% yield; (b) CuCl·H_2_O (12 mol%), PdCl_2_(PhCN)_2_ (12 mol%), AgNO_2_ (6 mol%), *t*-BuOH, nitromethane under O_2_, 75% yield; (c) NaOEt, EtOH, 23 °C, 84% yield.

To demonstrate the synthetic utility of the α-quaternary products, we performed a number of product transformations on both α-quaternary lactone **3aa** (Scheme 2) and lactam **5aa** (Scheme 3). Selective reduction of the lactone functionality in **3aa** provides diol **6** in 88% yield. Additionally, vinyl Grignard addition into lactone **3aa** affords enone **7** in 67% yield with no erosion of enantioselectivity. These enantioenriched acyclic products **6** and **7** bearing a quaternary stereocenter are envisioned to be useful chiral building blocks as they contain multiple functional handles for further manipulations. For example, enantioenriched spirocycle **8** can be accessed *via* ring-closing metathesis followed by lactonization of enone **7**.

We also performed experiments to probe the reactivity of our α-quaternary lactam products. Reduction of lactam **5aa** with lithium aluminium hydride delivers chiral piperidine derivative **9**, which is of potential value to medicinal chemists.^9^ Use of the aldehyde selective Wacker procedure^19^ affords aldehyde **10** in 75% yield. Lastly, cleavage of the benzoyl protecting group under basic conditions provides unprotected lactam **11** in 84% yield.

## Conclusions

In summary, we have developed the first nickel-catalyzed enantioselective allylic alkylation of α-substituted lactones and lactams with free allylic alcohols. Utilizing a commercially available chiral bisphosphine ligand, α-quaternary lactones and lactams can be constructed in good yield (up to 91% yield) and with high enantiomeric excess (up to 90% ee). A broad range of functional groups are compatible with the reaction conditions. A number of product derivatizations showed the synthetic utility of this methodology for constructing small chiral building blocks with multiple functional handles. Future work to further elucidate the mechanism of this transformation is underway and will be reported in due course.

## Conflicts of interest

There are no conflicts to declare.

## Supplementary Material

Supplementary information

Crystal structure data
