# Supplementary material for: Nickel-catalyzed enantioselective allylic alkylation of lactones and lactams with unactivated allylic alcohols
Source: Chem Sci. 2018 Jan 24;9(9):2547–51. doi: 10.1039/c7sc05216b (PMC5912103; doi:10.1039/c7sc05216b)
Supplement: Supplementary file 1 [file SC-009-C7SC05216B-s001.pdf]

Electronic Supplementary Material (ESI) for Chemical Science.
This journal is © The Royal Society of Chemistry 2018

Nickel-Catalyzed Enantioselective Allylic Alkylation of Lactones and Lactams with Unactivated Allylic Alcohols

Aurapat Ngamnithiporn, Carina I. Jette, Shoshana Bachman, Scott C. Virgil, and Brain M. Stoltz*

Warren and Katharine Schlinger Laboratory of Chemistry and Chemical Engineering, Division of Chemistry and Chemical Engineering, California Institute of Technology, Pasadena, California 91125

stoltz@caltech.edu

Table of Contents:

Materials and Methods .... **SI 2**
List of Abbreviations .... **SI 3**
Nucleophile Synthesis .... **SI 3**
General Procedure for Catalytic Reactions .... **SI 7**
Additional Ligand Screen Results .... **SI 9**
Solvent Effects in Nickel-Catalyzed Asymmetric Allylic Alkylation of Lactones .... **SI 11**
Additional Optimization with Lactam **4a** .... **SI 12**
Spectroscopic Data for Products from Catalytic Reactions .... **SI 13**
Experimental Procedure and Characterization Data for Product Transformations .... **SI 25**
Determination of Enantiomeric Excess .... **SI 29**
Crystal Structure Analysis of Allylated Product **3af** (sample No.: p17471) .... **SI 33**
References .... **SI 42**
NMR and IR Spectra of New Compounds .... **SI 43**
SFC Traces of Racemic and Enantioenriched Compounds .... **SI 105**

Materials and Methods

Unless otherwise stated, reactions were performed in flame-dried glassware under an argon or nitrogen atmosphere using dry, deoxygenated solvents. Solvents were dried by passage through an activated alumina column under argon.[1] Reaction progress was monitored by thin-layer chromatography (TLC) or Agilent 1290 UHPLC-MS. TLC was performed using E. Merck silica gel 60 F254 precoated glass plates (0.25 mm) and visualized by UV fluorescence quenching, *p*-anisaldehyde, or $KMnO_4$ staining. Silicycle Silia*Flash*® P60 Academic Silica gel (particle size 40–63 nm) was used for flash chromatography. ^{1}H NMR spectra were recorded on Bruker 400 MHz or Varian Mercury 300 MHz spectrometers and are reported relative to residual $CHCl_3$ (δ 7.26 ppm). ^{13}C NMR spectra were recorded on Bruker 400 MHz spectrometer (101 MHz) and are reported relative to $CHCl_3$ (δ 77.16 ppm). ^{19}F NMR spectra were recorded on Varian Mercury 300 MHz spectrometer (282 MHz). Data for ^{1}H NMR are reported as follows: chemical shift (δ ppm) (multiplicity, coupling constant (Hz), integration). Multiplicities are reported as follows: s = singlet, d = doublet, t = triplet, q = quartet, p = pentet, sept = septuplet, m = multiplet, br s = broad singlet, br d = broad doublet, app = apparent. Data for ^{13}C NMR are reported in terms of chemical shifts (δ ppm). IR spectra were obtained using Perkin Elmer Spectrum BXII spectrometer or Nicolet 6700 FTIR spectrometer using thin films deposited on NaCl plates and reported in frequency of absorption (cm^{-1}). Optical rotations were measured with a Jasco P-2000 polarimeter operating on the sodium D-line (589 nm), using a 100 mm path-length cell and are reported as: $[\alpha]_D^T$ (concentration in 10 mg/1 mL, solvent) . Analytical SFC was performed with a Mettler SFC supercritical CO_2 analytical chromatography system utilizing Chiralpak (AD-H, AS-H or IC) or Chiralcel (OD-H, OJ-H, or OB-H) columns (4.6 mm x 25 cm) obtained from Daicel Chemical Industries, Ltd. High resolution mass spectra (HRMS) were obtained from Agilent 6200 Series TOF with an Agilent G1978A Multimode source in electrospray ionization (ESI+), atmospheric pressure chemical ionization (APCI+), or mixed ionization mode (MM: ESI-APCI+), or obtained from Caltech mass spectrometry laboratory. Low-temperature diffraction data (ϕ-and ω-scans) were collected on a Bruker AXS D8 VENTURE KAPPA diffractometer coupled to a PHOTON 100 CMOS detector with Cu K_α radiation (λ = 1.54178 Å) from an IμS micro-source for the structure of compound P17471. The structure was solved by direct methods using SHELXS[1] and refined against F^2 on all data by full-matrix least squares with SHELXL-2016[2] using established refinement techniques.[3] All non-

hydrogen atoms were refined anisotropically. All hydrogen atoms were included into the model at geometrically calculated positions and refined using a riding model. The isotropic displacement parameters of all hydrogen atoms were fixed to 1.2 times the *U* value of the atoms they are linked to (1.5 times for methyl groups).

Reagents were purchased from Sigma-Aldrich, Acros Organics, Strem, or Alfa Aesar and used as received unless otherwise stated.

List of Abbreviations:

ee – enantiomeric excess, SFC – supercritical fluid chromatography, TLC – thin-layer chromatography, IPA – isopropanol, MTBE – methyl *tert*-butyl ether, PE – petroleum ether, LHMDS – lithium bis(trimethylsilyl)amide, Bz – benzoyl, Ts – Tosyl, Boc – *tert*-butyloxycarbonyl

Synthesis of Nucleophiles: Experimental Procedures and Spectroscopic Data

General procedure 1: α-acylation of lactones

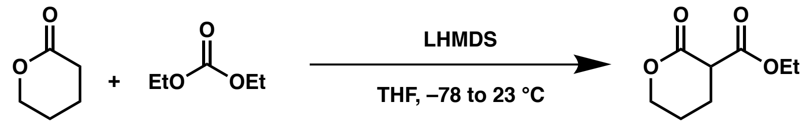

1a

Ethyl 2-oxotetrahydro-2*H*-pyran-3-carboxylate (1a):[4] To a solution of LHMDS (3.43 g, 20.5 mmol, 2.05 equiv) in THF (20 mL) was added a mixture of delta-valerolactone (1.00 g, 10.0 mmol, 1.00 equiv) and diethyl carbonate (1.3 mL, 11.0 mmol, 1.10 equiv) at –78 °C. After stirring at room temperature for 6 hours, the reaction was quenched with glacial acetic acid (5 mL), diluted with Et_2O (20 mL), and stirred for 5 minutes. The insoluble white solid was filtered off and rinsed with more Et_2O. The filtrate was concentrated and purified by column chromatography (50% to 65% Et_2O in PET) to afford **1a** as a colorless oil (1.20 g, 70% yield); ^{1}H NMR (300 MHz, $CDCl_3$) δ 4.46–4.31 (m, 2H), 4.25 (qd, *J* = 7.1, 1.7 Hz, 2H), 3.56 (dd, *J* = 8.3, 7.5 Hz, 1H), 2.38–2.08 (m, 2H), 2.08–1.80 (m, 2H), 1.30 (t, *J* = 7.1 Hz, 3H). All characterization data match those reported.[5]

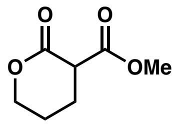

1b

Methyl 2-oxotetrahydro-2*H*-pyran-3-carboxylate (1b): Compound **1b** was prepared from dimethyl carbonate using general procedure 1 (1.38 g, 87% yield); ^{1}H NMR (300 MHz, $CDCl_3$) δ 4.46–4.32 (m, 2H), 3.80 (s, 3H), 3.58 (dd, *J* = 8.4, 7.5 Hz, 1H), 2.38–2.06 (m, 2H), 2.02–1.81 (m, 2H). All characterization data match those reported.[4]

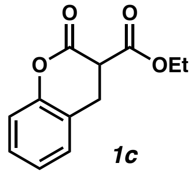

1c

Ethyl 2-oxochromane-3-carboxylate (1c): Compound **1c** was prepared from dihydrocoumarin and diethyl carbonate using general procedure 1 (0.28 g, 25% yield); ^{1}H NMR (300 MHz, $CDCl_3$) δ 7.33–7.18 (m, 2H), 7.17–6.99 (m, 2H), 4.34–4.08 (m, 2H), 3.76 (dd, *J* = 8.5, 6.1 Hz, 1H), 3.42 (dd, *J* = 16.0, 8.5 Hz, 1H), 3.18 (dd, *J* = 16.0, 6.0 Hz, 1H), 1.21 (t, *J* = 7.1 Hz, 3H). All characterization data match those reported.[6]

General procedure 2: α-acylation of lactams

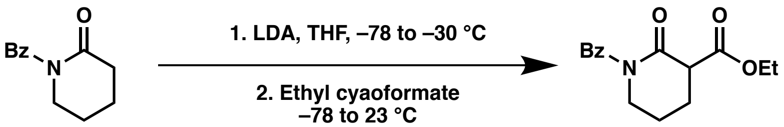

4a

Ethyl 1-benzoyl-2-oxopiperidine-3-carboxylate (4a):[7] To a solution of diisopropylamine (1.7 mL, 12 mmol, 1.2 equiv) in THF (65 mL) at 0 °C, *n*-BuLi (4.6 mL, 11 mmol, 2.4 M in hexanes, 1.1 equiv) was added dropwise over 10 minutes. After stirring for 30 min at 0 °C, the solution was cooled to –78 °C and a solution of benzoyl-protected lactam[8] (2.0 g, 12 mmol, 1.2 equiv) in THF (17 mL) was then added over 5 minutes. The reaction mixture was stirred at –78 °C for 2 hours and warmed to –30 °C for 1 hour. Ethyl cyanoformate (1.1 mL, 11 mmol, 1.1 equiv) was then added at –78 °C. The reaction was allowed to slowly warm to room temperature overnight. Upon complete consumption of starting material by TLC, the reaction was quenched with

saturated NH_4Cl. The aqueous layer was extracted with EtOAc (50 mL × 4). The combined organic phases were washed with brine (50 mL), dried over Na_2SO_4, filtered, and concentrated under vacuum. The crude residue was purified by column chromatography (30% EtOAc in hexanes) to provide product **4a** as a white amorphous solid (1.47 g, 53% yield); ^{1}H NMR (400 MHz, $CDCl_3$) δ 7.73–7.66 (m, 2H), 7.52–7.44 (m, 1H), 7.43–7.34 (m, 2H), 4.25 (q, *J* = 7.1 Hz, 2H), 3.89–3.75 (m, 2H), 3.58–3.50 (m, 1H), 2.40–2.27 (m, 1H), 2.22–2.00 (m, 2H), 2.00–1.89 (m, 1H), 1.32 (t, *J* = 7.1 Hz, 3H); ^{13}C NMR (101 MHz, $CDCl_3$) δ 174.7, 170.0, 169.6, 135.6, 132.0, 128.3, 128.2, 62.0, 51.2, 46.4, 25.6, 20.7, 14.2; IR (Neat Film, NaCl) 3062, 2980, 1734, 1701, 1683, 1476, 1449, 1392, 1285, 1258, 1185, 1152, 1113, 1026, 999, 730, 670, 638 cm^{-1}; HRMS (MM) *m/z* calc'd for $C_{15}H_{18}NO_4$ $[M+H]^+$: 276.1230, found 276.1237.

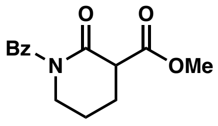

4b

Methyl 1-benzoyl-2-oxopiperidine-3-carboxylate (4b): Compound **4b** was prepared from Bz-protected lactam and methyl cyanoformate using general procedure 2 and purified by column chromatography (40% EtOAc in hexanes) to provide a colorless amorphous solid (0.33 g, 51% yield); ^{1}H NMR (400 MHz, $CDCl_3$) δ 7.73–7.65 (m, 2H), 7.48 (m, 1H), 7.43–7.36 (m, 2H), 3.86–3.80 (m, 2H), 3.79 (s, 3H), 3.59 (t, *J* = 6.4 Hz, 1H), 2.39–2.27 (m, 1H), 2.23–2.03 (m, 2H), 2.02–1.89 (m, 1H); ^{13}C NMR (101 MHz, $CDCl_3$) δ 174.7, 170.5, 169.6, 135.6, 132.0, 128.3, 128.3, 52.9, 51.1, 46.4, 25.6, 20.9; IR (Neat Film, NaCl) 2953, 1738, 1681, 1600, 1449, 1392, 1284, 1258, 1200, 1151, 1115, 1065, 973, 954, 857, 796, 731, 701, 639; HRMS (MM) *m/z* calc'd for $C_{14}H_{16}NO_4$ $[M+H]^+$: 262.1074, found 262.1066.

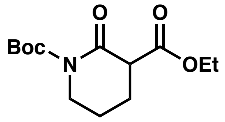

1-(*tert*-butyl) 3-ethyl 2-oxopiperidine-1,3-dicarboxylate: This compound was prepared from Boc-protected lactam[9] using general procedure 2 and purified by column chromatography (20% EtOAc in hexanes) to provide a colorless oil (0.47 g, 70% yield); ^{1}H NMR (400 MHz, $CDCl_3$) δ 4.30 – 4.13 (m, 2H), 3.75 – 3.62 (m, 2H), 3.49 (dd, *J* = 8.7, 6.8 Hz, 1H), 2.24 – 2.02 (m, 2H),

1.96 (dtt, *J* = 14.1, 6.6, 5.2 Hz, 1H), 1.81 (dddt, *J* = 14.1, 8.8, 7.5, 5.3 Hz, 1H), 1.52 (s, 9H), 1.29 (t, *J* = 7.1 Hz, 3H); ^{13}C NMR (101 MHz, $CDCl_3$) δ 170.1, 167.6, 152.8, 83.6, 61.7, 51.6, 45.9, 28.1, 24.4, 21.2, 14.2; IR (Neat Film, NaCl) 2980, 2939, 1772, 1717, 1478, 1458, 1393, 1369, 1297, 1252, 1146, 1115, 1096, 1056, 1029, 937, 852, 778, 748, 642; HRMS (MM) *m/z* calc'd for $C_{13}H_{21}NO_5Na$ $[M+Na]^+$: 294.1312, found 294.1315.

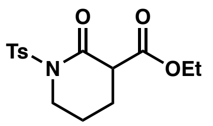

Ethyl 2-oxo-1-tosylpiperidine-3-carboxylate: This compound was prepared from tosyl-protected lactam[10] using general procedure 1 and purified by column chromatography (35% to 40% EtOAc in hexanes) to provide a colorless oil (0.32 g, 41% yield); ^{1}H NMR (400 MHz, $CDCl_3$) δ 7.90 (d, *J* = 8.4 Hz, 2H), 7.31 (d, *J* = 7.9, 2H), 4.12 (qd, *J* = 7.1, 1.2 Hz, 2H), 4.03–3.84 (m, 2H), 3.41 (dd, *J* = 7.5, 6.3 Hz, 1H), 2.43 (s, 3H), 2.19–1.97 (m, 3H), 1.96–1.82 (m, 1H), 1.18 (t, *J* = 7.1 Hz, 3H); ^{13}C NMR (101 MHz, $CDCl_3$) δ 169.3, 166.6, 145.1, 135.7, 129.5, 128.9, 61.9, 50.9, 46.6, 24.3, 21.8, 21.5, 14.1; IR (Neat Film, NaCl) 2980, 1737, 1694, 1456, 1353, 1289, 1169, 1089, 1036, 1008, 827, 815, 706, 670, 653; HRMS (MM) *m/z* calc'd for $C_{15}H_{20}NO_5S$ $[M+H]^+$: 326.1057, found 326.1066.

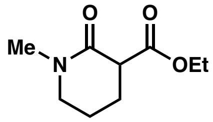

Ethyl 1-methyl-2-oxopiperidine-3-carboxylate: This compound was prepared from methyl-protected lactam using previously reported procedure;[11] ^{1}H NMR (300 MHz, $CDCl_3$) δ 4.31–4.08 (m, 2H), 3.44–3.20 (m, 3H), 2.96 (s, 3H), 2.24–1.89 (m, 3H), 1.89–1.69 (m, 1H), 1.28 (t, *J* = 7.1 Hz, 3H). All characterization data match those reported.[12]

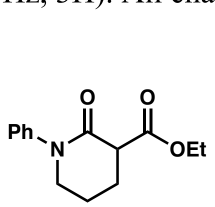

Ethyl 2-oxo-1-phenylpiperidine-3-carboxylate: This compound was prepared from phenyl-protected lactam[13] using general procedure 2 and purified by column chromatography (40%

EtOAc in hexanes) to provide a pale yellow solid (0.53 g, 42% yield); ^{1}H NMR (400 MHz, $CDCl_3$) δ 7.42–7.35 (m, 2H), 7.29–7.22 (m, 3H), 4.31–4.15 (m, 2H), 3.76–3.61 (m, 2H), 3.57 (dd, *J* = 7.8, 6.4 Hz, 1H), 2.35–2.04 (m, 3H), 2.00–1.88 (m, 1H), 1.30 (t, *J* = 7.1 Hz, 3H); ^{13}C NMR (101 MHz, $CDCl_3$) δ 171.1, 166.2, 142.9, 129.3, 127.0, 126.1, 61.5, 51.4, 49.7, 25.3, 21.5, 14.3; IR (Neat Film, NaCl) 2943, 1734, 1654, 1595, 1494, 1462, 1427, 1371, 1353, 1308, 1259, 1197, 1171, 1036, 763, 697, 659; HRMS (MM) *m/z* calc'd for $C_{14}H_{18}NO_3$ $[M+H]^+$: 248.1281, found 248.1278.

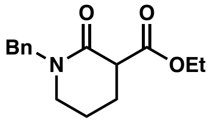

Ethyl 1-benzyl-2-oxopiperidine-3-carboxylate: This compound was prepared from benzyl-protected lactam[14] using general procedure 2 (0.32 g, 56% yield); ^{1}H NMR (300 MHz, $CDCl_3$) δ 7.37–7.23 (m, 5H), 4.73 (d, *J* = 14.7 Hz, 1H), 4.51 (d, *J* = 14.7 Hz, 1H), 4.24 (qd, *J* = 7.1, 4.0 Hz, 2H), 3.59–3.43 (m, 1H), 3.36–3.12 (m, 2H), 2.29–1.97 (m, 2H), 1.97–1.83 (m, 1H), 1.82–1.64 (m, 1H), 1.31 (t, *J* = 7.2 Hz, 3H). All characterization data match those reported.[15]

Nickel-Catalyzed Asymmetric Allylic Alkylation Reactions: General Procedures

Please note that the absolute configuration was determined only for compound **3af** via x-ray crystallographic analysis. The absolute configuration for all other products has been inferred by analogy. For respective HPLC and SFC conditions, please refer to Table S3.

General procedure 3: Nickel-catalyzed asymmetric allylic alkylation of lactones

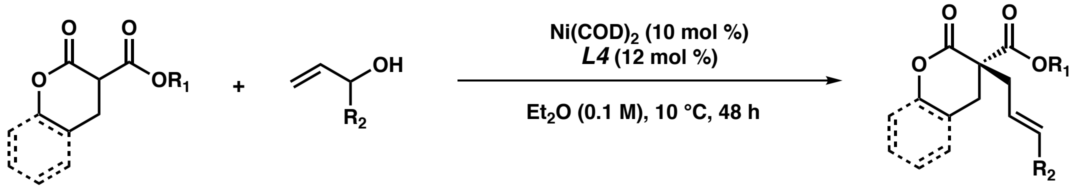

In a nitrogen-filled glovebox, to an oven-dried 4-mL vial equipped with a stir bar was added *(R)*-P-Phos ligand ***L4*** (15.5 mg, 0.024 mmol, 12 mol%) and $Ni(COD)_2$ (5.5 mg, 0.02 mmol, 10 mol%) in Et_2O (1.2 mL). The vial was then capped with a PTFE-lined septum cap and stirred at room temperature. After 30 minutes, the catalyst mixture was cooled to 10 °C. Precooled

nucleophile (0.2 mmol, 1 equiv) in Et_2O (0.4 mL) and electrophile (0.2 mmol, 1 equiv) in Et_2O (0.4 mL) at 10 °C were prepared and then added to the catalyst mixture at 10 °C. The vial was sealed with a PTFE-lined septum cap and stirred at 10 °C. After 48 h, the vial was removed from the glovebox. The crude reaction mixture was filtered through a silica plug with Et_2O, concentrated under vacuum, and purified by silica gel flash chromatography to furnish the product.

General procedure 4: Nickel-catalyzed asymmetric allylic alkylation of lactams

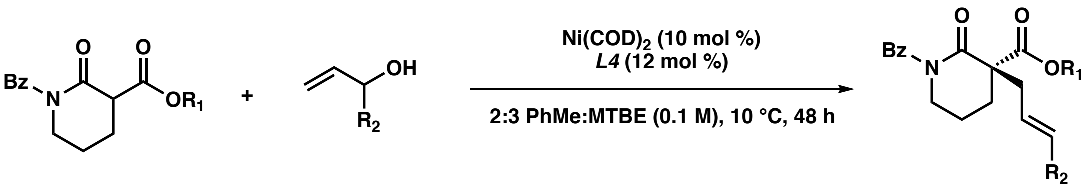

In a nitrogen-filled glovebox, to an oven-dried 4-mL vial equipped with a stir bar was added *(R)*-P-Phos ligand ***L4*** (15.5 mg, 0.024 mmol, 12 mol%) and $Ni(COD)_2$ (5.5 mg, 0.02 mmol, 10 mol %) in MTBE (1.2 mL). The vial was then capped with a PTFE-lined septum cap and stirred at room temperature. After 30 minutes, the catalyst mixture was cooled to 10 °C. Precooled nucleophile (0.2 mmol, 1 equiv) in toluene (0.4 mL) and electrophile (0.2 mmol, 1 equiv) in toluene (0.4 mL) at 10 °C were prepared and then added to the catalyst mixture at 10 °C. The vial was sealed with a PTFE-lined septum cap and stirred at 10 °C. After 48 h, the vial was removed from the glovebox. The crude reaction mixture was filtered through a silica plug with Et_2O, concentrated under vacuum, and purified by silica gel flash chromatography to furnish the product.

Additional Ligand Screen Results

Table S1. Additional Ligand Screen

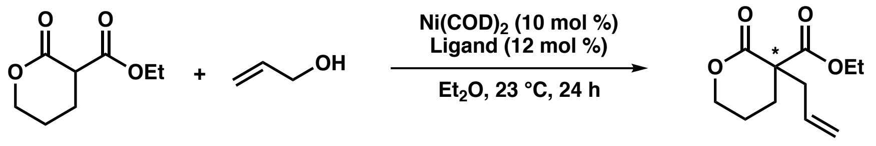

Entry	Ligand	ee (%)	Entry	Ligand	ee (%)
1	*L5*	14	19	*L23*	0
2	*L6*	20	20	*L24*	–34
3	*L7*	–	21	*L25*	–
4	*L8*	–60	22	*L26*	–6
5	*L9*	57	23	*L27*	3
6	*L10*	67	24	*L28*	–
7	*L11*	–63	25	*L29*	31
8	*L12*	–	26	*L30*	9
9	*L13*	8	27	*L31*	–15
10	*L14*	19	28	*L32*	–22
11	*L15*	–	29	*L33*	–
12	*L16*	11	30	*L34*	–73
13	*L17*	24	31	*L35*	–
14	*L18*	–	32	*L36*	–
15	*L19*	12	33	*L37*	–
16	*L20*	–	34	*L38*	–
17	*L21*	–	35	*L39*	–
18	*L22*	17	36	*L40*	–44

Ligand List:

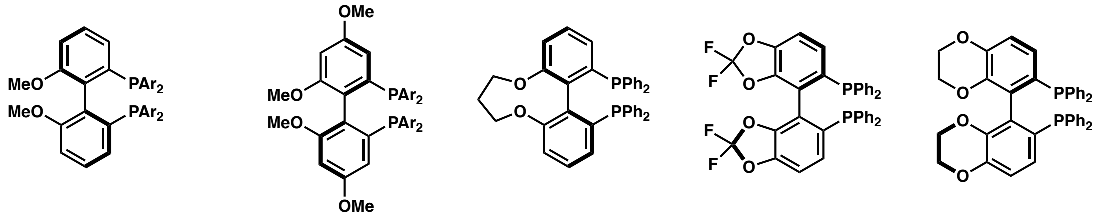

L5: Ar = 3,5-t-Bu$_2$C$_6$H$_3$
L6: Ar = 3,5-t-Bu$_2$-4-MeOC$_6$H$_2$

L7: Ar = 3,5-(CF$_3$)$_2$C$_6$H$_3$

L8 ***L9*** ***L10***

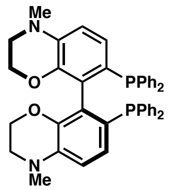

L11

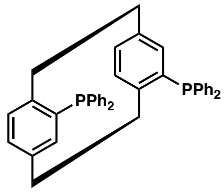

L12

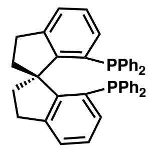

L13

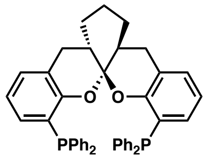

L14

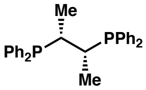

L15

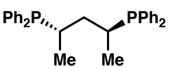

L16

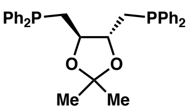

L17

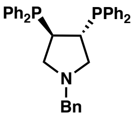

L18

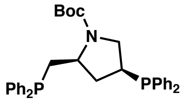

L19

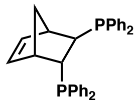

L20

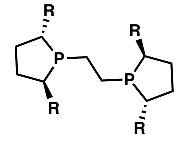

L21: R = Me
L22: R = i-Pr

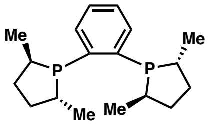

L23

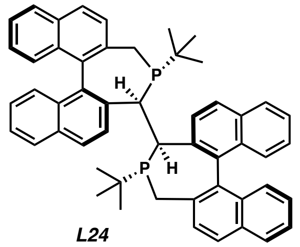

L24

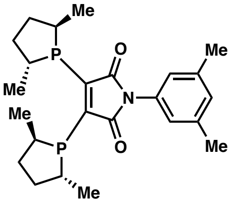

L25

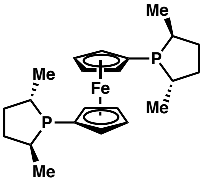

L26

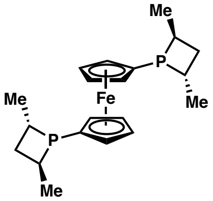

L27

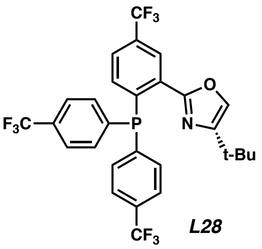

L28

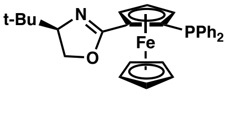

L29

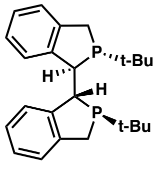

L30

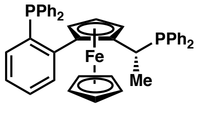

L31

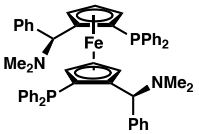

L32

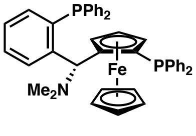

L33

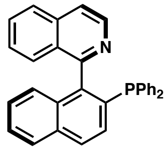

L35

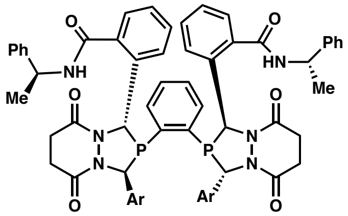

L34

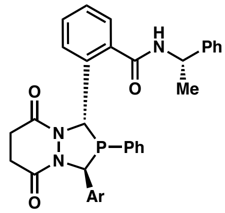

L36

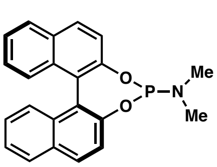

L37

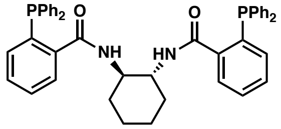

L38

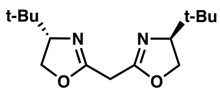

L39

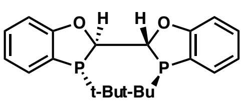

L40

Solvent Effects in Nickel-Catalyzed Asymmetric Allylic Alkylation of Lactones

Table S2. Solvent Effects[a]

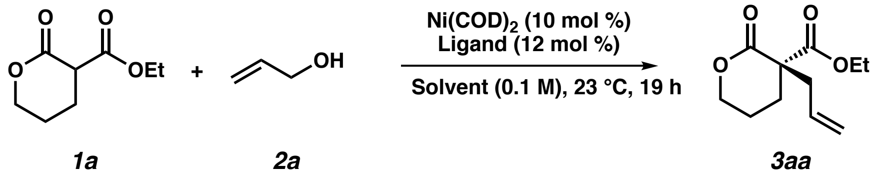

Ligand	Solvent (% ee)[b]				
	Et_2O	MTBE	THF	Dioxane	Toluene
L1: *(R)*-BINAP	62% ee	65% ee	41% ee	18% ee	45% ee
L2: *(R)*-H_8-BINAP	74% ee	72% ee	60% ee	22% ee	46% ee
L3: *(R)*-Segphos	72% ee	70% ee	45% ee	28% ee	46% ee
L4: *(R)*-P-phos	74% ee	67% ee	52% ee	25% ee	51% ee

[a] Conditions: lactone (0.05 mmol), alcohol (0.05 mmol), $Ni(COD)_2$ (10 mol %), ligand (12 mol %) for 19 h.
[b] Determined by chiral SFC analysis.

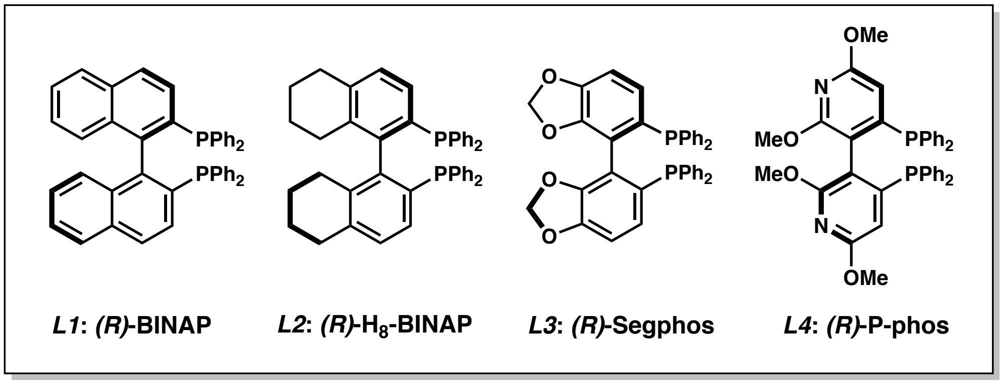

Optimization of Reaction Parameters for Lactams

Table S3. Optimization of reaction parameters for lactam 4a[a]

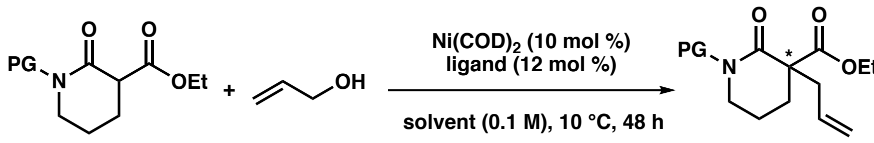

entry	PG	ligand	solvent	yield [%][b]	ee [%][c]
1	Bz	L2	PhMe:MTBE (2:3)	95	77
2	Bz	L3	PhMe:MTBE (2:3)	>95	88
3	Bz	L4	PhMe:MTBE (2:3)	79	90
4[d]	Bz	L4	PhMe:MTBE (2:3)	28	88
5	Bz	L4	PhMe:Et_2O (2:3)	70	88
6	Bz	L4	PhMe	51	88
7	Bz	L4	THF	15	76
8[e]	Bz	L4	PhMe:MTBE (2:3)	>95	88

[a] Conditions: lactam (0.1 mmol), alcohol (0.1 mmol), Ni(COD)$_2$ (10 mol %), ligand (12 mol %) for 48 h. [b] Yields determined by ^{1}H NMR of crude reaction mixture using trimethoxybenzene as a standard. [c] Determined by chiral SFC analysis. [d] 5 mol % Ni(COD)$_2$ and 6 mol % L4 were used. [e] Reaction performed at 23 °C.

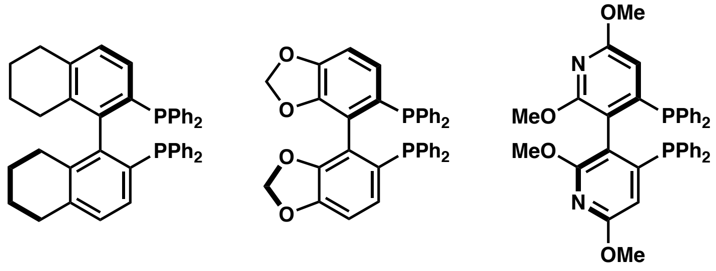

L2: *(R)*-H$_8$-BINAP L3: *(R)*-Segphos L4: *(R)*-P-phos

Spectroscopic Data for Products from Catalytic Reactions

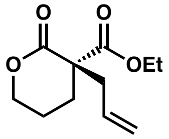

3aa

Ethyl (*R*)-3-allyl-2-oxotetrahydro-2*H*-pyran-3-carboxylate (3aa)

Product **3aa** was prepared using general procedure 3 at –10 °C and purified by column chromatography (15% EtOAc in hexanes) to provide a colorless oil (31.8 mg, 75% yield); 86% ee, $[\alpha]_D^{25}$ +3.84 (*c* 0.99, $CHCl_3$); ^{1}H NMR (400 MHz, $CDCl_3$) δ ^{1}H NMR (400 MHz, $CDCl_3$) δ 5.84–5.69 (m, 1H), 5.19–5.08 (m, 2H), 4.34–4.23 (m, 2H), 4.21 (q, *J* = 7.1 Hz, 2H), 2.73 (ddt, *J* = 13.8, 6.8, 1.2 Hz, 1H), 2.59 (ddt, *J* = 13.9, 7.9, 1.0 Hz, 1H), 2.38–2.25 (m, 1H), 2.05–1.88 (m, 1H), 1.92–1.79 (m, 2H), 1.27 (t, *J* = 7.1 Hz, 3H); ^{13}C NMR (101 MHz, $CDCl_3$) δ 171.2, 170.0, 132.6, 119.9, 69.0, 62.2, 54.0, 40.8, 28.0, 20.6, 14.2; IR (Neat Film, NaCl) 2981, 1732, 1457, 1399, 1367, 1348, 1244, 1200, 1162, 1108, 1026, 974, 925, 857, 640 cm^{-1}; HRMS (MM) *m/z* calc'd for $C_{11}H_{17}O_4$ $[M+H]^+$: 213.1121, found 213.1120; SFC Conditions: 25% IPA, 2.5 mL/min, Chiralpak IC column, λ = 210 nm, t_R (min): major = 2.66, minor = 3.29.

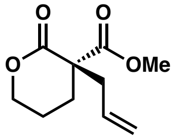

3ba

Methyl (*R*)-3-allyl-2-oxotetrahydro-2*H*-pyran-3-carboxylate (3ba)

Product **3ba** was prepared using general procedure 3 at –10 °C and purified by column chromatography (30% EtOAc in hexanes) to provide a colorless oil (25.5 mg, 64% yield); 86% ee, $[\alpha]_D^{25}$ + 5.071 (*c* 0.896, $CHCl_3$); ^{1}H NMR (400 MHz, $CDCl_3$) δ 5.85–5.66 (m, 1H), 5.20–5.10 (m, 2H), 4.33–4.26 (m, 2H), 3.76 (s, 3H), 2.75 (ddt, *J* = 13.8, 6.8, 1.3 Hz, 1H), 2.61 (ddt, *J* = 13.8, 7.8, 1.0 Hz, 1H), 2.39–2.26 (m, 1H), 2.03–1.77 (m, 3H); ^{13}C NMR (101 MHz, $CDCl_3$) δ 171.8, 169.9, 132.5, 120.1, 69.2, 54.1, 53.2, 40.9, 28.1, 20.6; IR (Neat Film, NaCl) 3079, 2955, 2920, 1733, 1640, 1480, 1436, 1401, 1349, 1321, 1277, 1247, 1204, 1164, 1122, 1108, 1076, 1000, 978, 126, 844, 716, 659, 640; HRMS (MM) *m/z* calc'd for $C_{10}H_{15}O_4$ $[M+H]^+$: 199.0965,

found 199.0970; SFC Conditions 20% IPA, 2.5 mL/min, Chiralpak IC column λ = 210 nm, t_R (min): major = 3.35, minor = 3.99.

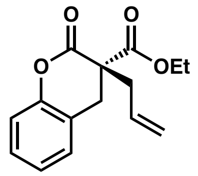

3ca

Ethyl (*S*)-3-allyl-2-oxochromane-3-carboxylate (3ca)

Product **3ca** was prepared using general procedure 3 at –10 °C and purified by column chromatography (5% EtOAc in hexanes) to provide a colorless oil (31.9 mg, 61% yield); 64% ee, $[\alpha]_D^{25}$ –30.75 (*c* 0.92, $CHCl_3$); ^{1}H NMR (400 MHz, $CDCl_3$) δ 7.33–7.13 (m, 2H), 7.13–7.00 (m, 2H), 5.91 (ddt, *J* = 16.6, 10.6, 7.3 Hz, 1H), 5.23–5.12 (m, 2H), 4.05 (qq, *J* = 10.8, 7.1 Hz, 2H), 3.26 (d, 15.9 Hz, 1 H), 3.04 (d, *J* = 15.9 Hz, 1H), 2.84–2.67 (m, 2H), 1.02 (t, *J* = 7.1 Hz, 3H); ^{13}C NMR (101 MHz, $CDCl_3$) δ 169.5, 167.2, 151.2, 132.1, 128.7, 128.5, 124.8, 121.4, 120.4, 116.5, 62.2, 53.3, 38.6, 32.5, 14.0; IR (Neat Film, NaCl) 3079, 2982, 2936, 1774, 1738, 1653, 1640, 1590, 1541, 1490, 1460, 1344, 1232, 1190, 1145, 1096, 1020, 921, 858, 759, 658; HRMS (MM) *m/z* calc'd for $C_{15}H_{17}O_4$ $[M+H]^+$: 261.1121, found 261.1123; SFC Conditions: 5% IPA, 2.5 mL/min, Chiralcel OB-H column, λ = 210 nm, t_R (min): minor = 2.22, major = 2.64.

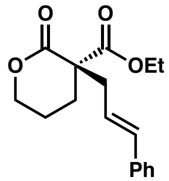

3ab

Ethyl (*R*)-3-cinnamyl-2-oxotetrahydro-2*H*-pyran-3-carboxylate (3ab)

Product **3ab** was prepared using general procedure 3 and purified by column chromatography (15% EtOAc in hexanes) to provide a colorless oil (34.4 mg, 60% yield); 90% ee, $[\alpha]_D^{25}$ –12.15 (*c* 0.64, $CHCl_3$); ^{1}H NMR (400 MHz, $CDCl_3$) δ 7.38–7.25 (m, 4H), 7.27–7.17 (m, 1H), 6.47 (dt, *J* = 16.0, 1.4 Hz, 1H), 6.19 (ddd, *J* = 15.8, 8.0, 7.0 Hz, 1H), 4.35–4.17 (m, 4H), 2.91 (ddd, *J* = 13.8, 7.0, 1.4 Hz, 1H), 2.74 (ddd, *J* = 13.8, 8.0, 1.2 Hz, 1H), 2.45–2.31 (m, 1H), 2.11–1.77 (m, 3H), 1.27 (t, *J* = 7.1 Hz, 3H); ^{13}C NMR (101 MHz, $CDCl_3$) δ 171.2, 170.1, 136.9, 134.8, 128.7,

127.7, 126.4, 124.1, 69.1, 62.3, 54.4, 40.1, 28.1, 20.6, 14.2; IR (Neat Film, NaCl) 2980, 2342, 1955, 1733, 1577, 1449, 1399, 1367, 1243, 1198, 1164, 1026, 971, 910, 858, 746, 695, 642 cm^{-1}; HRMS (MM) *m/z* calc'd for $C_{17}H_{21}O_4$ $[M+H]^+$: 289.1430, found 289.1434; SFC Conditions: 10% IPA, 2.5 mL/min, Chiralpak AD-H column, λ = 254 nm, t_R (min): major = 5.49, minor = 6.31.

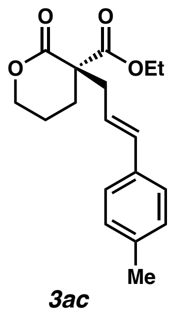

3ac

Ethyl (*R*,*E*)-2-oxo-3-(3-(*p*-tolyl)allyl)tetrahydro-2*H*-pyran-3-carboxylate (3ac)

Product **3ac** was prepared using general procedure 3 and purified by column chromatography (15% EtOAc in hexanes) to provide a white amorphous solid (37.5 mg, 62% yield); 90% ee, $[\alpha]_D^{25}$ –14.42 (*c* 0.95, $CHCl_3$); ^1H NMR (400 MHz, $CDCl_3$) δ 7.24 (d, *J* = 8.1 Hz, 2H), 7.15–6.98 (m, 2H), 6.51–6.33 (m, 1H), 6.13 (ddd, *J* = 15.8, 8.1, 7.0 Hz, 1H), 4.31–4.26 (m, 2H), 4.23 (q, *J* = 7.1 Hz, 2H), 2.90 (ddd, *J* = 13.8, 7.0, 1.4 Hz, 1H), 2.72 (ddd, *J* = 13.8, 8.1, 1.2 Hz, 1H), 2.41–2.25 (m, 4H), 2.02–1.78 (m, 3H), 1.27 (t, *J* = 7.1 Hz, 3H); ^{13}C NMR (101 MHz, $CDCl_3$) δ 171.3, 170.1, 137.5, 134.7, 134.2, 129.3, 126.3, 123.0, 69.1, 62.3, 54.4, 40.2, 28.1, 21.3, 20.6, 14.2; IR (Neat Film, NaCl) 2978, 1731, 1513, 1456, 1399, 1367, 1269, 1242, 1197, 1163, 1096, 1025, 972, 859, 803, 642 cm^{-1}; HRMS (MM) *m/z* calc'd for $C_{18}H_{23}O_4$ $[M+H]^+$: 303.1591, found 303.1591; SFC Conditions: 10% IPA, 2.5 mL/min, Chiralpak AD-H column, λ = 254 nm, t_R (min): major 6.47, minor = 7.71.

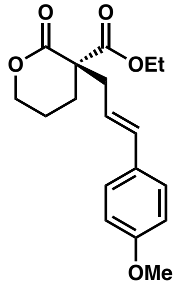

3ad

Ethyl (*R*,*E*)-3-(3-(4-methoxyphenyl)allyl)-2-oxotetrahydro-2*H*-pyran-3-carboxylate (3ad)

Product **3ad** was prepared using general procedure 3 and purified by column chromatography (15% EtOAc in hexanes) to provide a colorless oil (50.5 mg, 79% yield); 88% ee, $[\alpha]_D^{25}$ –15.9 (*c* 0.95, $CHCl_3$); ^1H NMR (400 MHz, $CDCl_3$) δ 7.32–7.24 (m, 2H), 6.89–6.79 (m, 2H), 6.41 (d, 15.8 Hz, 1H), 6.03 (ddd, *J* = 15.7, 8.0, 7.0 Hz, 1H), 4.29 (t, *J* = 5.9 Hz, 2H), 4.22 (q, *J* = 7.2 Hz, 2H), 3.79 (s, 3H), 2.89 (ddd, *J* = 13.8, 7.0, 1.4 Hz, 1H), 2.71 (ddd, *J* = 13.7, 8.1, 1.2 Hz, 1H), 2.43–2.29 (m, 1H), 2.05–1.79 (m, 3H), 1.27 (t, *J* = 7.1 Hz, 3H); ^{13}C NMR (101 MHz, $CDCl_3$) δ 171.3, 170.2, 159.2, 134.2, 129.8, 127.5, 121.7, 114.0, 69.1, 62.2, 55.4, 54.5, 40.1, 28.1, 20.6, 14.2; IR (Neat Film, NaCl) 2978, 2837, 1732, 1608, 1577, 1512, 1457, 1400, 1349, 1367, 1249, 1198, 1108, 1032, 972, 840, 757, 667, 640; HRMS (MM) *m/z* calc'd for $C_{18}H_{23}O_5$ $[M+H]^+$: 319.1540, found 319.1525; SFC Conditions: 15% IPA, 2.5 mL/min, Chiralpak AD-H column, λ = 254 nm, t_R (min): major = 5.37, minor = 6.37.

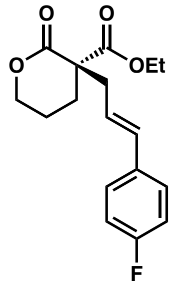

3ae

Ethyl (*R*,*E*)-3-(3-(4-fluorophenyl)allyl)-2-oxotetrahydro-2*H*-pyran-3-carboxylate (3ae)

Product **3ae** was prepared using general procedure 3 and purified by column chromatography (15% EtOAc in hexanes) to provide a colorless oil (38.2 mg, 62% yield); 88% ee, $[\alpha]_D^{25}$ –10.19 (*c* 0.86, $CHCl_3$); ^1H NMR (400 MHz, $CDCl_3$) δ 7.34–7.27 (m, 2H), 7.05–6.90 (m, 2H), 6.53–6.34 (m, 1H), 6.20–6.02 (m, 1H), 4.29 (t, *J* = 5.6 Hz, 2H), 4.22 (q, *J* = 7.1 Hz, 2H), 2.87 (ddd, *J* = 13.9, 7.1, 1.4 Hz, 1H), 2.72 (ddd, *J* = 13.8, 7.9, 1.2 Hz, 1H), 2.47–2.31 (m, 1H), 2.07–1.78 (m,

3H), 1.26 (t, *J* = 7.1 Hz, 3H); ^{13}C NMR (101 MHz, $CDCl_3$) δ 171.2, 170.1, 162.4 (d, *J* = 246.8 Hz), 133.5, 133.1 (d, *J* = 3.3 Hz), 127.9 (d, *J* = 8.0 Hz), 123.9 (d, *J* = 2.2 Hz), 115.5 (d, *J* = 21.7 Hz), 69.0, 62.3, 54.4, 40.1, 28.2, 20.6, 14.2; ^{19}F NMR (282 MHz, $CDCl_3$) δ -114.56 (tt, *J* = 8.6, 5.3 Hz); IR (Neat Film, NaCl) 2981, 2342, 1733, 1602, 1508, 1456, 1400, 1368, 1349, 1298, 1269, 1226, 1198, 1160, 1095, 1025, 972, 847, 767, 711, 668, 639 cm^{-1}; HRMS (MM) *m/z* calc'd for $C_{17}H_{20}FO_4$ $[M+H]^+$: 307.1340 found 307.1343; SFC Conditions: 10% IPA, 2.5 mL/min, Chiralpak AD-H column, λ = 254 nm, t_R (min): major = 5.12, minor = 5.95.

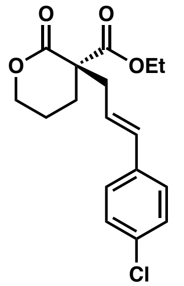

3af

Ethyl (*R*,*E*)-3-(3-(4-chlorophenyl)allyl)-2-oxotetrahydro-2*H*-pyran-3-carboxylate (3af)

Product **3af** was prepared using general procedure 3 and purified by column chromatography (15% EtOAc in hexanes) to provide a colorless oil (39.5 mg, 61% yield); 87% ee, $[\alpha]_D^{25}$ –10.81 (*c* 0.83, $CHCl_3$); ^{1}H NMR (400 MHz, $CDCl_3$) δ 7.26 (s, 4H), 6.42 (dt, *J* = 15.7, 1.3 Hz, 1H), 6.18 (ddd, *J* = 15.9, 7.9, 7.1 Hz, 1H), 4.29 (t, *J* = 5.7 Hz, 2H), 4.22 (q, *J* = 7.1 Hz, 2H), 2.87 (ddd, *J* = 13.9, 7.1, 1.4 Hz, 1H), 2.74 (ddd, *J* = 13.8, 7.9, 1.2 Hz, 1H), 2.46–2.32 (m, 1H), 2.15–1.80 (m, 3H), 1.26 (t, *J* = 7.1 Hz, 3H); ^{13}C NMR (101 MHz, $CDCl_3$) δ 171.1, 170.1, 135.4, 133.5, 133.2, 128.8, 127.6, 124.9, 69.0, 62.3, 54.4, 40.1, 28.2, 20.6, 14.2; IR (Neat Film, NaCl) 2979, 2358, 1729, 1490, 1455, 1404, 1243, 1197, 1164, 1092, 971, 820, 760, 679 cm^{-1}; HRMS (MM) *m/z* calc'd for $C_{17}H_{20}ClO_4$ $[M+H]^+$: 323.1045, found 323.1041; SFC Conditions: 30% IPA, 2.5 mL/min, Chiralpak AD-H column, λ = 254 nm, t_R (min): major = 2.29, minor = 2.57.

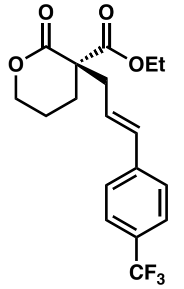

3ag

Ethyl (*R*,*E*)-2-oxo-3-(3-(4-(trifluoromethyl)phenyl)allyl)tetrahydro-2*H*-pyran-3-carboxylate (3ag)

Product **3ag** was prepared using general procedure 3 and purified by column chromatography (15% EtOAc in hexanes) to provide a colorless oil (31.2 mg, 44% yield); 86% ee, $[\alpha]_D^{25}$ –6.52 (*c* 0.98, $CHCl_3$); ^1H NMR (400 MHz, $CDCl_3$) δ 7.60–7.47 (m, 2H), 7.47–7.38 (m, 2H), 6.50 (d, *J* = 15.8 Hz, 1H), 6.32 (dt, *J* = 15.8, 7.5 Hz, 1H), 4.30 (dd, *J* = 6.3, 5.2 Hz, 2H), 4.23 (q, *J* = 7.1 Hz, 2H), 2.90 (ddd, *J* = 13.8, 7.1, 1.3 Hz, 1H), 2.77 (ddd, *J* = 13.8, 7.7, 1.2 Hz, 1H), 2.47–2.34 (m, 1H), 2.05–1.81 (m, 3H), 1.26 (t, *J* = 7.1 Hz, 3H); ^{13}C NMR (101 MHz, $CDCl_3$) δ 171.1, 170.0, 140.4 (d, *J* = 1.6 Hz), 133.4, 129.4 (q, *J* = 32.4 Hz), 127.2, 126.5, 125.6 (q, *J* = 3.7 Hz), 122.9, 69.0, 62.4, 54.4, 40.1, 28.3, 20.6, 14.2; ^{19}F NMR (282 MHz, $CDCl_3$) δ –62.52 (s); IR (Neat Film, NaCl) 2982, 1733, 1684, 1616, 1540, 1414, 1326, 1244, 1198, 1163, 1120, 1068, 1016, 972, 862, 833, 652; HRMS (MM) *m/z* calc'd for $C_{18}H_{20}F_3O_4$ $[M+H]^+$: 357.1308, found 357.1307; SFC Conditions: 10% IPA, 2.5 mL/min, Chiralpak AD-H column, λ = 254 nm, t_R (min): major = 4.02, minor = 4.72.

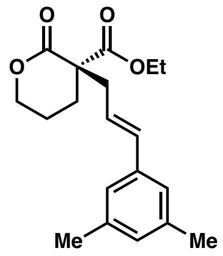

3ah

Ethyl (*R*,*E*)-3-(3-(3,5-dimethylphenyl)allyl)-2-oxotetrahydro-2*H*-pyran-3-carboxylate (3ah)

Product **3ah** was prepared using general procedure 3 and purified by column chromatography (15% EtOAc in hexanes) to provide a colorless oil (41.0 mg, 65% yield); 88% ee, $[\alpha]_D^{25}$ –13.58 (*c* 0.84, $CHCl_3$); ^1H NMR (400 MHz, $CDCl_3$) δ 7.00–6.94 (m, 2H), 6.87 (dt, *J* = 1.9, 1.0 Hz,

1H), 6.46–6.36 (m, 1H), 6.15 (ddd, *J* = 15.7, 8.2, 6.8 Hz, 1H), 4.32–4.27 (m, 2H), 4.27–4.20 (m, 2H), 2.91 (ddd, *J* = 13.8, 6.8, 1.4 Hz, 1H), 2.71 (ddd, *J* = 13.7, 8.2, 1.2 Hz, 1H), 2.43–2.26 (m, 7H), 2.04–1.79 (m, 3H), 1.28 (t, *J* = 7.1 Hz, 3H); ^{13}C NMR (101 MHz, $CDCl_3$) δ 171.3, 170.2, 138.1, 136.8, 135.0, 129.4, 124.3, 123.6, 69.1, 62.3, 54.4, 40.2, 28.1, 21.3, 20.6, 14.2; IR (Neat Film, NaCl) 2978, 2917, 1731, 1602, 1456, 1398, 1367, 1350, 1242, 1198, 1163, 1096, 1026, 972, 853, 759, 693, 638 cm^{-1}; HRMS (MM) *m/z* calc'd for $C_{19}H_{25}O_4$ [M+H]$^+$: 317.1747, found 317.1749; SFC Conditions: 5% IPA, 3.0 mL/min, Chiralpak AD-H column, λ = 254 nm, t_R (min): minor = 9.68, major = 11.56.

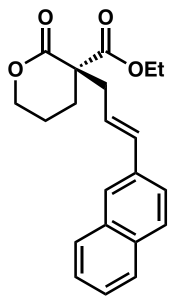

3ai

Ethyl (*R*,*E*)-3-(3-(naphthalen-2-yl)allyl)-2-oxotetrahydro-2*H*-pyran-3-carboxylate (3ai)

Product **3ai** was prepared using general procedure 3 and purified by column chromatography (15% EtOAc in hexanes) to provide a colorless oil (42.1 mg, 62% yield); 88% ee, $[\alpha]_D^{25}$ +27.34 (*c* 0.82, $CHCl_3$); ^{1}H NMR (400 MHz, $CDCl_3$) δ 7.84–7.73 (m, 3H), 7.72–7.67 (m, 1H), 7.57 (dd, *J* = 8.5, 1.8 Hz, 1H), 7.52–7.38 (m, 2H), 6.68–6.59 (m, 1H), 6.34 (ddd, *J* = 15.8, 8.0, 7.0 Hz, 1H), 4.30 (t, *J* = 5.8 Hz, 2H), 4.24 (q, *J* = 7.1 Hz, 2H), 2.96 (ddd, *J* = 13.7, 7.0, 1.4 Hz, 1H), 2.81 (ddd, *J* = 13.7, 8.0, 1.2 Hz, 1H), 2.48–2.34 (m, 1H), 2.03–1.81 (m, 3H), 1.28 (t, *J* = 7.1 Hz, 3H); ^{13}C NMR (101 MHz, $CDCl_3$) δ 171.2, 170.1, 134.8, 134.3, 133.6, 133.0, 128.2, 128.0, 127.7, 126.3, 126.1, 125.9, 124.5, 123.6, 69.0, 62.3, 54.4, 40.2, 28.1, 20.6, 14.2; IR (Neat Film, NaCl) 2980, 1732, 1597, 1507, 1456, 1399, 1367, 1243, 1198, 1097, 1023, 971, 896, 861, 815, 751, 667, 639, 624; HRMS (MM) *m/z* calc'd for $C_{21}H_{23}O_4$ [M+H]$^+$: 339.1591, found 339.1595; SFC Conditions 30% IPA, 2.5 mL/min, Chiralpak AD-H column λ = 254 nm, t_R (min): major = 3.36, minor = 4.24.

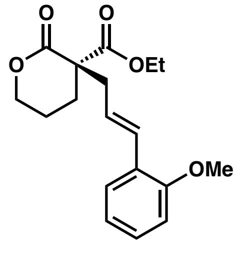

3aj

Ethyl (*R*,*E*)-3-(3-(2-methoxyphenyl)allyl)-2-oxotetrahydro-2*H*-pyran-3-carboxylate (3aj)

Product **3aj** was prepared using general procedure 3 and purified by column chromatography (15% EtOAc in hexanes) to provide a colorless oil (32.4 mg, 51% yield); 90% ee, $[\alpha]_D^{25}$ –11.96 (*c* 0.87, $CHCl_3$); ^{1}H NMR (400 MHz, $CDCl_3$) δ 7.40 (dd, *J* = 7.7, 1.7 Hz, 1H), 7.21 (ddd, *J* = 8.2, 7.4, 1.7 Hz, 1H), 6.90 (td, *J* = 7.6, 1.1 Hz, 1H), 6.88–6.75 (m, 2H), 6.16 (ddd, *J* = 15.9, 8.2, 6.9 Hz, 1H), 4.29 (dd, *J* = 6.2, 5.5 Hz, 2H), 4.23 (q, *J* = 7.1 Hz, 2H), 3.83 (s, 3H), 2.92 (ddd, *J* = 13.8, 6.8, 1.5 Hz, 1H), 2.77 (ddd, *J* = 13.7, 8.2, 1.2 Hz, 1H), 2.44–2.29 (m, 1H), 2.03–1.81 (m, 3H), 1.28 (t, *J* = 7.1 Hz, 3H); ^{13}C NMR (101 MHz, $CDCl_3$) δ 171.3, 170.2, 156.5, 129.5, 128.7, 126.8, 126.0, 124.5, 120.7, 110.9, 69.2, 62.2, 55.5, 54.4, 40.6, 28.1, 20.7, 14.2; IR (Neat Film, NaCl) 2978, 2838, 1732, 1598, 1489, 1464, 1399, 1244, 1198, 1163, 1104, 1051, 1027, 976, 858, 755, 641; HRMS (MM) *m/z* calc'd for $C_{18}H_{23}O_5$ $[M+H]^+$: 319.1540, found 319.1542; SFC Conditions 10% IPA, 2.5 mL/min, Chiralcel OD-H column λ = 254 nm, t_R (min): minor = 9.05, major = 9.85.

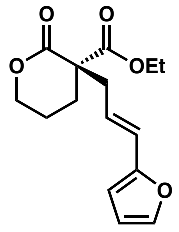

3ak

Ethyl (*R*,*E*)-3-(3-(furan-2-yl)allyl)-2-oxotetrahydro-2*H*-pyran-3-carboxylate (3ak)

Product **3ak** was prepared using general procedure 3 and purified by column chromatography (15% EtOAc in hexanes) to provide a colorless oil (45.5 mg, 82% yield); 88% ee, $[\alpha]_D^{25}$ –11.85 (*c* 0.99, $CHCl_3$); ^{1}H NMR (400 MHz, $CDCl_3$) δ 7.32–7.27 (m, 1H), 6.37–6.23 (m, 2H), 6.17 (d, *J* = 3.2 Hz, 1H), 6.14–6.01 (m, 1H), 4.29 (dd, *J* = 6.3, 5.5 Hz, 2H), 4.22 (q, *J* = 7.1 Hz, 2H), 2.86 (ddd, *J* = 13.9, 7.2, 1.3 Hz, 1H), 2.70 (ddd, *J* = 13.9, 8.0, 1.2 Hz, 1H), 2.40–2.29 (m, 1H), 2.05—

1.78 (m, 3H), 1.26 (t, *J* = 7.1 Hz, 3H); ^{13}C NMR (101 MHz, $CDCl_3$) δ 171.2, 170.0, 152.4, 141.9, 123.2, 122.7, 111.3, 107.6, 69.1, 62.3, 54.4, 39.8, 28.1, 20.6, 14.1; IR (Neat Film, NaCl) 2980, 1732, 1456, 1399, 1244, 1200, 1166, 1097, 1017, 969, 926, 858, 749, 640 ; HRMS (MM) *m/z* calc'd for $C_{15}H_{19}O_5$ $[M+H]^+$: 343.1329, found 343.1327; SFC Conditions 10% IPA, 2.5 mL/min, Chiralpak AD-H column λ = 254 nm, t_R (min): major = 3.97, minor = 4.62.

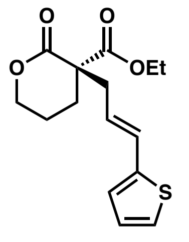

3al

Ethyl (*R,E*)-2-oxo-3-(3-(thiophen-2-yl)allyl)tetrahydro-2*H*-pyran-3-carboxylate (3al)

Product **3al** was prepared using general procedure 3 and purified by column chromatography (15% EtOAc in hexanes) to provide a colorless oil (39.9 mg, 68% yield); 88% ee, $[\alpha]_D^{25}$ –15.7 (*c* 0.98, $CHCl_3$); ^{1}H NMR (400 MHz, $CDCl_3$) δ 7.12 (dt, *J* = 4.9, 1.0 Hz, 1H), 6.97–6.87 (m, 2H), 6.59 (dtt, *J* = 15.7, 1.4, 0.6 Hz, 1H), 6.00 (ddd, *J* = 15.4, 8.0, 7.2 Hz, 1H), 4.29 (t, *J* = 5.9 Hz, 2H), 4.22 (q, *J* = 7.1 Hz, 2H), 2.86 (ddd, *J* = 13.9, 7.2, 1.4 Hz, 1H), 2.70 (ddd, *J* = 13.8, 8.0, 1.2 Hz, 1H), 2.42–2.29 (m, 1H), 2.06–1.80 (m, 3H), 1.27 (t, *J* = 7.1 Hz, 3H); ^{13}C NMR (101 MHz, $CDCl_3$) δ 171.2, 170.0, 142.0, 127.9, 127.4, 125.5, 124.2, 123.7, 69.1, 62.3, 54.4, 40.0, 28.2, 20.6, 14.2; IR (Neat Film, NaCl) 3107, 2980, 1731, 1446, 1367, 1348, 1244, 1199, 1165, 1096, 1024, 965, 855, 750, 704, 643; HRMS (MM) *m/z* calc'd for $C_{15}H_{19}O_4S$ $[M+H]^+$: 295.0999, found 295.0994; SFC Conditions 10% IPA, 2.5 mL/min, Chiralpak AD-H column λ = 254 nm, t_R (min): major = 6.33, minor = 7.51.

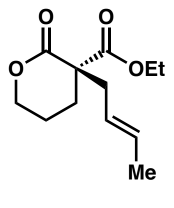

3am

Ethyl (*R*,*E*)-3-(but-2-en-1-yl)-2-oxotetrahydro-2*H*-pyran-3-carboxylate (3am)

Product **3am** was prepared using general procedure 3 and purified by column chromatography (15% EtOAc in hexanes) to provide a colorless oil (25.6 mg, 57% yield); 78% ee, $[\alpha]_D^{25}$ –0.22 (*c*

1.13, $CHCl_3$); ^{1}H NMR (400 MHz, $CDCl_3$) δ 5.55 (dqt, *J* = 15.0, 6.2, 1.1 Hz, 1H), 5.47–5.30 (m, 1H), 4.27 (t, *J* = 5.7 Hz, 2H), 4.20 (q, *J* = 7.1 Hz, 2H), 2.72–2.61 (m, 1H), 2.51 (ddt, *J* = 13.8, 7.7, 1.1 Hz, 1H), 2.35–2.26 (m, 1H), 2.02–1.90 (m, 1H), 1.90–1.78 (m, 2H), 1.65 (dq, *J* = 6.5, 1.2 Hz, 3H), 1.26 (t, *J* = 7.1 Hz, 3H); ^{13}C NMR (101 MHz, $CDCl_3$) δ 171.4, 170.2, 130.7, 124.9, 69.0, 62.1, 54.3, 39.7, 27.9, 20.6, 18.1, 14.2; IR (Neat Film, NaCl) 2965, 2938, 1730, 1447, 1400, 1272, 1223, 1198, 1163, 1107, 1077, 973, 856; HRMS (MM) *m/z* calc'd for $C_{12}H_{19}O_4$ $[M+H]^+$: 227.1278, found 227.1275; SFC Conditions 25% IPA, 2.5 mL/min, Chiralpak IC column λ = 210 nm, t_R (min): major = 2.87, minor = 3.69.

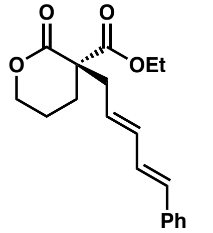

3an

Ethyl (*R*)-2-oxo-3-((2*E*,4*E*)-5-phenylpenta-2,4-dien-1-yl)tetrahydro-2*H*-pyran-3-carboxylate (3an)

Product **3an** was prepared using general procedure 3 and purified by column chromatography (15% EtOAc in hexanes) to provide a colorless oil (57.3 mg, 91% yield); 88% ee, $[\alpha]_D^{25}$ –22.45 (*c* 0.96, $CHCl_3$); ^{1}H NMR (400 MHz, $CDCl_3$) δ 7.39–7.35 (m, 2H), 7.30 (ddd, *J* = 7.7, 6.8, 1.2 Hz, 2H), 7.24–7.17 (m, 1H), 6.74 (ddd, *J* = 15.7, 10.4, 0.8 Hz, 1H), 6.49 (d, *J* = 15.7 Hz, 1H), 6.28 (ddq, *J* = 15.4, 10.5, 1.1 Hz, 1H), 5.83–5.69 (m, 1H), 4.29 (t, *J* = 5.8 Hz, 2H), 4.23 (q, *J* = 7.1 Hz, 2H), 2.84 (ddd, *J* = 13.9, 7.2, 1.3 Hz, 1H), 2.68 (ddd, *J* = 13.8, 8.1, 1.1 Hz, 1H), 2.41–2.26 (m, 1H), 2.03–1.80 (m, 3H), 1.28 (t, *J* = 7.1 Hz, 3H); ^{13}C NMR (101 MHz, $CDCl_3$) δ 171.2, 170.0, 137.2, 135.3, 132.1, 128.7, 128.5, 128.3, 127.6, 126.4, 69.0, 62.3, 54.4, 39.9, 28.1, 20.6, 14.2; IR (Neat Film, NaCl) 3058, 3024, 2980, 1732, 1490, 1478, 1448, 1400, 1367, 1347, 1241, 1198, 1097, 1025, 994, 910, 857, 750, 694, 667, 640; HRMS (MM) *m/z* calc'd for $C_{19}H_{23}O_4$ $[M+H]^+$: 315.1585, found 315.1585; SFC Conditions 15% IPA, 2.5 mL/min, Chiralpak AD-H column λ = 254 nm, t_R (min): major = 5.30, minor = 6.23.

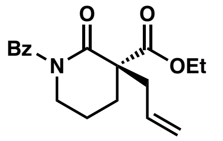

5aa

Ethyl (*S*)-3-allyl-1-benzoyl-2-oxopiperidine-3-carboxylate (5aa)

Product **5aa** was prepared using general procedure 4 and purified by column chromatography (15% EtOAc in hexanes) to provide a colorless oil (45.9 mg, 73% yield); 90% ee, $[\alpha]_D^{25}$ +42.42 (*c* 0.968, $CHCl_3$); ^{1}H NMR (400 MHz, $CDCl_3$) δ ^{1}H NMR (400 MHz, $CDCl_3$) δ 7.84–7.70 (m, 2H), 7.54–7.44 (m, 1H), 7.44–7.34 (m, 2H), 5.80–5.62 (m, 1H), 5.17–5.03 (m, 2H), 4.30 (q, *J* = 7.2 Hz, 2H), 3.84–3.71 (m, 2H), 2.72 (ddt, *J* = 13.8, 6.8, 1.2 Hz, 1H), 2.56 (ddt, *J* = 13.8, 7.9, 1.0 Hz, 1H), 2.43–2.25 (m, 1H), 2.04–1.83 (m, 3H), 1.36 (t, *J* = 7.1 Hz, 3H); ^{13}C NMR (101 MHz, $CDCl_3$) δ 175.1, 171.9, 171.8, 135.9, 133.0, 131.8, 128.2, 128.1, 119.7, 62.1, 56.4, 46.6, 40.0, 30.3, 20.3, 14.3; IR (Neat Film, NaCl) 3074, 2936, 2341, 1734, 1700, 1684, 1450, 1388, 1278, 1147, 1177, 1050, 1027, 919, 824, 726, 694, 668 cm^{-1}; HRMS (MM) *m/z* calc'd for $C_{18}H_{22}NO_4$ $[M+H]^+$: 316.1543, found 316.1543; SFC Conditions: 20% IPA, 2.5 mL/min, Chiralpak IC column, λ = 254 nm, t_R (min): major = 3.77, minor = 4.39.

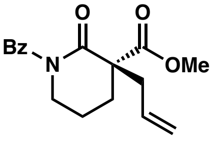

5ba

Methyl (*S*)-3-allyl-1-benzoyl-2-oxopiperidine-3-carboxylate (5ba)

Product **5ba** was prepared using general procedure 4 and purified by column chromatography (20% EtOAc in hexanes) to provide a colorless oil (51.0 mg, 85% yield); 90% ee, $[\alpha]_D^{25}$ +48.58 (*c* 0.890, $CHCl_3$); ^{1}H NMR (400 MHz, $CDCl_3$) δ 7.77–7.59 (m, 2H), 7.55–7.44 (m, 1H), 7.40 (ddt, *J* = 8.3, 6.6, 1.2 Hz, 2H), 5.84–5.63 (m, 1H), 5.20–5.02 (m, 2H), 3.83 (s, 3H), 3.77 (dd, *J* = 6.7, 5.4 Hz, 2H), 2.73 (ddt, *J* = 13.7, 6.8, 1.2 Hz, 1H), 2.57 (ddt, *J* = 13.7, 7.7, 1.1 Hz, 1H), 2.41–2.29 (m, 1H), 2.07–1.85 (m, 3H); ^{13}C NMR (101 MHz, $CDCl_3$) δ 175.1, 172.4, 171.8, 135.9, 133.0, 131.8, 128.2, 128.1, 119.8, 56.5, 52.9, 46.6, 39.9, 30.3, 20.2; IR (Neat Film, NaCl) 3075, 2953, 1738, 1702, 1683, 1640, 1583, 1478, 1449, 1436, 1349, 1277, 1252, 1177, 1147, 1078, 1052, 1027, 1001, 844, 819, 796, 726, 695, 651; HRMS (MM) *m/z* calc'd for $C_{17}H_{20}NO_4$

$[M+H]^+$: 302.1387, found 302.1377; SFC Conditions 10% IPA, 2.5 mL/min, Chiralpak AD-H column λ = 254 nm, t_R (min): minor = 3.96, major = 4.53.

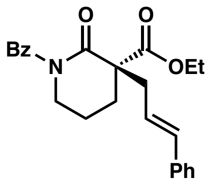

5ab

Ethyl (*S*)-1-benzoyl-3-cinnamyl-2-oxopiperidine-3-carboxylate (5ab)

Product **5ab** was prepared using general procedure 4 at 30 °C and purified by column chromatography (20% to 40% Et_2O in hexanes) to provide a colorless oil (58.2 mg, 74% yield); 90% ee, $[\alpha]_D^{25}$ +71.0 (*c* 0.88, $CHCl_3$); ^1H NMR (400 MHz, $CDCl_3$) δ 7.81–7.73 (m, 2H), 7.55–7.45 (m, 1H), 7.45–7.37 (m, 2H), 7.36–7.27 (m, 4H), 7.25–7.20 (m, 1H), 6.46 (dt, *J* = 15.7, 1.3 Hz, 1H), 6.14 (ddd, *J* = 15.8, 8.0, 6.9 Hz, 1H), 4.32 (q, *J* = 7.1 Hz, 2H), 3.86–3.73 (m, 2H), 2.91 (ddd, *J* = 13.8, 7.0, 1.4 Hz, 1H), 2.72 (ddd, *J* = 13.8, 8.0, 1.2 Hz, 1H), 2.49–2.35 (m, 1H), 2.10–1.91 (m, 3H), 1.37 (t, *J* = 7.1 Hz, 3H); ^{13}C NMR (101 MHz, $CDCl_3$) δ 175.0, 172.0, 171.9, 137.0, 135.9, 134.6, 131.9, 128.6, 128.2, 128.2, 127.6, 126.4, 124.5, 62.2, 56.9, 46.6, 39.3, 30.5, 20.3, 14.3; IR (Neat Film, NaCl) 2979, 1728, 1684, 1600, 1578, 1449, 1390, 1277, 1194, 1172, 1150, 1026, 970, 923, 934, 857, 822, 795, 745, 725, 694, 661 cm^{-1}; HRMS (MM) *m/z* calc'd for $C_{24}H_{26}NO_4$ $[M+H]^+$: 392.1856, found 392.1849; SFC Conditions: 30% IPA, 2.5 mL/min, Chiralpak AD-H column, λ = 254 nm, t_R (min): minor = 2.56, major = 2.95.

Experimental Procedures and Characterization Data for Product Transformations

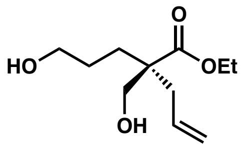

6

Ethyl (*S*)-2-(hydroxymethyl)-2-(3-hydroxypropyl)pent-4-enoate (6)

To a solution of allylated product **3aa** (42.5 mg, 0.2 mmol, 1 equiv) in 4:1 methanol:THF (1.4 mL), $CeCl_3{\cdot}7H_2O$ was added (149.0 mg, 0.4 mmol, 2 equiv). After cooling the reaction mixture at 0°C for 10 minutes, $NaBH_4$ (37.5 mg, 1.0 mmol, 5 equiv) was added in three portions over the course of 20 minutes. Additional methanol (1.5 mL) was added to rinse the side of the flask and the reaction mixture was stirred for another 10 minutes. The reaction was quenched with glacial acetic acid. The crude mixture was then concentrated under reduced pressure. The resultant residue was extracted with EtOAc, washed with $NaHCO_3$ and brine, dried over anhydrous $MgSO_4$, filtered, and purified by column chromatography (70% EtOAc in hexanes) to afford diol **6** as a colorless oil (54.1 mg, 88% yield). $[\alpha]_D^{25}$ +1.222 (*c* 0.92, $CHCl_3$); ^{1}H NMR (400 MHz, $CDCl_3$) δ 5.71 (ddt, *J* = 17.4, 10.1, 7.4 Hz, 1H), 5.14–4.99 (m, 2H), 4.15 (q, *J* = 7.1 Hz, 2H), 3.72–3.62 (m, 2H), 3.59 (td, *J* = 6.2, 1.6 Hz, 2H), 2.65 (br s, 2H), 2.38 (ddt, *J* = 14.0, 7.3, 1.2 Hz, 1H), 2.30 (ddt, *J* = 13.9, 7.5, 1.1 Hz, 1H), 1.75–1.58 (m, 2H), 1.58–1.42 (m, 2H), 1.25 (t, *J* = 7.1 Hz, 3H); ^{13}C NMR (101 MHz, $CDCl_3$) δ 176.0, 133.4, 118.6, 64.5, 62.9, 60.8, 50.8, 38.0, 29.3, 27.1, 14.4; IR (Neat Film, NaCl) 2281, 3078, 2940, 1725, 1641, 1465, 1447, 1372, 1329, 1300, 1219, 1191, 1138, 1112, 1053, 920, 862, 824, 782, 748, 679, 634; HRMS (MM) *m/z* calc'd for $C_{11}H_{21}O_4$ $[M+H]^+$: 217.1434, found 217.1427.

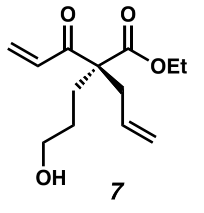

7

Ethyl (*S*)-2-allyl-2-(3-hydroxypropyl)-3-oxopent-4-enoate (7)

A 0.5 M solution of vinylmagnesium bromide in THF (0.3 mmol, 1.5 equiv) was added dropwise to a solution of allylated product **3aa** (42.5 mg, 0.2 mmol, 1 equiv) in THF (0.7 mL) at –78 °C

over 15 minutes. After 9 hours at –78 °C, the reaction was quenched with NH_4Cl. The mixture was diluted with EtOAc, washed with brine, and dried over anhydrous Na_2SO_4. Flash column chromatography (50% EtOAc in hexanes) of the crude residue afforded compound **7** as a colorless oil (80.0 mg, 67% yield); 86% ee, $[\alpha]_D^{25}$ –9.914 (*c* 0.798, $CHCl_3$); ^{1}H NMR (400 MHz, $CDCl_3$) δ 6.53 (dd, *J* = 16.9, 10.2 Hz, 1H), 6.39 (dd, *J* = 17.0, 1.8 Hz, 1H), 5.70 (dd, *J* = 10.1, 1.8 Hz, 1H), 5.57 (ddt, *J* = 16.8, 10.1, 7.4 Hz, 1H), 5.16–5.04 (m, 2H), 4.19 (qd, *J* = 7.1, 0.7 Hz, 2H), 3.62 (td, *J* = 6.4, 1.1 Hz, 2H), 2.79–2.55 (m, 2H), 2.04–1.82 (m, 2H), 1.51–1.30 (m, 3H), 1.23 (t, *J* = 7.1 Hz, 3H); ^{13}C NMR (101 MHz, $CDCl_3$) δ 195.5, 172.1, 132.2, 131.8, 129.5, 119.3, 62.9, 61.7, 61.6, 35.9, 27.5, 27.0, 14.2; IR (Neat Film, NaCl) 340, 3079, 2924, 1732, 1698, 1642, 1612, 1447, 1402, 1368, 1299, 1262, 1200, 1137, 1096, 1057, 1029, 983, 923, 856, 808, 739, 670, 686, 654; HRMS (MM) *m/z* calc'd for $C_{13}H_{21}O_4$ $[M+H]^+$: 241.1440, found 241.1443; SFC Conditions: 30% IPA, 2.5 mL/min, Chiralpak IC column, λ = 210 nm, t_R (min): major = 7.14, minor = 7.64.

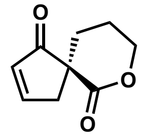

8

(*S*)-7-oxaspiro[4.5]dec-2-ene-1,6-dione (8)

Compound **7** (68.9 mg, 0.29 mmol, 1 equiv) in degassed toluene (3.0 mL) was added to a stirred solution of Grubbs' II catalyst (12.2 mg, 5 mol%) in toluene (15 mL) at 23 °C. After stirring at 40 °C for 4 hours under argon atmosphere, the dark brown solution was filtered through silica plug, flushed with acetone, and concentrated under vacuum. The crude residue was then redissolved in acetonitrile, 1,8-Diazabicyclo[5.4.0]undec-7-ene (DBU) was added (52 uL, 0.35 mmol, 1.2 equiv), and the reaction mixture was stirred at room temperature. Upon complete consumption of starting material by TLC, the reaction was quenched with NH_4Cl, extracted with EtOAc, washed with brine, dried over Na_2SO_4, filtered, and concentrated under vacuum. The crude residue was purified by column chromatography (30% acetone in hexanes) to provide spirocycle **8** as a colorless oil (25.6 mg, 53% yield). $[\alpha]_D^{25}$ –62.168 (*c* 0.75, $CHCl_3$); ^{1}H NMR (400 MHz, $CDCl_3$) δ 7.77 (dt, *J* = 5.6, 2.7 Hz, 1H), 6.14 (dt, *J* = 5.7, 2.2 Hz, 1H), 4.66–4.50 (m, 1H), 4.47–4.40 (m, 1H), 3.39 (dt, *J* = 18.9, 2.5 Hz, 1H), 2.58 (dt, *J* = 18.9, 2.4 Hz, 1H), 2.41–2.25 (m, 1H), 2.25–2.13 (m, 1H), 1.92–1.75 (m, 2H); ^{13}C NMR (101 MHz, $CDCl_3$) δ 206.2,

170.1, 163.8, 131.2, 71.0, 53.9, 44.5, 30.7, 20.4; IR (Neat Film, NaCl) 3082, 2932, 2871, 1728, 1699, 1592, 1422, 1403, 1343, 1272, 1217, 1160, 1108, 1080, 963, 816, 763; HRMS (MM) *m/z* calc'd for $C_9H_{11}O_3$ $[M+H]^+$: 167.0703, found 167.0696.

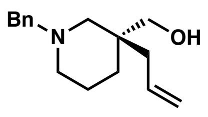

9

(*S*)-(3-allyl-1-benzylpiperidin-3-yl)methanol (9)

To a flame-dried microwave vial under argon was added lactam **5aa** (63 mg, 0.2 mmol) and dry diethyl ether (2.0 mL). Lithium aluminum hydride (91 mg, 2.4 mmol) was added slowly. The reaction was allowed to stir at room temperature for 10 minutes, after which it was sealed and heated to 65°C for 36 h. The reaction was quenched with water and 15% sodium hydroxide solution and extracted with ethyl acetate (5 mL × 4). The combined extracts were dried with Na_2SO_4, filtered, and concentrated under vacuum. The crude residue was purified by column chromatography (50% EtOAc in hexanes) to afford alcohol **9** as a colorless oil (39.3 mg, 80% yield). $[\alpha]_D^{25}$ +29.393 (*c* 0.965, $CHCl_3$); ^1H NMR (400 MHz, $CDCl_3$) δ 7.36–7.22 (m, 5H), 5.74 (ddt, *J* = 16.7, 10.4, 7.6 Hz, 1H), 5.06–4.95 (m, 2H), 3.63 (qd, *J* = 10.6, 1.6 Hz, 2H), 3.52–3.39 (m, 2H), 2.78–2.66 (m, 2H), 2.10–2.00 (m, 3H), 1.91 (d, *J* = 7.5 Hz, 2H), 1.69–1.54 (m, 2H), 1.36–1.19 (m, 2H); ^{13}C NMR (101 MHz, $CDCl_3$) δ 138.0, 133.9, 129.1, 128.5, 127.3, 117.8, 72.4, 63.5, 62.8, 54.0, 37.2, 33.2, 29.8, 23.0; IR (Neat Film, NaCl) 3392, 3065, 3028, 3003, 2932, 2858, 2797, 2759, 1949, 1822, 1730, 1638, 1586, 1603, 1586, 1553, 1494, 1466, 1453, 1415, 1392, 1370, 1352, 1311, 1300, 1259, 1248, 1208, 1180, 1162, 1127, 1116, 1072, 1045, 1028, 1045, 1001, 913, 875, 834, 810, 739, 699, 635, 619; HRMS (MM) *m/z* calc'd for $C_{16}H_{24}NO$ $[M+H]^+$: 246.1852, found 246.1847.

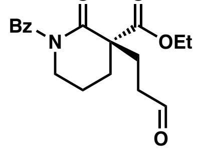

10

Ethyl (*S*)-1-benzoyl-2-oxo-3-(3-oxopropyl)piperidine-3-carboxylate (10)

To a flame dried vial was added $CuCl_2 \cdot H_2O$ (4.1 mg, 0.024 mmol), $PdCl_2(PhCN)_2$ (9.2 mg, 0.024 mmol), $AgNO_2$(1.9 mg, 0.012 mmol), *t*-BuOH (3.75 mL) and nitromethane (0.25 mL). The solution was sparged with O_2 for 15 minutes, and then neat lactam **5aa** (63.1 mg, 0.2 mmol) was added. The solution was then sparged for another 3 minutes and allowed to stir for 14 hours under an oxygen atmosphere. Upon reaction completion by TLC, water (4 mL) was added and the aqueous layer was extracted with DCM (4 mL × 3). The combined organic layers were dried with Na_2SO_4, filtered, and concentrated under reduced pressure. The product was purified by column chromatography (50% EtOAc in hexanes) to yield 75% of product **10**. $[\alpha]_D^{25}$ +3.159 (*c* 0.685, $CHCl_3$); ^{1}H NMR (400 MHz, $CDCl_3$) δ 9.69 (s, 1H), 7.78–7.69 (m, 2H), 7.52–7.44 (m, 1H), 7.44–7.35 (m, 2H), 4.38–4.24 (m, 2H), 3.89–3.70 (m, 2H), 2.73–2.59 (m, 1H), 2.55–2.38 (m, 2H), 2.23–2.13 (m, 2H), 2.06–1.91 (m, 2H), 1.82 (ddd, *J* = 13.6, 9.9, 5.4 Hz, 1H), 1.37 (t, *J* = 7.1 Hz, 3H); ^{13}C NMR (101 MHz, $CDCl_3$) δ 200.9, 175.0, 172.1, 171.9, 135.8, 132.0, 128.2, 128.2, 62.4, 55.8, 46.6, 39.9, 31.5, 27.8, 20.2, 14.3; IR (Neat Film, NaCl) 2924, 2853, 2727, 1723, 1704, 1681, 1601, 1449, 1391, 1348, 1275, 1195, 1174, 1150, 1062, 1023, 959, 916, 856, 824, 796, 726, 695, 659; HRMS (MM) *m/z* calc'd for $C_{18}H_{22}NO_5$ $[M+H]^+$: 332.1492, found 332.1483.

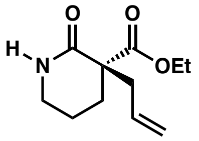

11

Ethyl (*S*)-3-allyl-2-oxopiperidine-3-carboxylate (11)[16]

To a flame dried vial under argon was added NaOEt (17.4 mg, 0.26 mmol) and ethanol (1.3 mL). Lactam **5aa** (63.1 mg, 0.20 mmol) was added and the resulting mixture was stirred for 48 h at 65 °C. The reaction was quenched with citric acid (154 mg, 0.80 mmol) and the EtOH was removed in vacuo. The resulting oil was then diluted with water (2 mL) and extracted with chloroform. The combined organic layers were dried with Na_2SO_4 and the solvent was removed in vacuo. The product was purified by column chromatography (80% EtOAc in hexanes) to afford amide **11** as a colorless oil (35.6 mg, 84% yield). $[\alpha]_D^{25}$ +36.162 (*c* 0.89, $CHCl_3$); ^{1}H NMR (400 MHz, $CDCl_3$) δ 6.40 (s, 1H), 5.76 (dddd, *J* = 16.8, 10.2, 8.1, 6.5 Hz, 1H), 5.20–5.05 (m, 2H), 4.29–4.10 (m, 2H), 3.40–3.18 (m, 2H), 2.78 (ddt, *J* = 13.8, 6.5, 1.3 Hz, 1H), 2.66–2.50 (m, 1H), 2.14–2.04

(m, 1H), 1.93–1.68 (m, 3H), 1.26 (t, *J* = 7.1 Hz, 3H); ^{13}C NMR (101 MHz, $CDCl_3$) δ 172.7, 170.8, 133.7, 119.2, 61.6, 53.5, 42.5, 40.0, 29.4, 19.6, 14.3; IR (Neat Film, NaCl) 3213, 3077, 2978, 2941, 2873, 1732, 1668, 1490, 1469, 1417, 1392, 1356, 1326, 1314, 1297, 1282, 1241, 1193, 1153, 1116, 1094, 1026, 1005, 921, 856, 812, 763, 719, 663; HRMS (MM) *m/z* calc'd for $C_{11}H_{18}NO_3$ $[M+H]^+$: 212.1281, found 212.1280.

Table S4. Determination of Enantiomeric Excess

entry	compound	SFC analytic conditions	ee (%)
1	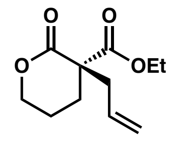 **3aa**	Chiralpak IC, λ = 210 nm<br>25% IPA/CO_2, 2.5 mL/min<br>t_R (min) major 2.66, minor 3.29	86
2	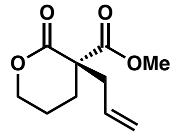 **3ba**	Chiralpak IC, λ = 210 nm<br>20% IPA/CO_2, 2.5 mL/min<br>t_R (min) major 3.35, minor 3.99	86
3	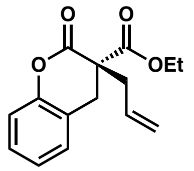 **3ca**	Chiracel OB-H, λ = 210 nm<br>5% IPA/CO_2, 2.5 mL/min<br>t_R (min) minor 2.22, major 2.64	64
4	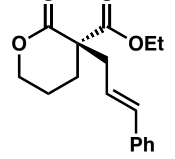 **3ab**	Chiralpak AD-H, λ = 254 nm<br>10% IPA/CO_2, 2.5 mL/min<br>t_R (min) major 5.49, minor 6.31	90
5	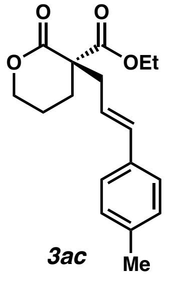 **3ac**	Chiralpak AD-H, λ = 254 nm<br>10% IPA/CO_2, 2.5 mL/min<br>t_R (min) major 6.47, minor 7.71	90

entry	compound	SFC analytic conditions	ee (%)
6	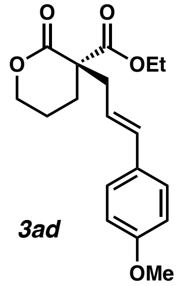 ***3ad***	Chiralpak AD-H, λ = 254 nm<br>15% IPA/CO_2, 2.5 mL/min<br>t_R (min) major 5.37, minor 6.37	88
7	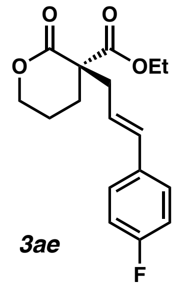 ***3ae***	Chiralpak AD-H, λ = 254 nm<br>10% IPA/CO_2, 2.5 mL/min<br>t_R (min) major 5.12, minor 5.95	88
8	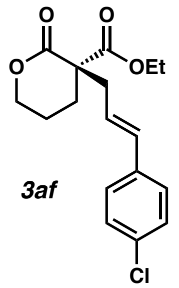 ***3af***	Chiralpak AD-H, λ = 254 nm<br>30% IPA/CO_2, 2.5 mL/min<br>t_R (min) major 2.29, minor 2.57	87
9	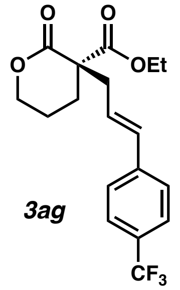 ***3ag***	Chiralpak AD-H, λ = 254 nm<br>10% IPA/CO_2, 2.5 mL/min<br>t_R (min) major 4.02, minor 4.72	86
10	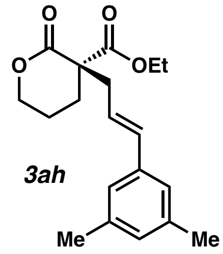 ***3ah***	Chiralpak AD-H, λ = 254 nm<br>5% IPA/CO_2, 3 mL/min<br>t_R (min) minor 9.68, major 11.56	88

entry	compound	SFC analytic conditions	ee (%)
11	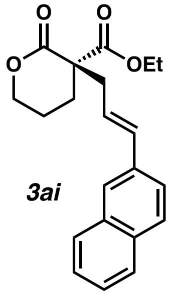 **3ai**	Chiralpak AD-H, λ = 254 nm<br>30% IPA/CO_2, 2.5 mL/min<br>t_R (min) major 3.36, minor 4.24	88
12	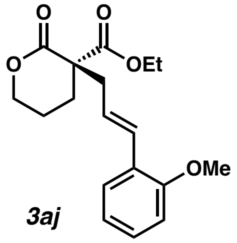 **3aj**	Chiralcel OD-H, λ = 254 nm<br>10% IPA/CO_2, 2.5 mL/min<br>t_R (min) minor 9.05, major 9.85	90
13	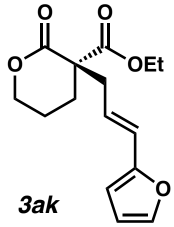 **3ak**	Chiralpak AD-H, λ = 254 nm<br>10% IPA/CO_2, 2.5 mL/min<br>t_R (min) major 3.97, minor 4.62	88
14	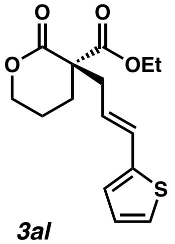 **3al**	Chiralpak AD-H, λ = 254 nm<br>10% IPA/CO_2, 2.5 mL/min<br>t_R (min) major 6.33, minor 7.51	88
15	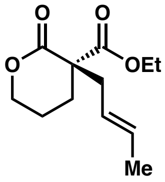 **3am**	Chiralpak IC, λ = 210 nm<br>25% IPA/CO_2, 2.5 mL/min<br>t_R (min) major 2.87, minor 3.69	78
16	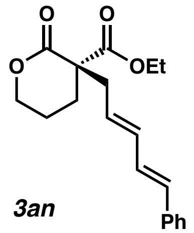 **3an**	Chiralpak AD-H, λ = 254 nm<br>15% IPA/CO_2, 2.5 mL/min<br>t_R (min) major 5.30, minor 6.23	88

entry	compound	SFC analytic conditions	ee (%)
17	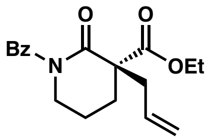 **5aa**	Chiralpak IC, λ = 254 nm<br>20% IPA/CO_2, 2.5 mL/min<br>t_R (min) major 3.77, minor 4.39	90
18	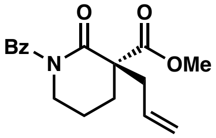 **5ba**	Chiralpak AD-H, λ = 254 nm<br>10% IPA/CO_2, 2.5 mL/min<br>t_R (min) minor 3.96 major 4.53	90
19	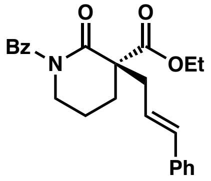 **5ab**	Chiralpak AD-H, λ = 254 nm<br>30% IPA/CO_2, 2.5 mL/min<br>t_R (min) minor 2.56, major 2.95	90
20	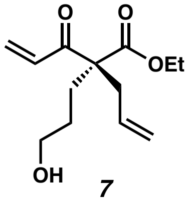 **7**	Chiralpak IC, λ = 210 nm<br>10% IPA/CO_2, 2.5 mL/min<br>t_R (min) major 7.14, minor 7.64	86

X-Ray Crystal Structure Data for Allylated Product 3af

The alpha-quaternary lactone product **3af** (87% ee) was crystallized from chloroform at –30 °C to provide crystals suitable for X-ray analysis.

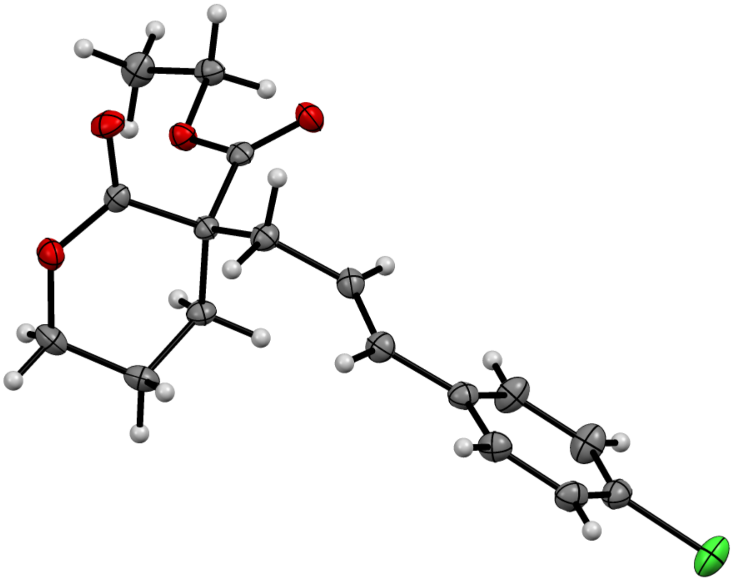

Table S5. Crystal data and structure refinement for 5am (P17471)

Identification code	P17471	
Empirical formula	C17 H19 Cl O4	
Formula weight	322.77	
Temperature	100(2) K	
Wavelength	1.54178 Å	
Crystal system	Orthorhombic	
Space group	$P2_12_12_1$	
Unit cell dimensions	a = 6.9832(6) Å	α= 90°.
	b = 8.5007(7) Å	β= 90°.
	c = 26.483(2) Å	γ = 90°.
Volume	1572.1(2) $Å^3$	
Z	4	
Density (calculated)	1.364 Mg/m^3	
Absorption coefficient	2.289 mm^{-1}	
F(000)	680	

Crystal size	0.300 x 0.150 x 0.050 mm^3
Theta range for data collection	3.337 to 74.260°.
Index ranges	-8<=h<=8, -10<=k<=10, -32<=l<=32
Reflections collected	25120
Independent reflections	3188 [R(int) = 0.0489]
Completeness to theta = 67.679°	100.0 %
Absorption correction	Semi-empirical from equivalents
Max. and min. transmission	0.7538 and 0.6272
Refinement method	Full-matrix least-squares on F^2
Data / restraints / parameters	3188 / 0 / 200
Goodness-of-fit on F^2	1.060
Final R indices [I>2sigma(I)]	R1 = 0.0260, wR2 = 0.0656
R indices (all data)	R1 = 0.0278, wR2 = 0.0664
Absolute structure parameter	0.061(4)
Extinction coefficient	n/a
Largest diff. peak and hole	0.227 and -0.175 e.$Å^{-3}$

Table S6. Atomic coordinates (x 10^4) and equivalent isotropic displacement parameters ($Å^2$x 10^3) for P17471. U(eq) is defined as one third of the trace of the orthogonalized U^{ij} tensor.

	x	y	z	U(eq)
O(1)	928(2)	7806(2)	4419(1)	23(1)
C(1)	835(2)	6277(2)	4310(1)	17(1)
O(2)	-713(2)	5637(2)	4313(1)	24(1)
C(2)	2622(2)	5380(2)	4140(1)	16(1)
C(3)	4511(2)	6200(2)	4290(1)	19(1)
C(4)	4363(3)	7952(2)	4186(1)	24(1)
C(5)	2756(3)	8620(2)	4498(1)	26(1)
C(6)	2541(2)	3772(2)	4398(1)	17(1)
O(3)	2596(2)	2523(1)	4186(1)	24(1)
C(7)	2528(3)	2501(2)	5202(1)	20(1)
O(4)	2462(2)	3939(1)	4901(1)	19(1)
C(8)	2567(3)	3008(2)	5747(1)	27(1)
C(9)	2380(3)	5189(2)	3561(1)	20(1)
C(10)	4143(3)	4629(2)	3291(1)	21(1)

C(11)	4812(3)	5296(2)	2874(1)	22(1)
C(12)	6521(2)	4825(2)	2586(1)	21(1)
C(13)	7937(3)	3834(3)	2784(1)	27(1)
C(14)	9558(3)	3457(3)	2508(1)	29(1)
C(15)	9789(3)	4068(2)	2030(1)	24(1)
Cl(1)	11856(1)	3606(1)	1692(1)	34(1)
C(16)	8423(3)	5039(2)	1816(1)	24(1)
C(17)	6799(3)	5404(2)	2097(1)	24(1)

Table S7. Bond lengths [Å] and angles [°] for P17471.

O(1)-C(1)	1.333(2)
O(1)-C(5)	1.467(2)
C(1)-O(2)	1.210(2)
C(1)-C(2)	1.530(2)
C(2)-C(6)	1.530(2)
C(2)-C(3)	1.543(2)
C(2)-C(9)	1.551(2)
C(3)-C(4)	1.518(3)
C(3)-H(3A)	0.9900
C(3)-H(3B)	0.9900
C(4)-C(5)	1.505(3)
C(4)-H(4A)	0.9900
C(4)-H(4B)	0.9900
C(5)-H(5A)	0.9900
C(5)-H(5B)	0.9900
C(6)-O(3)	1.202(2)
C(6)-O(4)	1.340(2)
C(7)-O(4)	1.459(2)
C(7)-C(8)	1.508(3)
C(7)-H(7A)	0.9900
C(7)-H(7B)	0.9900
C(8)-H(8A)	0.9800
C(8)-H(8B)	0.9800
C(8)-H(8C)	0.9800

C(9)-C(10)	1.502(2)
C(9)-H(9A)	0.9900
C(9)-H(9B)	0.9900
C(10)-C(11)	1.326(3)
C(10)-H(10)	0.9500
C(11)-C(12)	1.472(3)
C(11)-H(11)	0.9500
C(12)-C(17)	1.397(3)
C(12)-C(13)	1.401(3)
C(13)-C(14)	1.384(3)
C(13)-H(13)	0.9500
C(14)-C(15)	1.379(3)
C(14)-H(14)	0.9500
C(15)-C(16)	1.382(3)
C(15)-Cl(1)	1.7431(19)
C(16)-C(17)	1.392(3)
C(16)-H(16)	0.9500
C(17)-H(17)	0.9500

C(1)-O(1)-C(5)	122.26(14)
O(2)-C(1)-O(1)	118.72(16)
O(2)-C(1)-C(2)	120.46(16)
O(1)-C(1)-C(2)	120.61(15)
C(1)-C(2)-C(6)	106.47(13)
C(1)-C(2)-C(3)	113.40(14)
C(6)-C(2)-C(3)	108.67(14)
C(1)-C(2)-C(9)	104.69(14)
C(6)-C(2)-C(9)	110.14(14)
C(3)-C(2)-C(9)	113.22(14)
C(4)-C(3)-C(2)	109.75(14)
C(4)-C(3)-H(3A)	109.7
C(2)-C(3)-H(3A)	109.7
C(4)-C(3)-H(3B)	109.7
C(2)-C(3)-H(3B)	109.7
H(3A)-C(3)-H(3B)	108.2
C(5)-C(4)-C(3)	108.75(16)

C(5)-C(4)-H(4A)	109.9
C(3)-C(4)-H(4A)	109.9
C(5)-C(4)-H(4B)	109.9
C(3)-C(4)-H(4B)	109.9
H(4A)-C(4)-H(4B)	108.3
O(1)-C(5)-C(4)	113.07(15)
O(1)-C(5)-H(5A)	109.0
C(4)-C(5)-H(5A)	109.0
O(1)-C(5)-H(5B)	109.0
C(4)-C(5)-H(5B)	109.0
H(5A)-C(5)-H(5B)	107.8
O(3)-C(6)-O(4)	124.08(16)
O(3)-C(6)-C(2)	125.33(16)
O(4)-C(6)-C(2)	110.55(14)
O(4)-C(7)-C(8)	106.47(14)
O(4)-C(7)-H(7A)	110.4
C(8)-C(7)-H(7A)	110.4
O(4)-C(7)-H(7B)	110.4
C(8)-C(7)-H(7B)	110.4
H(7A)-C(7)-H(7B)	108.6
C(6)-O(4)-C(7)	116.84(13)
C(7)-C(8)-H(8A)	109.5
C(7)-C(8)-H(8B)	109.5
H(8A)-C(8)-H(8B)	109.5
C(7)-C(8)-H(8C)	109.5
H(8A)-C(8)-H(8C)	109.5
H(8B)-C(8)-H(8C)	109.5
C(10)-C(9)-C(2)	114.54(15)
C(10)-C(9)-H(9A)	108.6
C(2)-C(9)-H(9A)	108.6
C(10)-C(9)-H(9B)	108.6
C(2)-C(9)-H(9B)	108.6
H(9A)-C(9)-H(9B)	107.6
C(11)-C(10)-C(9)	123.40(17)
C(11)-C(10)-H(10)	118.3
C(9)-C(10)-H(10)	118.3

C(10)-C(11)-C(12)	126.94(18)
C(10)-C(11)-H(11)	116.5
C(12)-C(11)-H(11)	116.5
C(17)-C(12)-C(13)	117.39(17)
C(17)-C(12)-C(11)	119.79(17)
C(13)-C(12)-C(11)	122.81(17)
C(14)-C(13)-C(12)	121.30(18)
C(14)-C(13)-H(13)	119.3
C(12)-C(13)-H(13)	119.3
C(15)-C(14)-C(13)	119.48(18)
C(15)-C(14)-H(14)	120.3
C(13)-C(14)-H(14)	120.3
C(14)-C(15)-C(16)	121.37(18)
C(14)-C(15)-Cl(1)	118.91(15)
C(16)-C(15)-Cl(1)	119.72(15)
C(15)-C(16)-C(17)	118.44(17)
C(15)-C(16)-H(16)	120.8
C(17)-C(16)-H(16)	120.8
C(16)-C(17)-C(12)	122.01(18)
C(16)-C(17)-H(17)	119.0
C(12)-C(17)-H(17)	119.0

Symmetry transformations used to generate equivalent atoms:

Table S8. Anisotropic displacement parameters (Å^{2}x 10^3) for P17471. The anisotropic displacement factor exponent takes the form: -2p^2[h^2 a*^{2}U^{11} + ... + 2 h k a* b* U^{12}]

	U^{11}	U^{22}	U^{33}	U^{23}	U^{13}	U^{12}
O(1)	22(1)	19(1)	28(1)	-3(1)	0(1)	4(1)
C(1)	16(1)	19(1)	17(1)	2(1)	0(1)	2(1)
O(2)	15(1)	27(1)	32(1)	4(1)	1(1)	0(1)
C(2)	13(1)	16(1)	19(1)	-1(1)	-1(1)	0(1)
C(3)	15(1)	21(1)	22(1)	-1(1)	-1(1)	-2(1)
C(4)	25(1)	19(1)	29(1)	2(1)	-2(1)	-7(1)
C(5)	31(1)	16(1)	30(1)	-4(1)	-2(1)	-4(1)

C(6)	11(1)	18(1)	23(1)	0(1)	-1(1)	-1(1)
O(3)	31(1)	16(1)	27(1)	-3(1)	2(1)	-1(1)
C(7)	17(1)	17(1)	26(1)	6(1)	0(1)	2(1)
O(4)	22(1)	16(1)	20(1)	2(1)	0(1)	0(1)
C(8)	28(1)	28(1)	24(1)	6(1)	1(1)	6(1)
C(9)	17(1)	22(1)	21(1)	0(1)	-1(1)	0(1)
C(10)	20(1)	21(1)	22(1)	-3(1)	-1(1)	2(1)
C(11)	20(1)	22(1)	24(1)	1(1)	-2(1)	2(1)
C(12)	20(1)	22(1)	21(1)	-1(1)	-1(1)	-3(1)
C(13)	24(1)	38(1)	18(1)	4(1)	0(1)	3(1)
C(14)	24(1)	40(1)	24(1)	2(1)	-3(1)	7(1)
C(15)	21(1)	27(1)	23(1)	-4(1)	1(1)	-3(1)
Cl(1)	27(1)	48(1)	28(1)	-1(1)	8(1)	5(1)
C(16)	27(1)	27(1)	19(1)	2(1)	1(1)	-4(1)
C(17)	24(1)	24(1)	24(1)	4(1)	-2(1)	0(1)

Table S9. Hydrogen coordinates (x 10^4) and isotropic displacement parameters (Å^{2}x 10 ^{3}) for P17471.

	x	y	z	U(eq)
H(3A)	4771	6023	4653	23
H(3B)	5585	5747	4093	23
H(4A)	5583	8477	4274	29
H(4B)	4108	8133	3822	29
H(5A)	3106	8551	4859	31
H(5B)	2594	9746	4413	31
H(7A)	3689	1884	5119	24
H(7B)	1387	1843	5134	24
H(8A)	3645	3726	5802	40
H(8B)	2714	2082	5964	40
H(8C)	1367	3547	5830	40
H(9A)	1329	4433	3497	24
H(9B)	1991	6214	3417	24

H(10)	4813	3751	3425	25
H(11)	4115	6171	2748	26
H(13)	7782	3413	3113	32
H(14)	10505	2782	2648	35
H(16)	8589	5447	1485	29
H(17)	5851	6067	1953	28

Table S10. Torsion angles [°] for P17471.

C(5)-O(1)-C(1)-O(2)	170.67(16)
C(5)-O(1)-C(1)-C(2)	-14.5(2)
O(2)-C(1)-C(2)-C(6)	-45.3(2)
O(1)-C(1)-C(2)-C(6)	139.99(16)
O(2)-C(1)-C(2)-C(3)	-164.76(17)
O(1)-C(1)-C(2)-C(3)	20.5(2)
O(2)-C(1)-C(2)-C(9)	71.34(19)
O(1)-C(1)-C(2)-C(9)	-103.36(18)
C(1)-C(2)-C(3)-C(4)	-43.6(2)
C(6)-C(2)-C(3)-C(4)	-161.81(15)
C(9)-C(2)-C(3)-C(4)	75.47(19)
C(2)-C(3)-C(4)-C(5)	60.8(2)
C(1)-O(1)-C(5)-C(4)	32.0(2)
C(3)-C(4)-C(5)-O(1)	-54.7(2)
C(1)-C(2)-C(6)-O(3)	124.19(18)
C(3)-C(2)-C(6)-O(3)	-113.33(19)
C(9)-C(2)-C(6)-O(3)	11.2(2)
C(1)-C(2)-C(6)-O(4)	-57.84(17)
C(3)-C(2)-C(6)-O(4)	64.64(18)
C(9)-C(2)-C(6)-O(4)	-170.80(13)
O(3)-C(6)-O(4)-C(7)	2.6(2)
C(2)-C(6)-O(4)-C(7)	-175.38(13)
C(8)-C(7)-O(4)-C(6)	175.89(14)
C(1)-C(2)-C(9)-C(10)	168.24(15)
C(6)-C(2)-C(9)-C(10)	-77.66(18)
C(3)-C(2)-C(9)-C(10)	44.2(2)

C(2)-C(9)-C(10)-C(11)	-132.25(19)
C(9)-C(10)-C(11)-C(12)	-179.98(17)
C(10)-C(11)-C(12)-C(17)	165.5(2)
C(10)-C(11)-C(12)-C(13)	-16.1(3)
C(17)-C(12)-C(13)-C(14)	0.7(3)
C(11)-C(12)-C(13)-C(14)	-177.81(19)
C(12)-C(13)-C(14)-C(15)	0.1(3)
C(13)-C(14)-C(15)-C(16)	-0.7(3)
C(13)-C(14)-C(15)-Cl(1)	179.04(16)
C(14)-C(15)-C(16)-C(17)	0.6(3)
Cl(1)-C(15)-C(16)-C(17)	-179.20(15)
C(15)-C(16)-C(17)-C(12)	0.2(3)
C(13)-C(12)-C(17)-C(16)	-0.8(3)
C(11)-C(12)-C(17)-C(16)	177.69(18)

Symmetry transformations used to generate equivalent atoms:

References:
[1] G. M. Sheldrick, *Acta Cryst.* **1990**, A46, 467.
[2] G. M. Sheldrick, *Acta Cryst.* **2015**, C71, 3.
[3] P. Müller, *Crystallography Reviews* **2009**, *15*, 57.
[4] Procedure adapted from: P. Jakubec, A. J. M. Farley, D. J. Dixon, *Beilstein J. Org. Chem.* **2016**, 12, 1096.
[5] (a) M. Szosak, M. Spain, K. A. Choquette, R. A. Flowers II, D. J. Procter, *J. Am. Chem. Soc.* **2013**, 135, 15702; (b) D. Parmar, L. A. Duffy, D. V. Sadasivam, H. Matsubara, P. A. Bradley, R. A. Flowers II, D. J. Procter, *J. Am. Chem. Soc.* **2009**, 131, 15467.
[6] S. Suljić, J. Pietruszka, *Adv. Synth. Catal.* **2014**, 356, 1007.
[7] Adapted from synthesis of a similar compound, see: D. C. Behenna, Y. Liu, T. Yurino, J. Kim, D. E. White, S. C. Virgil, B. M. Stoltz, *Nature Chem*. **2012**, 4, 130.
[8] (a) A. Filippis, D. G. Pardo, J. Cossy, *Synthesis*. **2004**, 17, 2930; (b) C. J. Foti, D. L., *J. Org. Chem.* **1995**, 60, 2656.
[9] E. C. Garnier, L. S. Liebeskind, *J. Am. Chem. Soc.* **2008**, 130, 7449
[10] J. Z. Deng, D. V. Paone, A. T. Ginnetti, H. Kurihara, S. D. Dreher, S. A. Weissman, S. R. Stauffer, C. S. Burgey, *Org. Lett.* **2009**, 11, 345.
[11] P. Sauerberg, J. W. Kindtler, L. Nielsen, M. J. Sheardown, T. Honoré *J. Med. Chem.* **1991**, 34, 687.
[12] A. Loreto, A. Migliorini, P. A. Tardella, A. Gambacorta. *Eur. J. Org. Chem.* **2007**, 14, 2365.
[13] R. Hosseinzadeh, M. Tajbakhsh, M. Mohadjerani, H. Mehdinejad, *Synlett*. **2004**, 9, 1517
[14] J. Cossy, A. de Filippis, D. Gomez Pardo, *Org. Lett.* **2003**, 5, 3037.
[15] N. Hoshiya, K. Takenaka, S. Shuto. J. Uenishi, *Org. Lett.* **2016**, 18, 48.
[16] Adapted from: S. G. Davies, J. R. Haggitt, O. Ichihara, R. J. Kelly, M. A. Leech, A. J. P. Mortimer, P. M. Roberts, A. D. Smith, *Org. Biomol. Chem.* **2004**, 2, 2630.

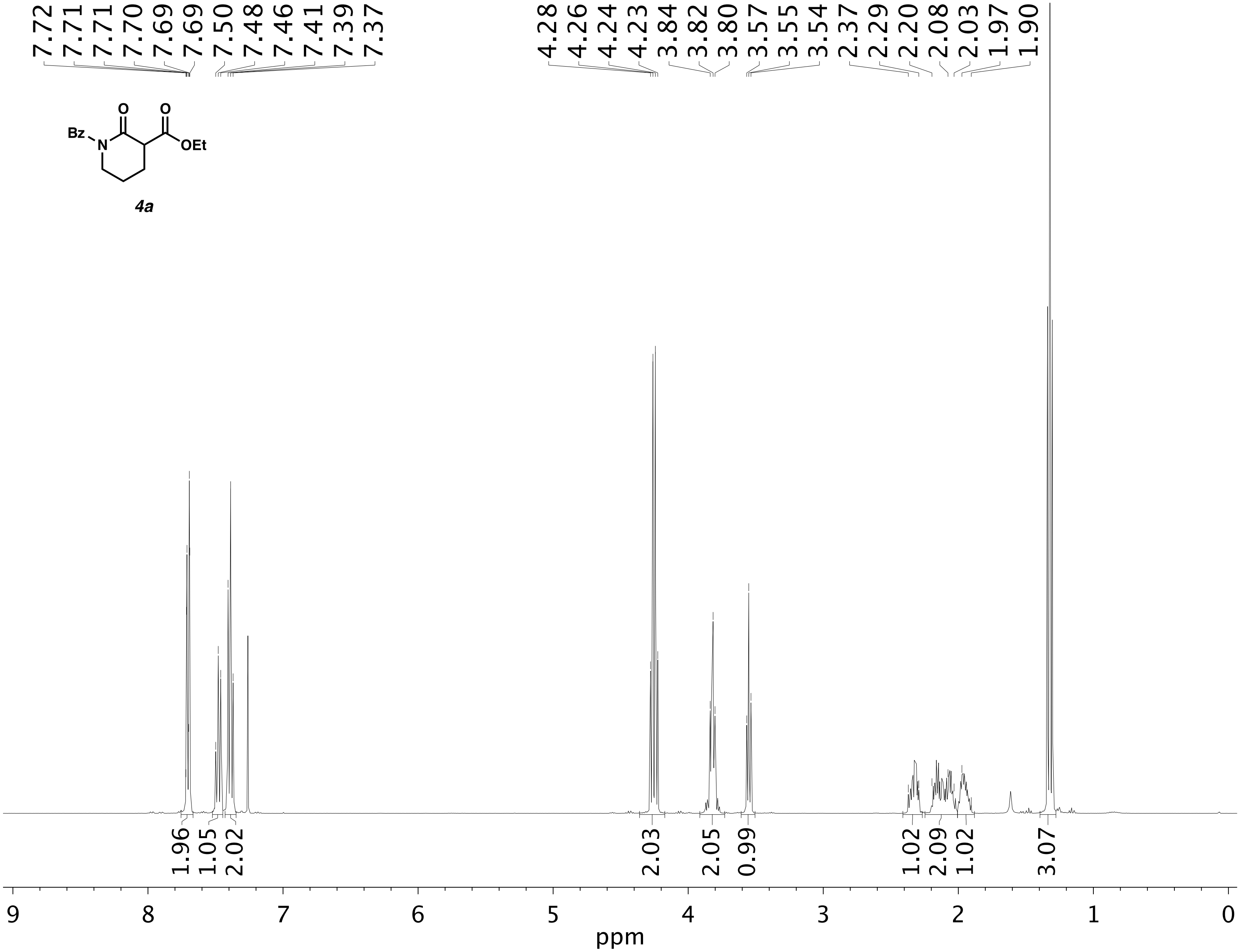

^{1}H NMR (400 MHz, $CDCl_3$) of compound **4a**.

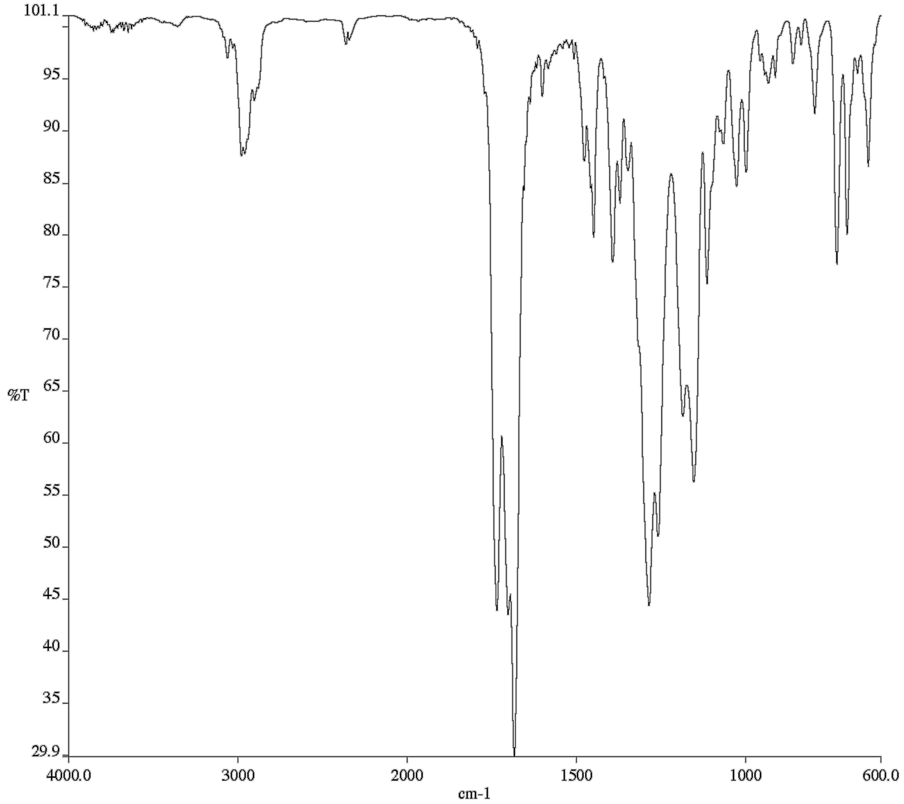

Infrared spectrum (Thin Film, NaCl) of compound **4a**.

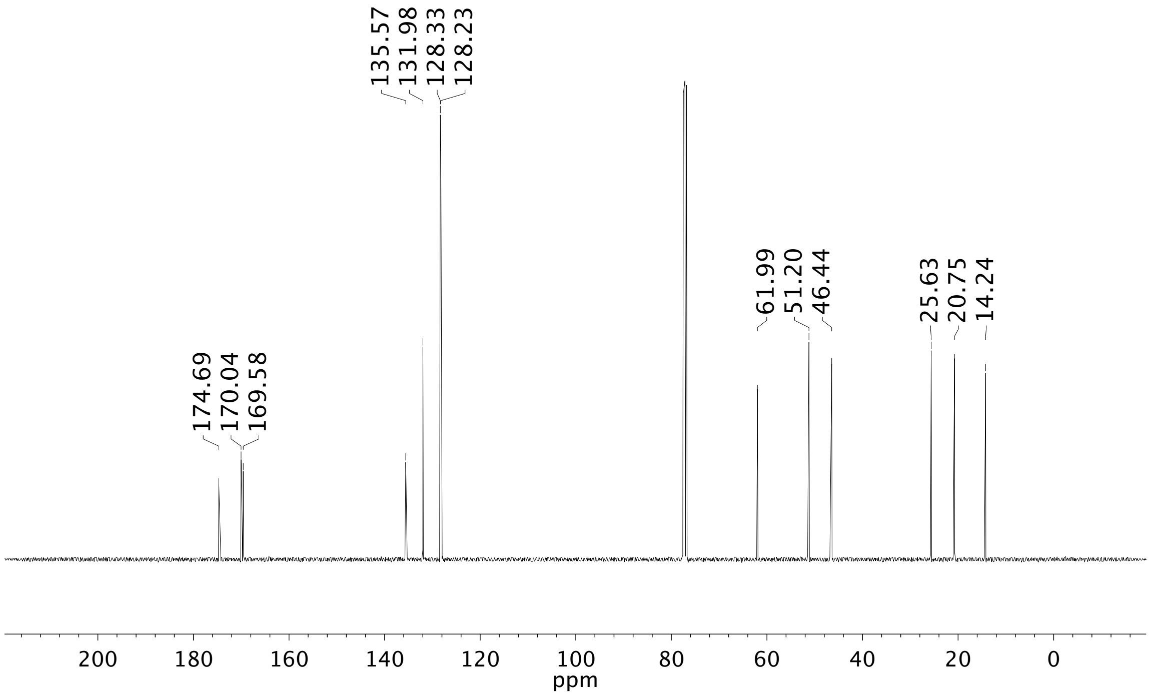

^{13}C NMR (101 MHz, $CDCl_3$) of compound **4a**.

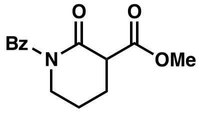

4b

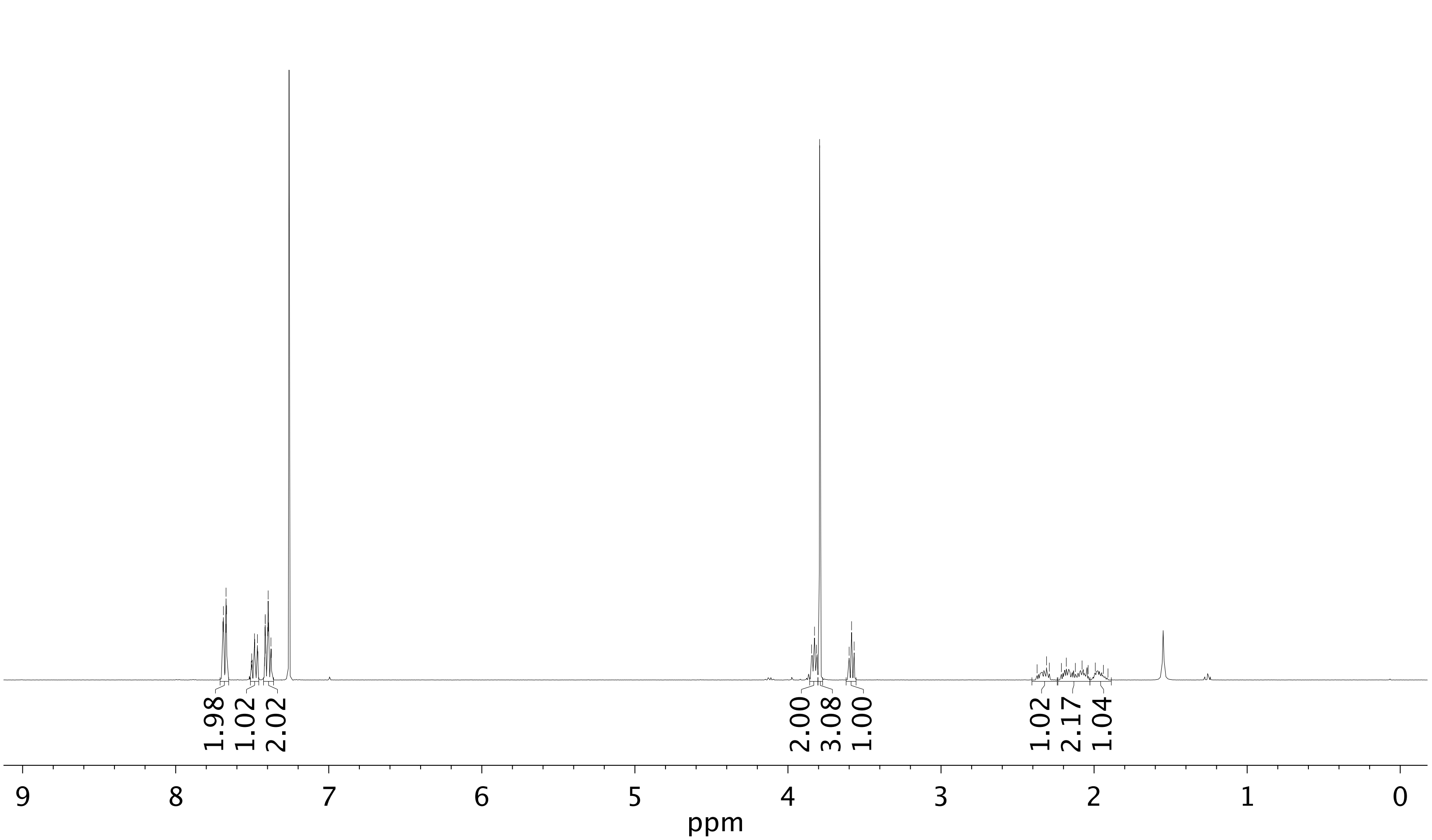

^1H NMR (400 MHz, $CDCl_3$) of compound **4b**.

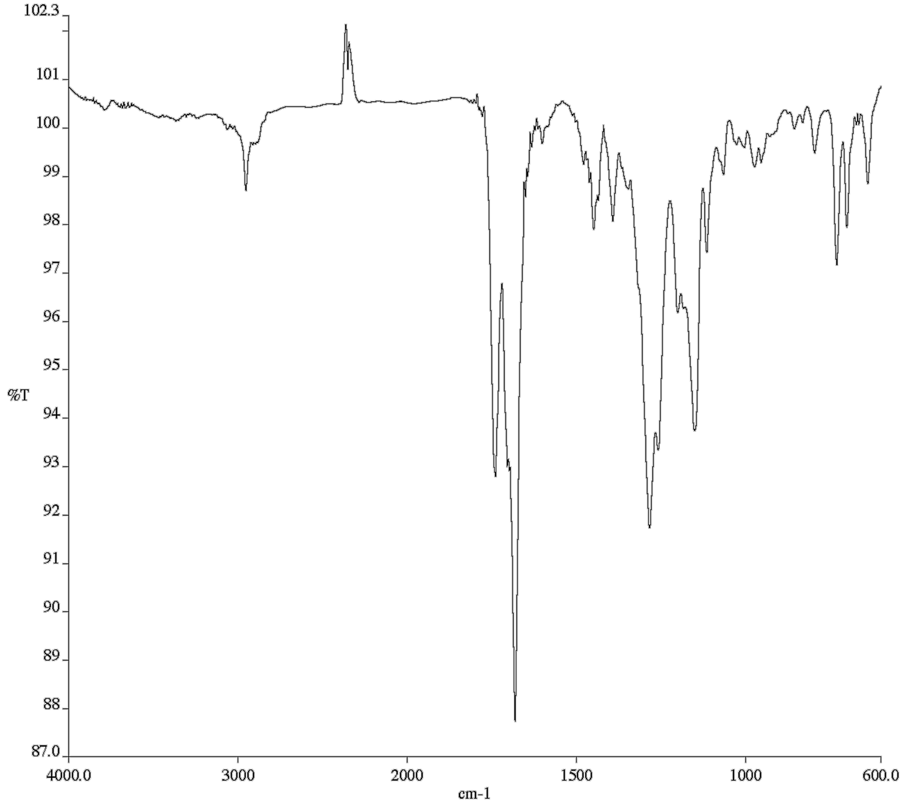

Infrared spectrum (Thin Film, NaCl) of compound **4b**.

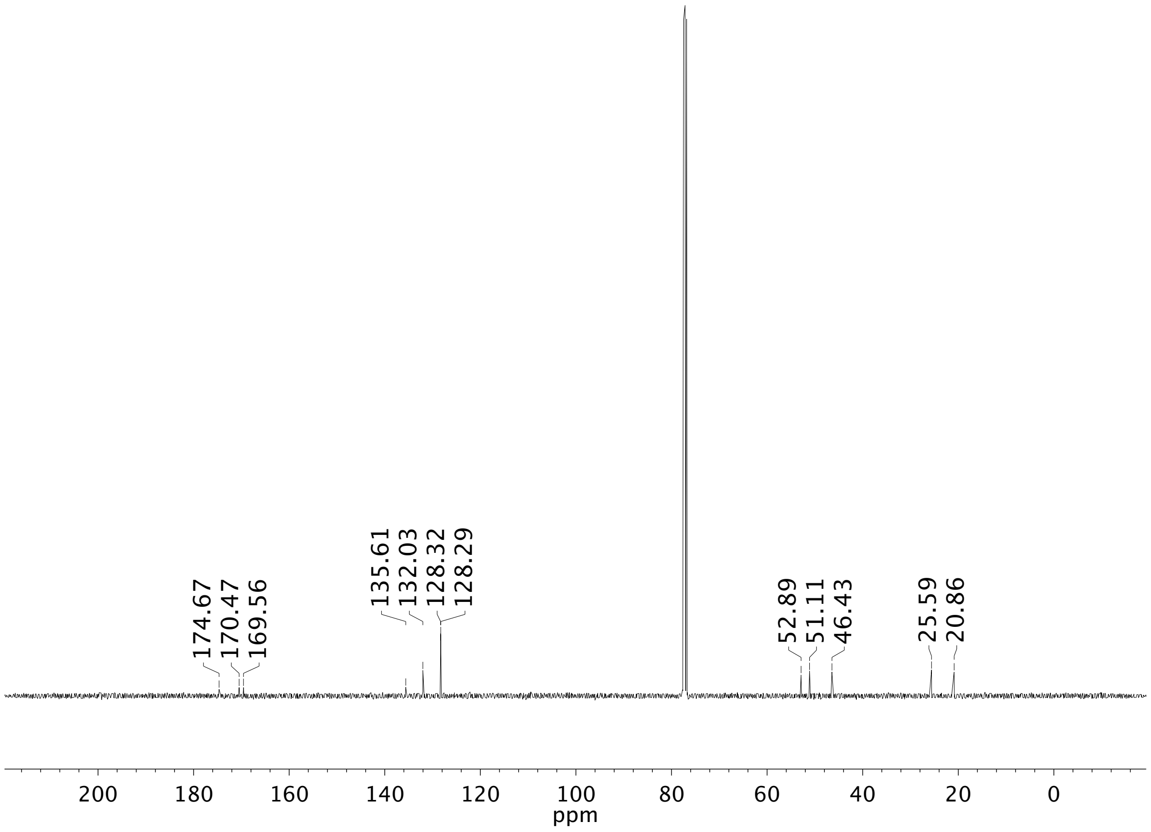

^{13}C NMR (101 MHz, $CDCl_3$) of compound **4b**.

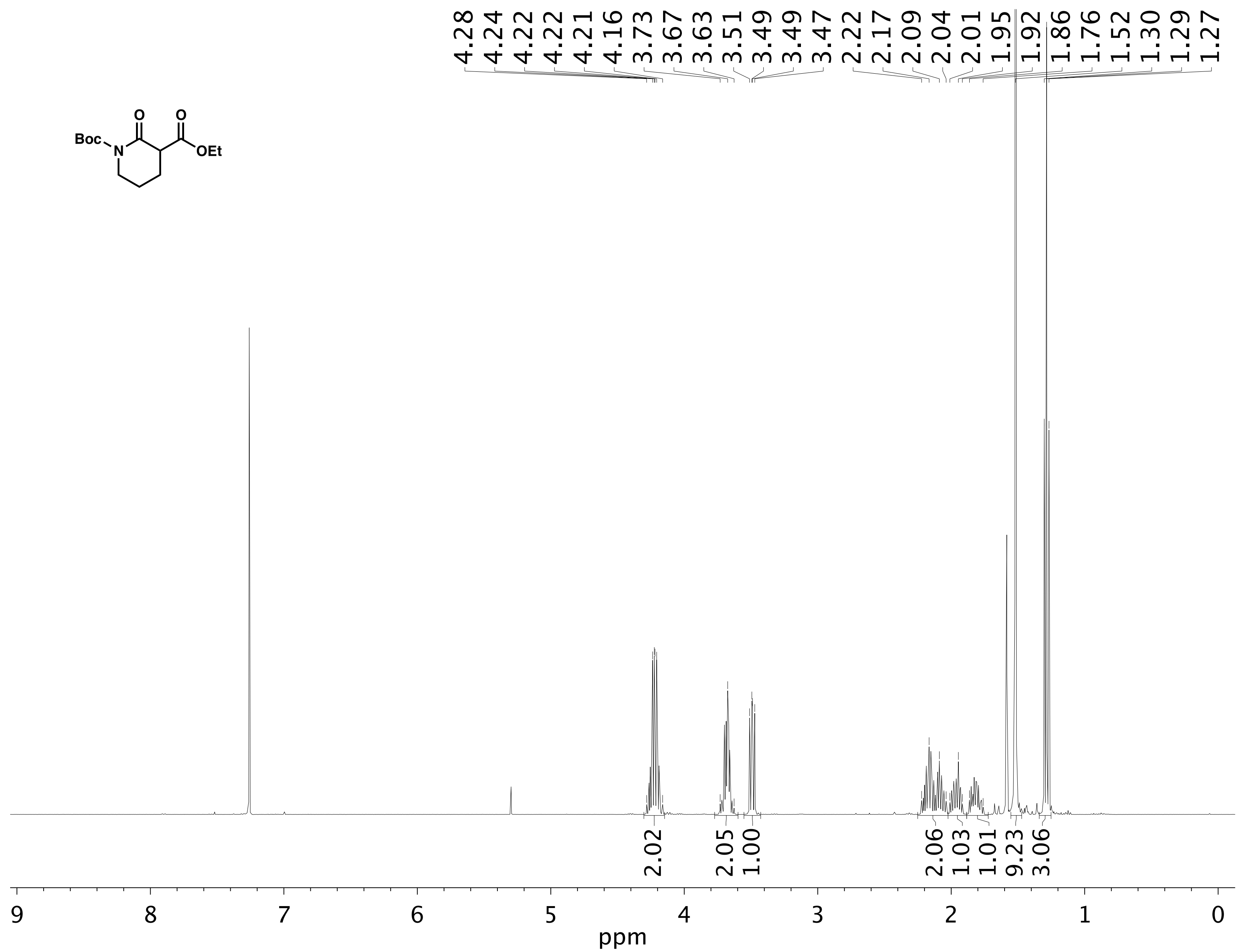

^{1}H NMR (400 MHz, $CDCl_3$) of Boc-protected lactam.

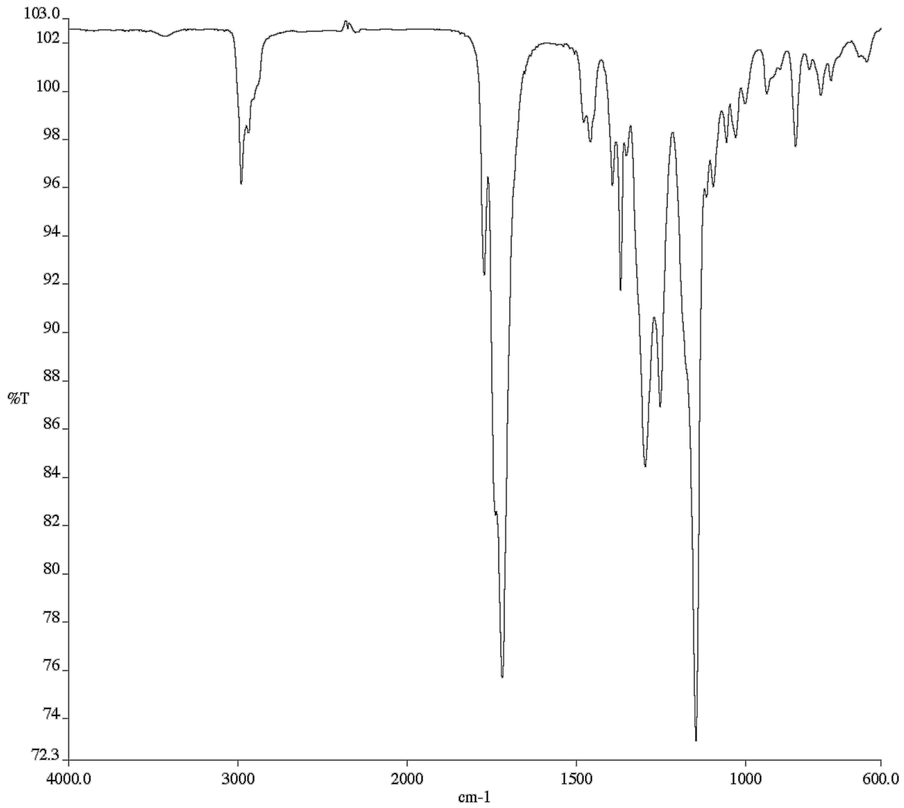

Infrared spectrum (Thin Film, NaCl) of Boc-protected lactam.

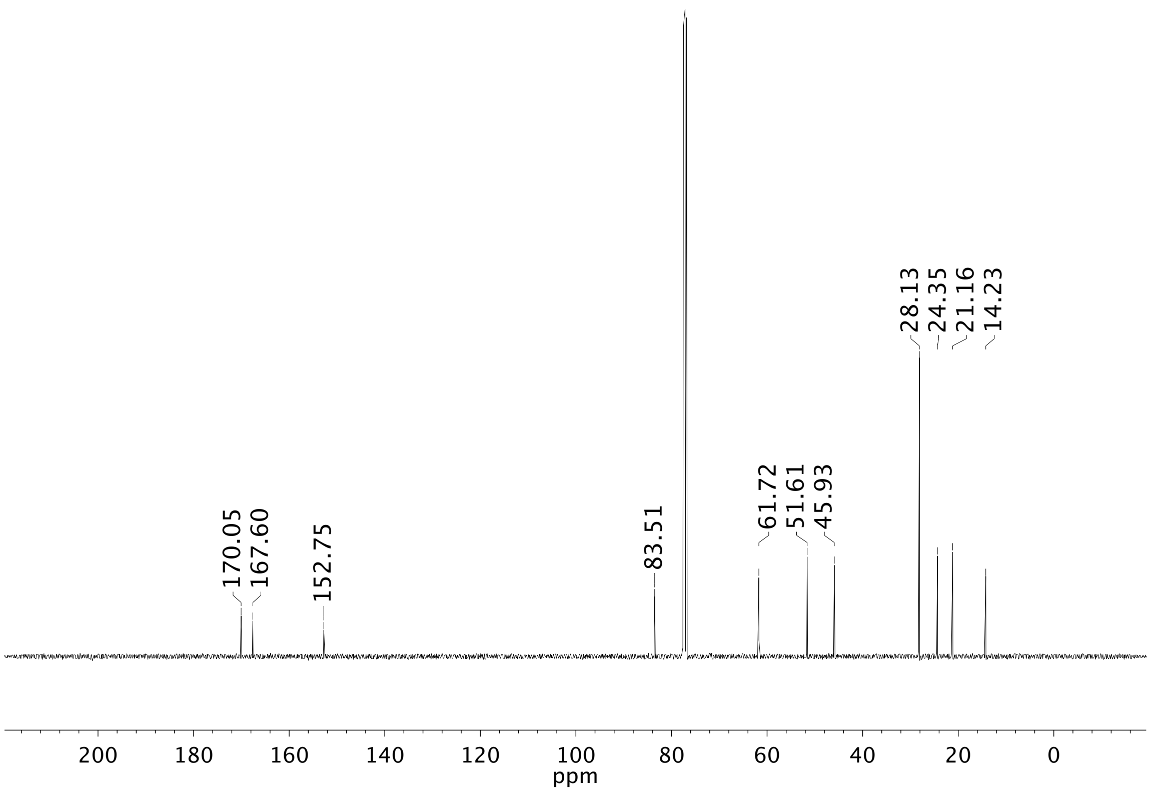

^{13}C NMR (101 MHz, $CDCl_3$) of Boc-protected lactam.

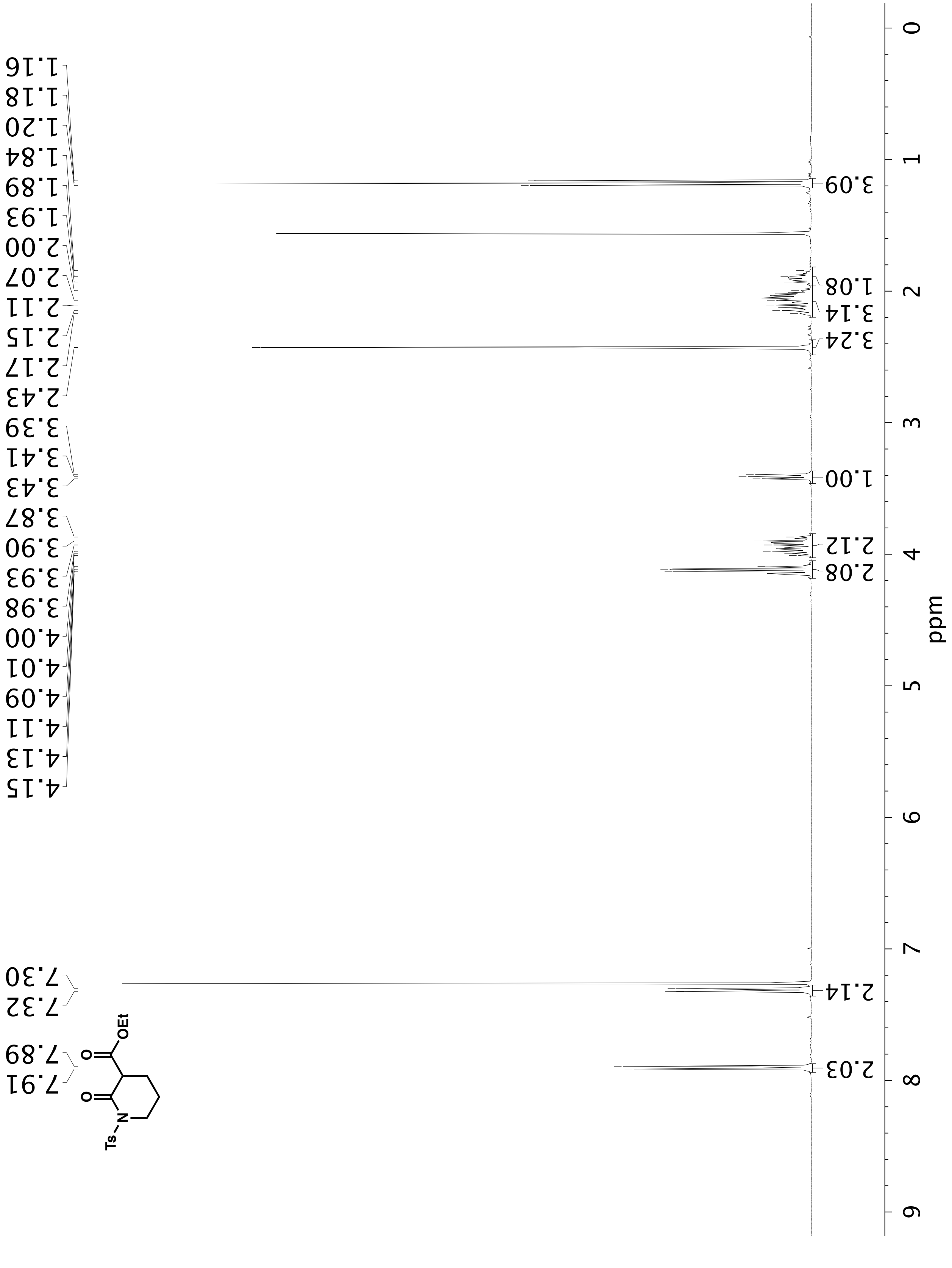

^{1}H NMR (400 MHz, $CDCl_3$) of Ts-protected lactam.

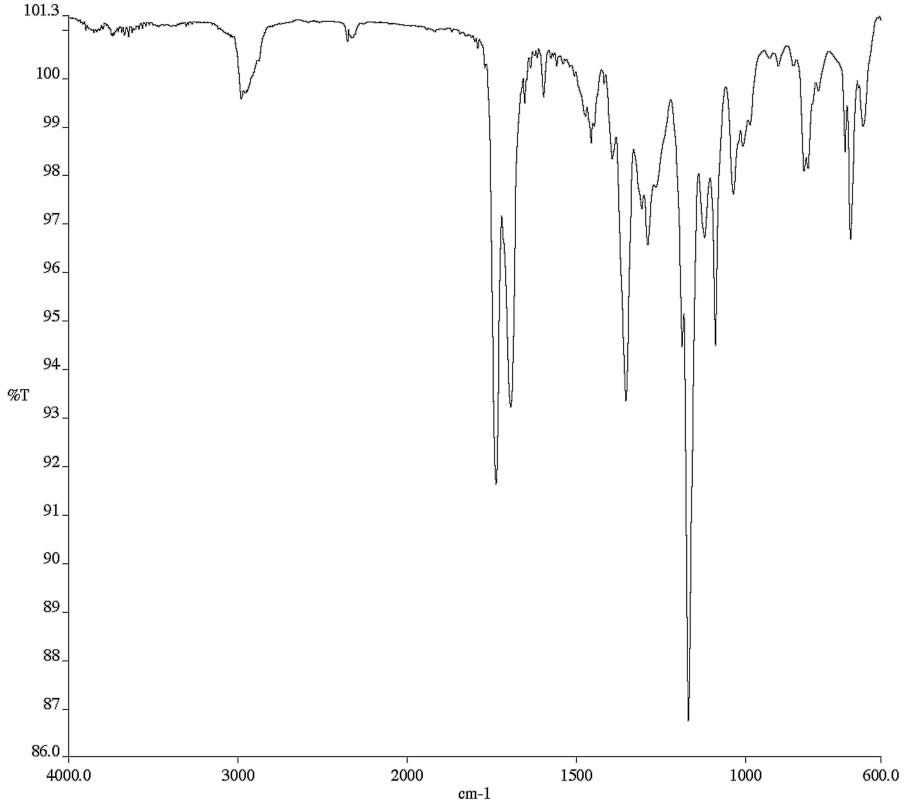

Infrared spectrum (Thin Film, NaCl) of Ts-protected lactam.

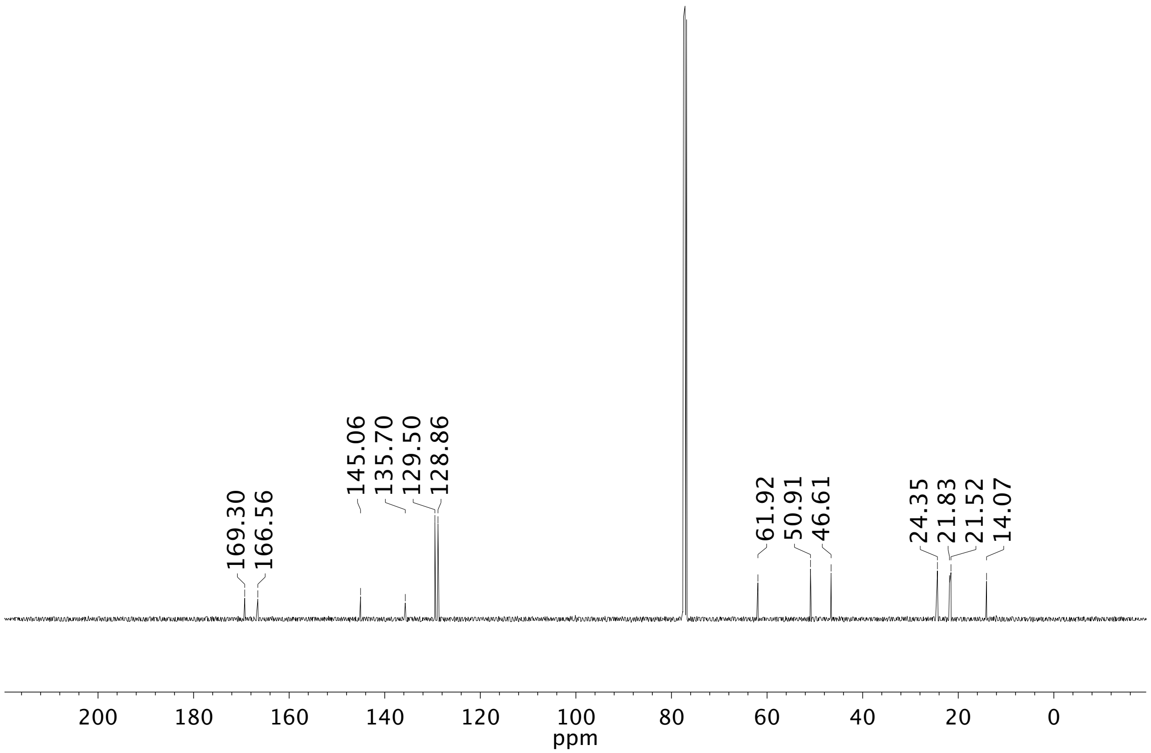

^{13}C NMR (101 MHz, $CDCl_3$) of Ts-protected lactam.

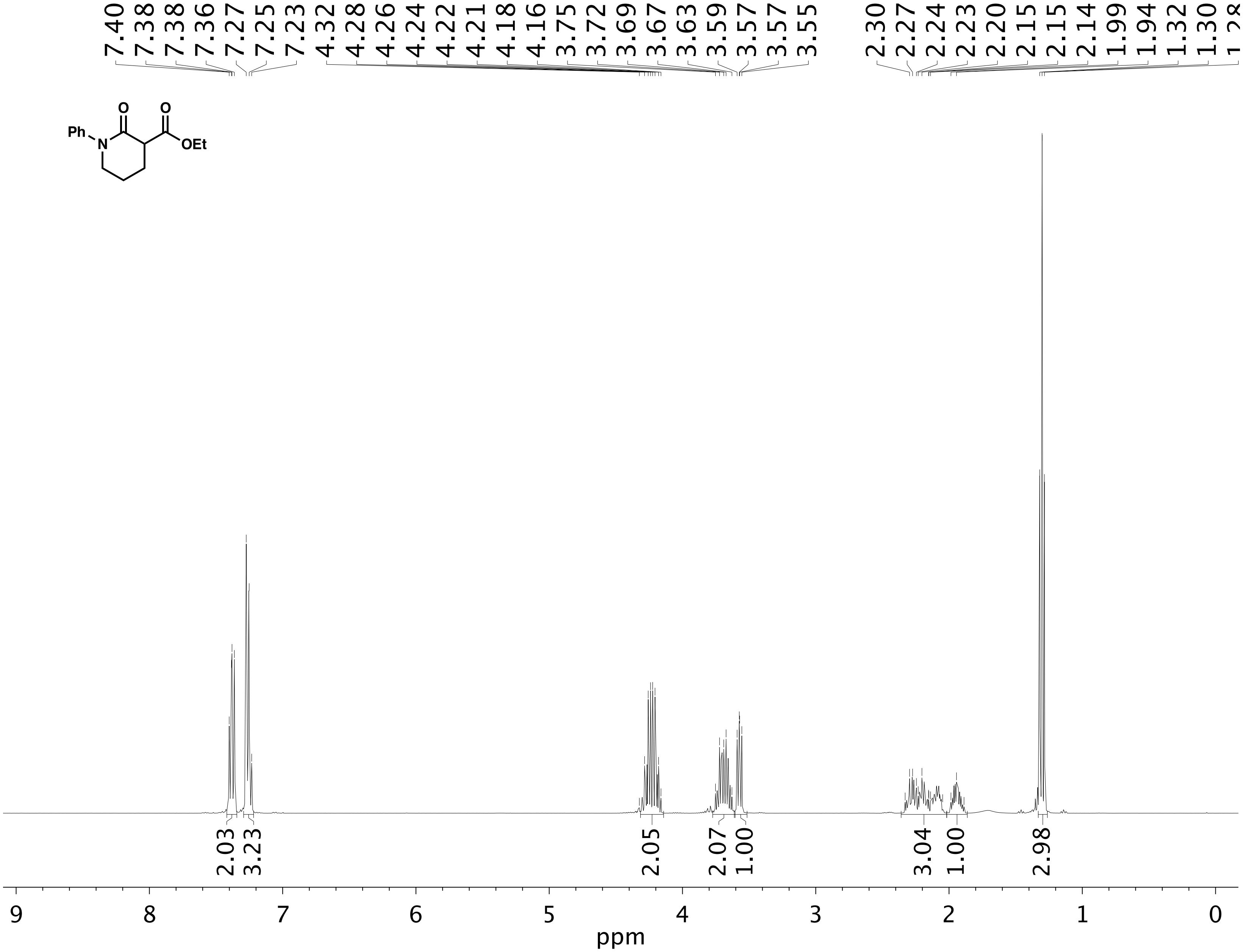

^{1}H NMR (400 MHz, $CDCl_3$) of Ph-protected lactam.

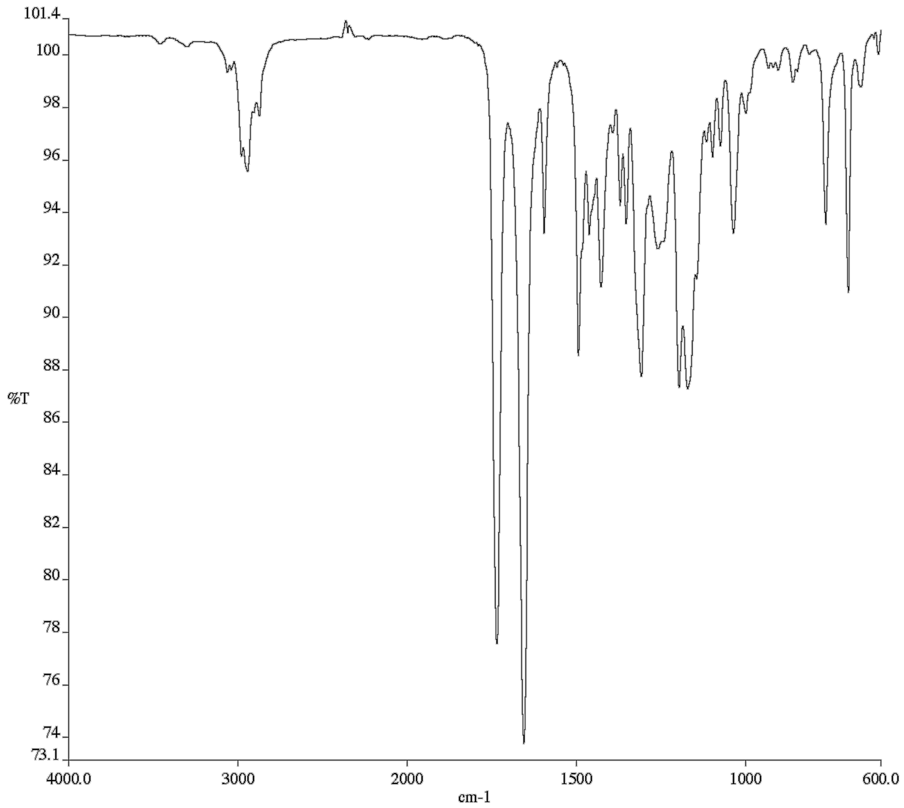

Infrared spectrum (Thin Film, NaCl) of Ph-protected lactam.

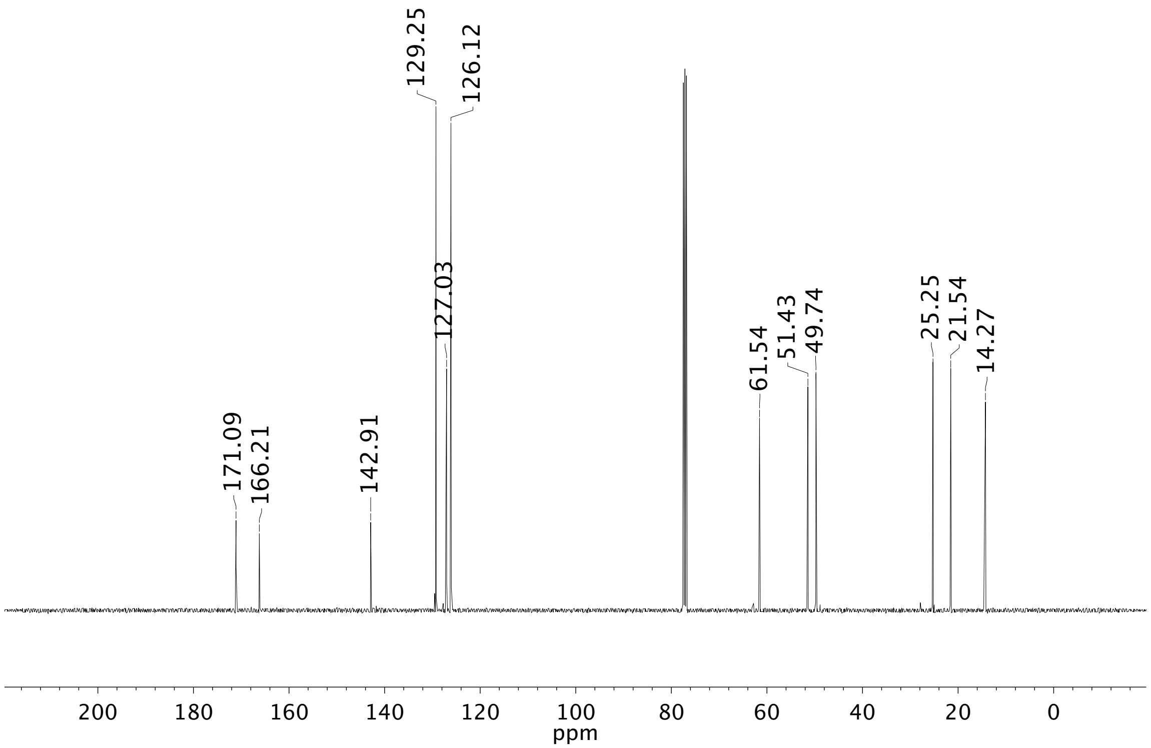

^{13}C NMR (101 MHz, $CDCl_3$) of Ph-protected lactam.

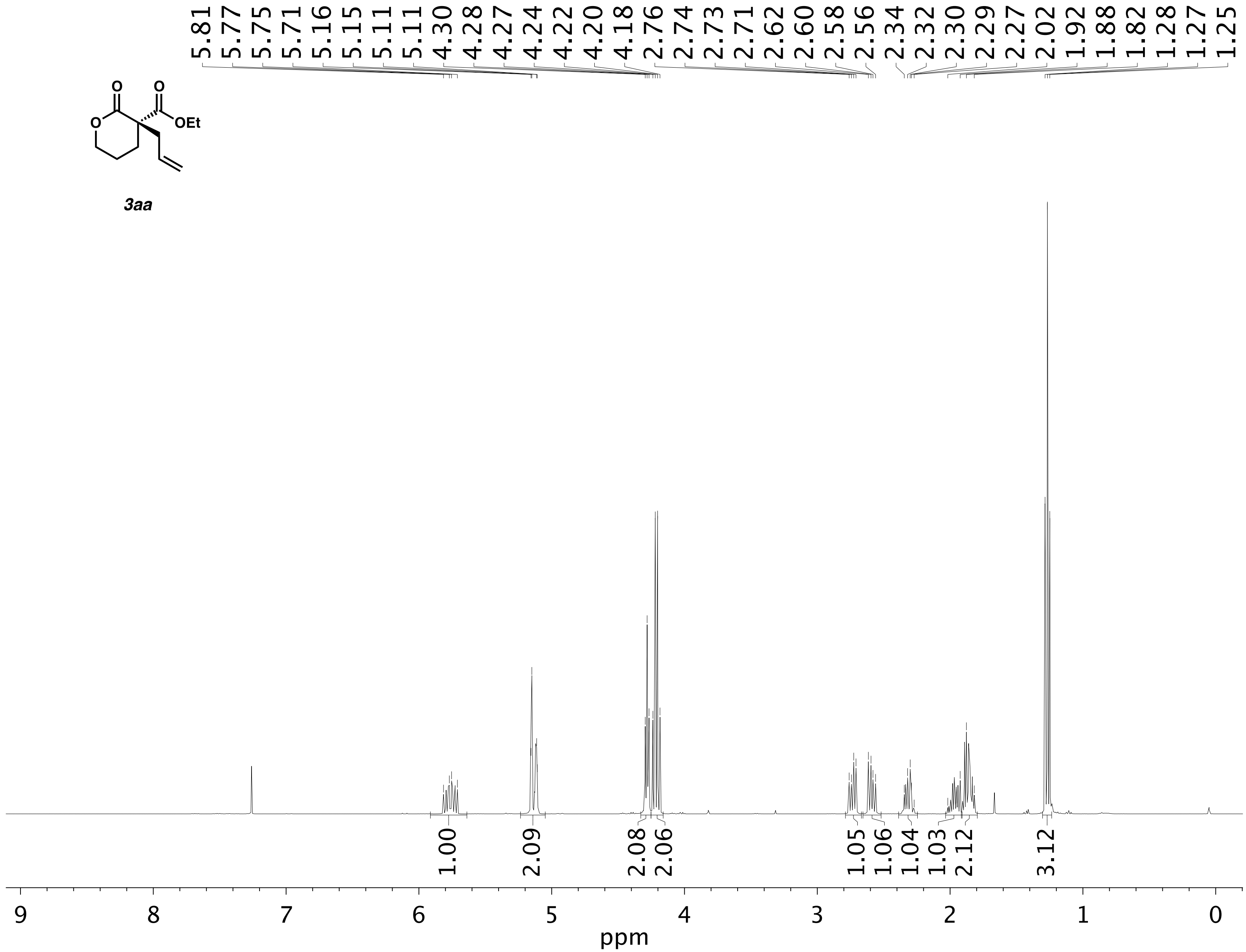

^{1}H NMR (400 MHz, $CDCl_3$) of compound **3aa**.

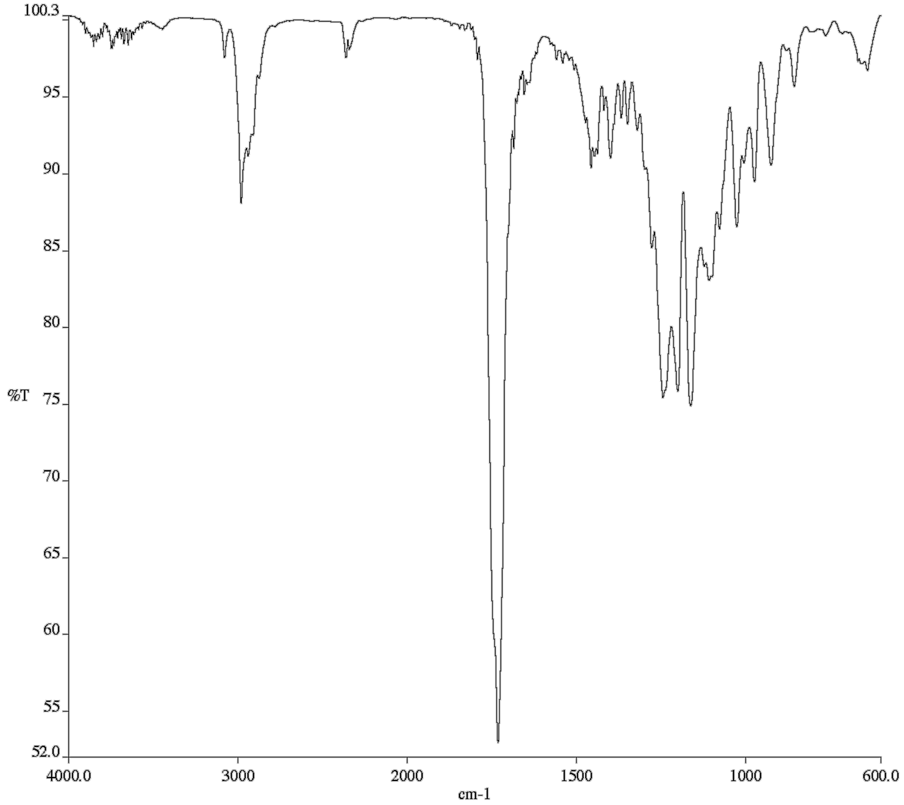

Infrared spectrum (Thin Film, NaCl) of compound **3aa**.

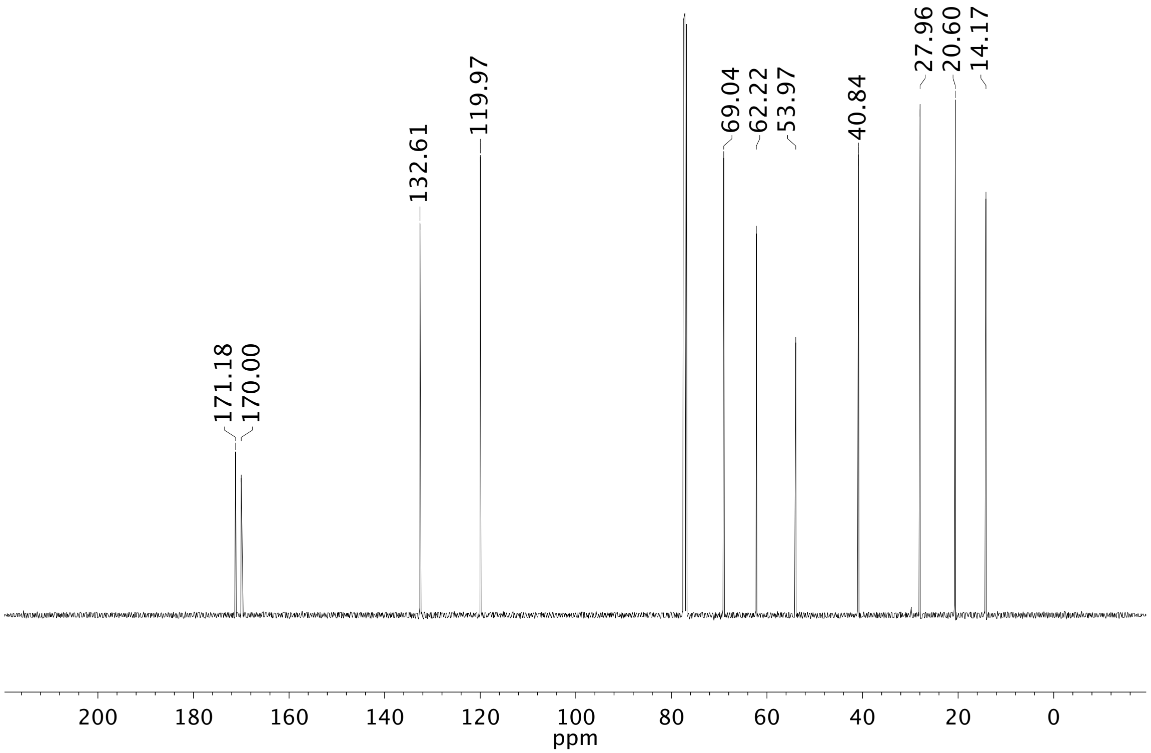

^{13}C NMR (101 MHz, $CDCl_3$) of compound **3aa**.

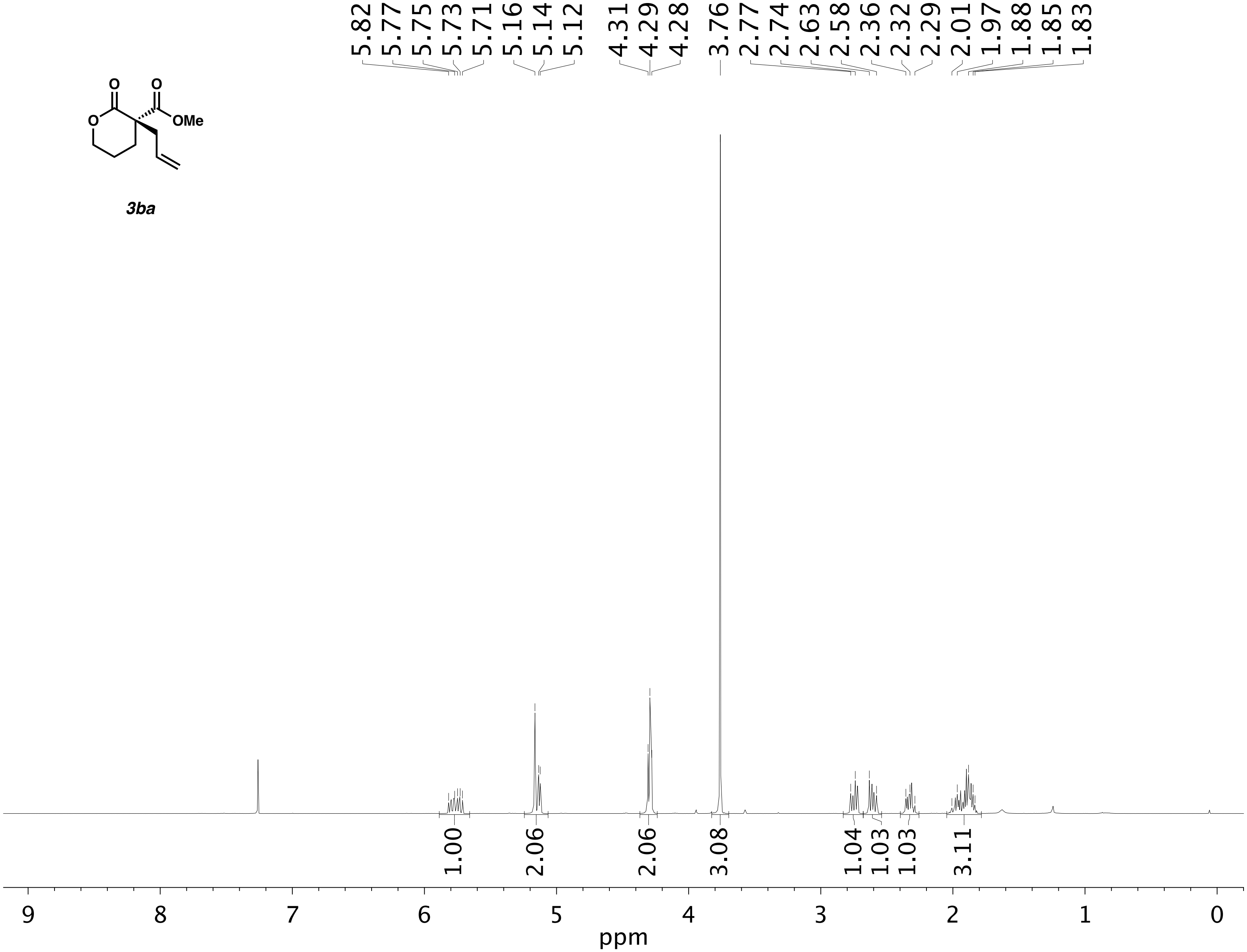

^{1}H NMR (400 MHz, $CDCl_3$) of compound **3ba**.

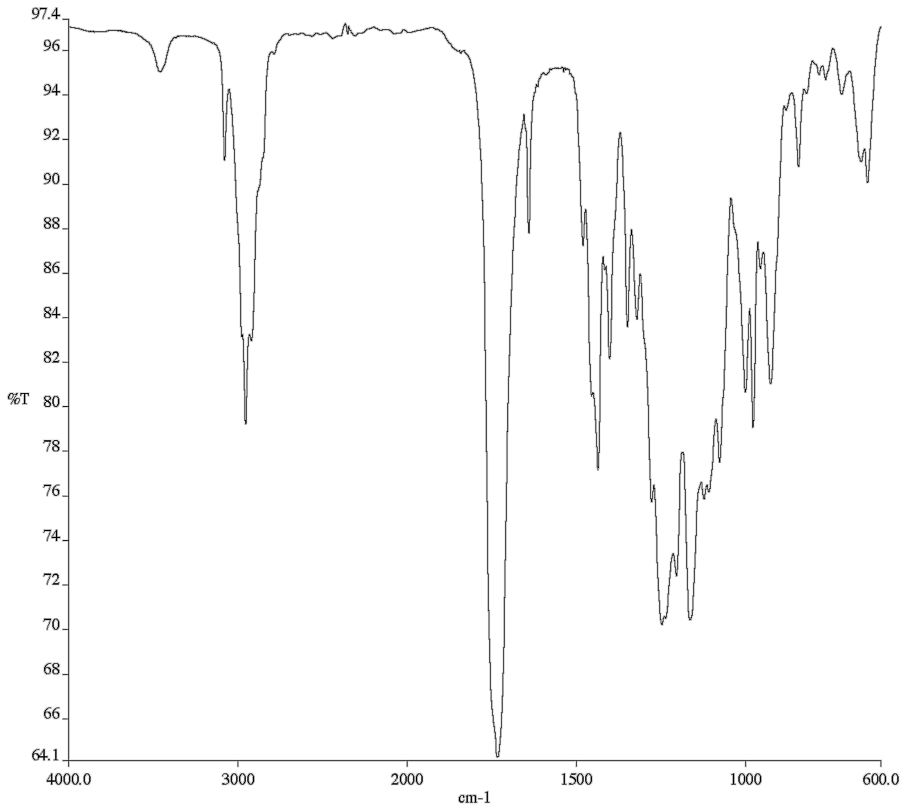

Infrared spectrum (Thin Film, NaCl) of compound **3ba**.

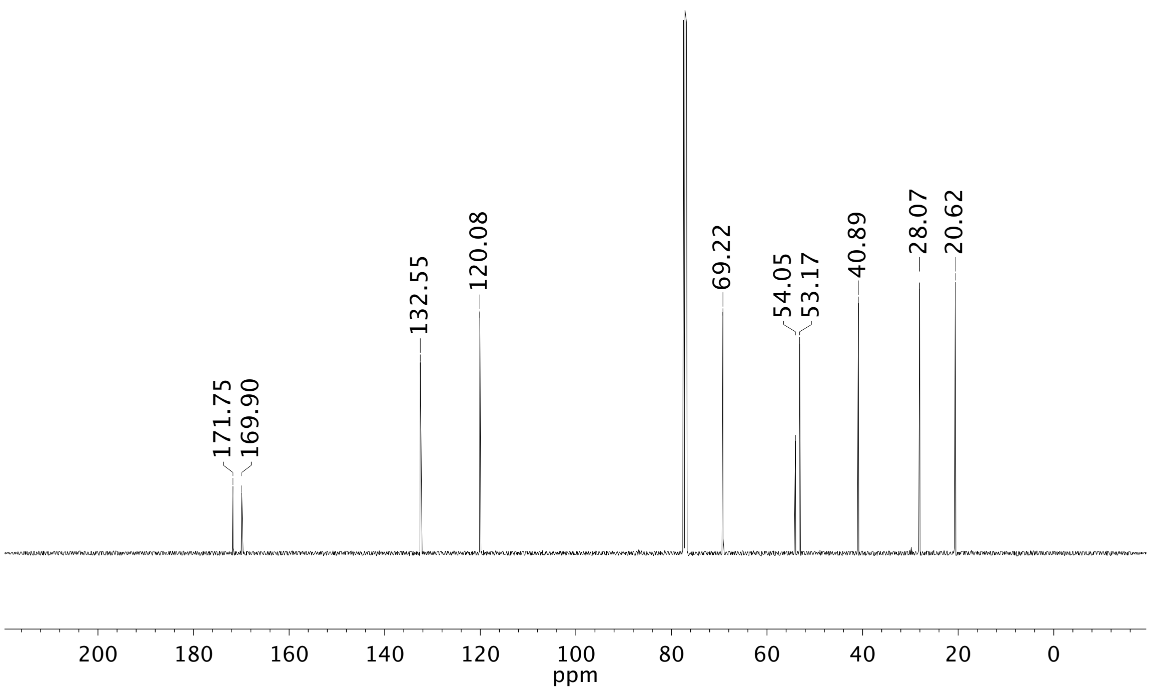

^{13}C NMR (101 MHz, $CDCl_3$) of compound **3ba**.

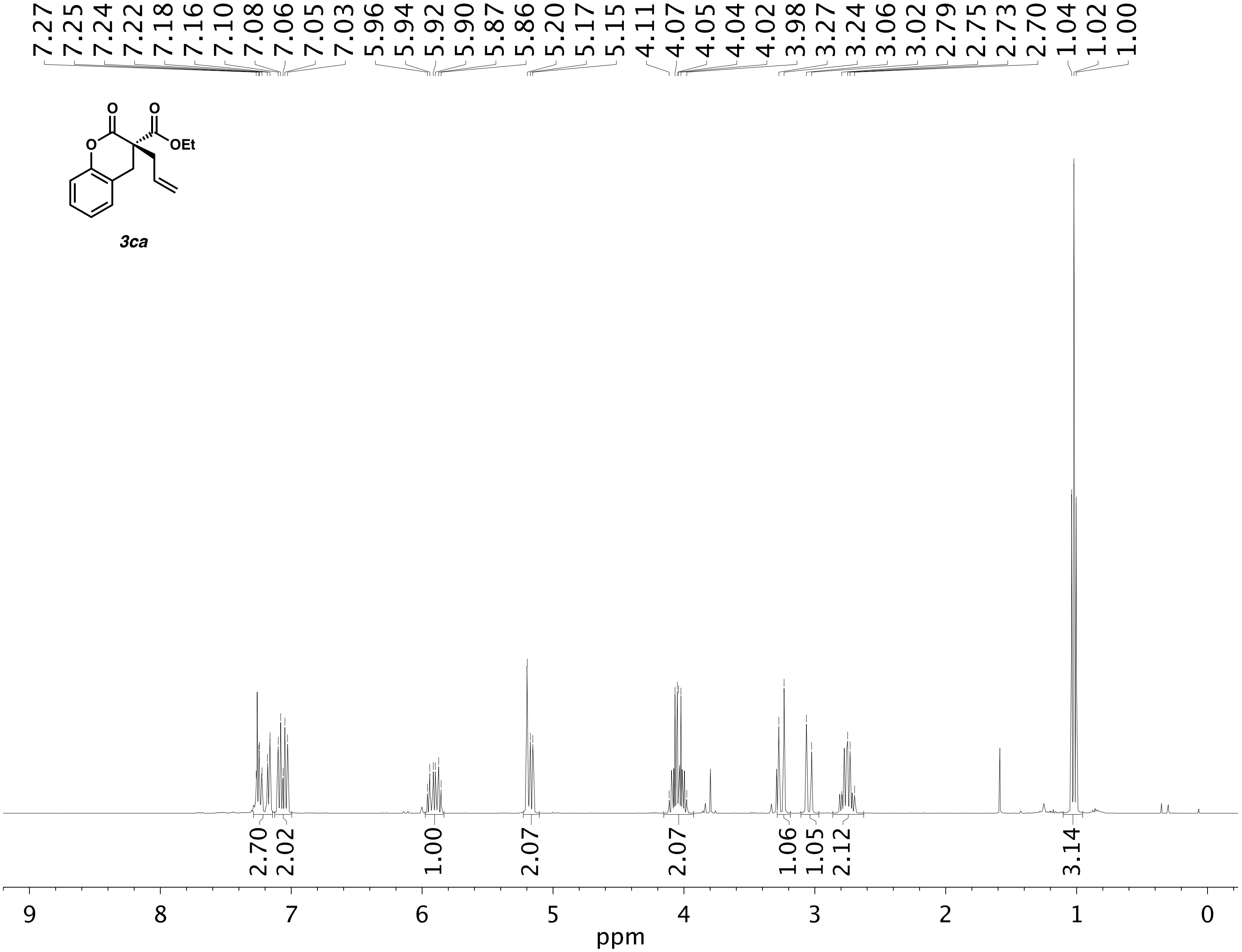

^1H NMR (400 MHz, $CDCl_3$) of compound **3ca**.

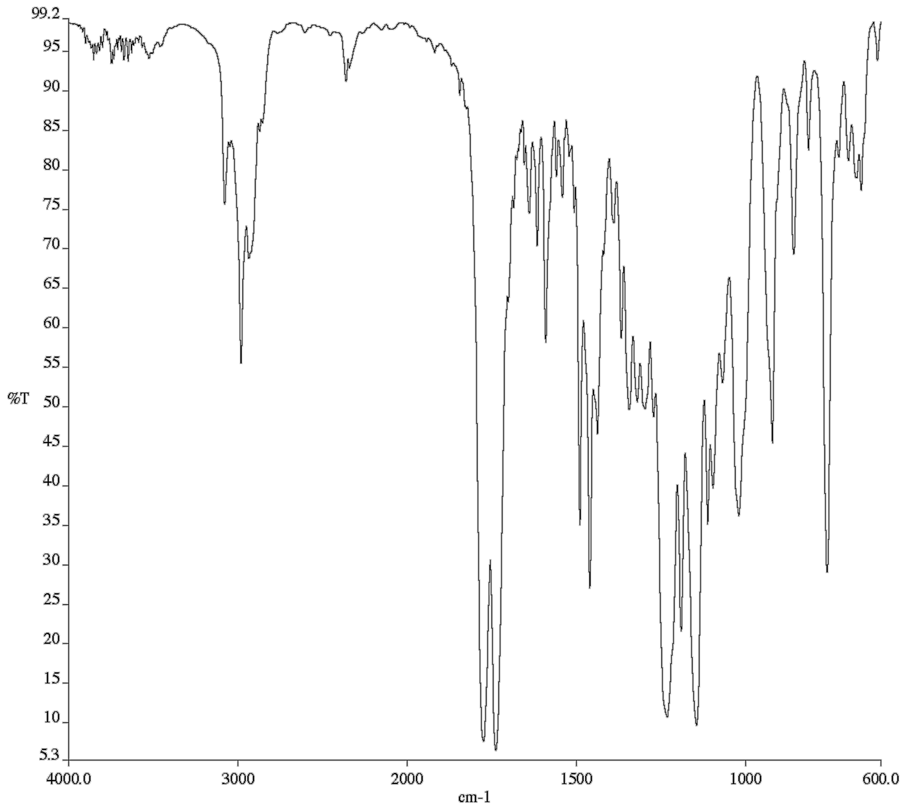

Infrared spectrum (Thin Film, NaCl) of compound **3ca**.

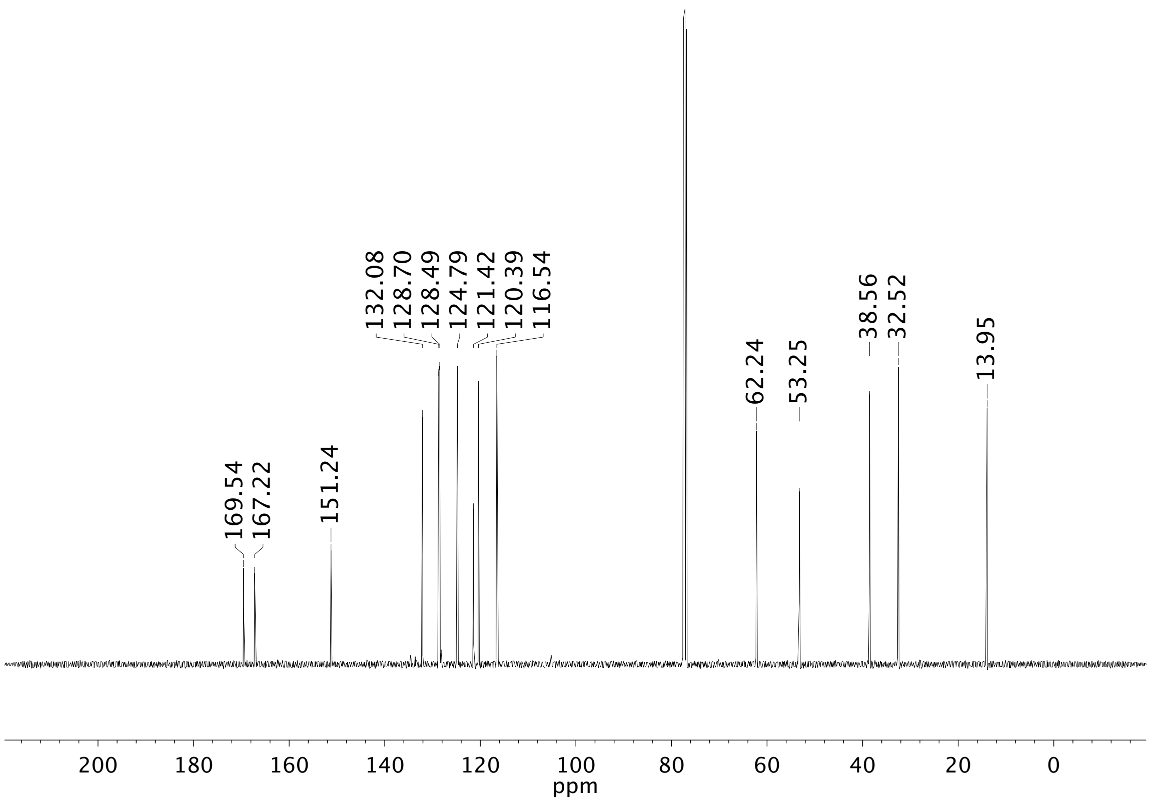

^{13}C NMR (101 MHz, $CDCl_3$) of compound **3ca**.

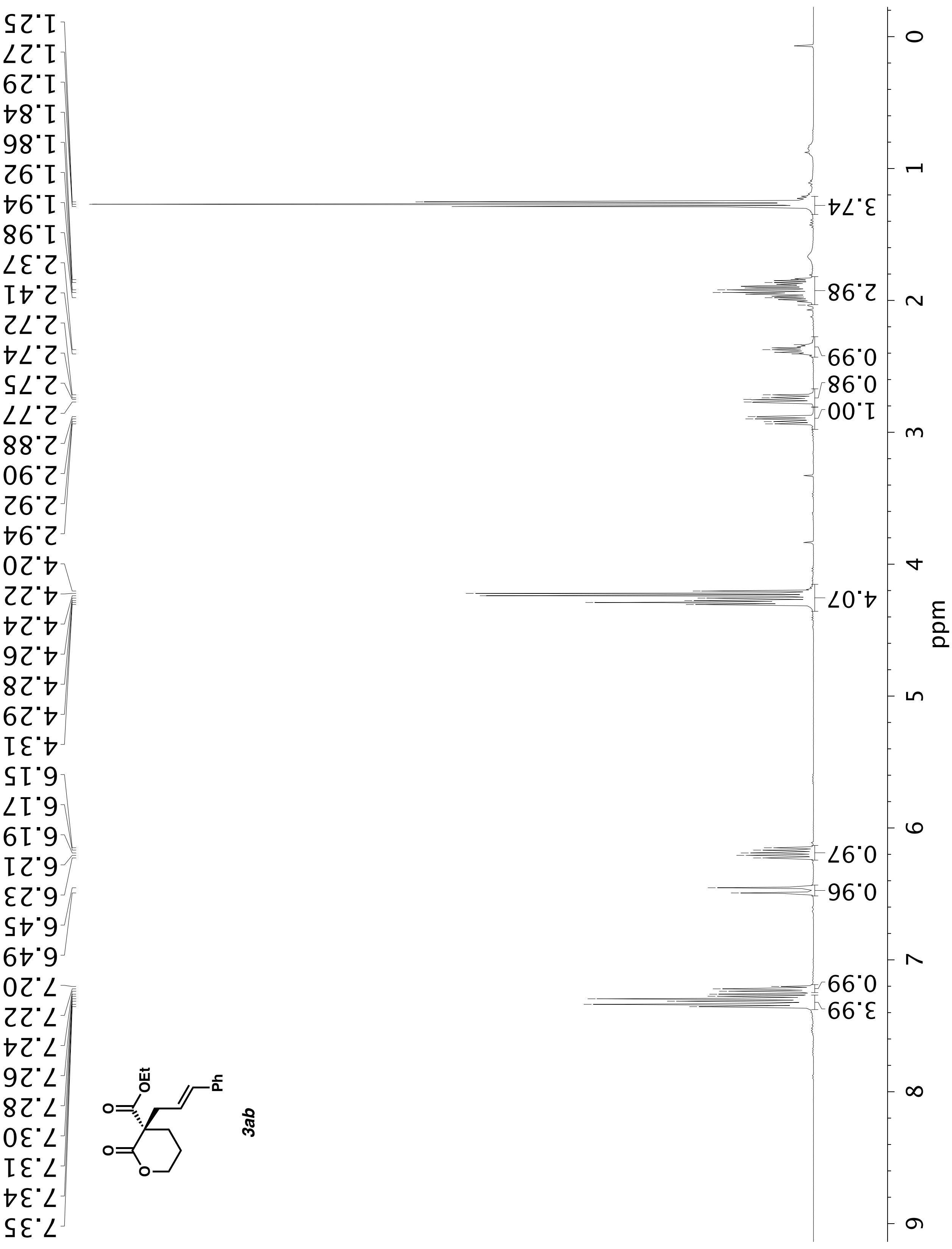

^{1}H NMR (400 MHz, $CDCl_3$) of compound **3ab**.

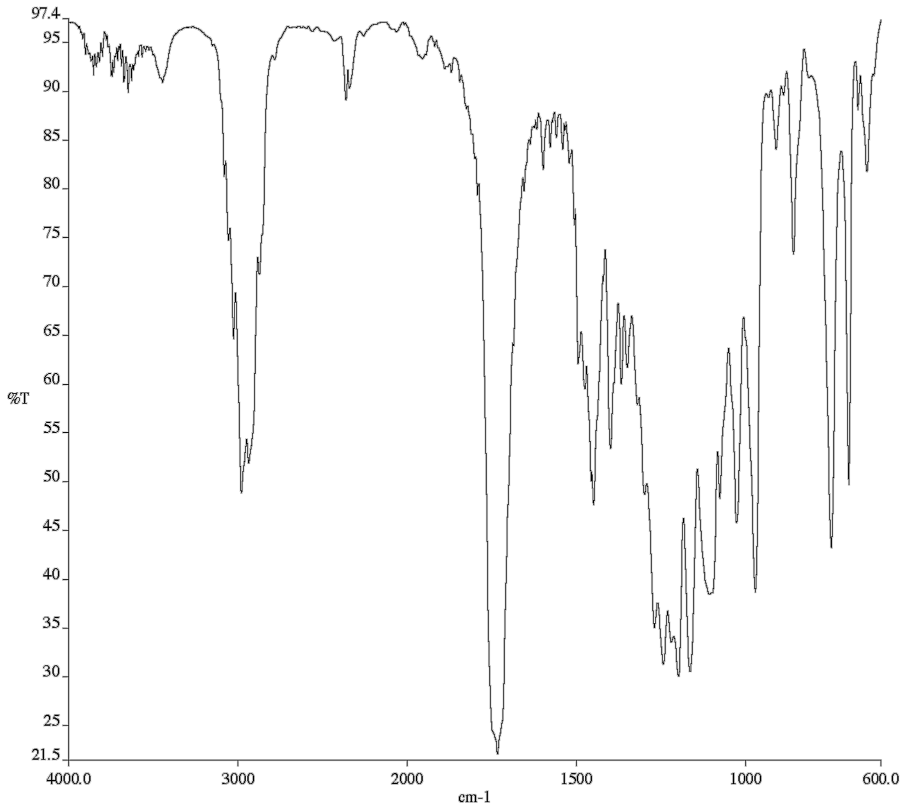

Infrared spectrum (Thin Film, NaCl) of compound **3ab**.

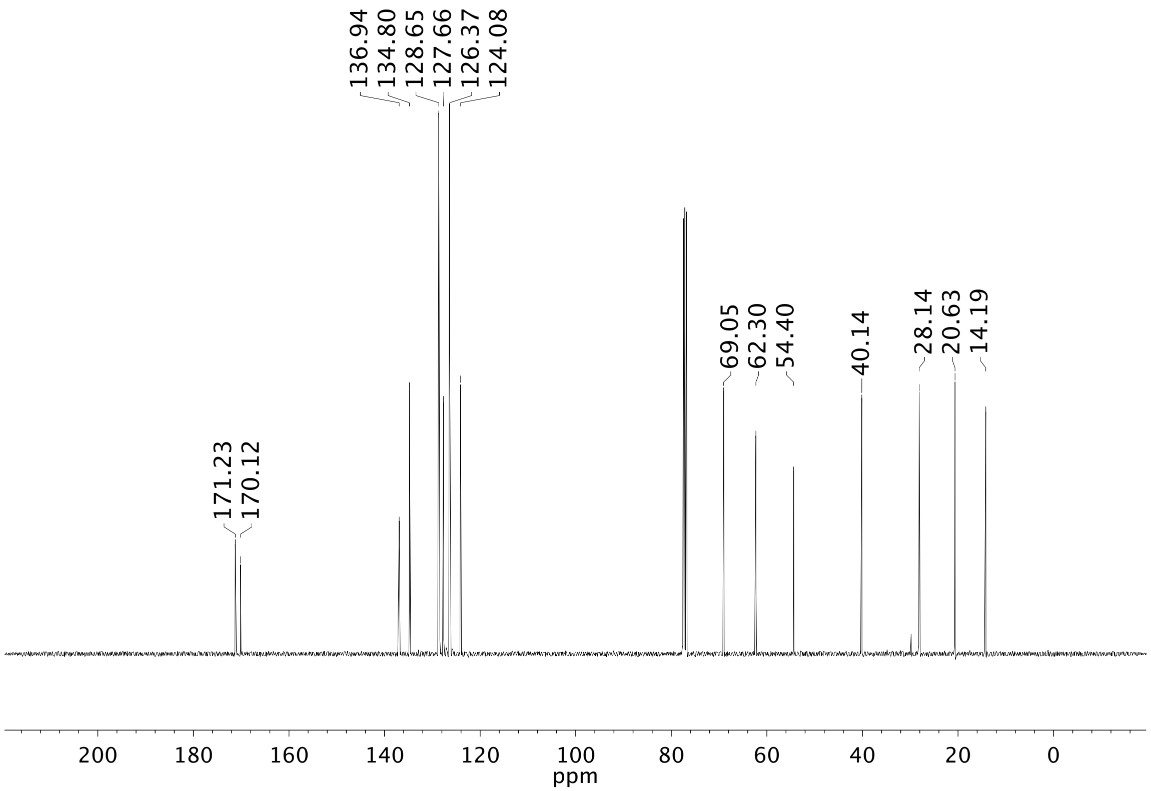

^{13}C NMR (101 MHz, $CDCl_3$) of compound **3ab**.

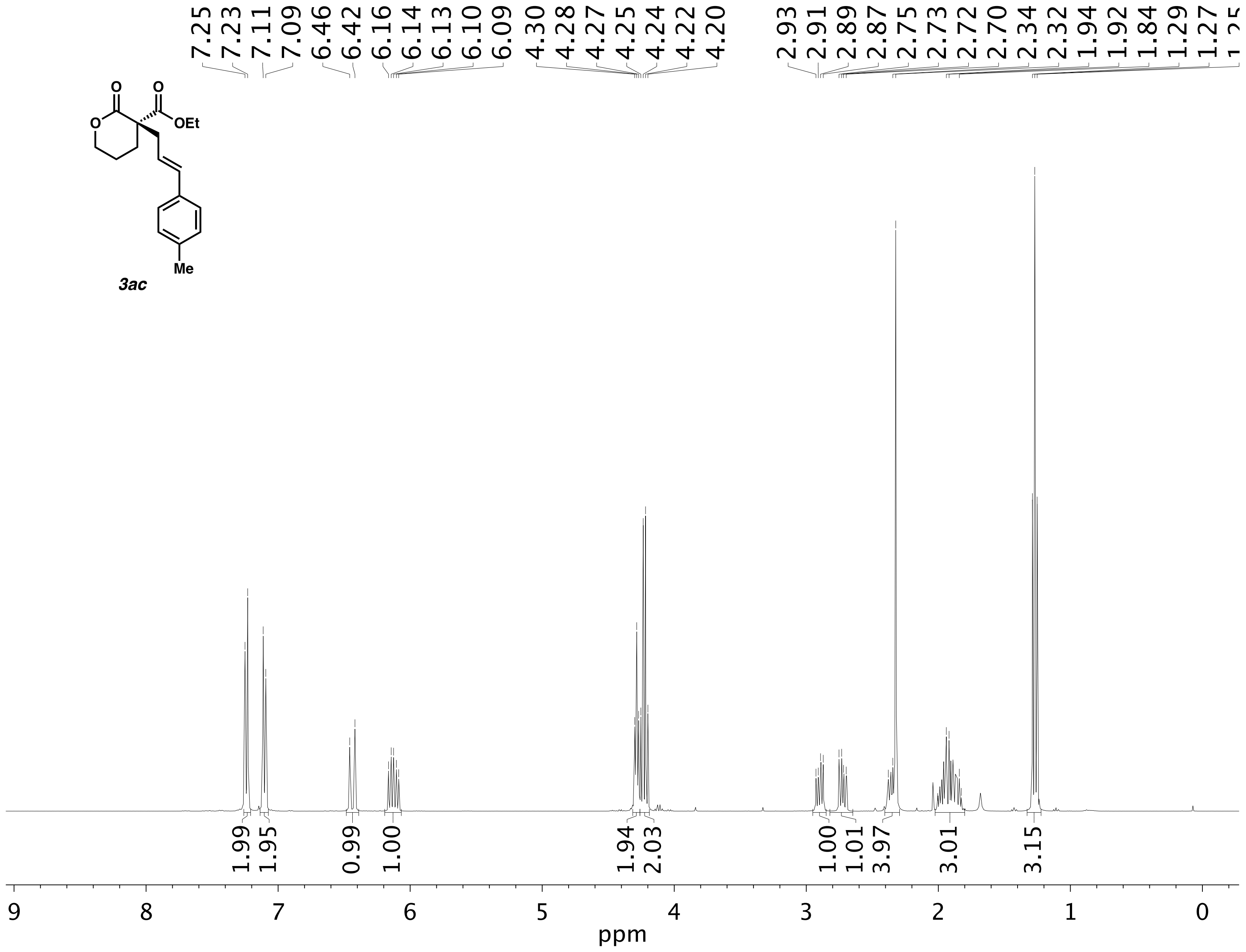

^{1}H NMR (400 MHz, $CDCl_3$) of compound **3ac**.

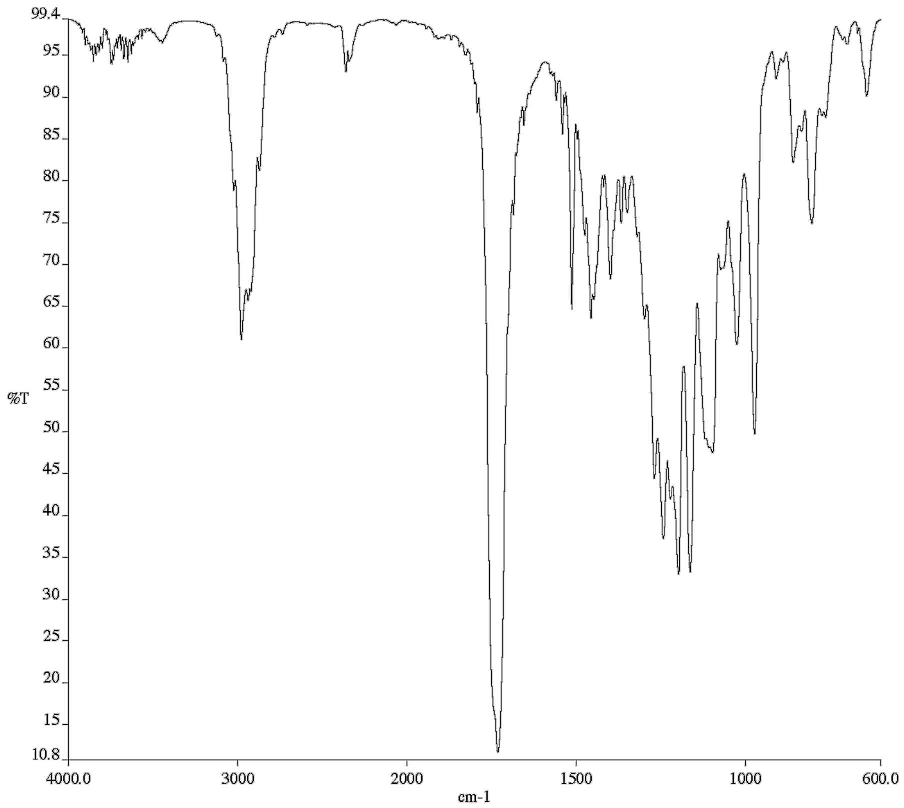

Infrared spectrum (Thin Film, NaCl) of compound **3ac**.

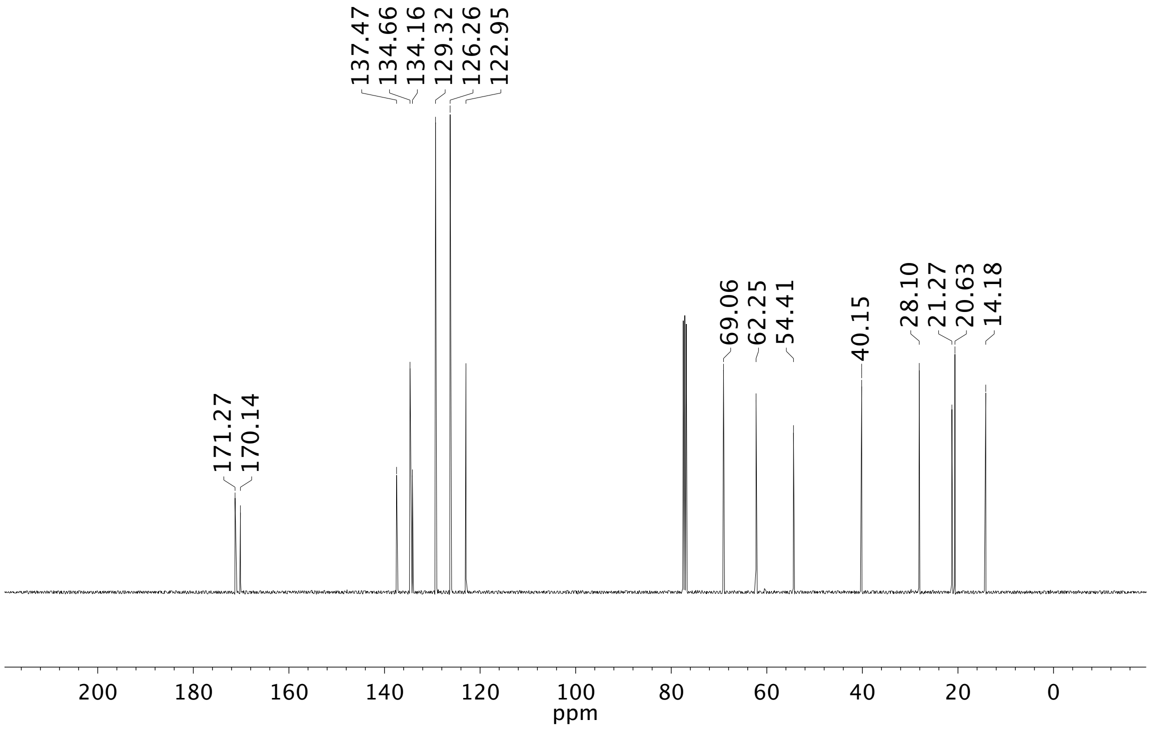

^{13}C NMR (101 MHz, $CDCl_3$) of compound **3ac**.

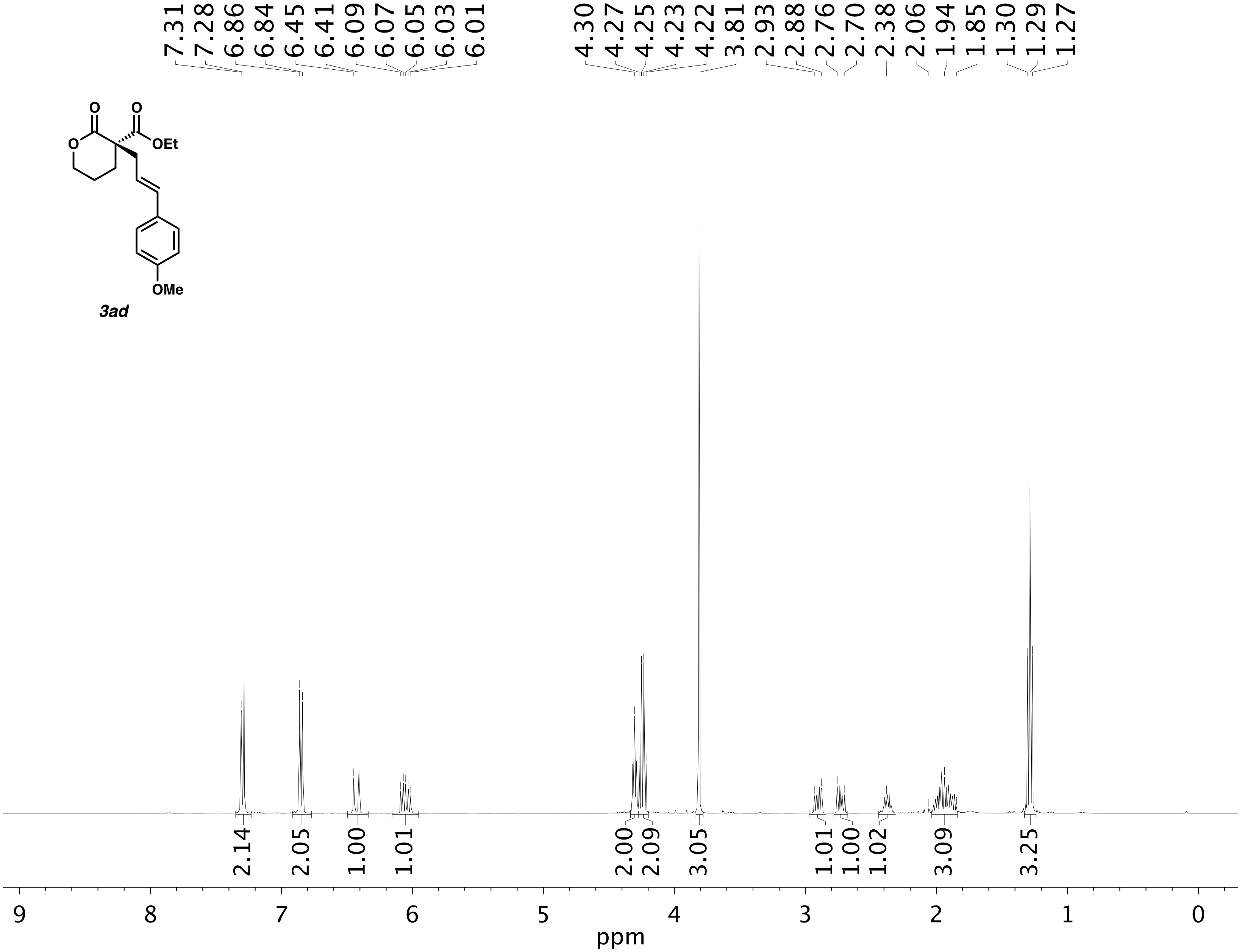

^{1}H NMR (400 MHz, $CDCl_3$) of compound **3ad**.

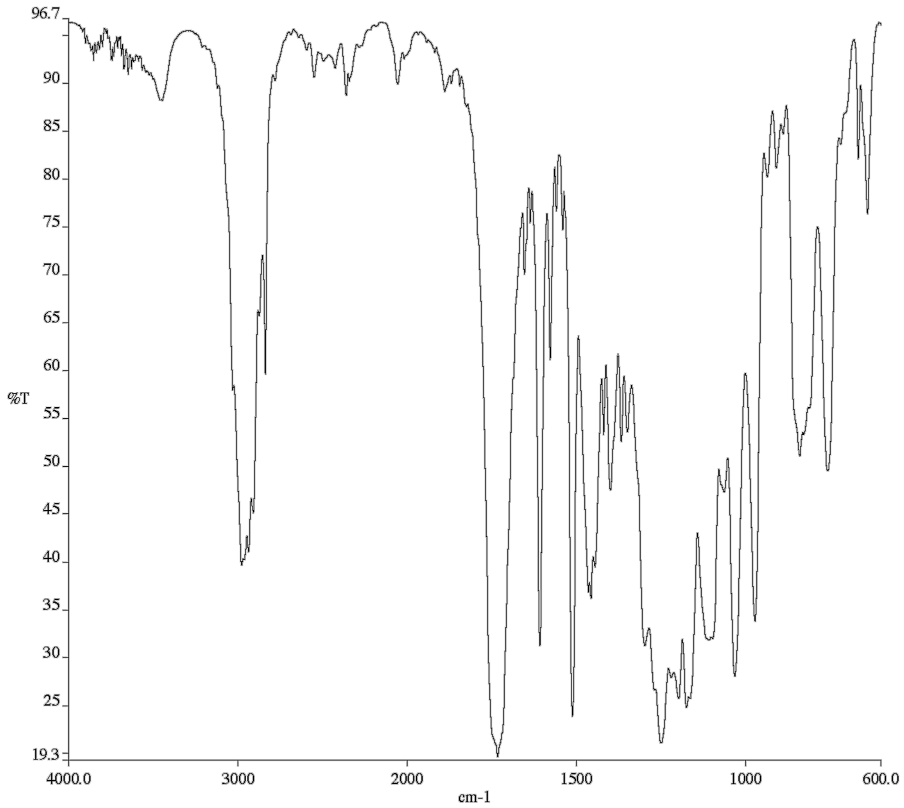

Infrared spectrum (Thin Film, NaCl) of compound **3ad**.

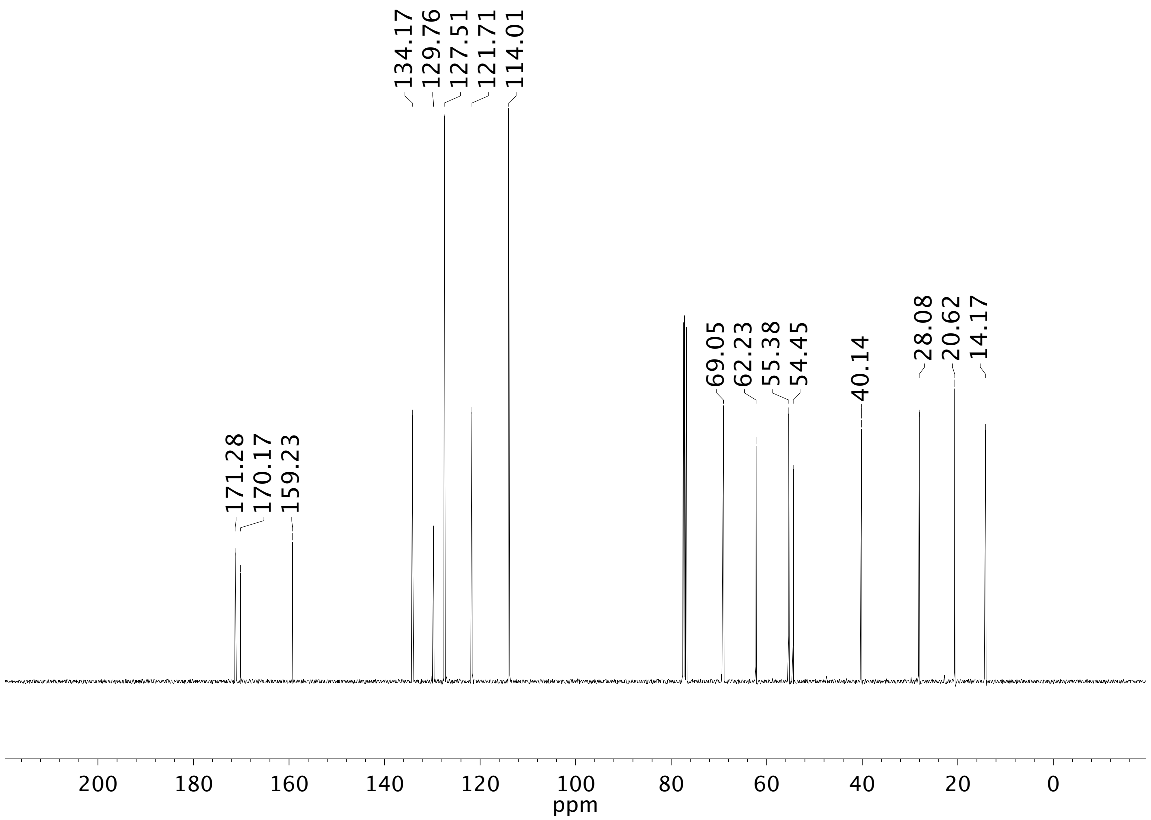

^{13}C NMR (101 MHz, $CDCl_3$) of compound **3ad**.

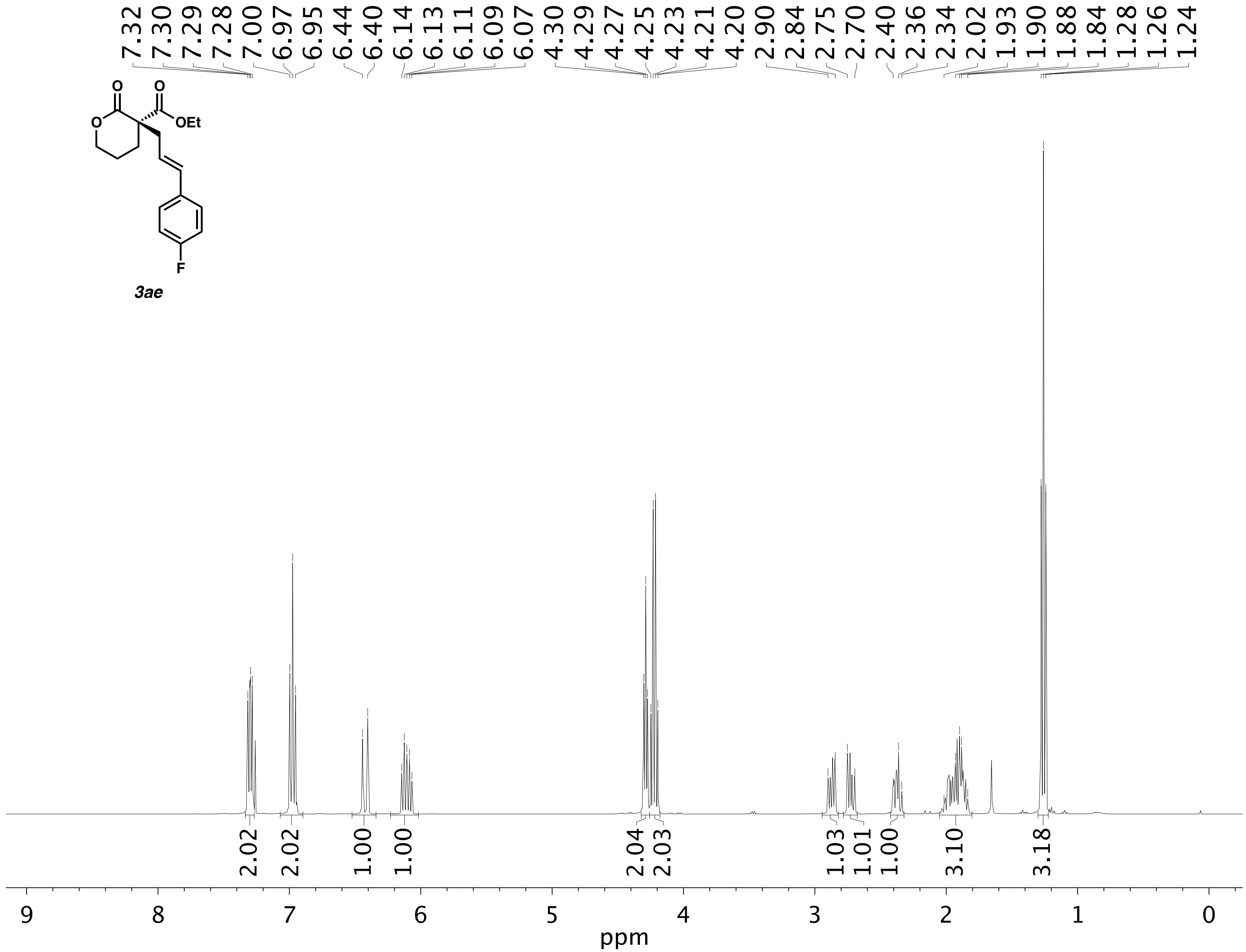

^1H NMR (400 MHz, $CDCl_3$) of compound **3ae**.

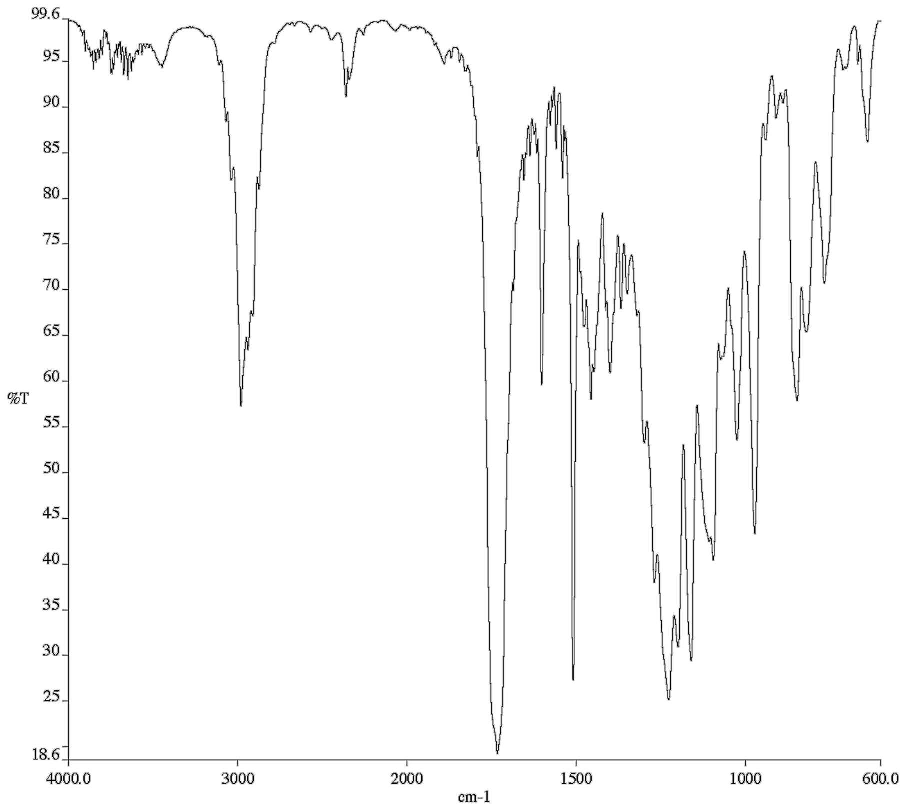

Infrared spectrum (Thin Film, NaCl) of compound **3ae**.

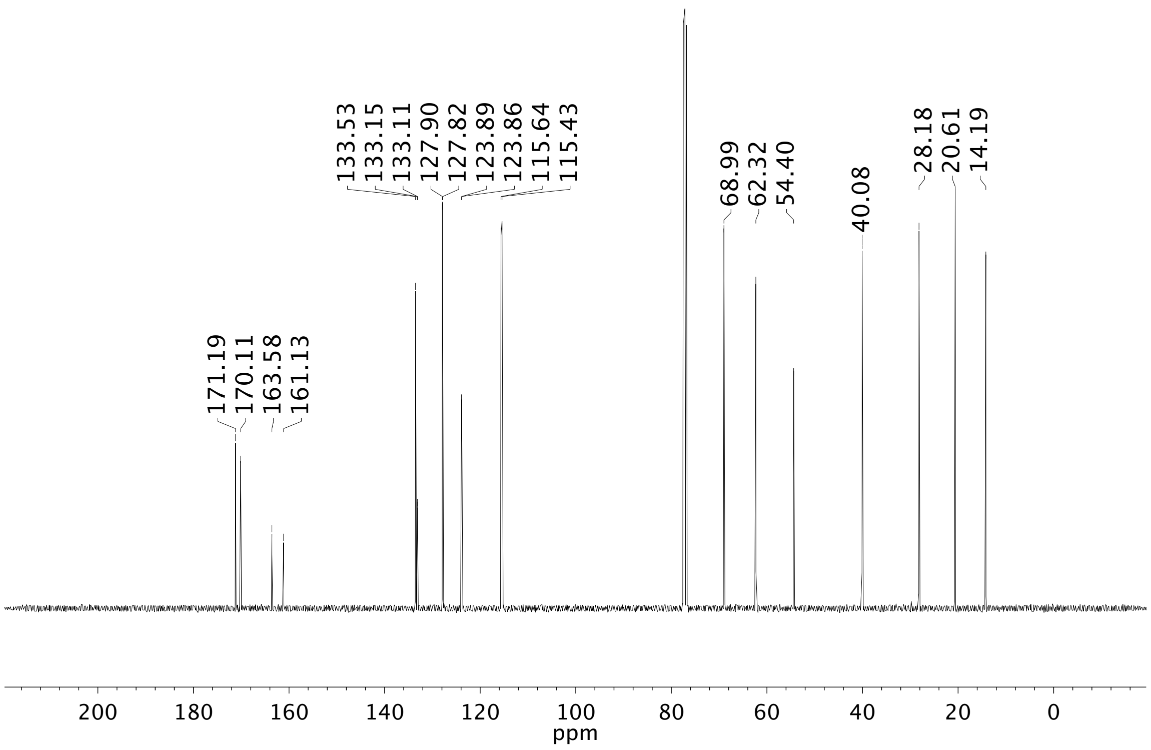

^{13}C NMR (101 MHz, $CDCl_3$) of compound **3ae**.

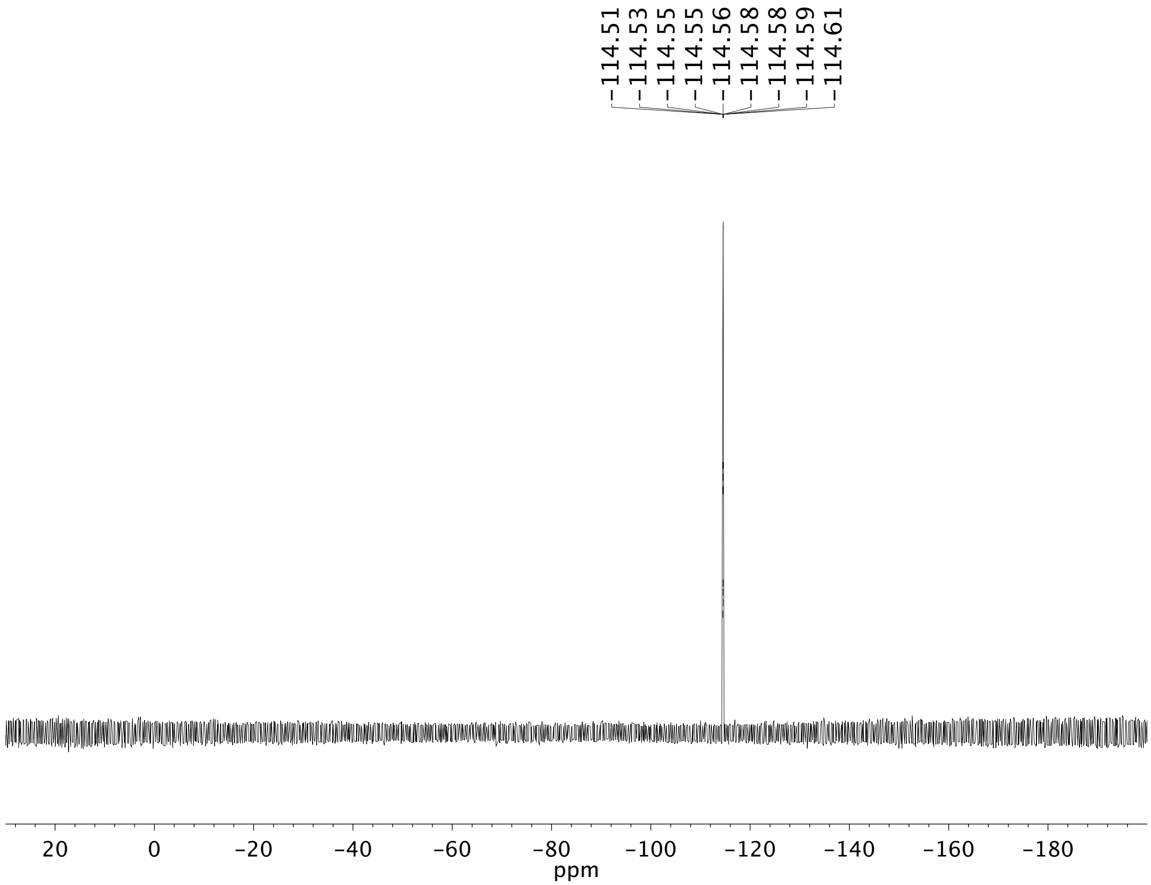

^{19}F NMR (282 MHz, $CDCl_3$) of compound **3ae**

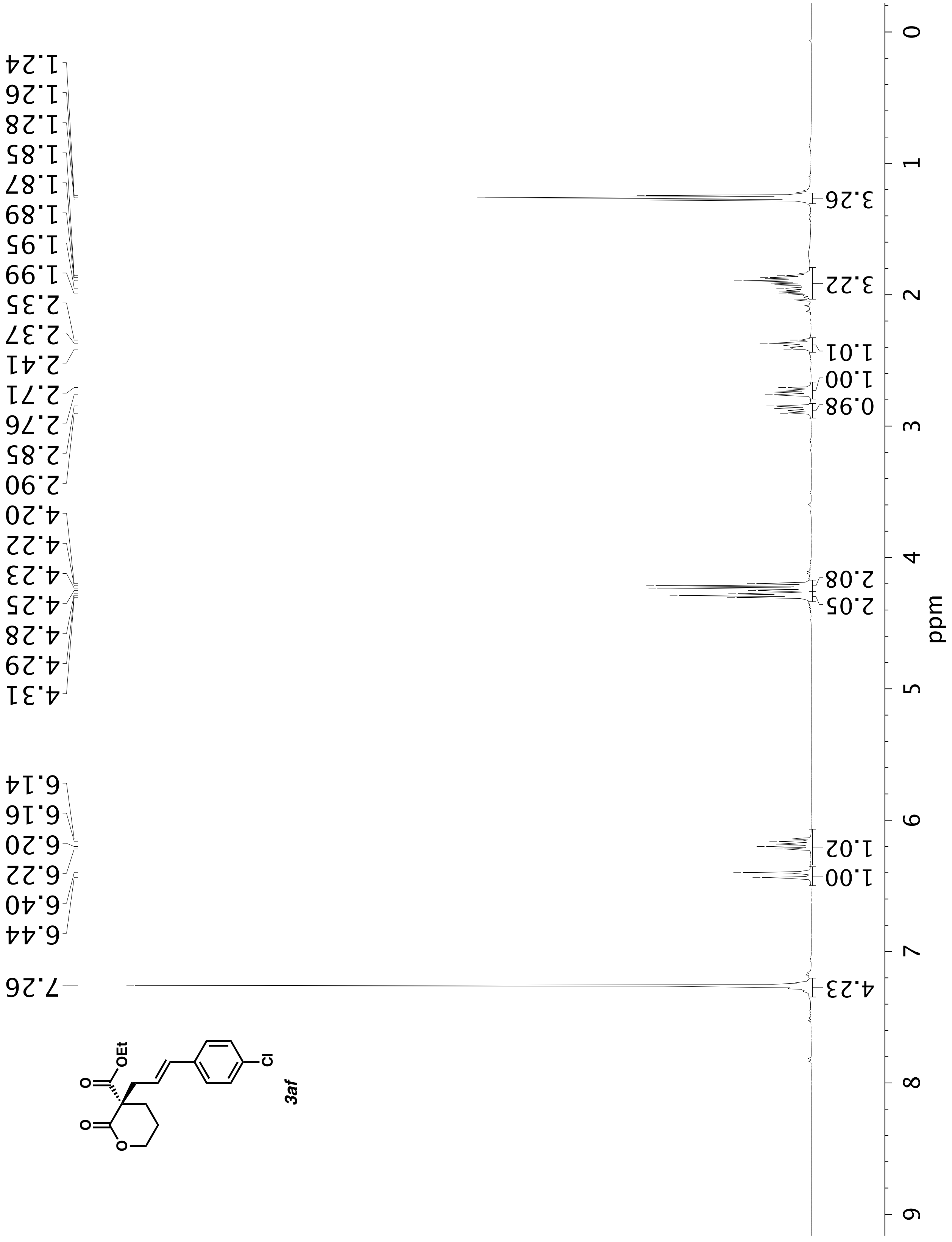

^{1}H NMR (400 MHz, $CDCl_3$) of compound **3af**.

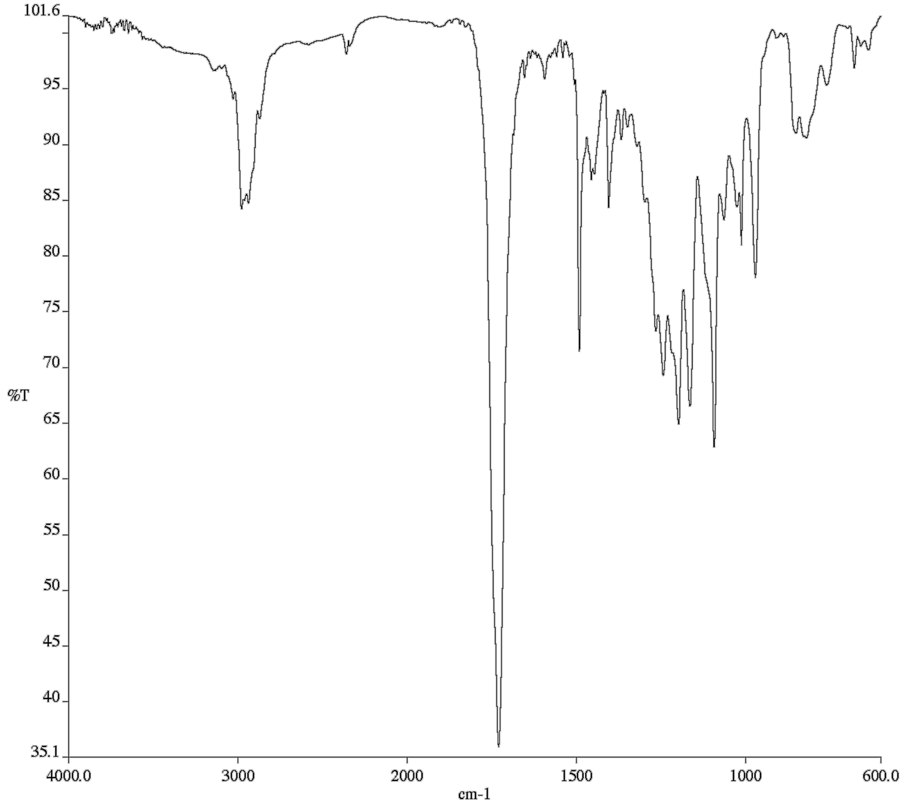

Infrared spectrum (Thin Film, NaCl) of compound **3af**.

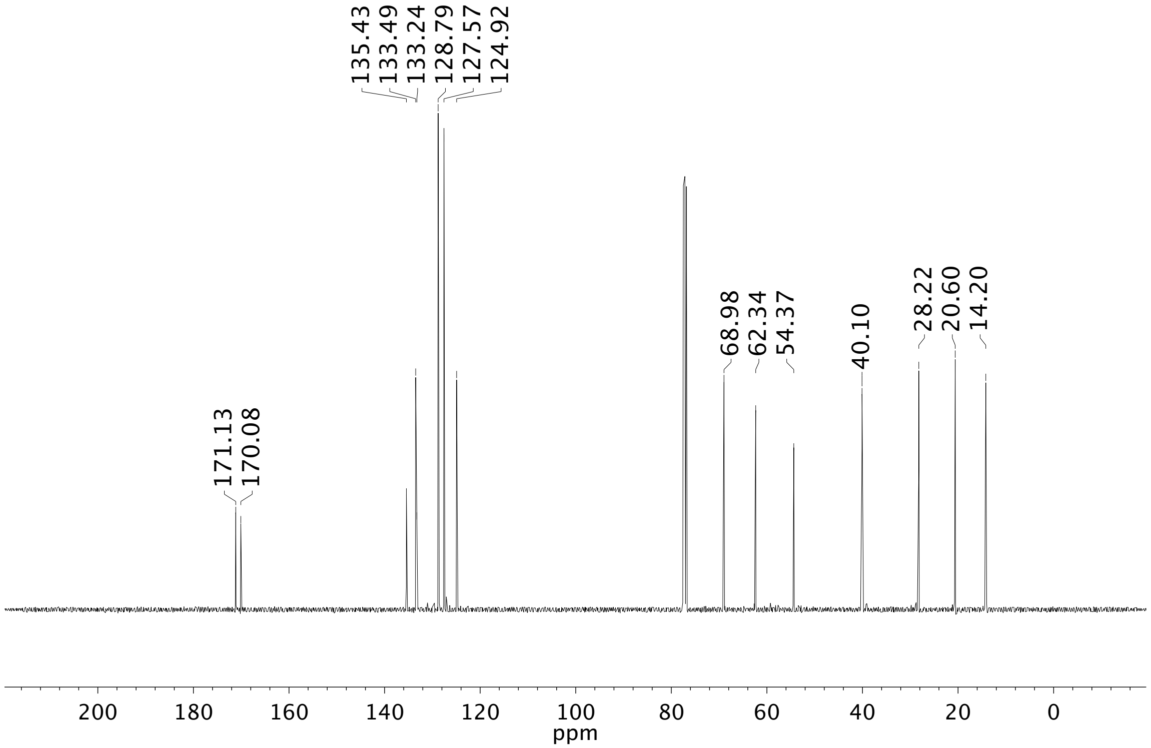

^{13}C NMR (101 MHz, $CDCl_3$) of compound **3af**.

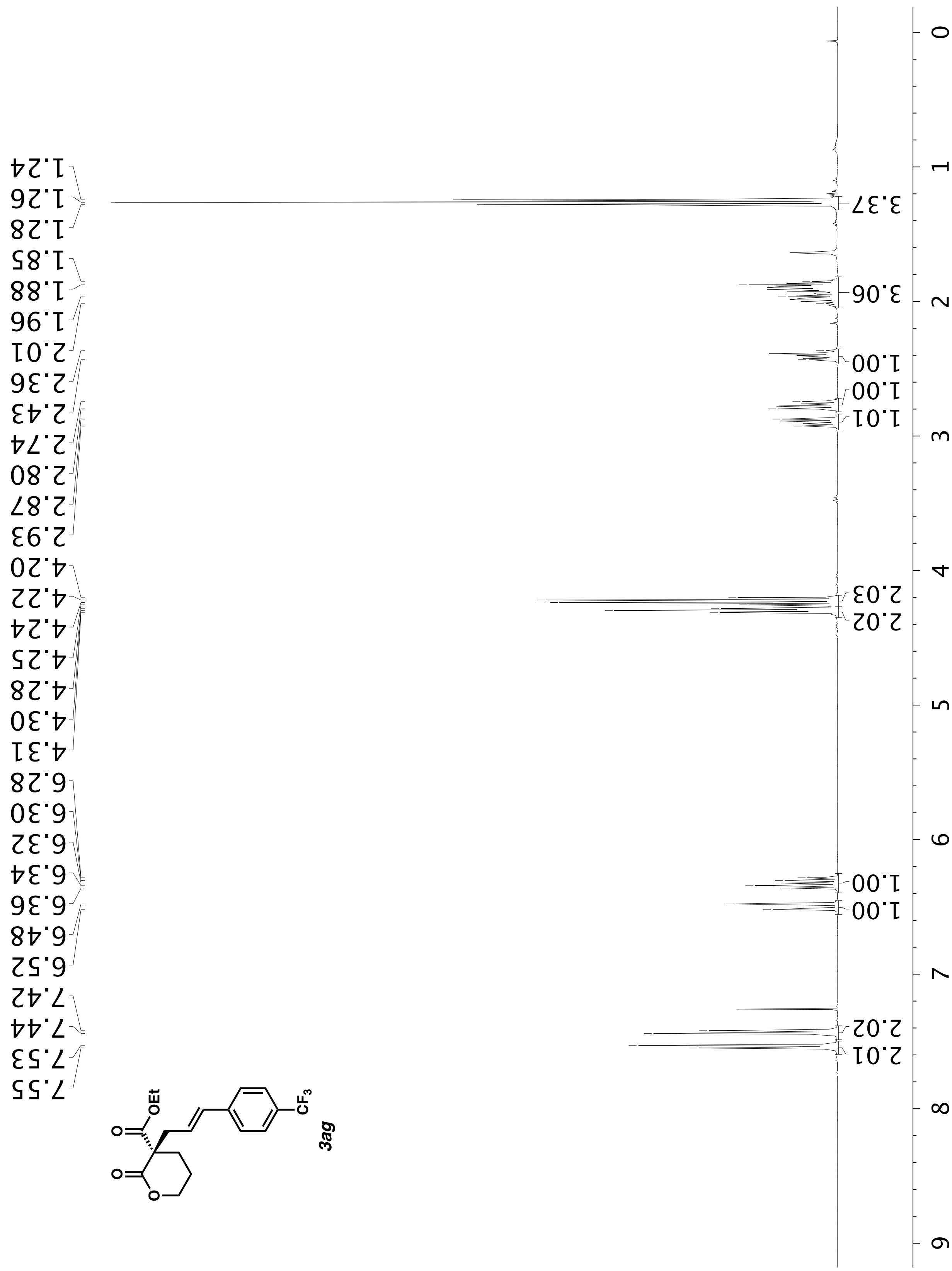

^{1}H NMR (400 MHz, $CDCl_3$) of compound **3ag**.

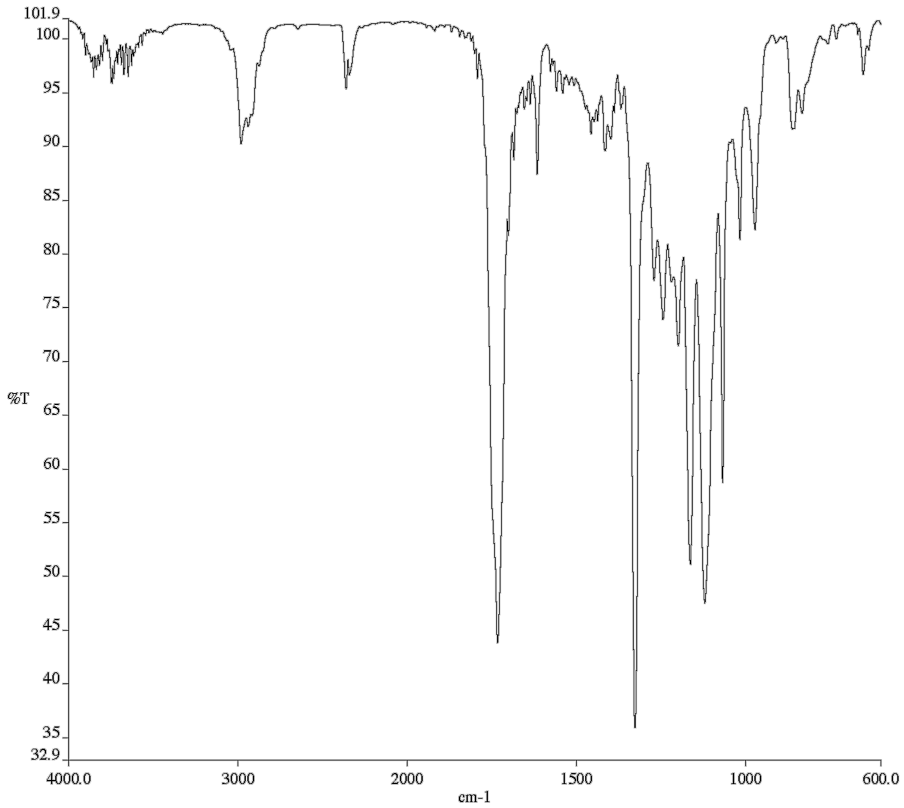

Infrared spectrum (Thin Film, NaCl) of compound **3ag**.

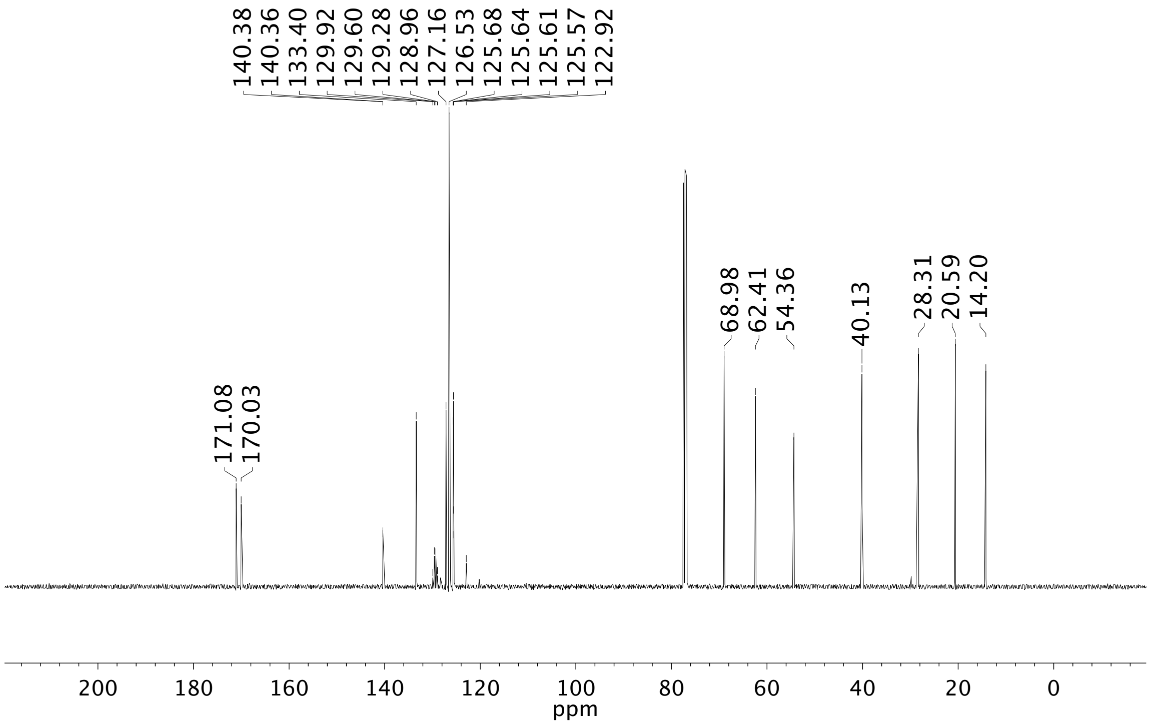

^{13}C NMR (101 MHz, $CDCl_3$) of compound **3ag**.

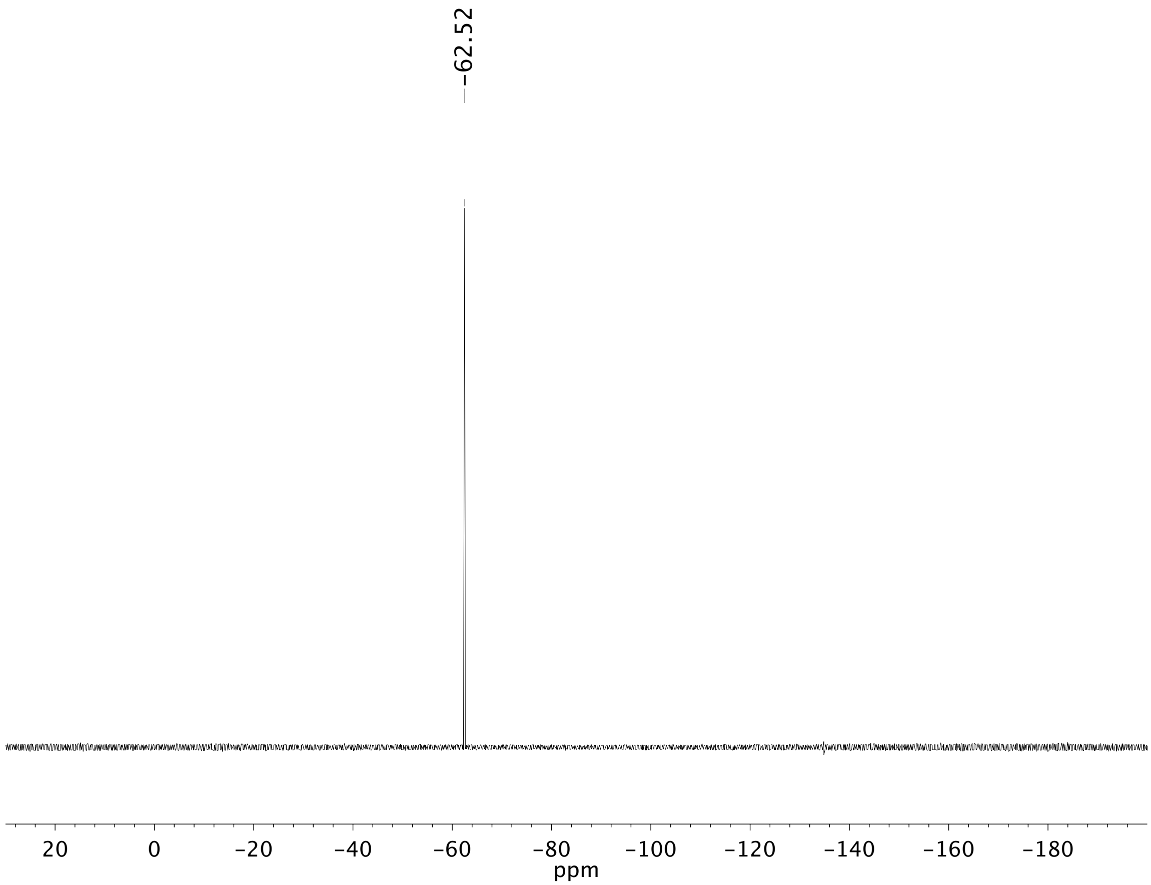

^{19}F NMR (282 MHz, $CDCl_3$) of compound **3ag**.

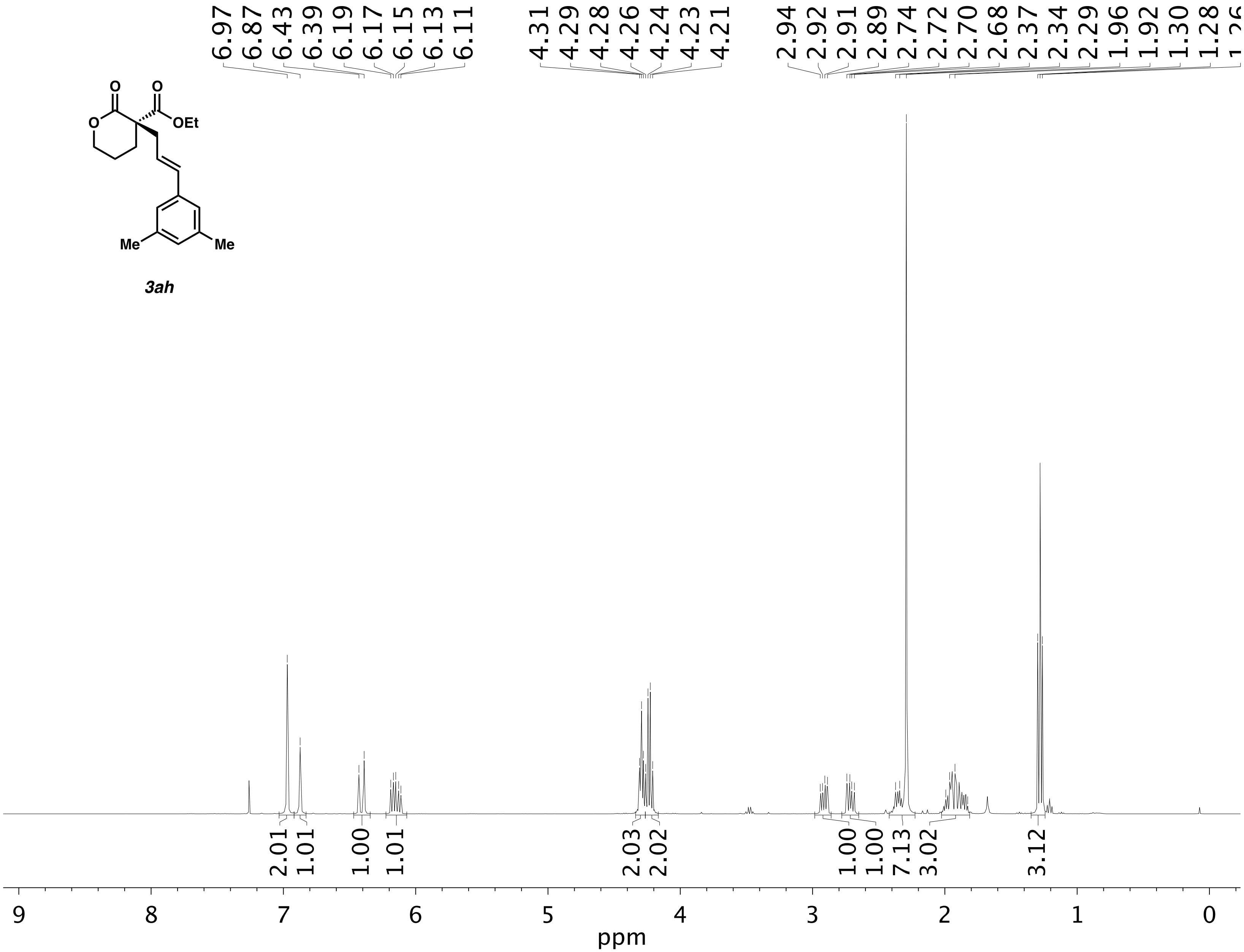

3ah

^{1}H NMR (400 MHz, $CDCl_3$) of compound **3ah**.

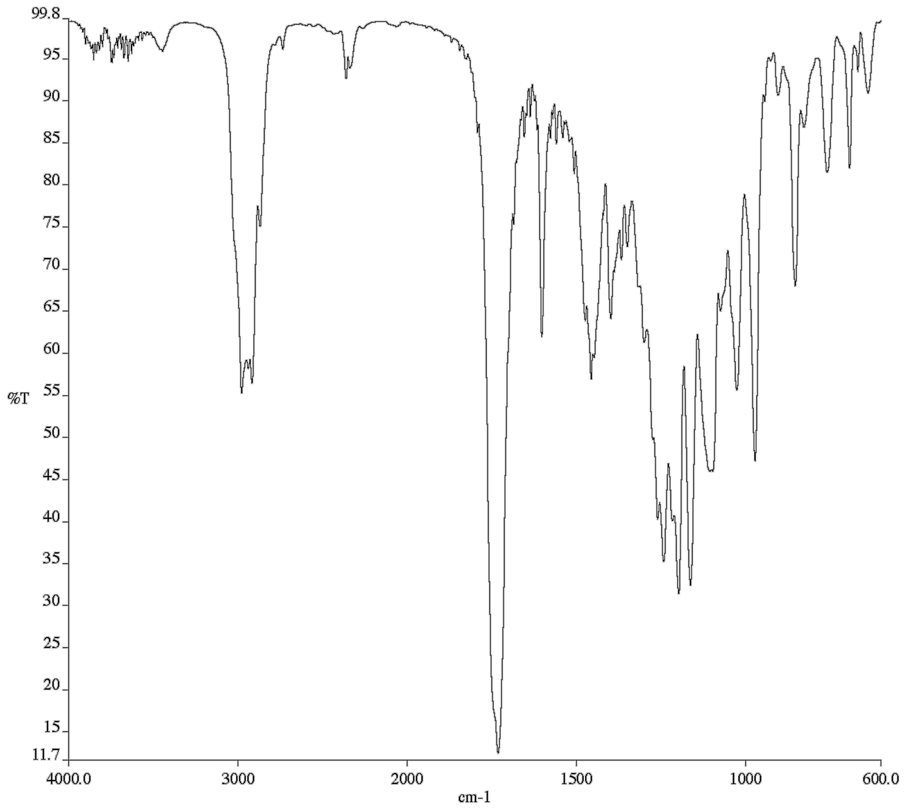

Infrared spectrum (Thin Film, NaCl) of compound **3ah**.

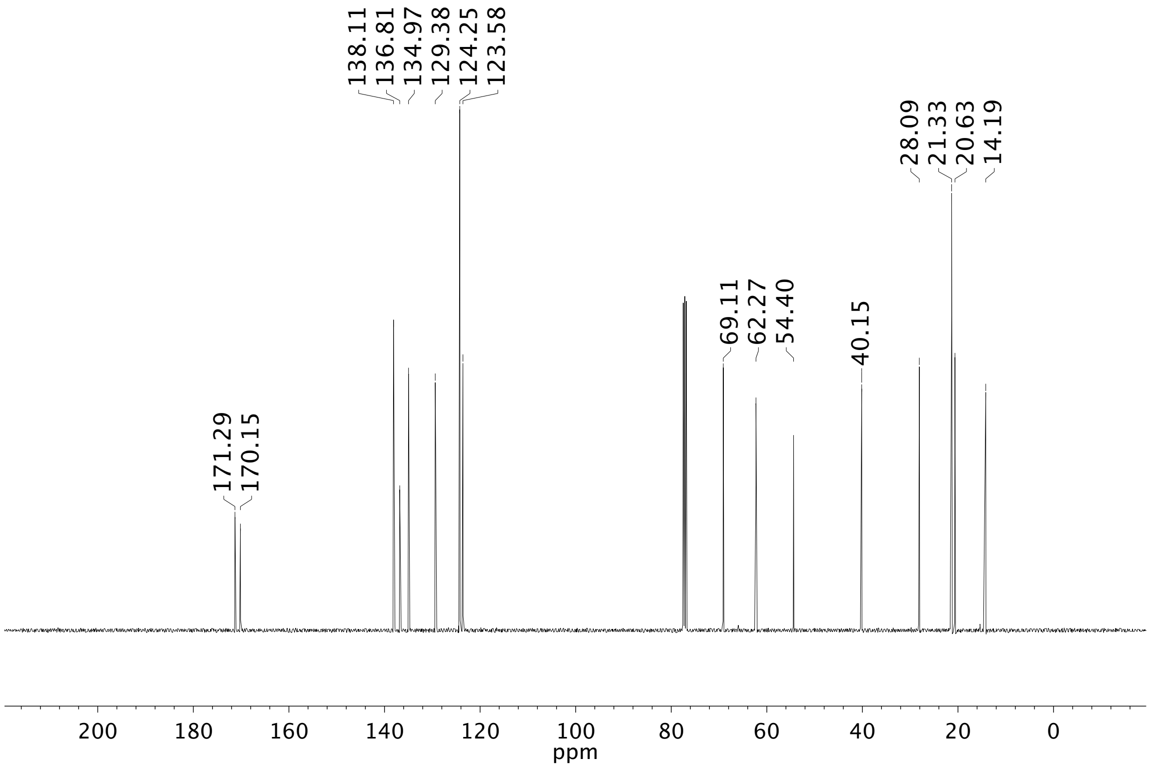

^{13}C NMR (101 MHz, $CDCl_3$) of compound **3ah**.

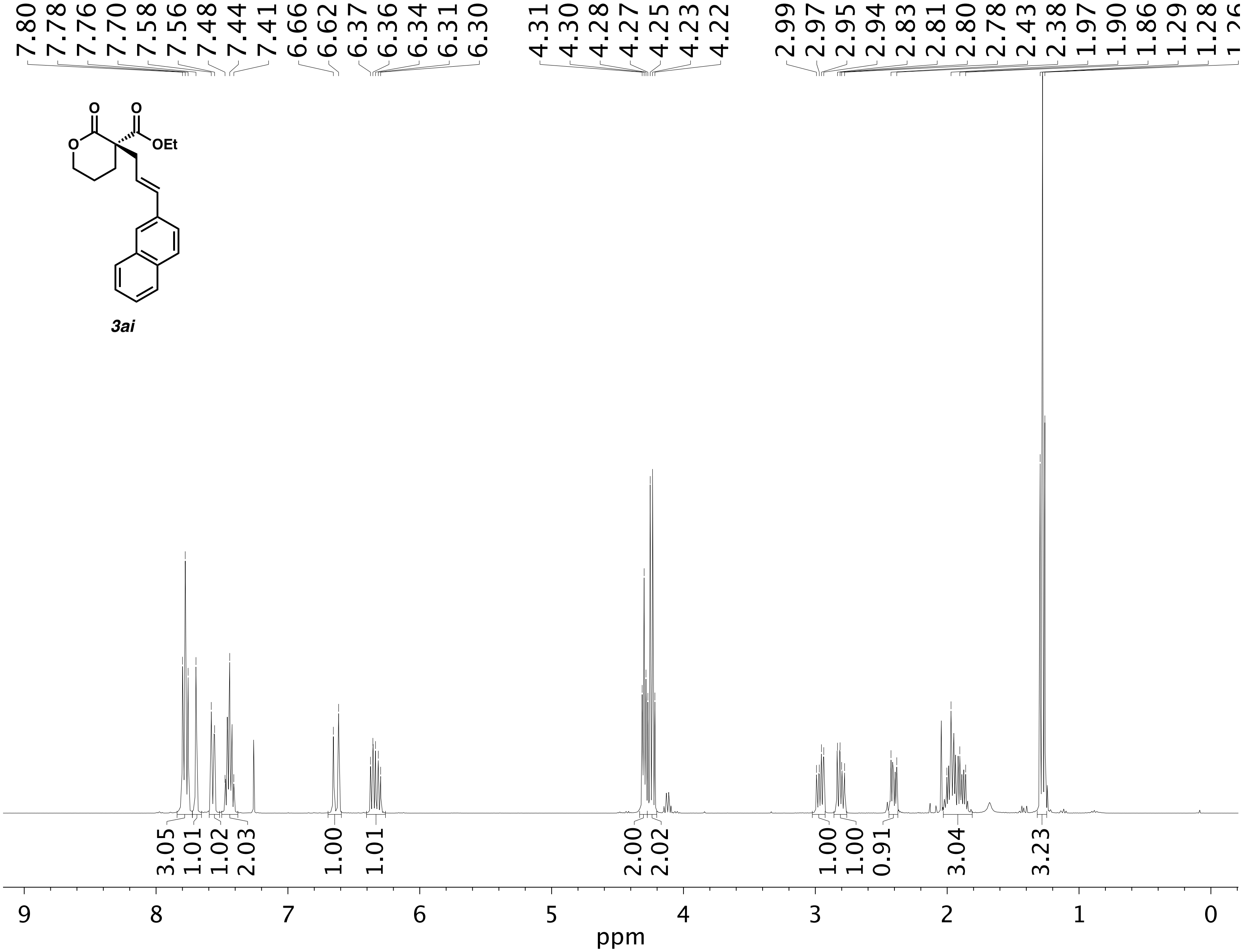

^{1}H NMR (400 MHz, $CDCl_3$) of compound **3ai**.

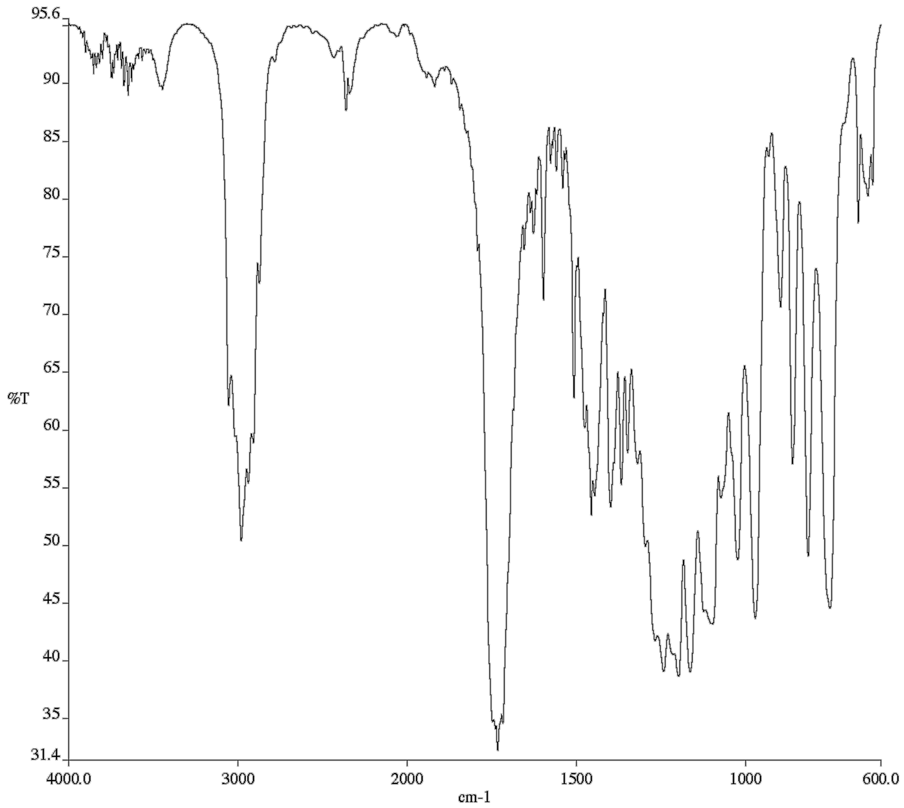

Infrared spectrum (Thin Film, NaCl) of compound **3ai**.

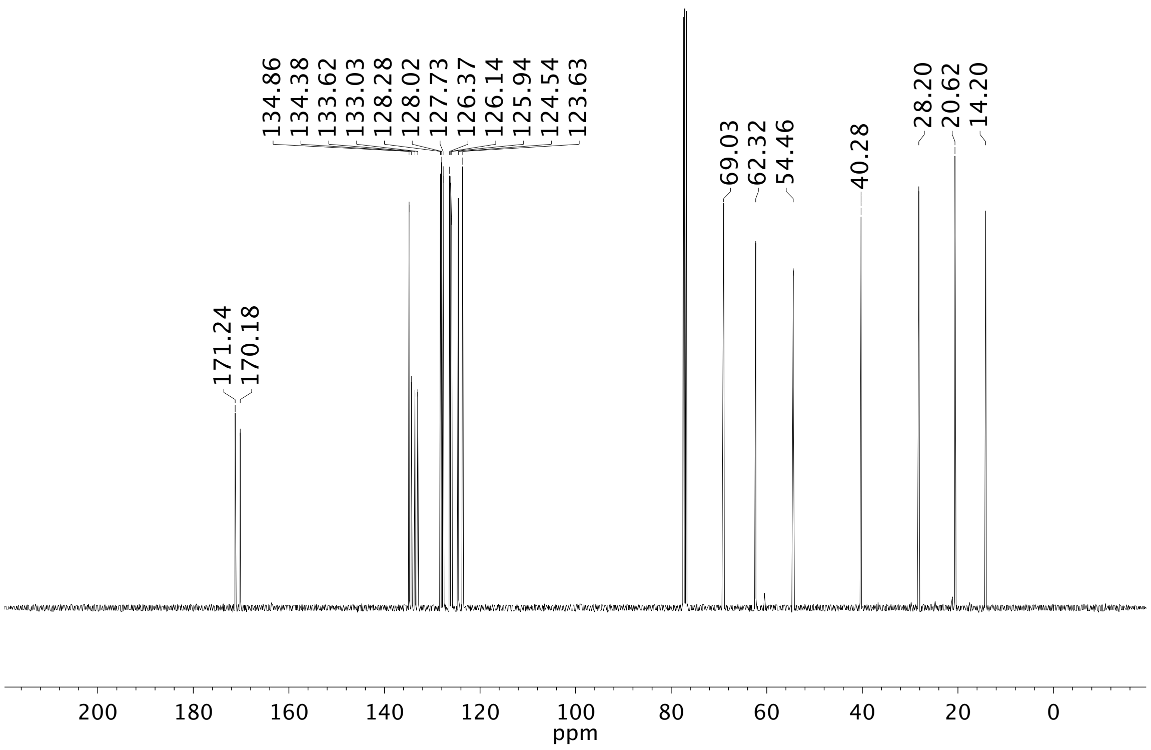

^{13}C NMR (101 MHz, $CDCl_3$) of compound **3ai**.

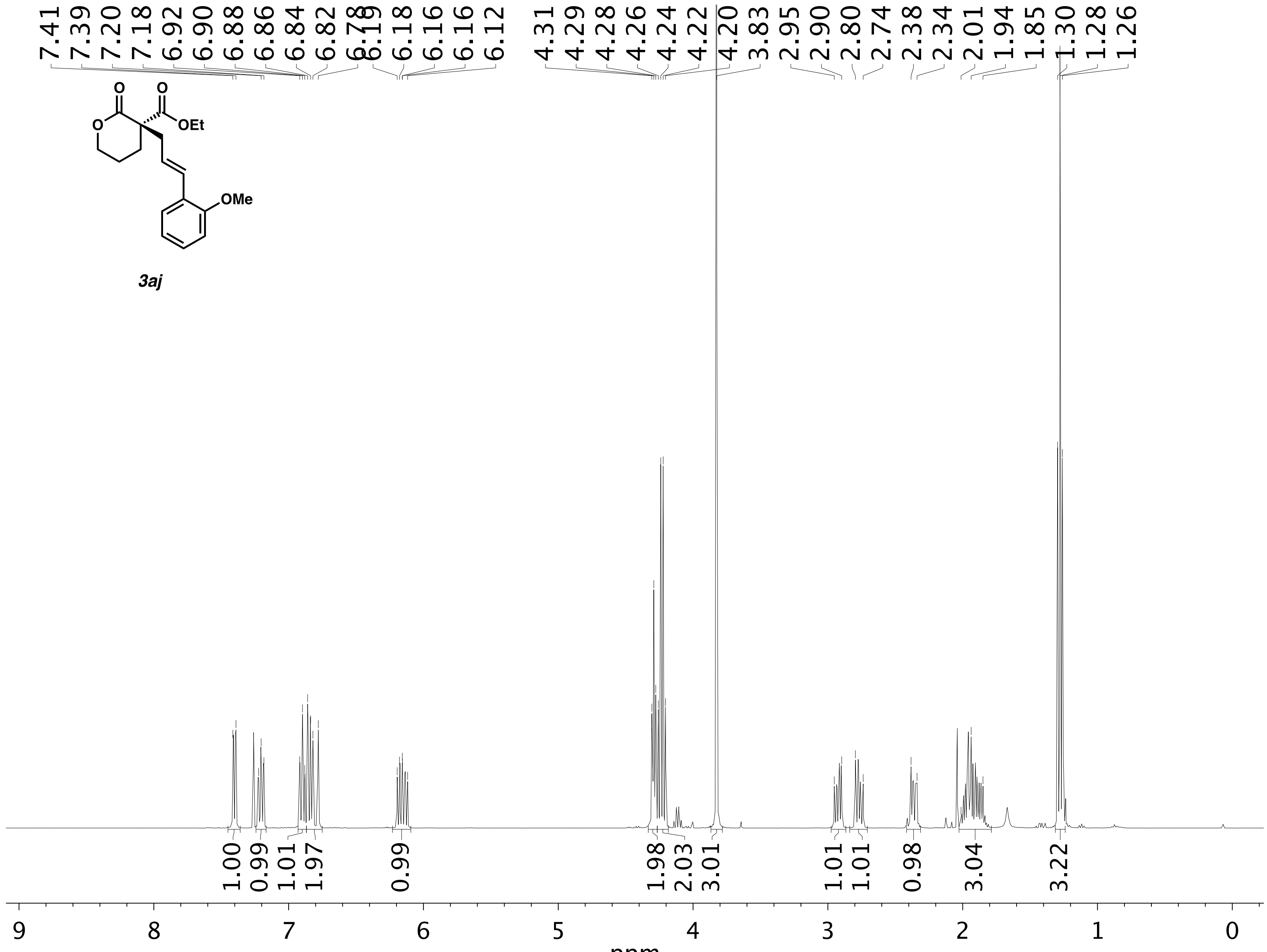

^1H NMR (400 MHz, $CDCl_3$) of compound **3aj**.

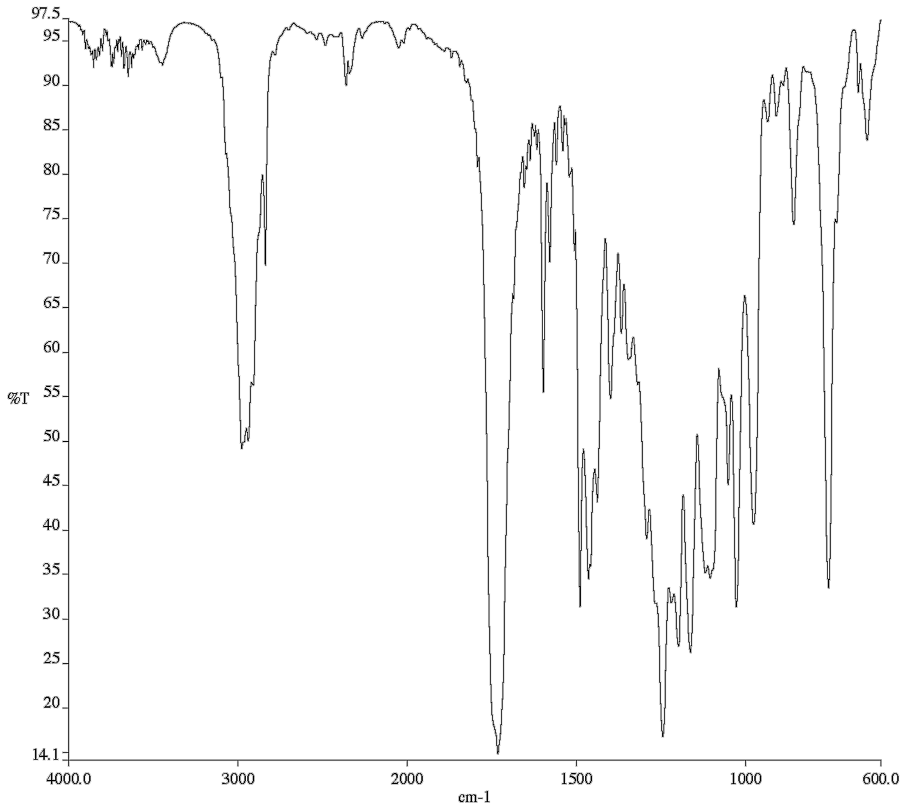

Infrared spectrum (Thin Film, NaCl) of compound **3aj**.

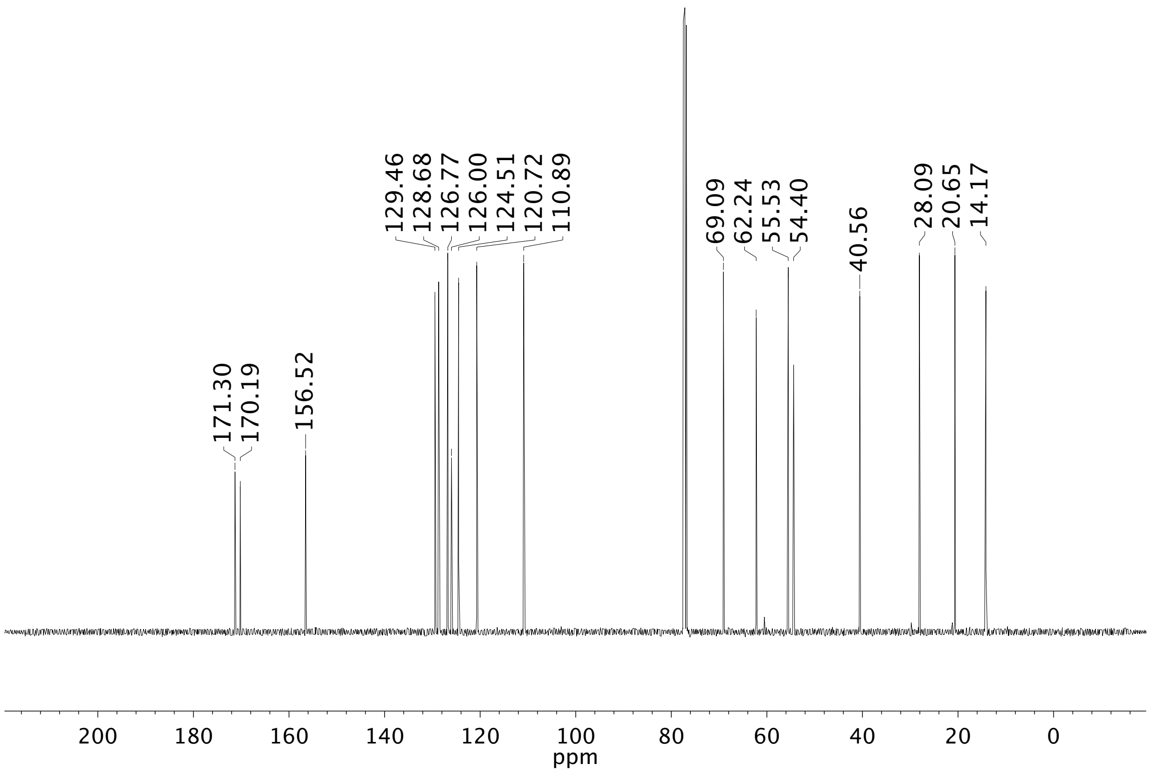

^{13}C NMR (101 MHz, $CDCl_3$) of compound **3aj**.

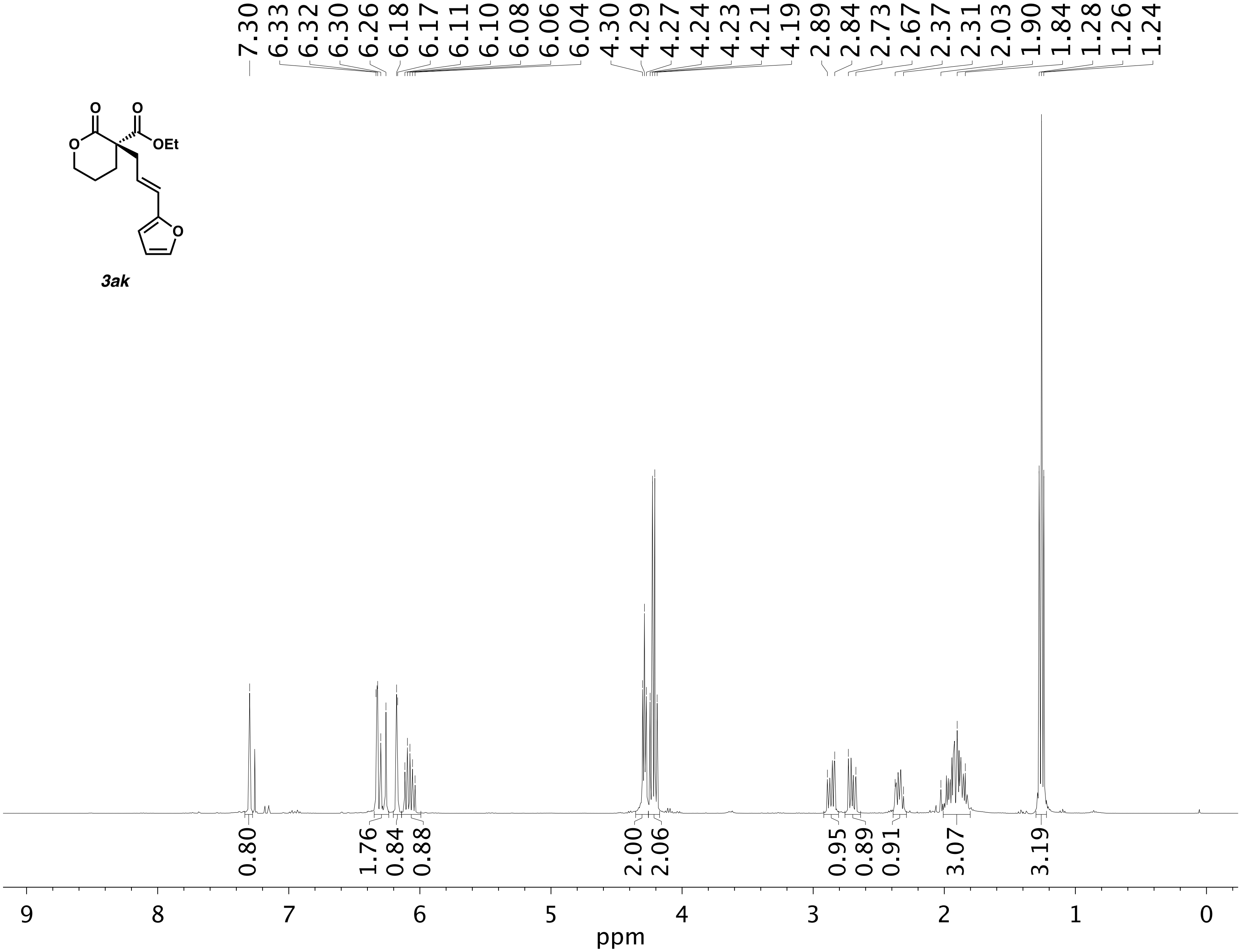

^{1}H NMR (400 MHz, $CDCl_3$) of compound **3ak**.

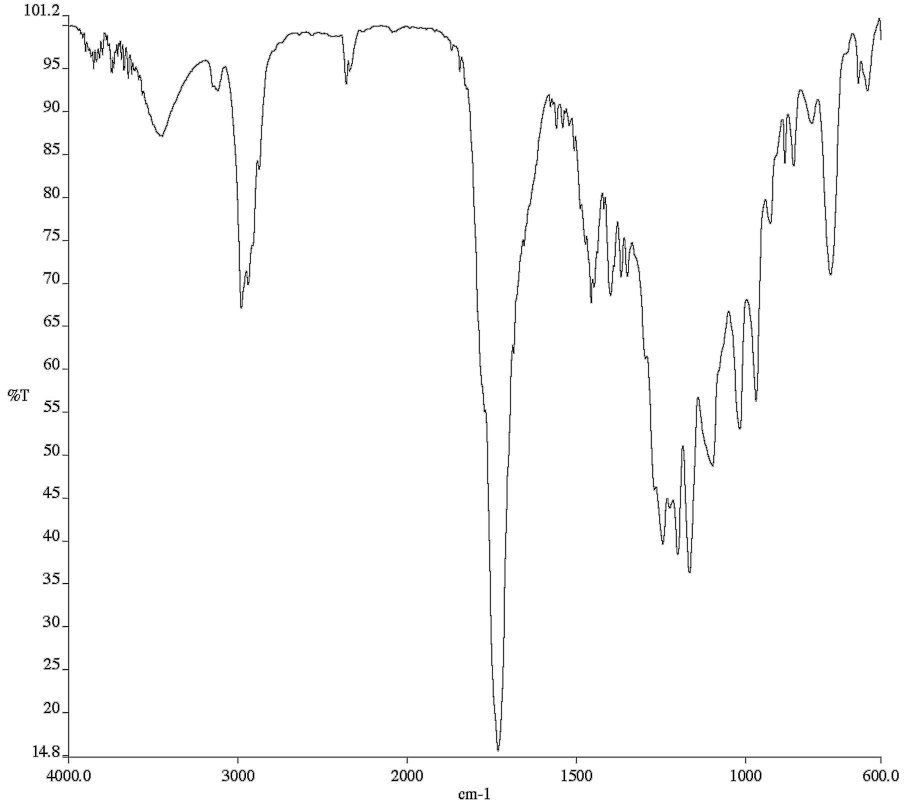

Infrared spectrum (Thin Film, NaCl) of compound **3ak**.

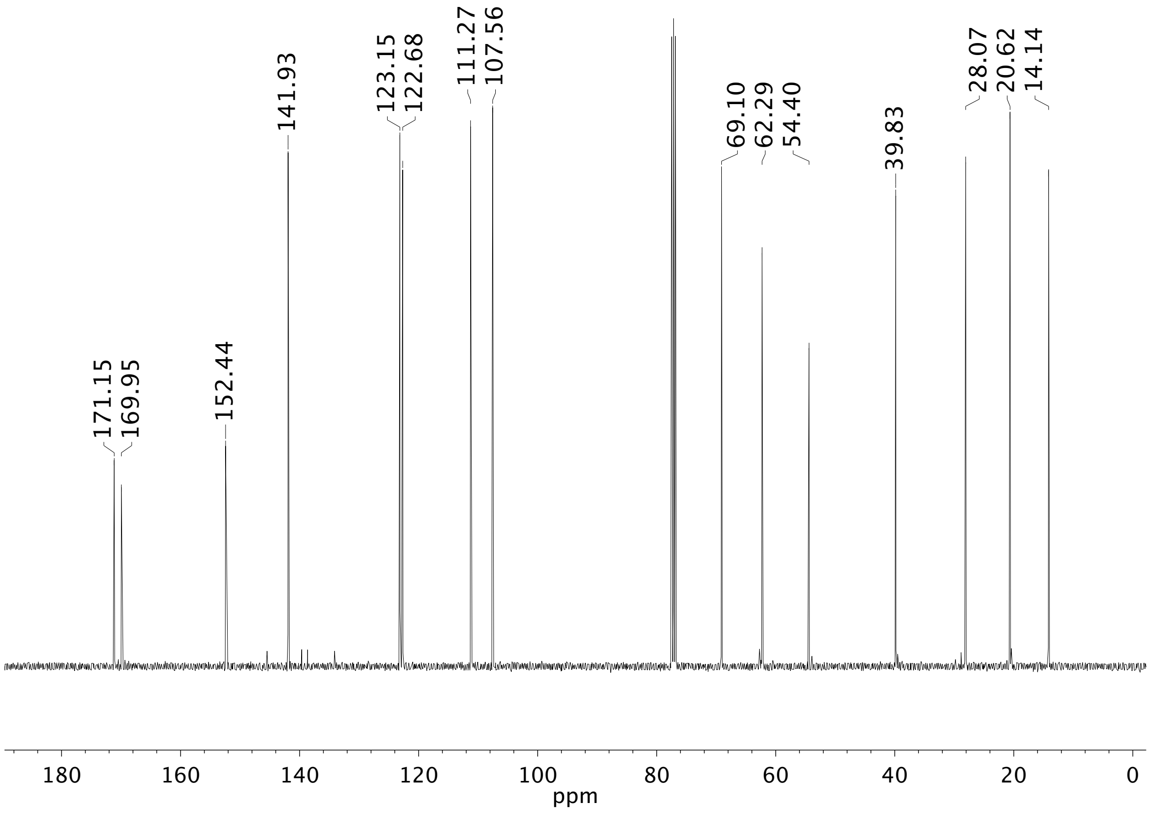

^{13}C NMR (101 MHz, $CDCl_3$) of compound **3ak**.

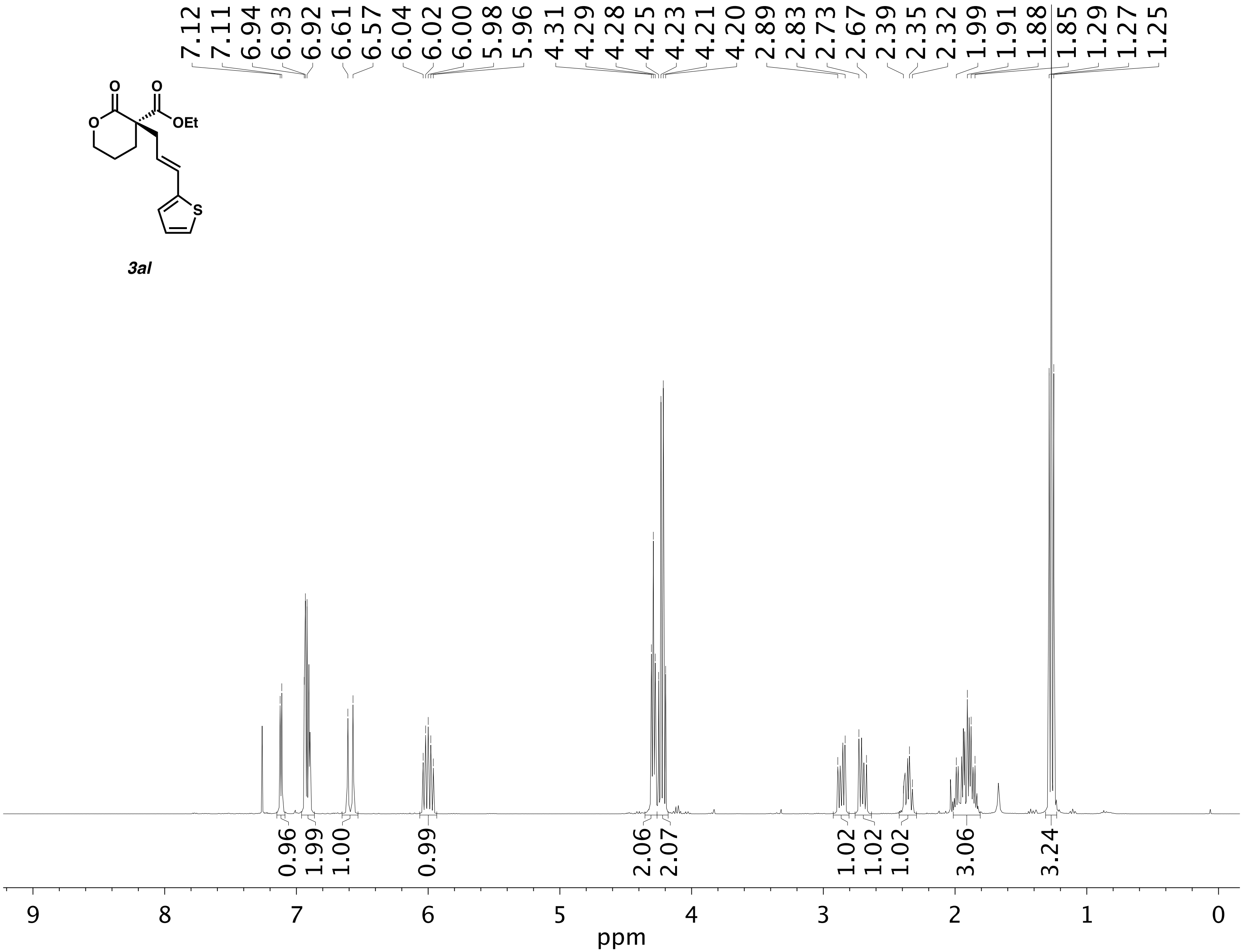

^{1}H NMR (400 MHz, $CDCl_3$) of compound **3al**.

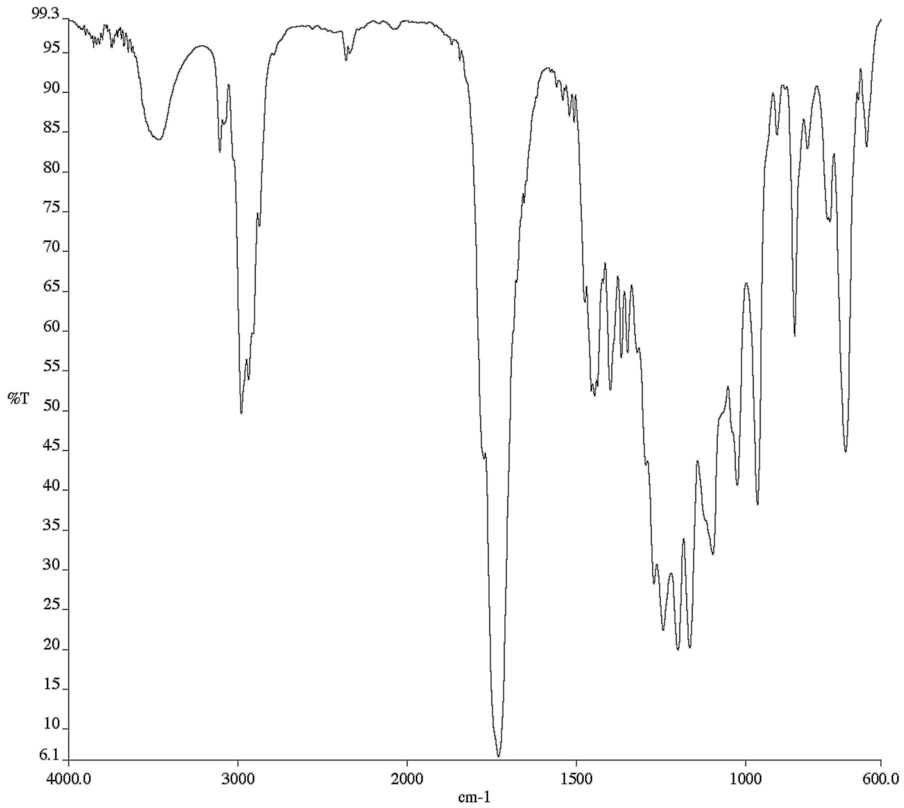

Infrared spectrum (Thin Film, NaCl) of compound **3al**.

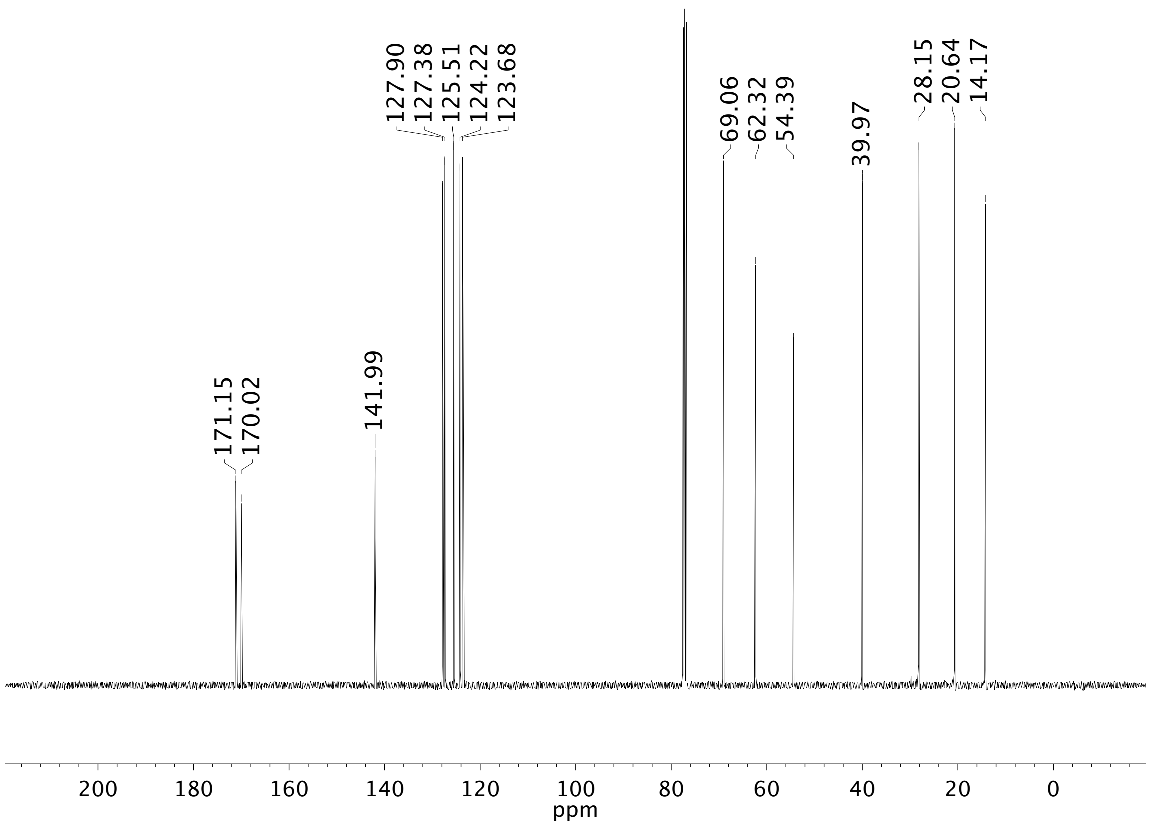

^{13}C NMR (101 MHz, $CDCl_3$) of compound **3al**.

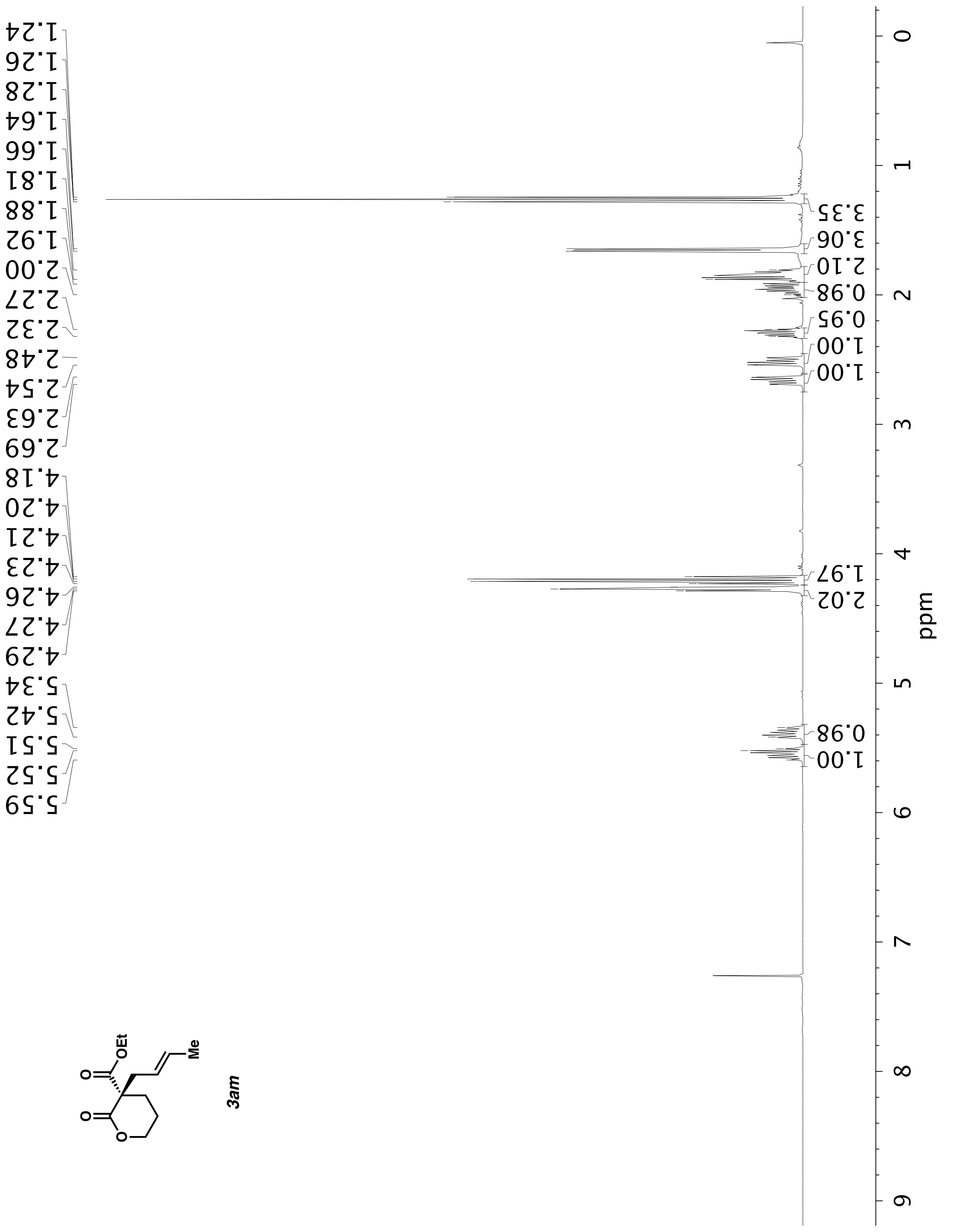

^{1}H NMR (400 MHz, $CDCl_3$) of compound **3am**.

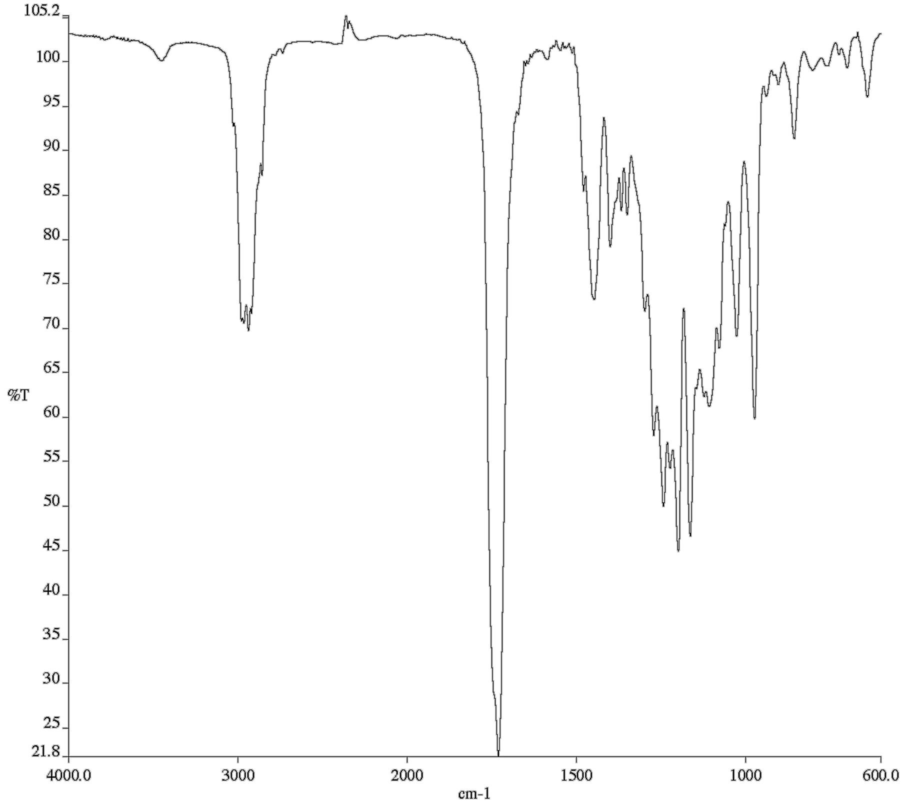

Infrared spectrum (Thin Film, NaCl) of compound **3am**.

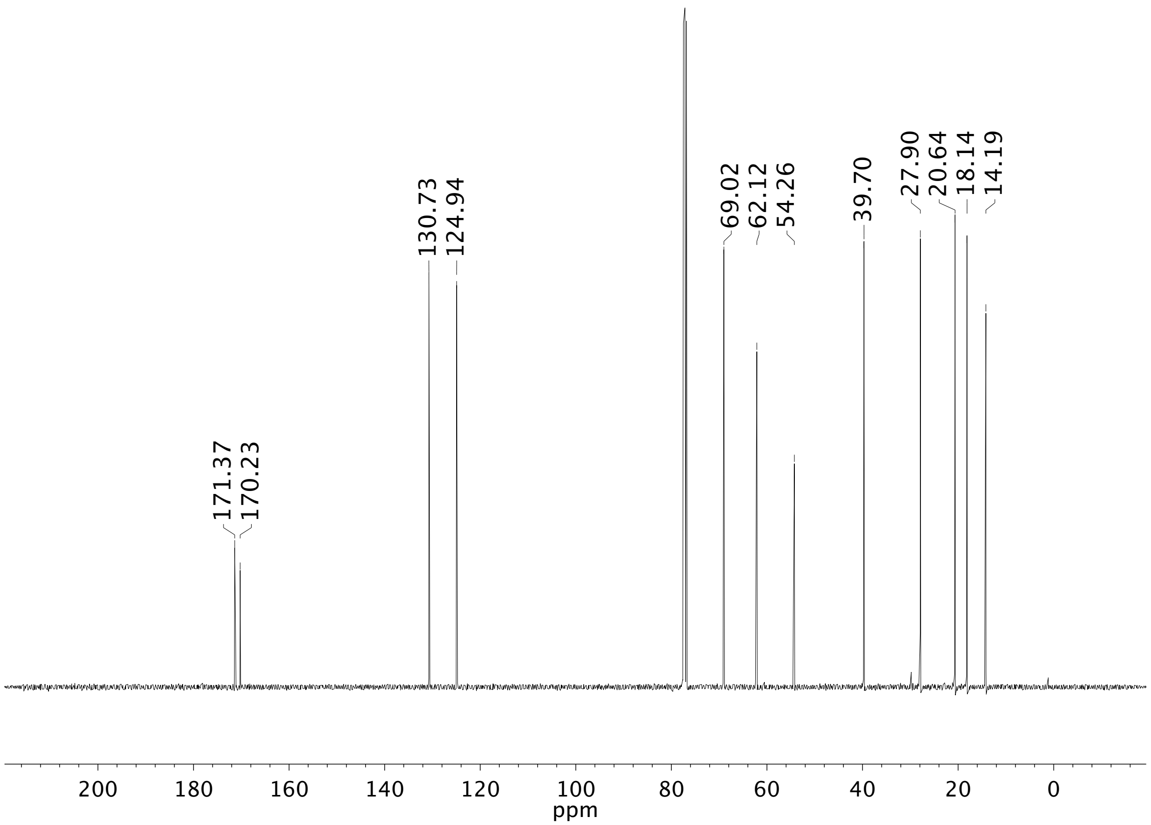

^{13}C NMR (101 MHz, $CDCl_3$) of compound **3am**.

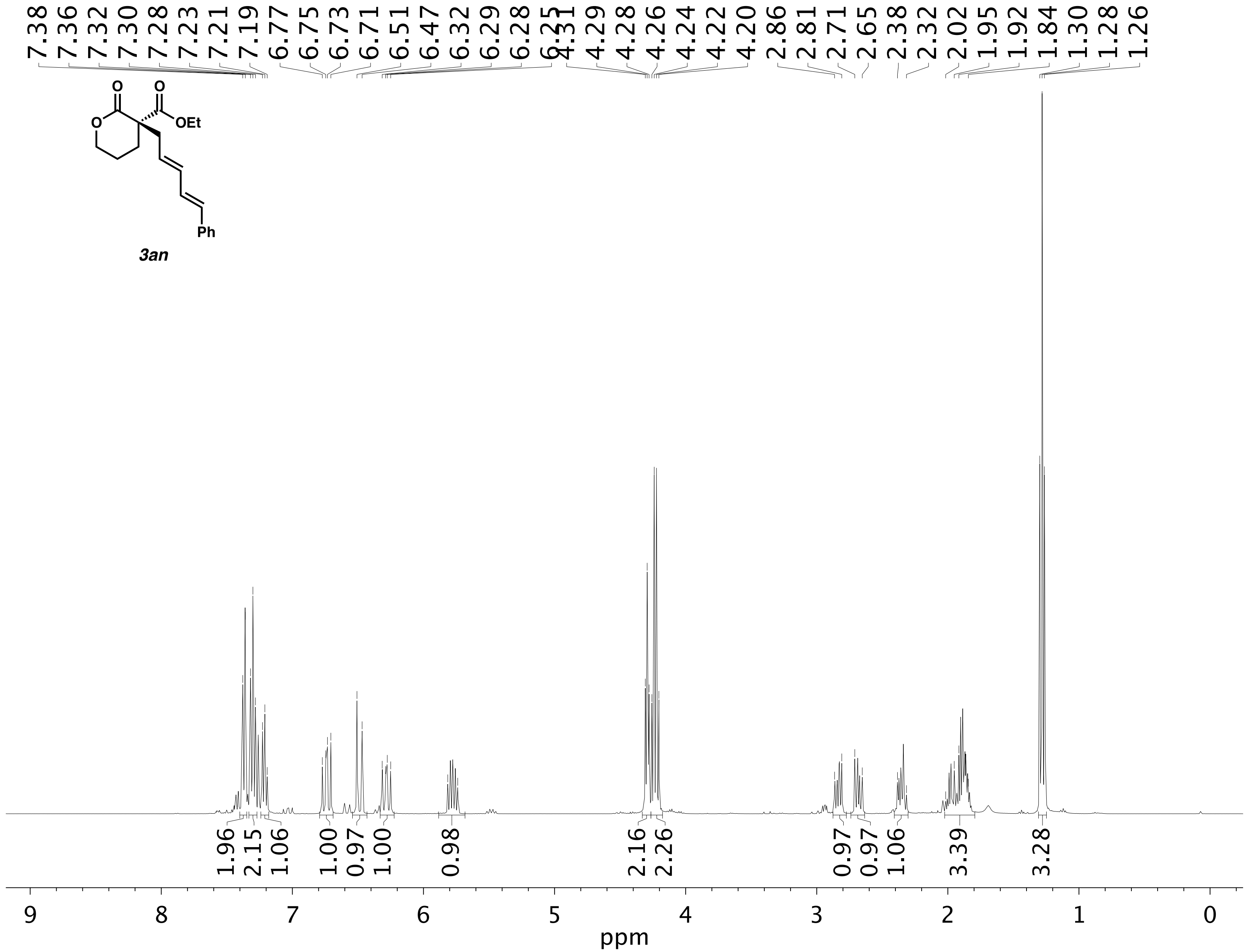

^{1}H NMR (400 MHz, $CDCl_3$) of compound **3an**.

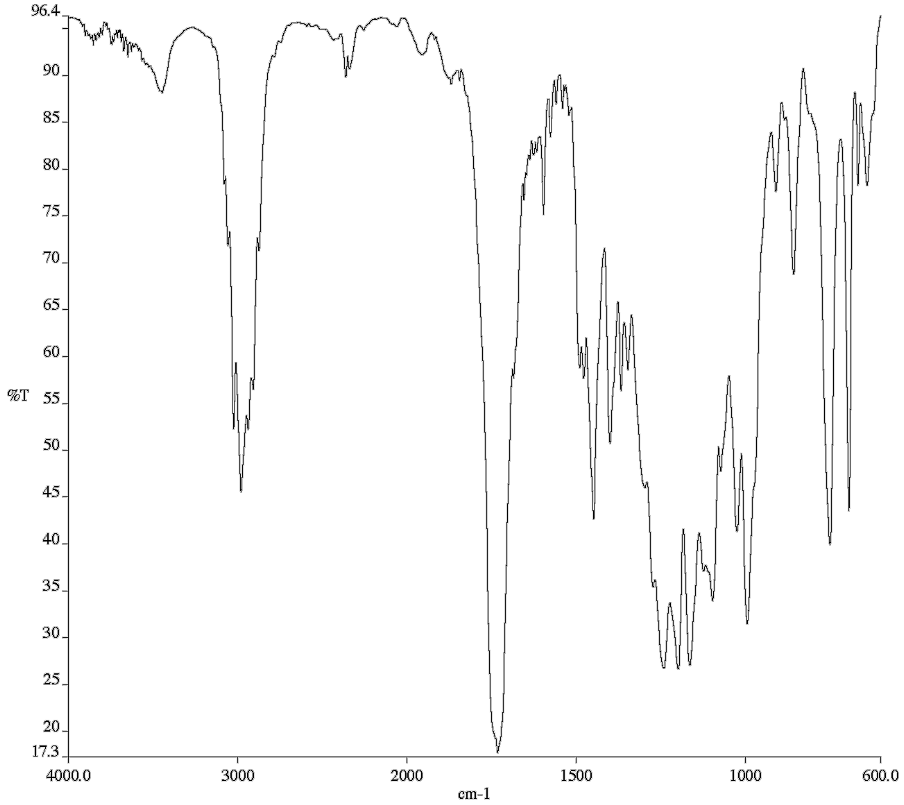

Infrared spectrum (Thin Film, NaCl) of compound **3an**.

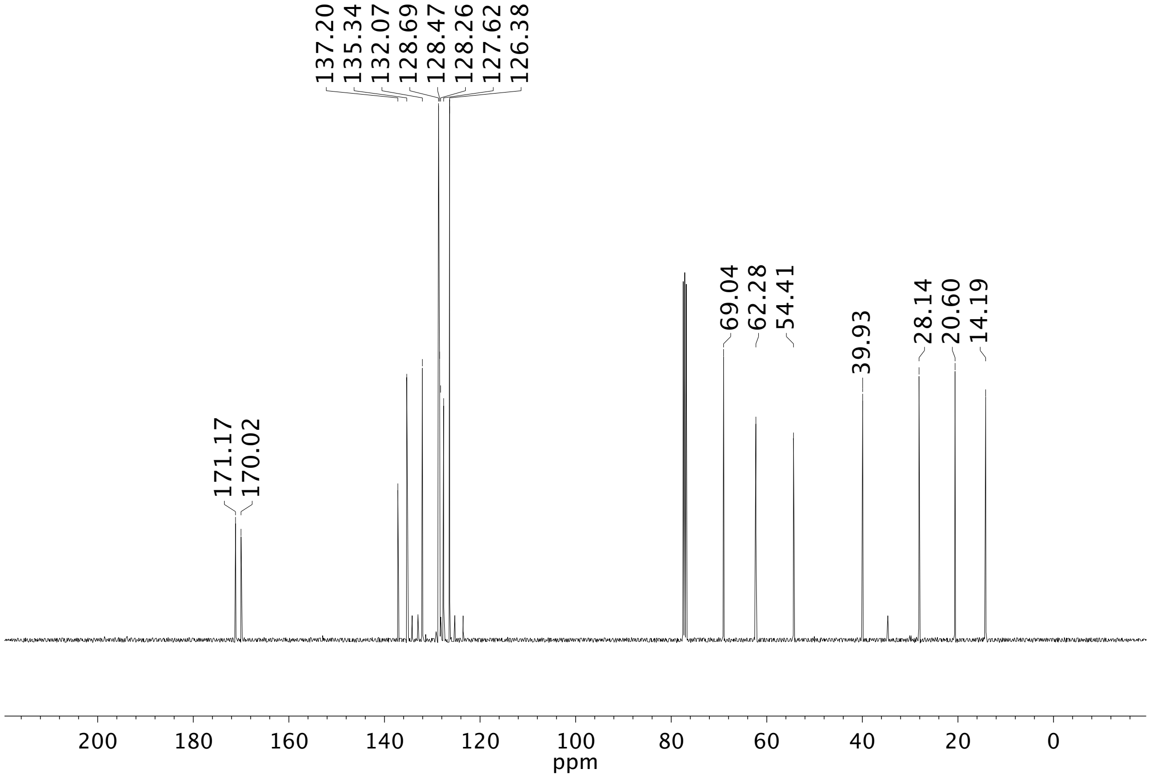

^{13}C NMR (101 MHz, $CDCl_3$) of compound **3an**.

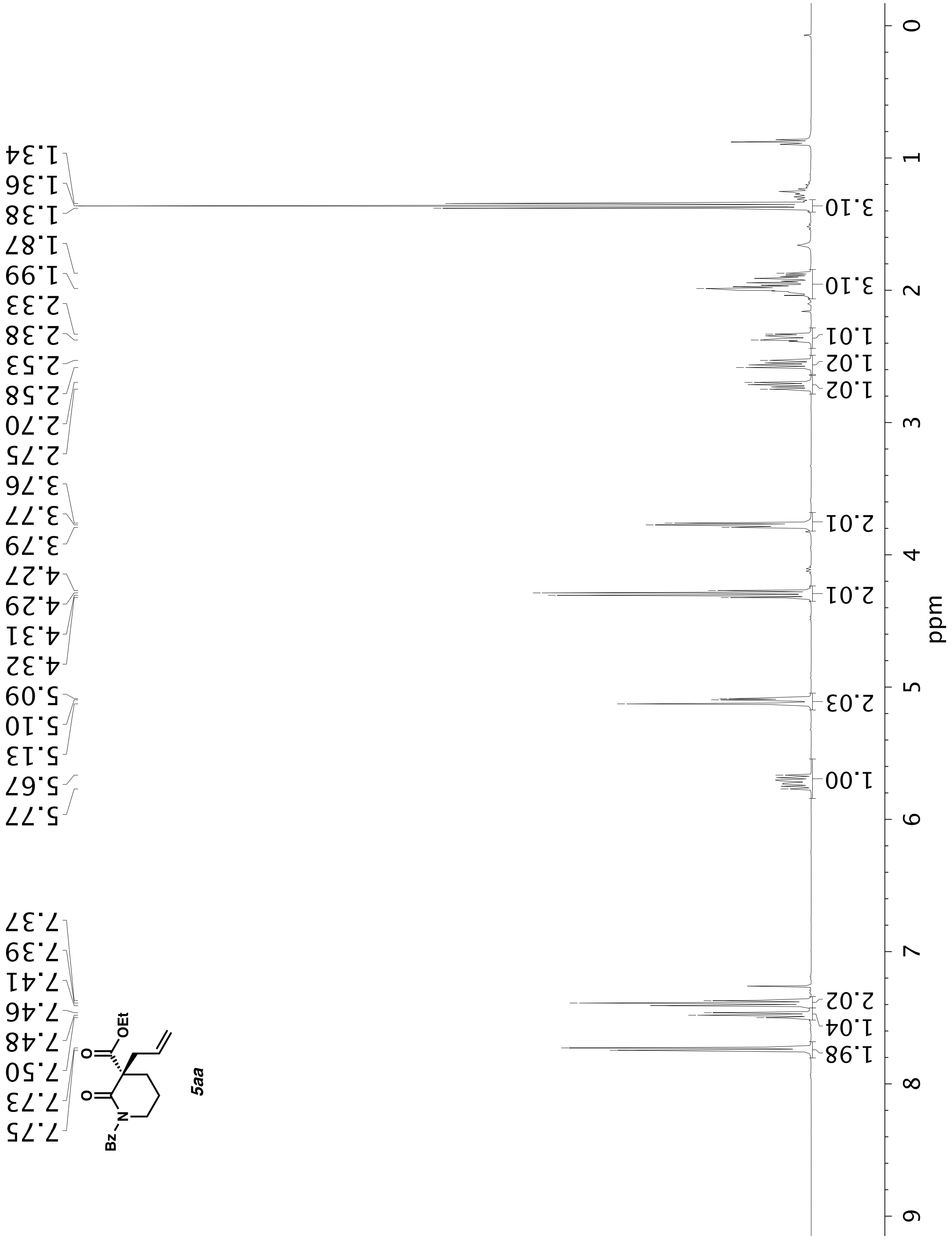

^{1}H NMR (400 MHz, $CDCl_3$) of compound **5aa**.

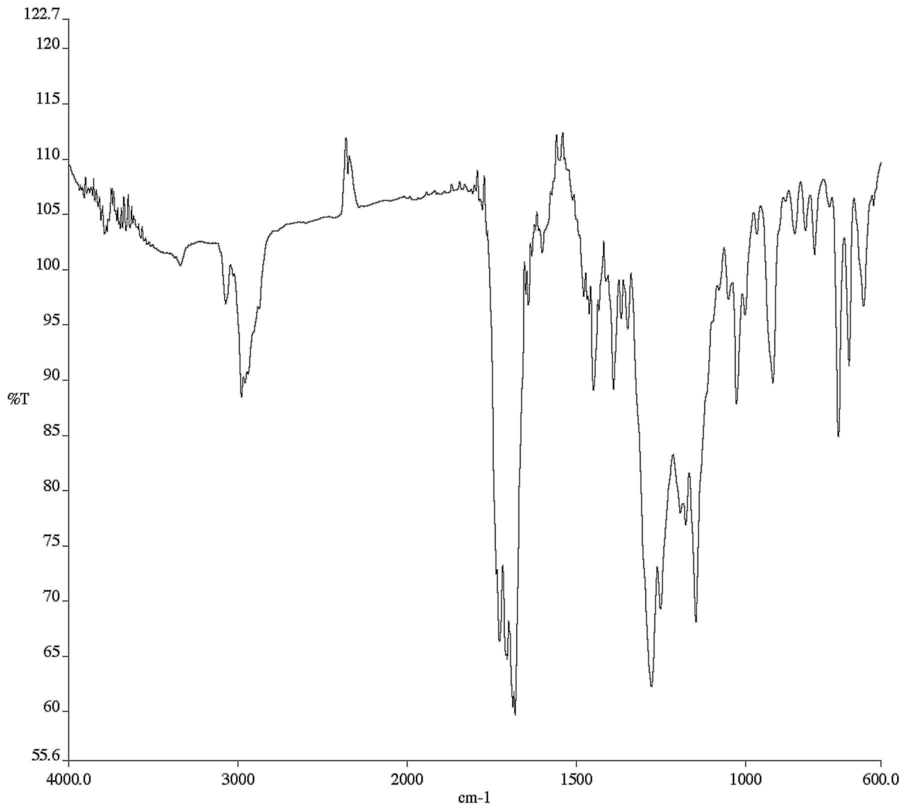

Infrared spectrum (Thin Film, NaCl) of compound **5aa**.

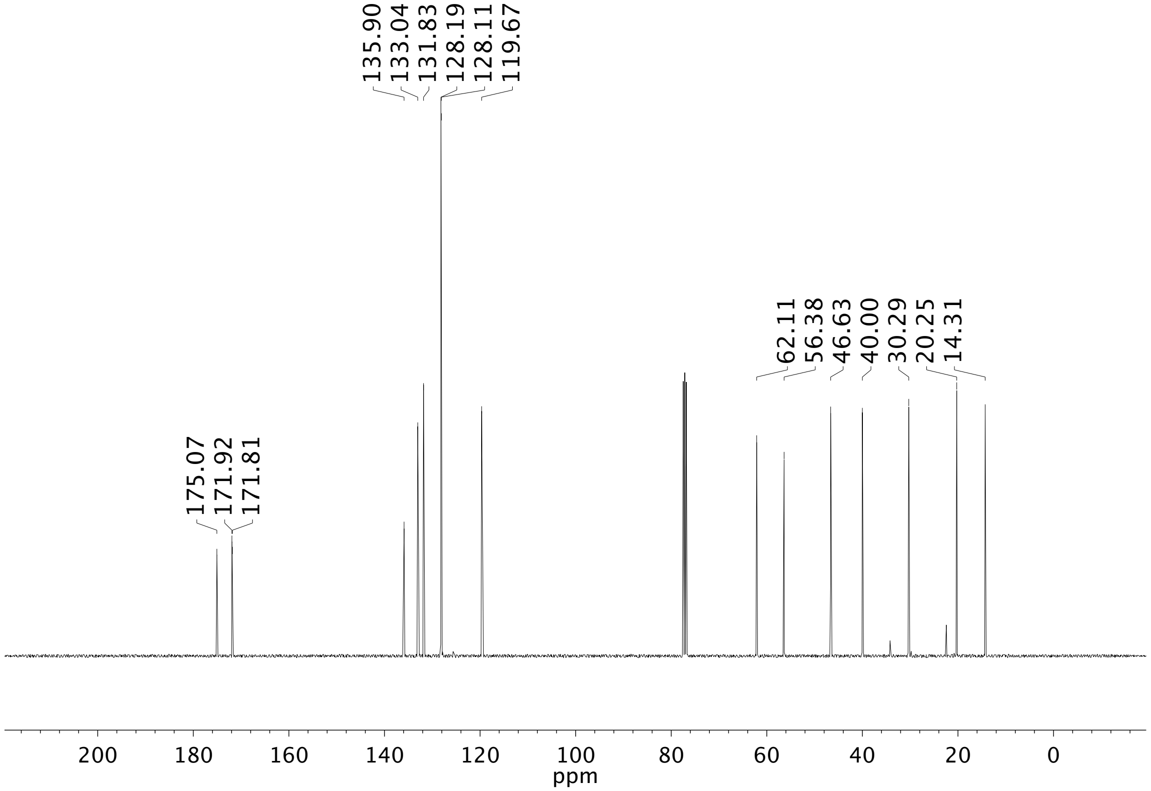

^{13}C NMR (101 MHz, $CDCl_3$) of compound **5aa**.

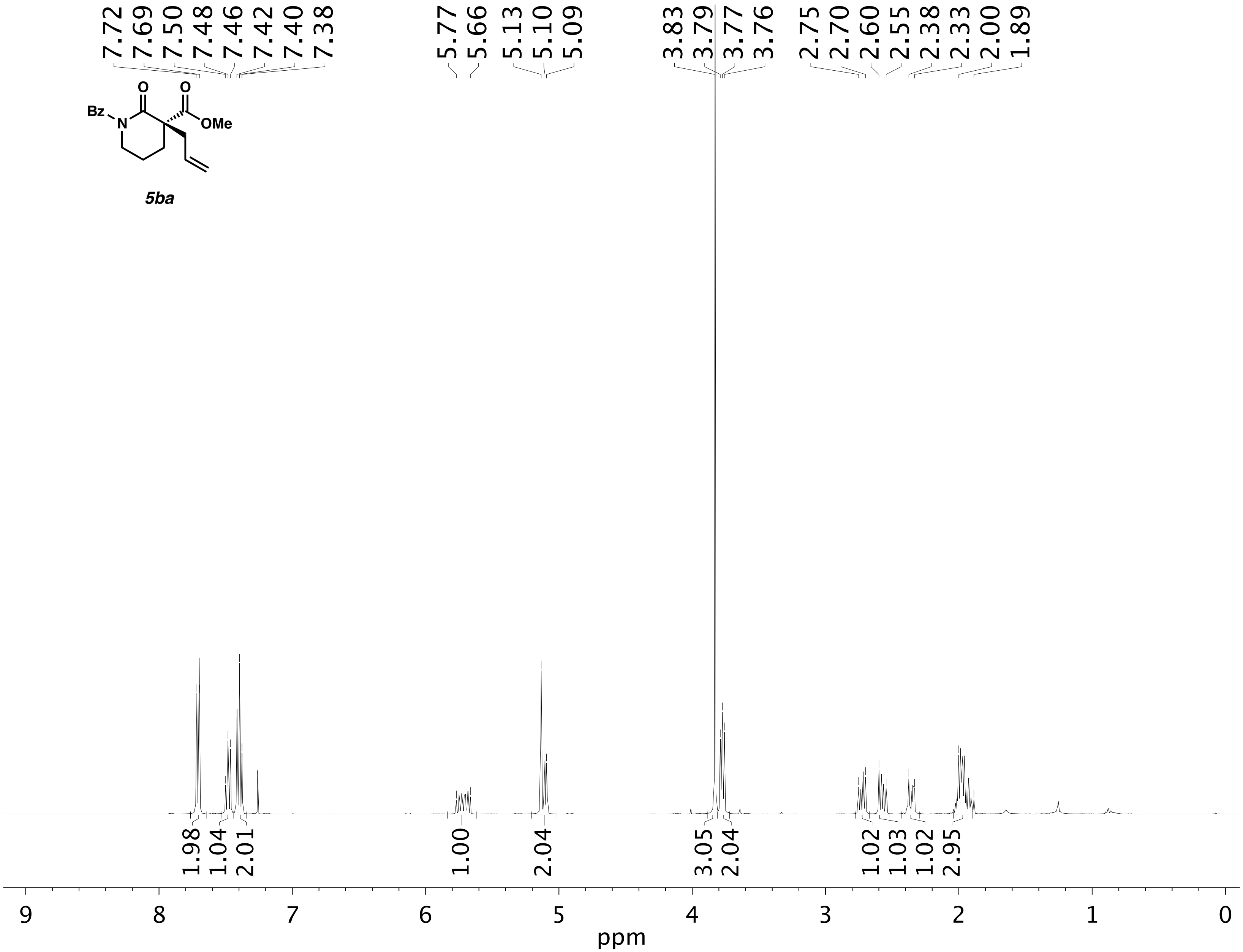

^{1}H NMR (400 MHz, $CDCl_3$) of compound **5ba**.

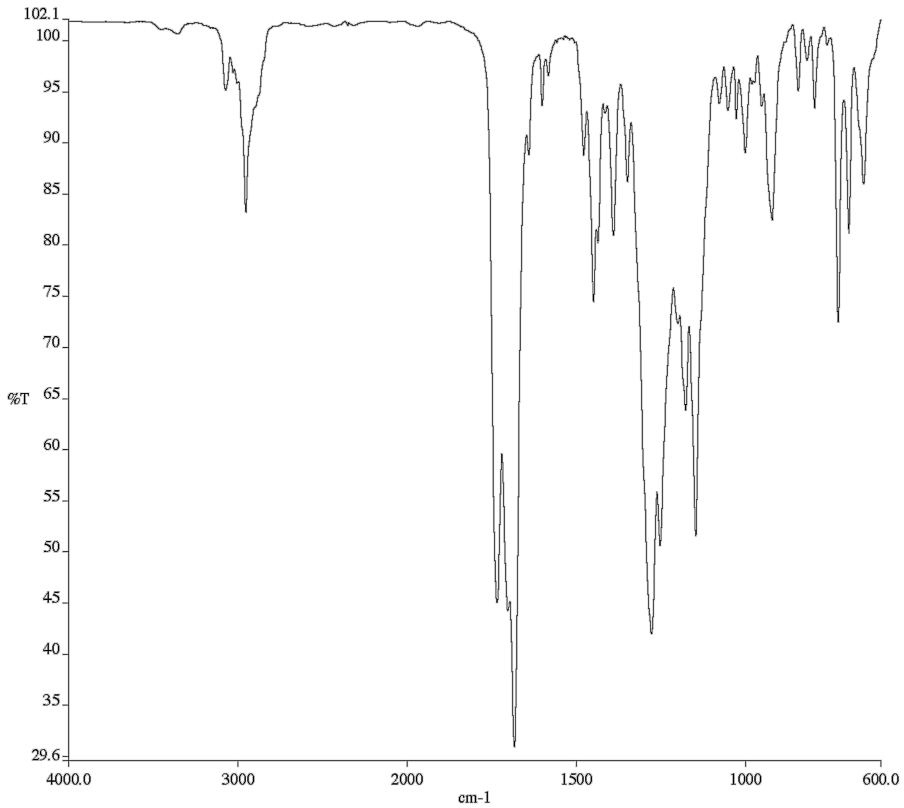

Infrared spectrum (Thin Film, NaCl) of compound **5ba**.

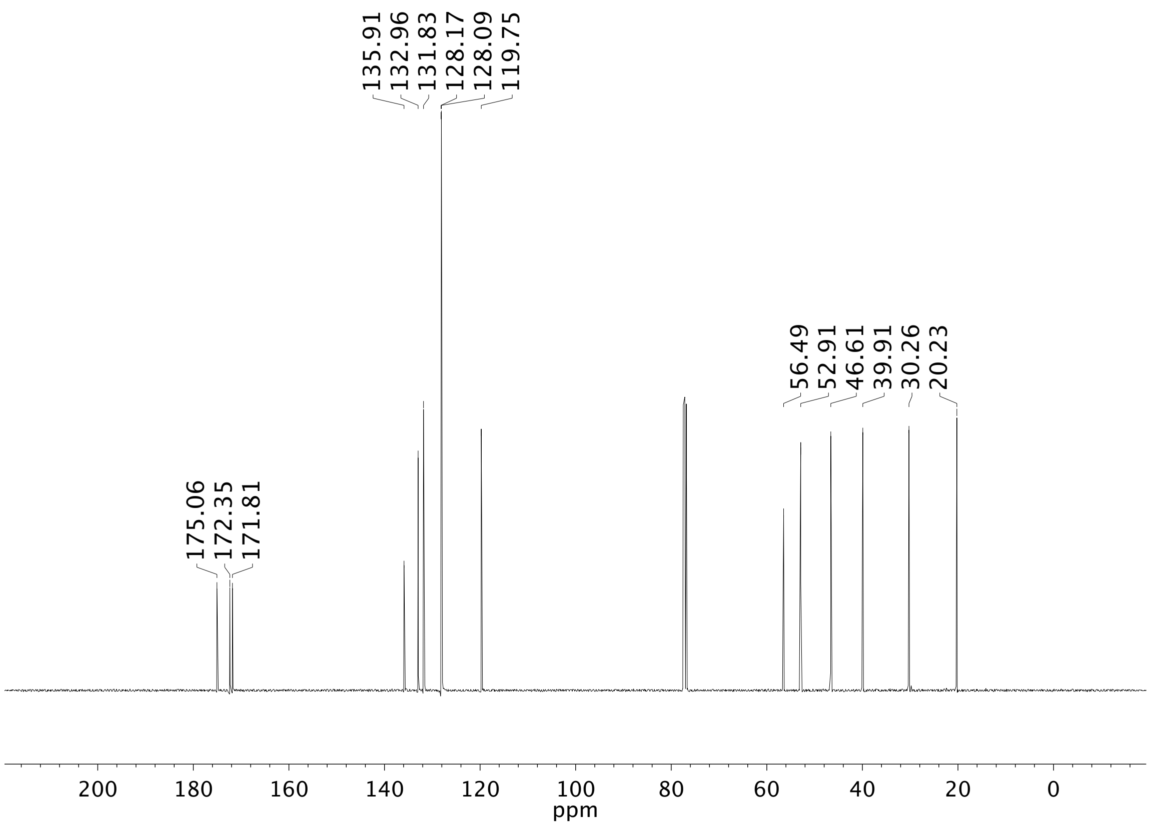

^{13}C NMR (101 MHz, $CDCl_3$) of compound **5ba**.

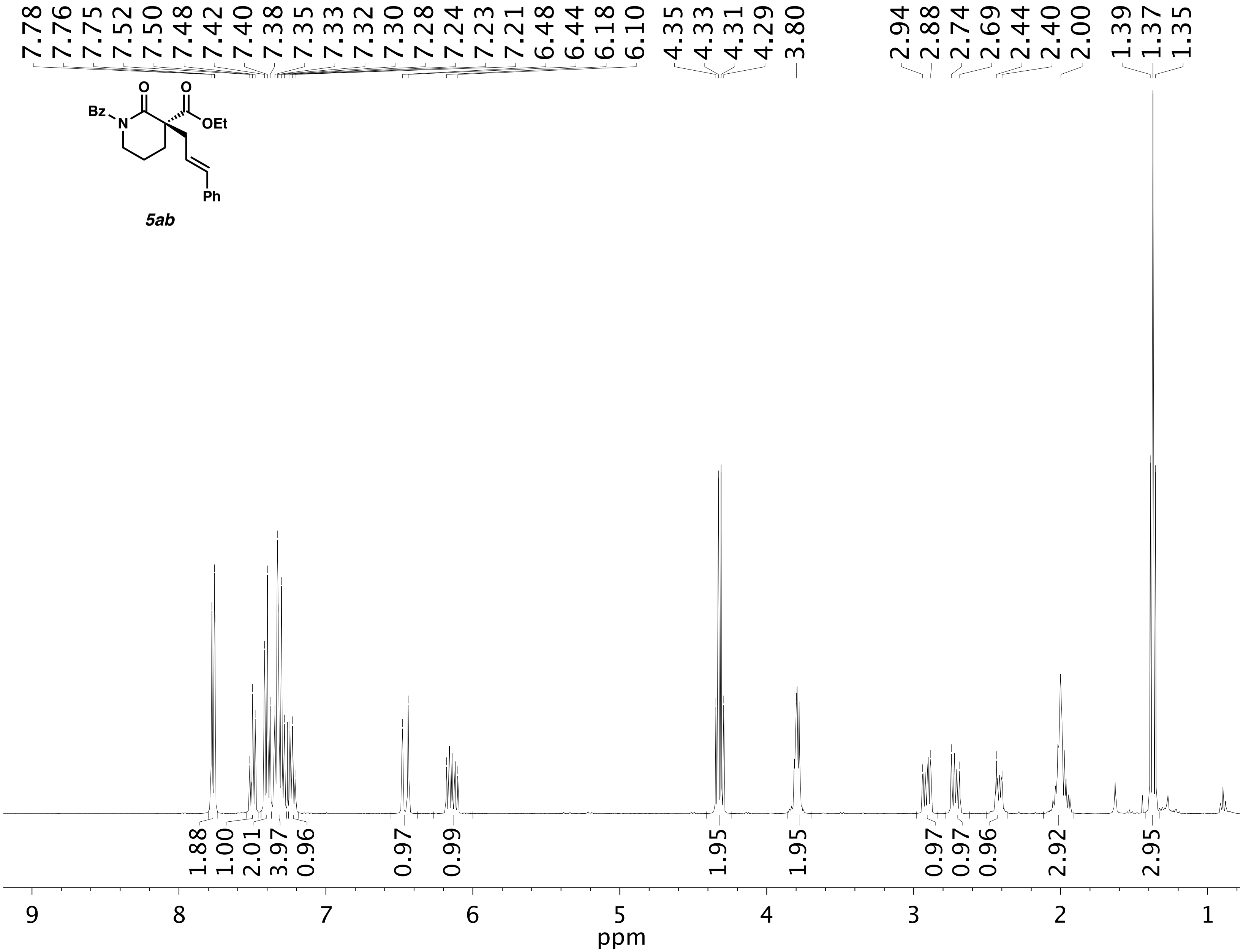

^{1}H NMR (400 MHz, $CDCl_3$) of compound **5ab**.

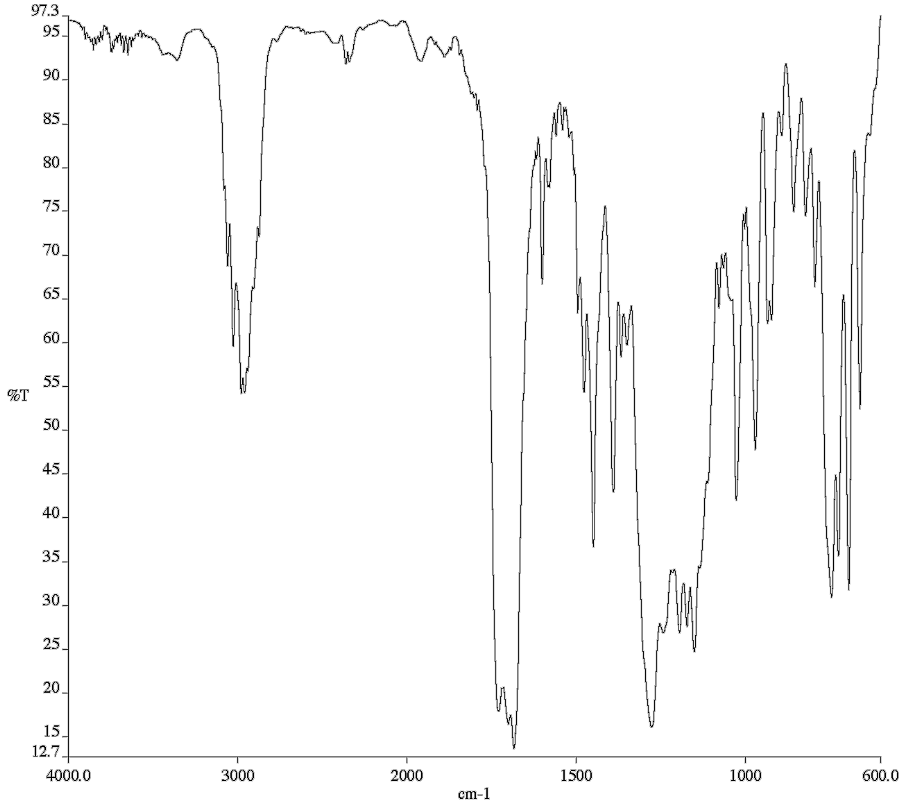

Infrared spectrum (Thin Film, NaCl) of compound **5ab**.

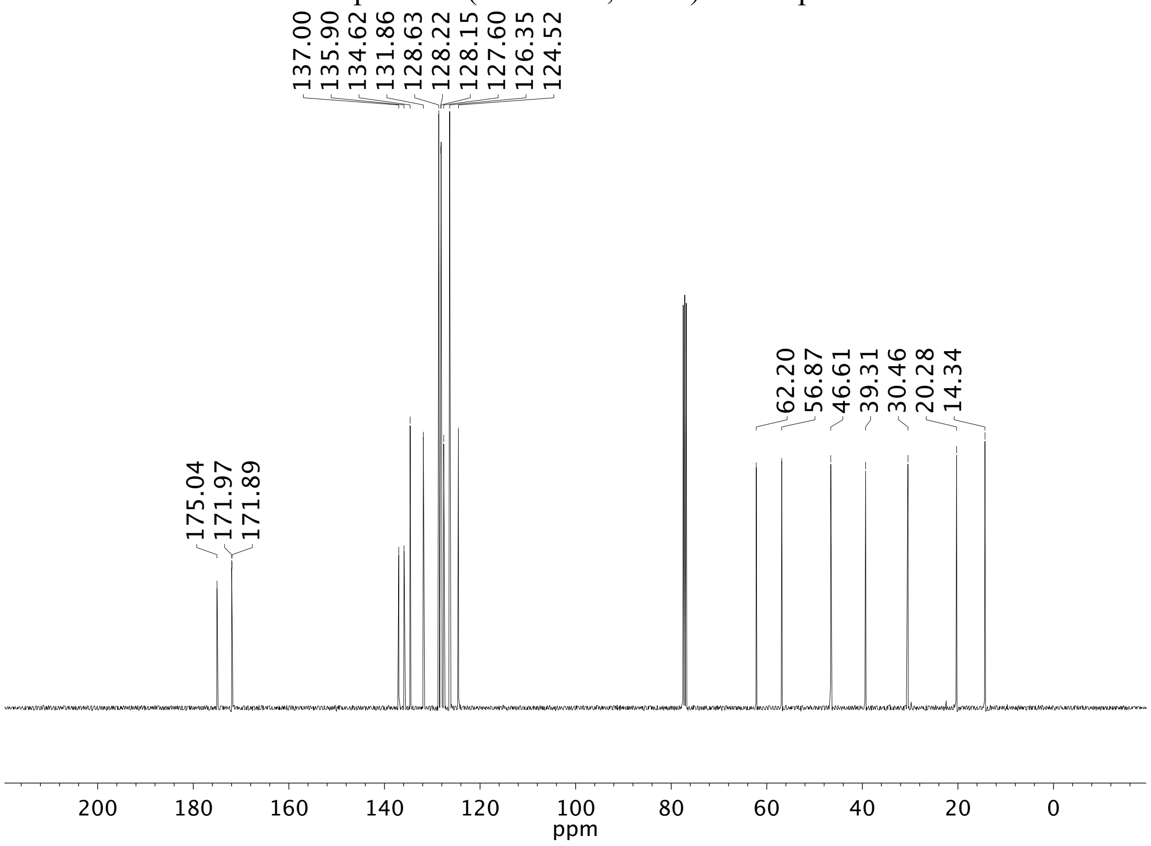

^{13}C NMR (101 MHz, $CDCl_3$) of compound **5ab**.

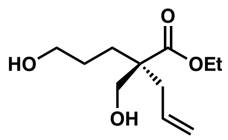

6

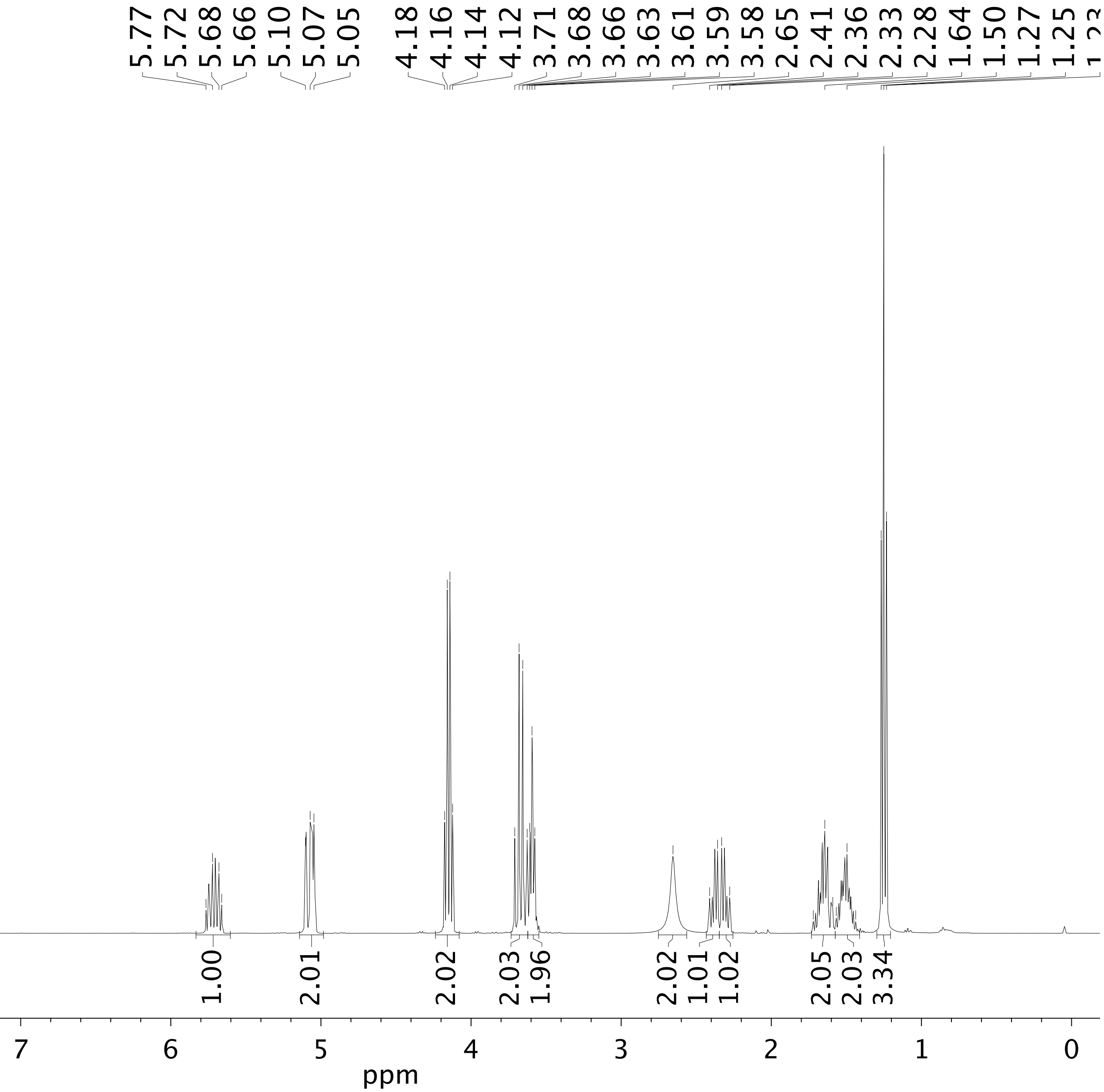

^{1}H NMR (400 MHz, $CDCl_3$) of compound **6**.

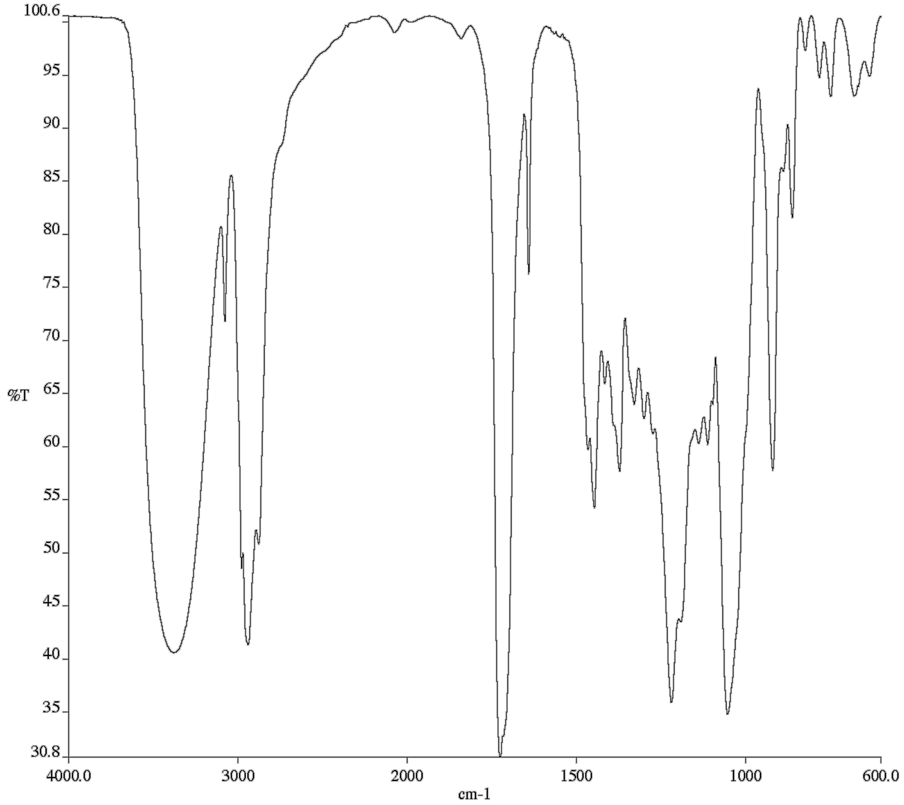

Infrared spectrum (Thin Film, NaCl) of compound **6**.

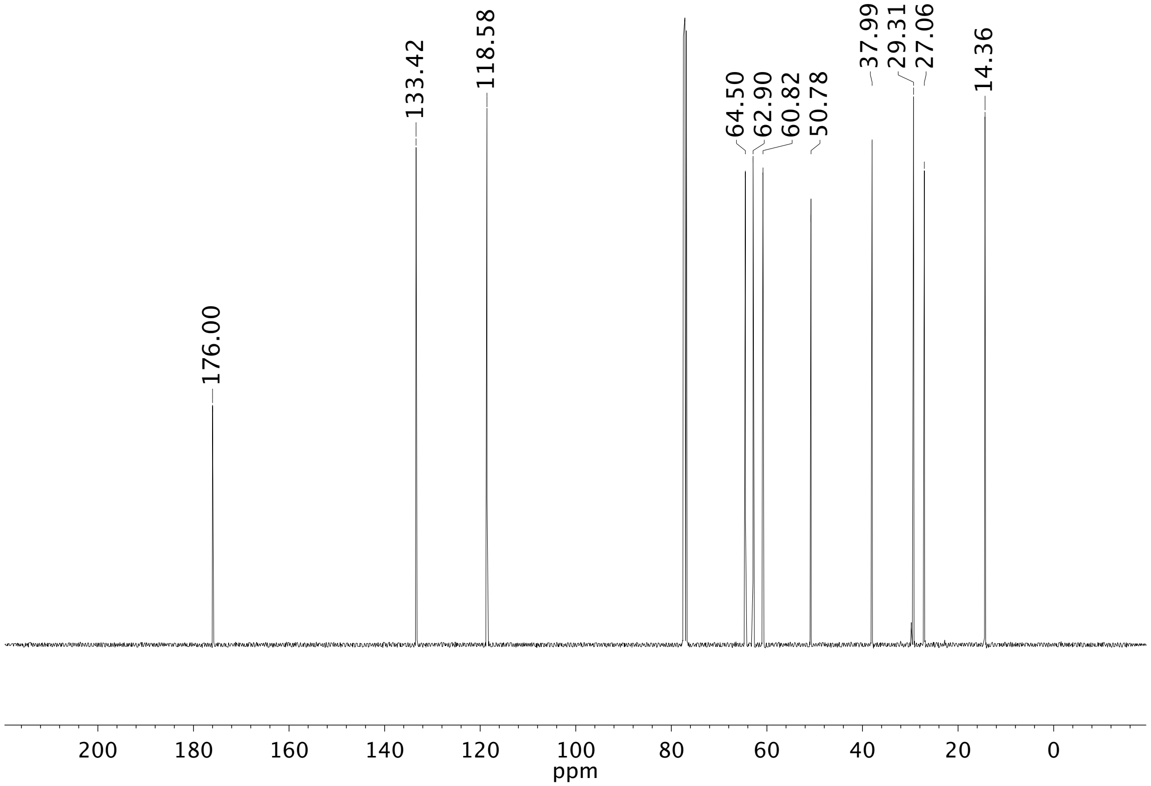

^{13}C NMR (101 MHz, $CDCl_3$) of compound **6**.

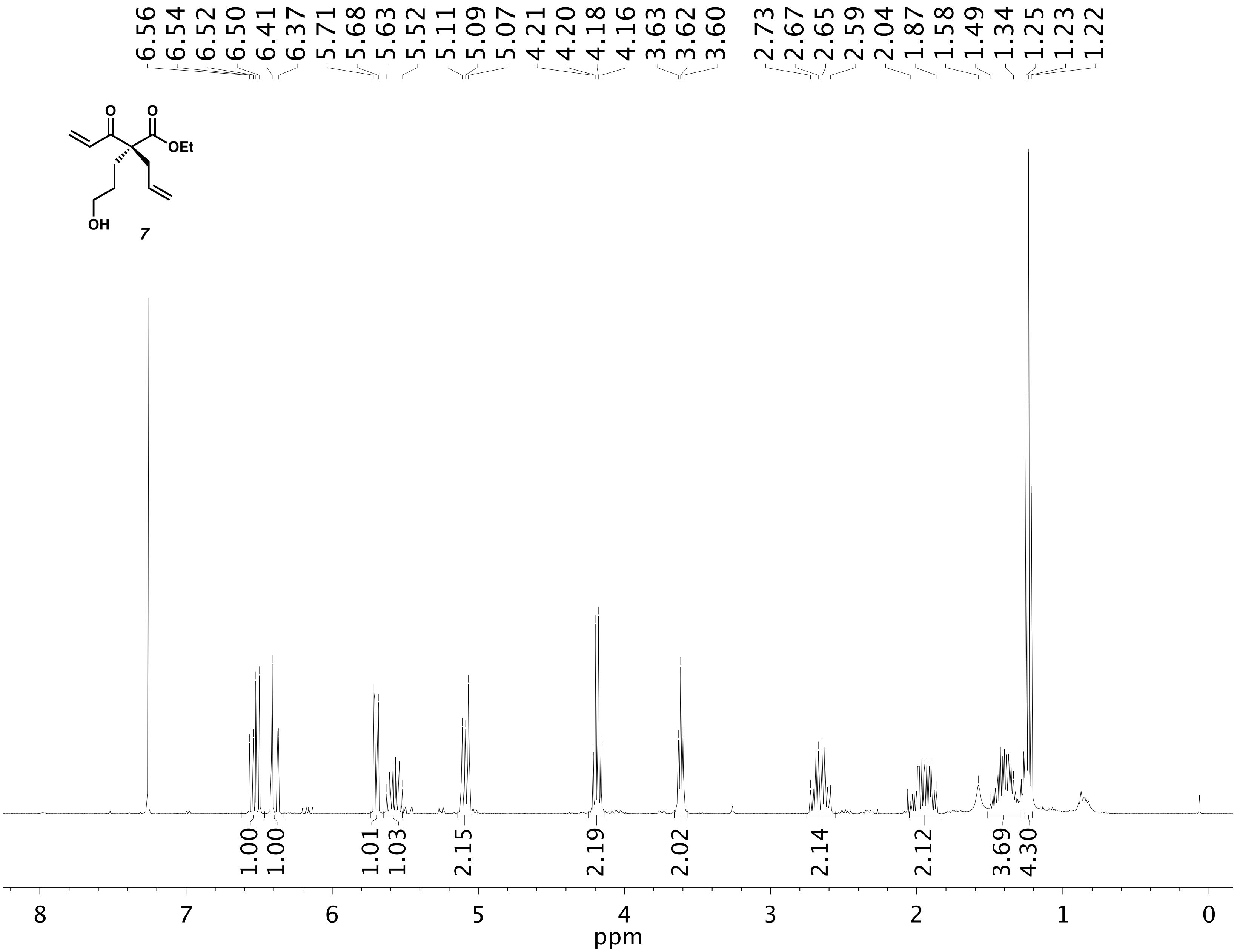

^{1}H NMR (400 MHz, $CDCl_3$) of compound **7**.

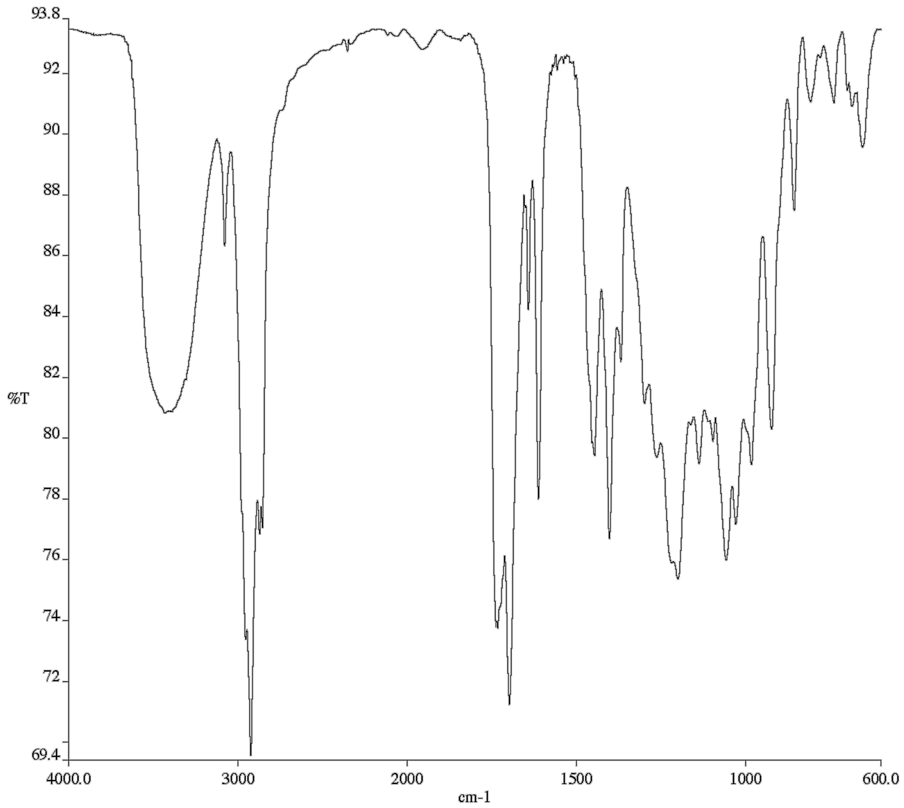

Infrared spectrum (Thin Film, NaCl) of compound **7**.

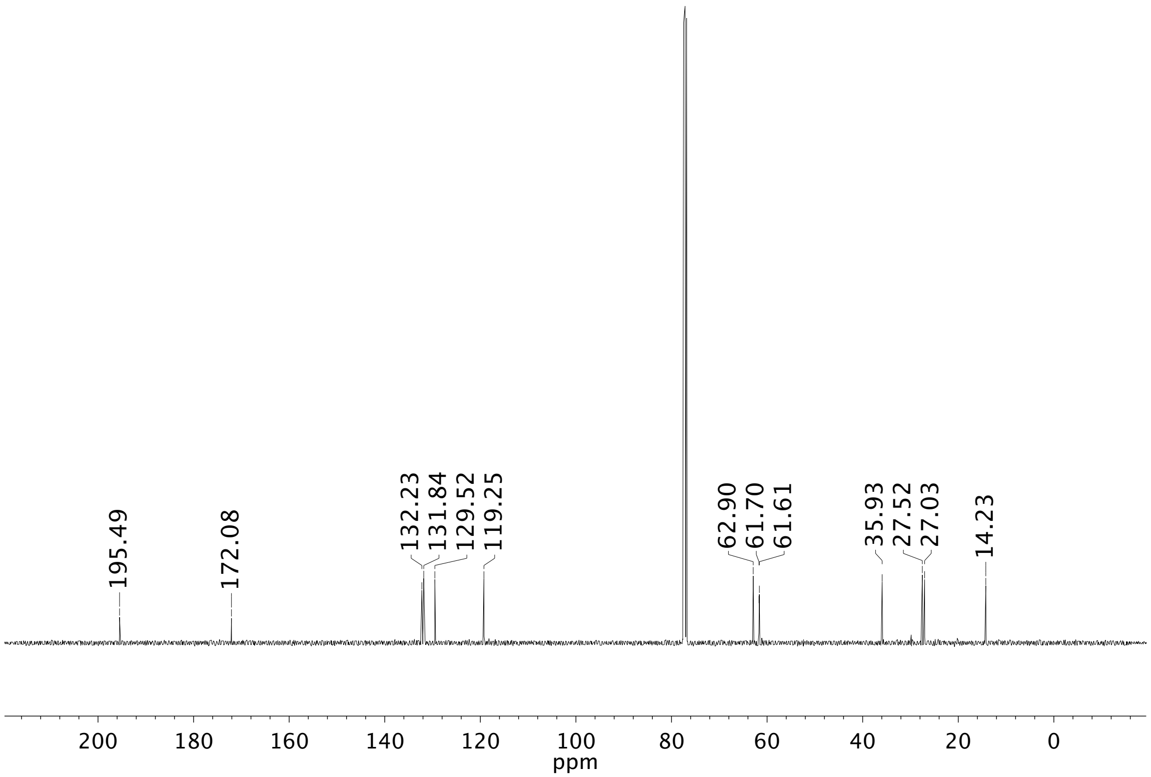

^{13}C NMR (101 MHz, $CDCl_3$) of compound **7**.

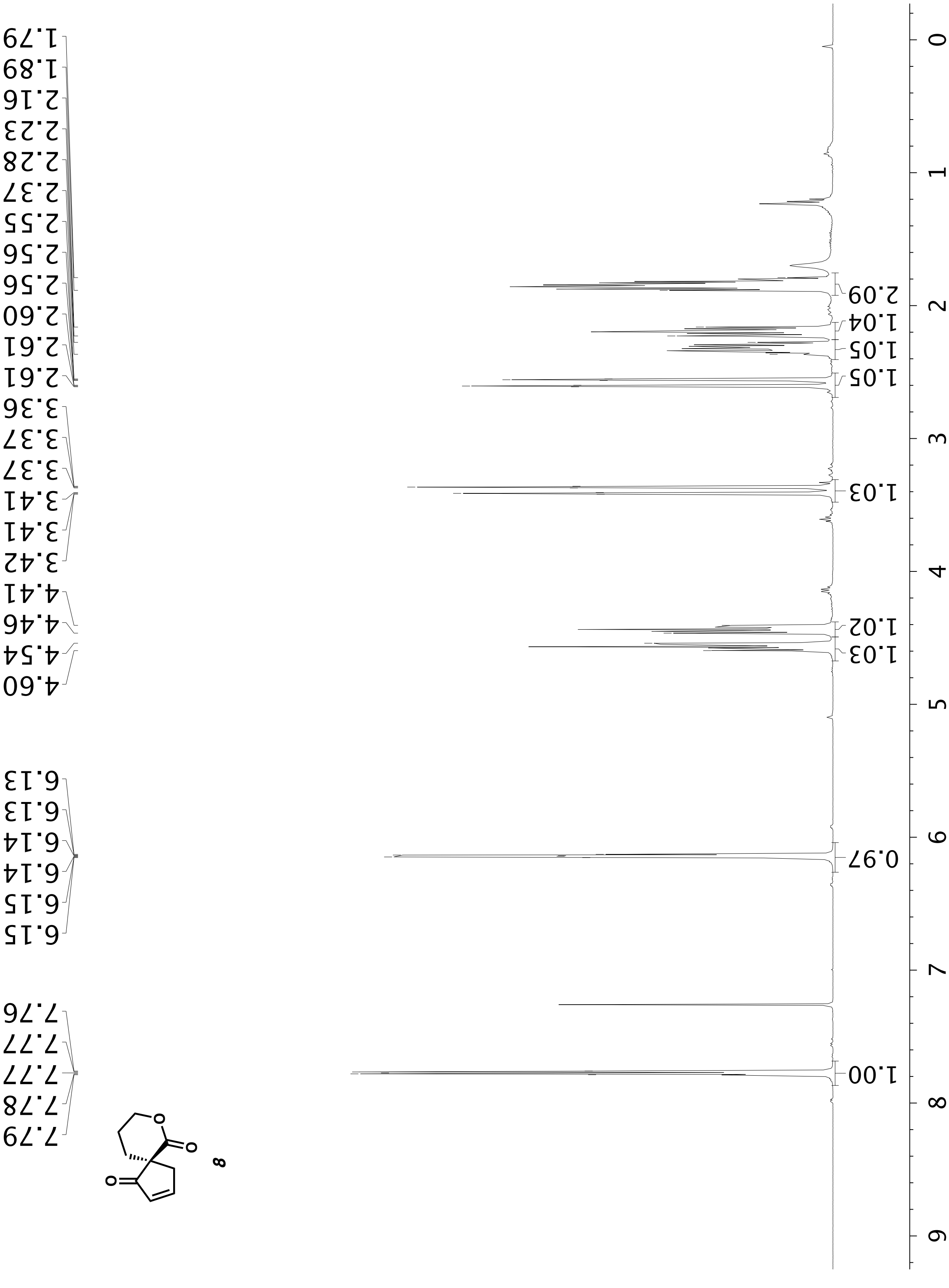

^{1}H NMR (400 MHz, $CDCl_3$) of compound **8**.

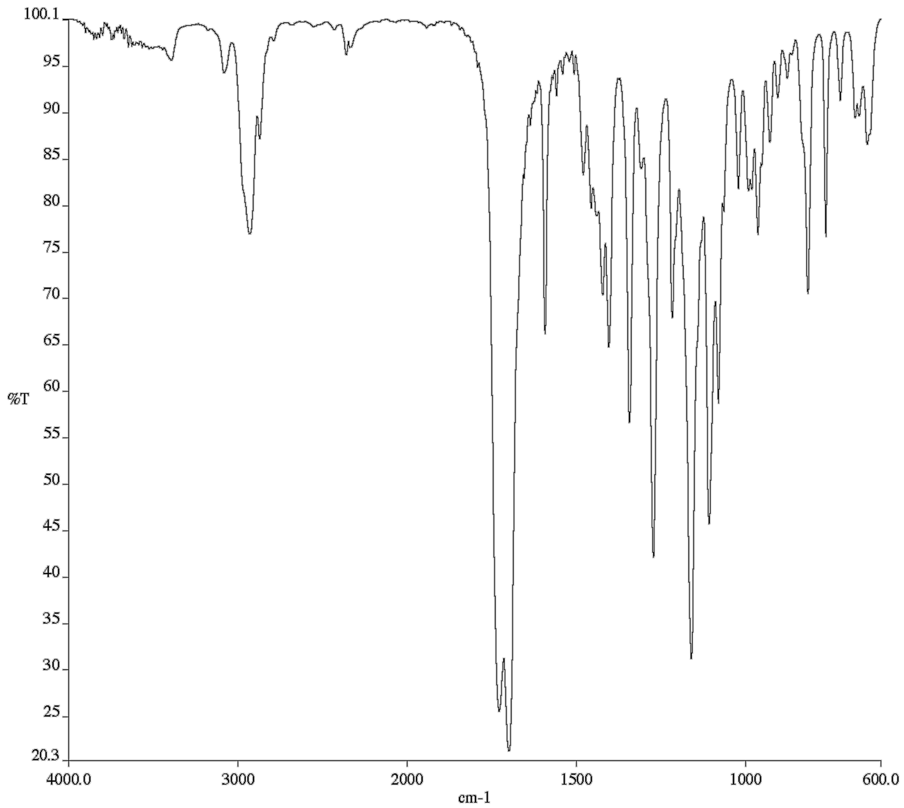

Infrared spectrum (Thin Film, NaCl) of compound **8**.

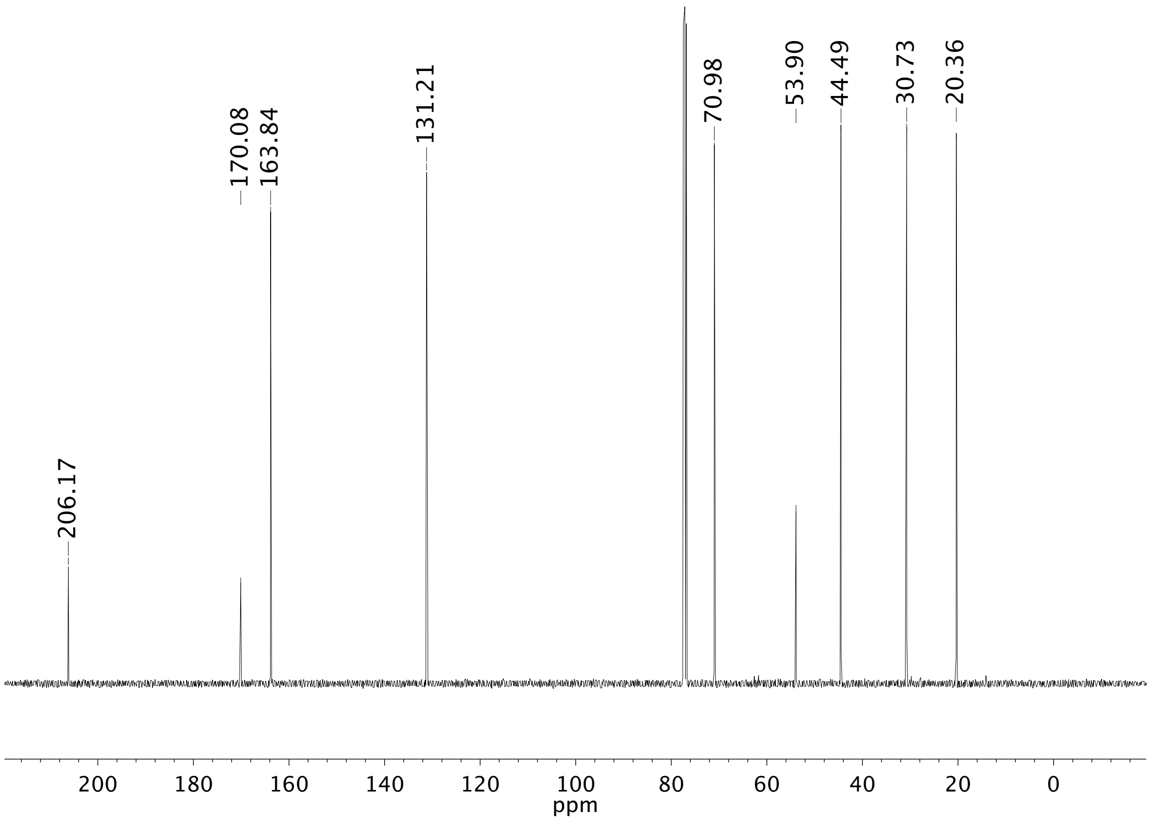

^{13}C NMR (101 MHz, $CDCl_3$) of compound **8**.

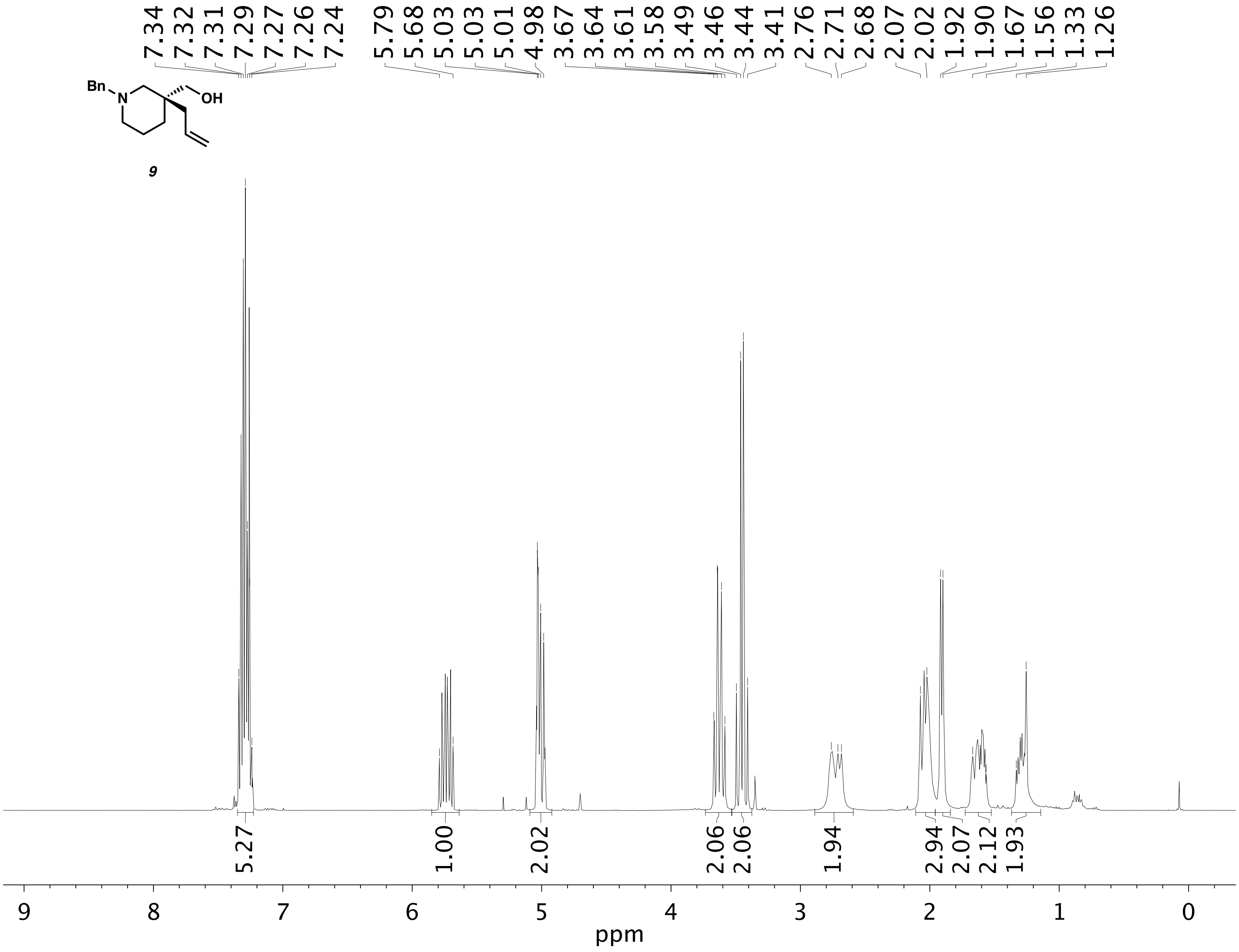

^{1}H NMR (400 MHz, $CDCl_3$) of compound **9**.

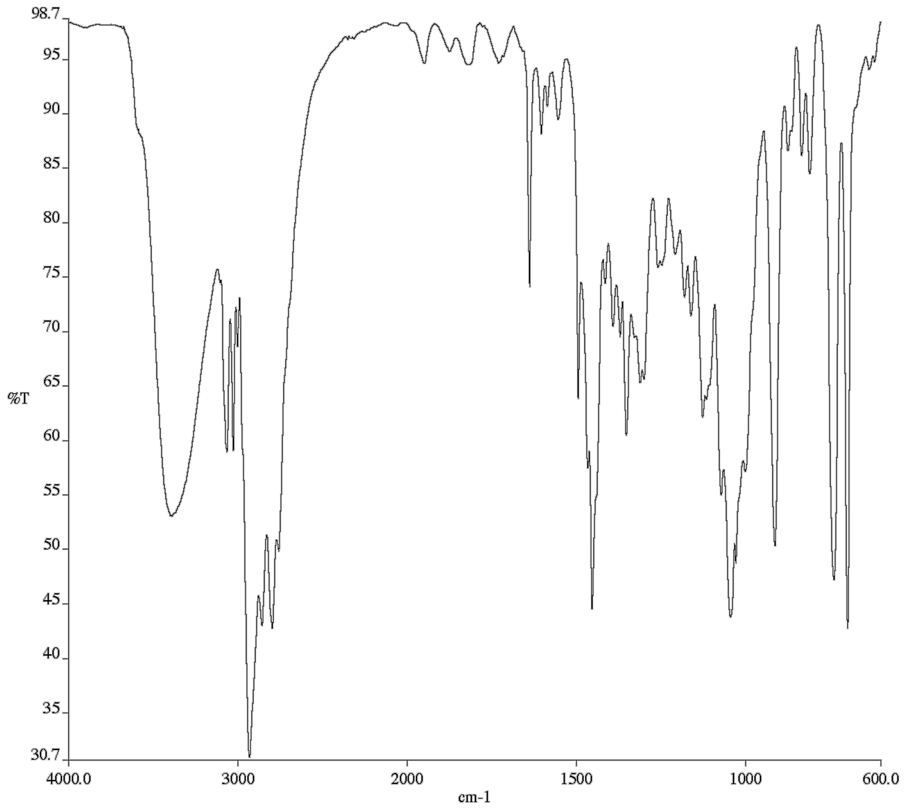

Infrared spectrum (Thin Film, NaCl) of compound **9**.

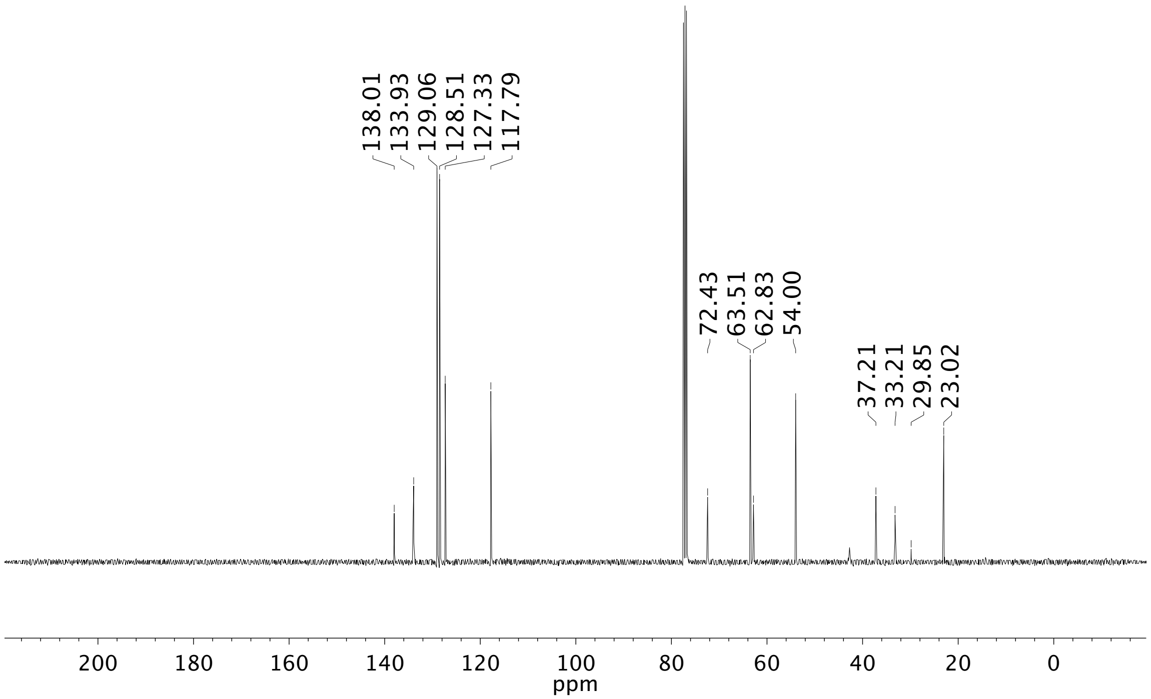

^{13}C NMR (101 MHz, $CDCl_3$) of compound **9**.

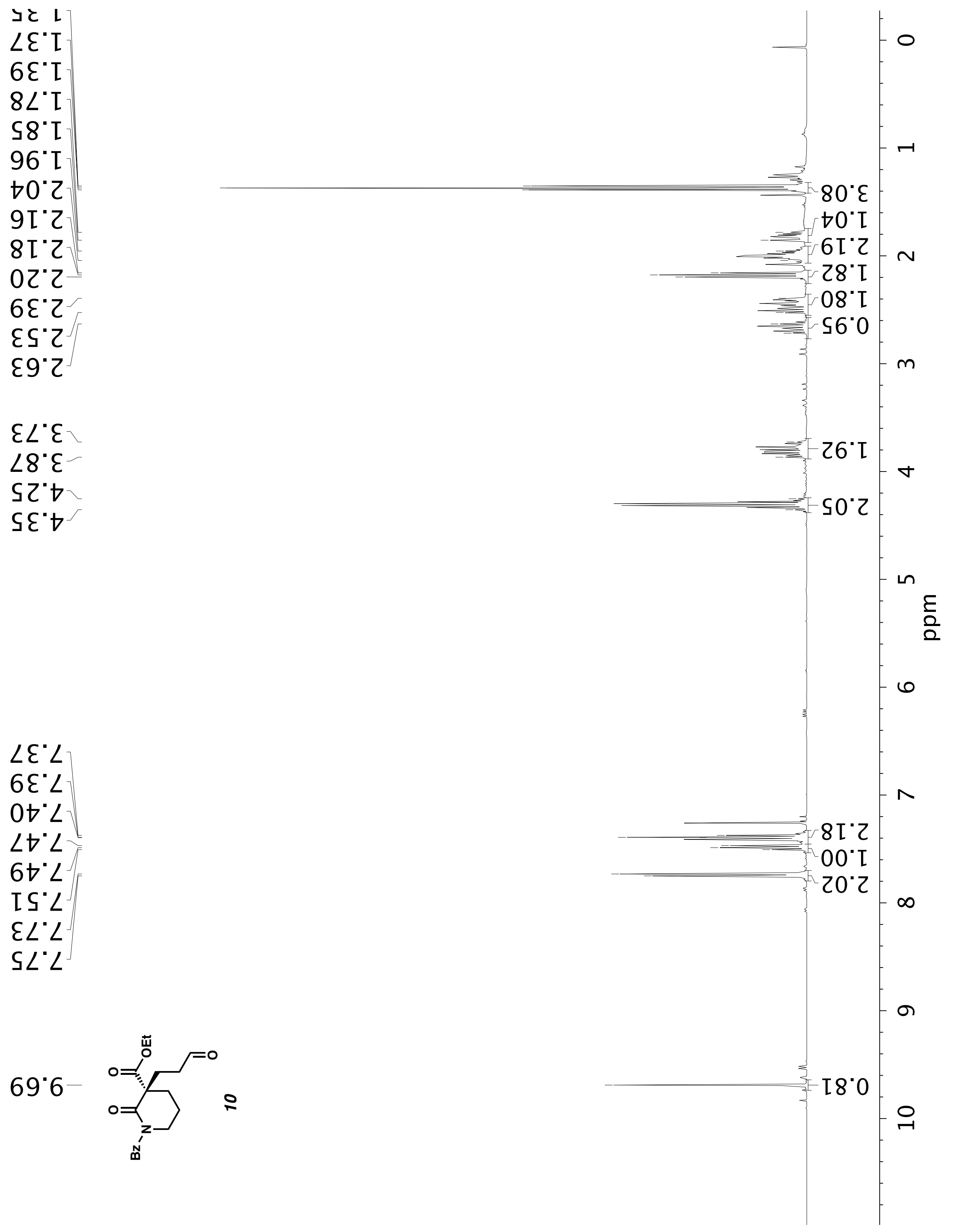

^1H NMR (400 MHz, $CDCl_3$) of compound **10**.

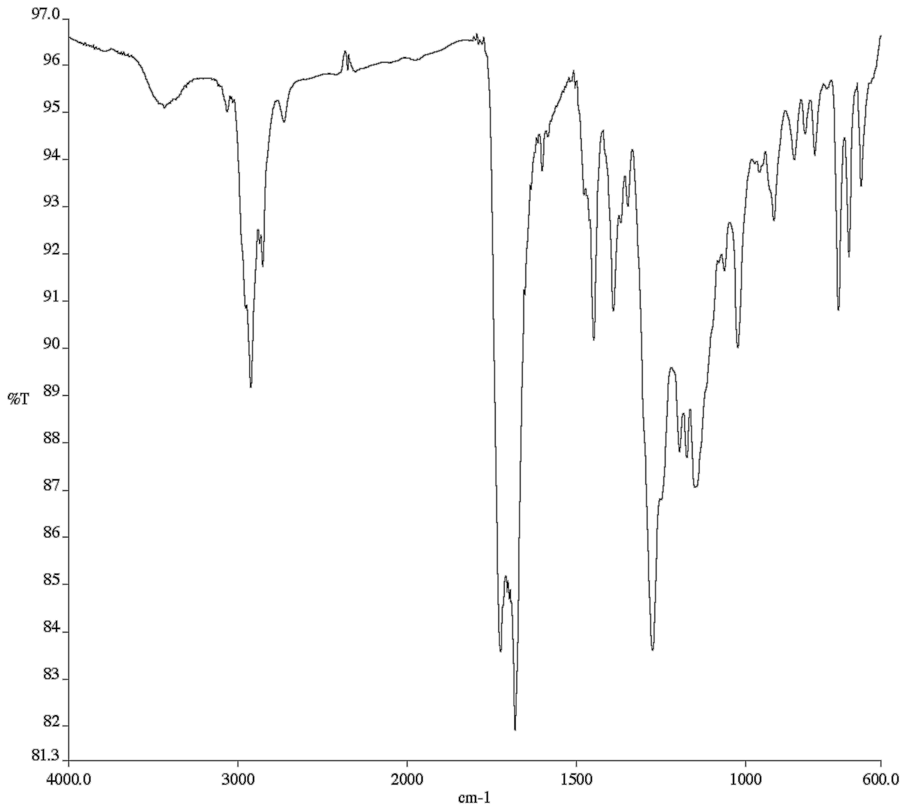

Infrared spectrum (Thin Film, NaCl) of compound **10**.

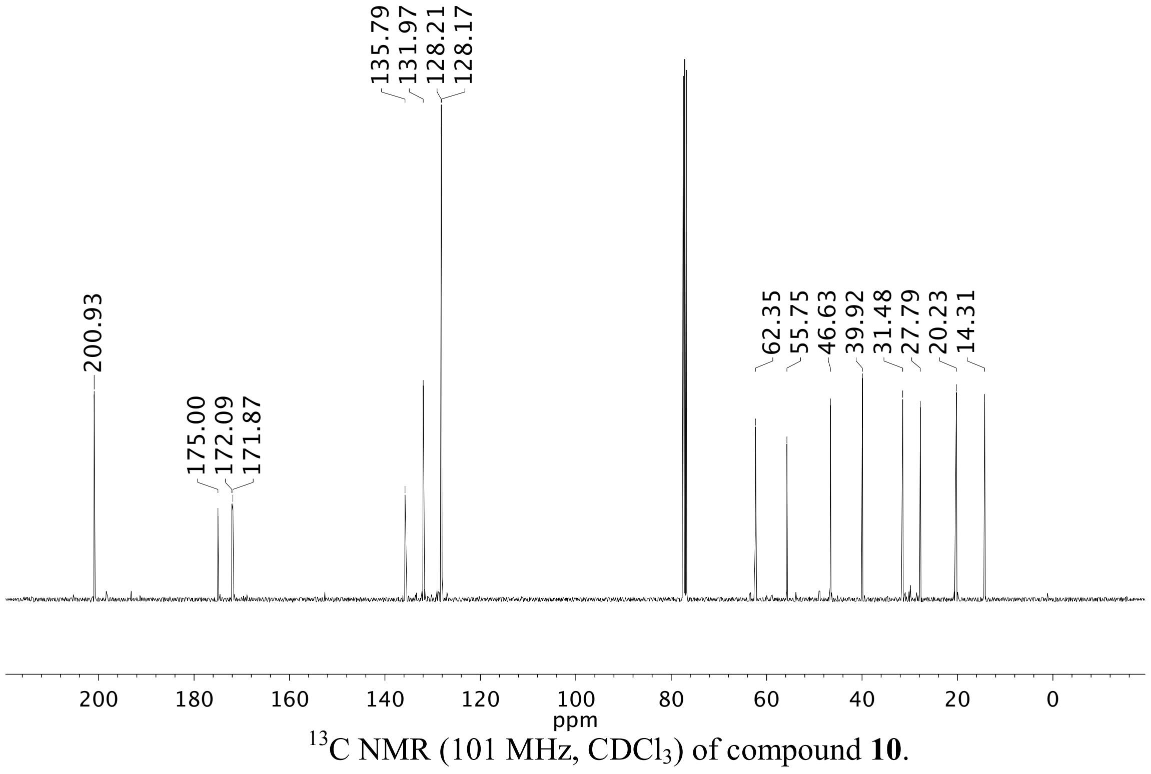

^{13}C NMR (101 MHz, $CDCl_3$) of compound **10**.

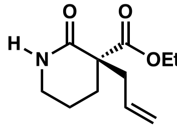

11

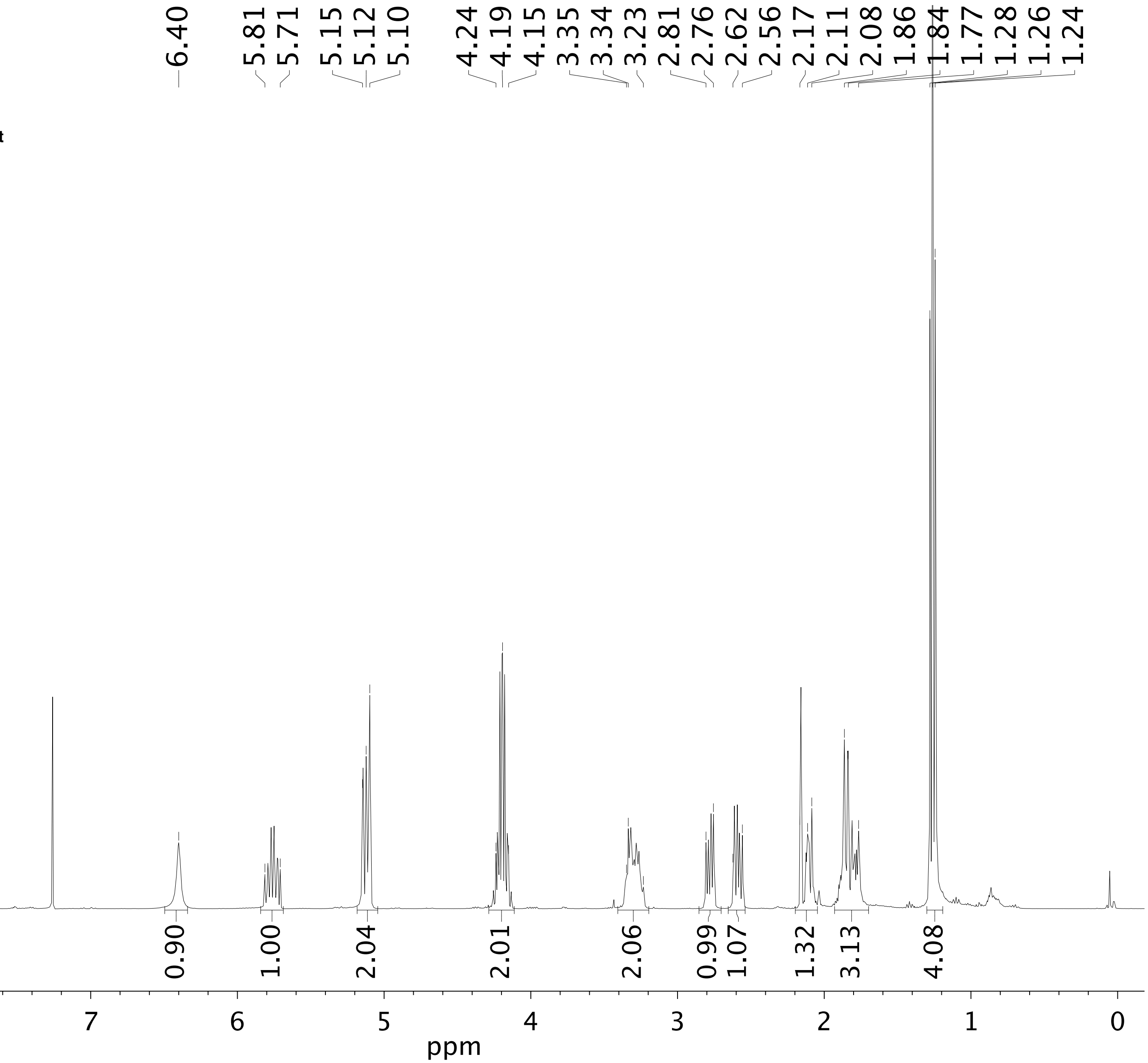

^{1}H NMR (400 MHz, $CDCl_3$) of compound **11**.

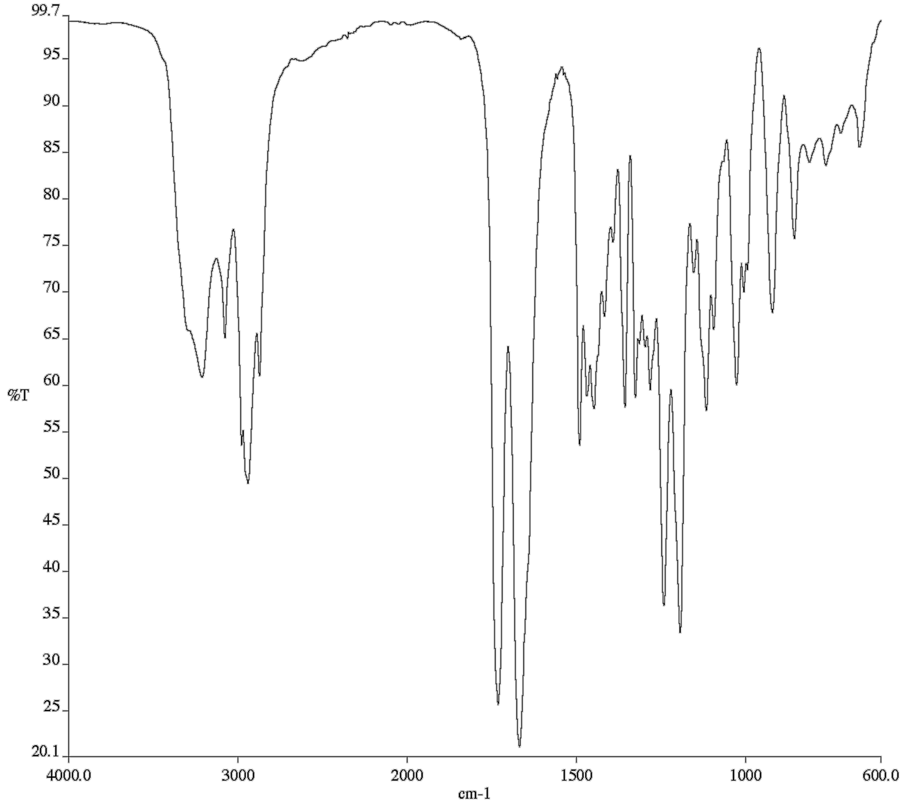

Infrared spectrum (Thin Film, NaCl) of compound **11**.

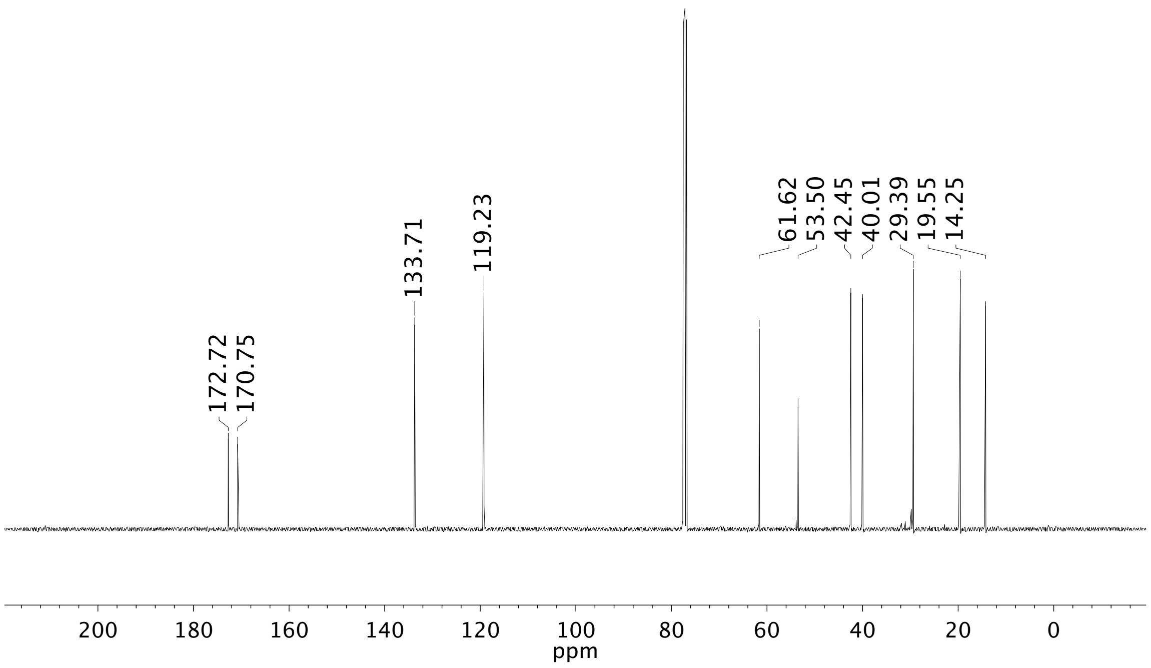

^{13}C NMR (101 MHz, $CDCl_3$) of compound **11**.

SFC Traces of Racemic and Enantioenriched Compounds

Racemic **3aa**

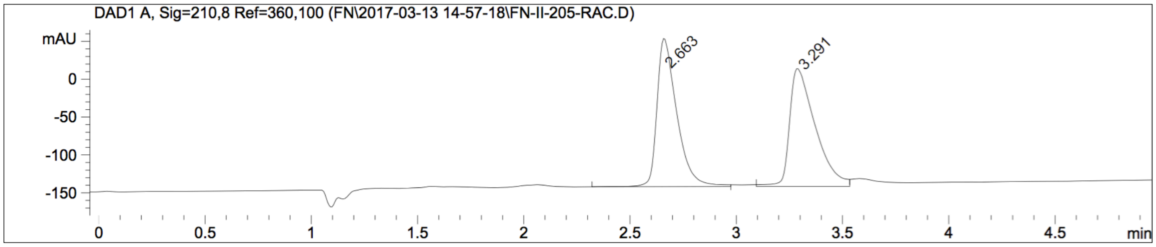

Signal 1: DAD1 A, Sig=210,8 Ref=360,100

Peak #	RetTime [min]	Type	Width [min]	Area [mAU*s]	Height [mAU]	Area %
1	2.663	BB	0.1010	1271.81873	192.82362	49.2206
2	3.291	BV	0.1273	1312.09692	154.23398	50.7794

Enantioenriched **3aa**

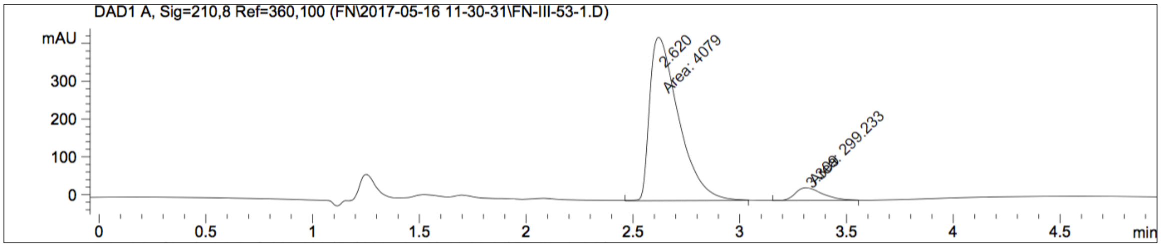

Signal 1: DAD1 A, Sig=210,8 Ref=360,100

Peak #	RetTime [min]	Type	Width [min]	Area [mAU*s]	Height [mAU]	Area %
1	2.620	MM	0.1572	4079.00391	432.53638	93.1655
2	3.309	MM	0.1476	299.23267	33.77860	6.8345

Racemic **3ba**

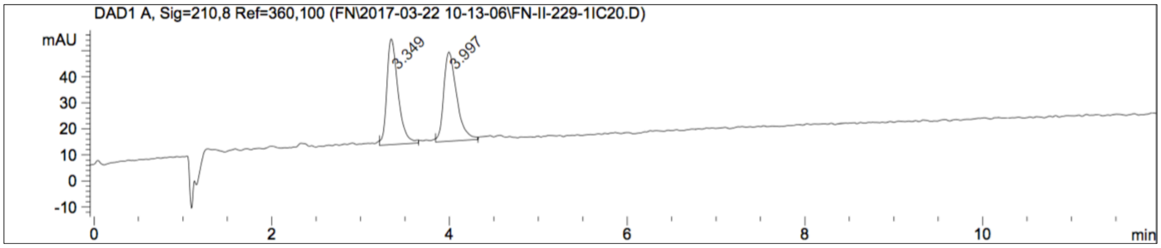

Signal 1: DAD1 A, Sig=210,8 Ref=360,100

Peak #	RetTime [min]	Type	Width [min]	Area [mAU*s]	Height [mAU]	Area %
1	3.349	BB	0.1347	356.33640	40.47424	50.6192
2	3.997	BB	0.1549	347.61929	34.09465	49.3808

Enantioenriched **3ba**

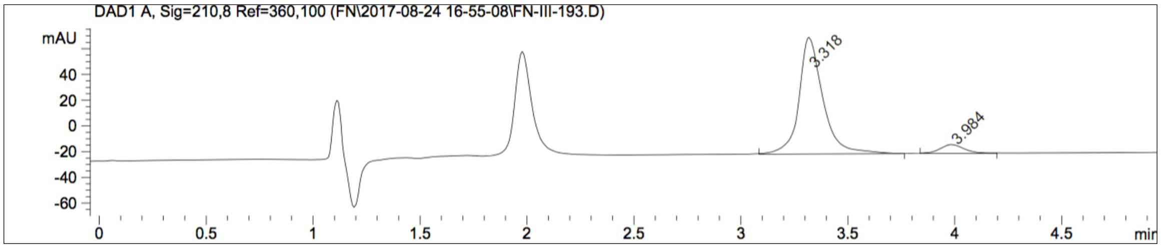

Signal 1: DAD1 A, Sig=210,8 Ref=360,100

Peak #	RetTime [min]	Type	Width [min]	Area [mAU*s]	Height [mAU]	Area %
1	3.318	BB	0.1152	709.51819	90.74887	92.9654
2	3.984	BB	0.1189	53.68814	6.88919	7.0346

Racemic **3ca**

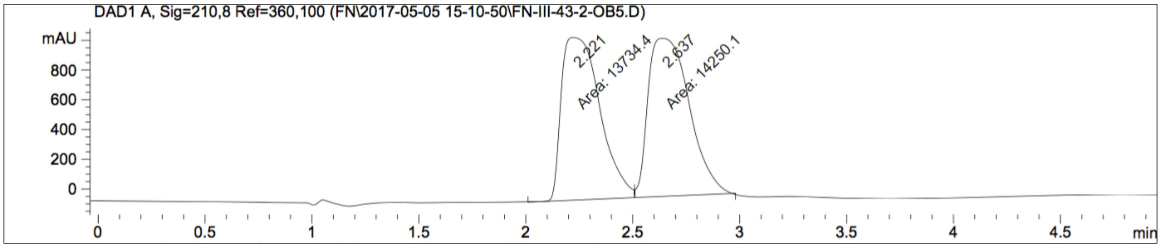

Signal 1: DAD1 A, Sig=210,8 Ref=360,100

Peak #	RetTime [min]	Type	Width [min]	Area [mAU*s]	Height [mAU]	Area %
1	2.221	MF	0.2089	1.37344e4	1096.02075	49.0786
2	2.637	FM	0.2228	1.42501e4	1066.19885	50.9214

Enantioenriched **3ca**

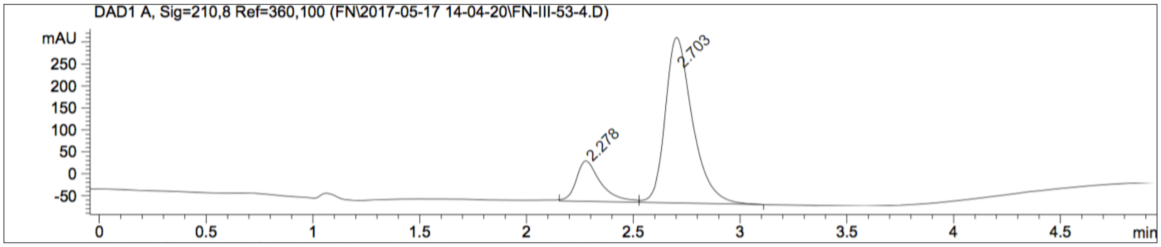

Signal 1: DAD1 A, Sig=210,8 Ref=360,100

Peak #	RetTime [min]	Type	Width [min]	Area [mAU*s]	Height [mAU]	Area %
1	2.278	BV	0.1192	737.10541	92.31647	17.9960
2	2.703	VB	0.1339	3358.83203	377.07315	82.0040

Racemic 3ab

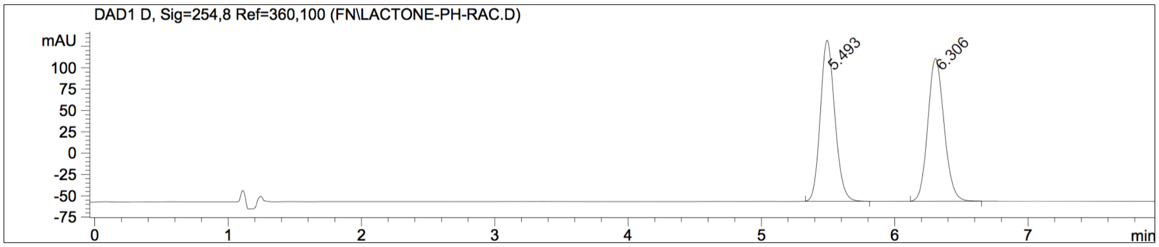

Signal 2: DAD1 D, Sig=254,8 Ref=360,100

Peak #	RetTime [min]	Type	Width [min]	Area [mAU*s]	Height [mAU]	Area %
1	5.493	BB	0.1167	1431.48157	188.18626	50.1938
2	6.306	BB	0.1311	1420.42896	167.16872	49.8062

Enantioenriched **3ab**

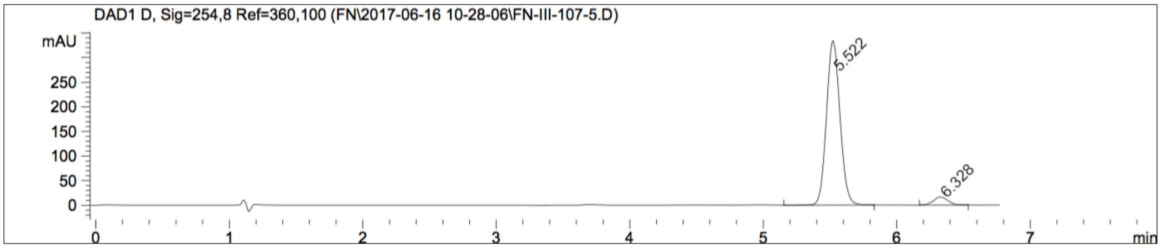

Signal 2: DAD1 D, Sig=254,8 Ref=360,100

Peak #	RetTime [min]	Type	Width [min]	Area [mAU*s]	Height [mAU]	Area %
1	5.522	VB	0.1101	2349.74902	333.80411	94.9723
2	6.328	BB	0.1187	124.39114	16.35471	5.0277

Racemic **3ac**

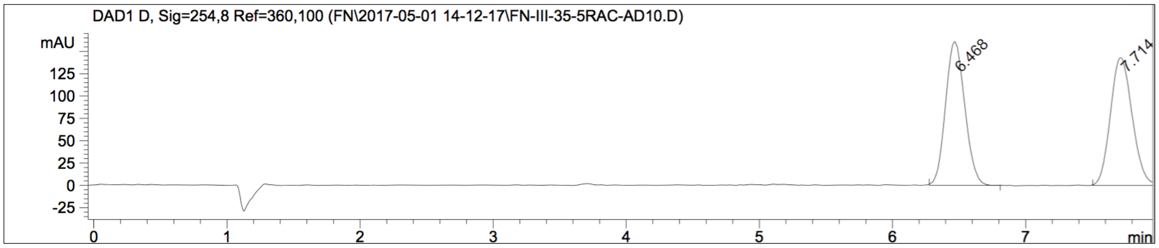

Signal 2: DAD1 D, Sig=254,8 Ref=360,100

Peak #	RetTime [min]	Type	Width [min]	Area [mAU*s]	Height [mAU]	Area %
1	6.468	BB	0.1577	1620.27124	160.62090	49.9937
2	7.714	BBA	0.1760	1620.67798	143.22626	50.0063

Enantioenriched **3ac**

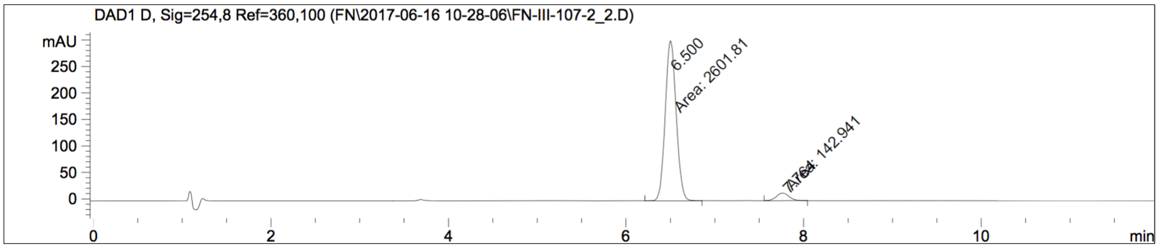

Signal 2: DAD1 D, Sig=254,8 Ref=360,100

Peak #	RetTime [min]	Type	Width [min]	Area [mAU*s]	Height [mAU]	Area %
1	6.500	MM	0.1435	2601.80762	302.23407	94.7922
2	7.764	MM	0.1656	142.94135	14.38287	5.2078

Racemic **3ad**

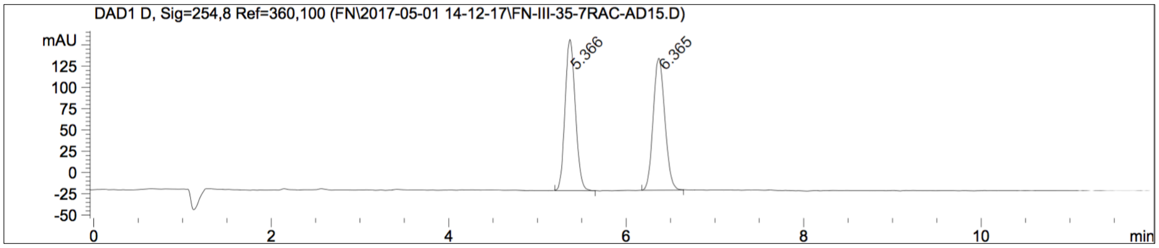

Signal 2: DAD1 D, Sig=254,8 Ref=360,100

Peak #	RetTime [min]	Type	Width [min]	Area [mAU*s]	Height [mAU]	Area %
1	5.366	BB	0.1257	1427.41321	177.71332	50.2194
2	6.365	BB	0.1424	1414.94116	155.19765	49.7806

Enantioenriched **3ad**

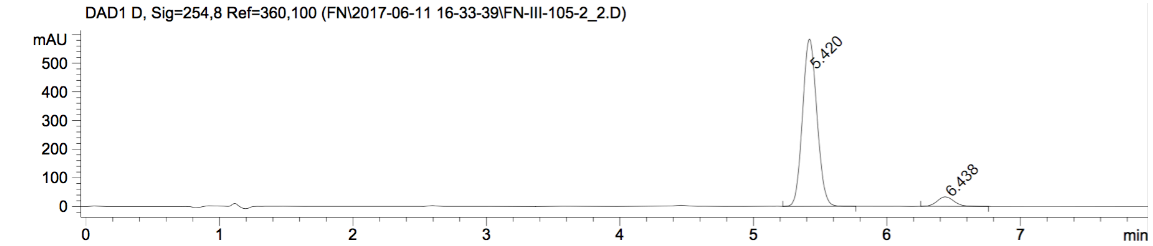

Signal 2: DAD1 D, Sig=254,8 Ref=360,100

Peak #	RetTime [min]	Type	Width [min]	Area [mAU*s]	Height [mAU]	Area %
1	5.420	BB	0.1175	4384.74365	584.36005	93.7721
2	6.438	BB	0.1358	291.21390	33.37838	6.2279

Racemic **3ae**

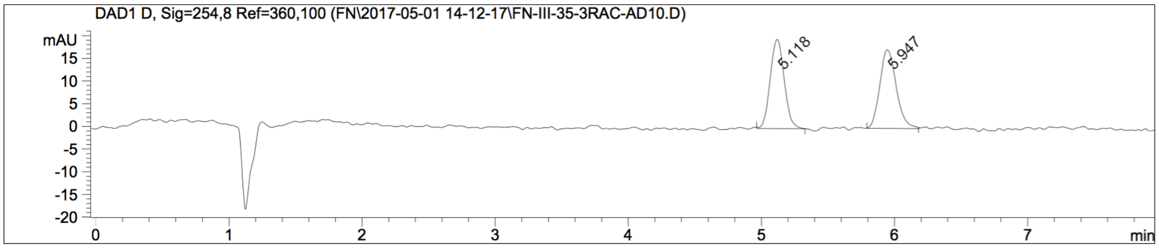

Signal 2: DAD1 D, Sig=254,8 Ref=360,100

Peak #	RetTime [min]	Type	Width [min]	Area [mAU*s]	Height [mAU]	Area %
1	5.118	BB	0.1154	144.14236	19.68591	49.1936
2	5.947	BB	0.1322	148.86833	17.34062	50.8064

Enantioenriched **3ae**

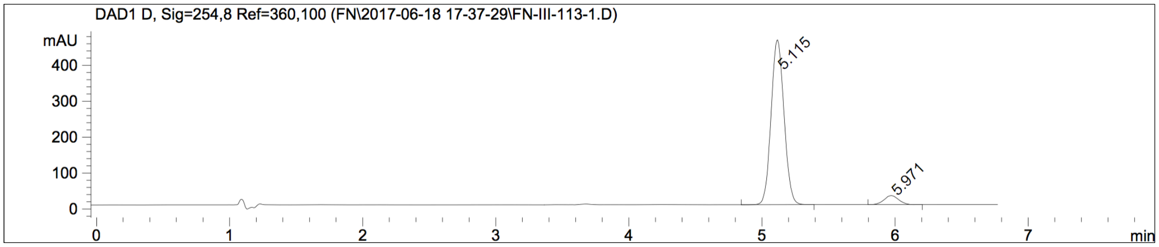

Signal 2: DAD1 D, Sig=254,8 Ref=360,100

Peak #	RetTime [min]	Type	Width [min]	Area [mAU*s]	Height [mAU]	Area %
1	5.115	BB	0.1047	3099.44531	459.32068	94.1400
2	5.971	BB	0.1189	192.93187	25.31065	5.8600

Racemic **3af**

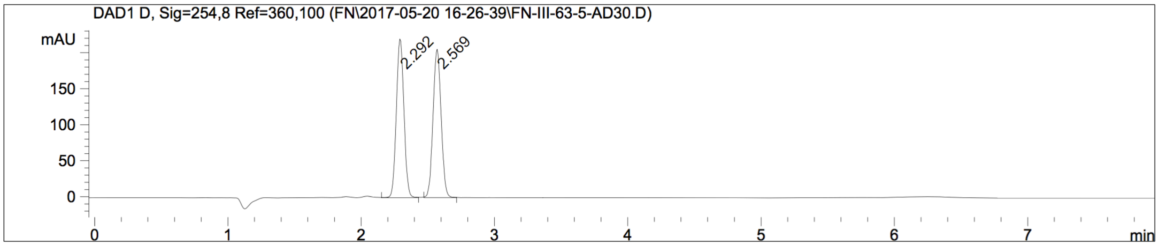

Signal 2: DAD1 D, Sig=254,8 Ref=360,100

Peak #	RetTime [min]	Type	Width [min]	Area [mAU*s]	Height [mAU]	Area %
1	2.292	VB	0.0621	871.48029	220.66896	50.0852
2	2.569	BB	0.0672	868.51556	205.93077	49.9148

Enantioenriched **3af**

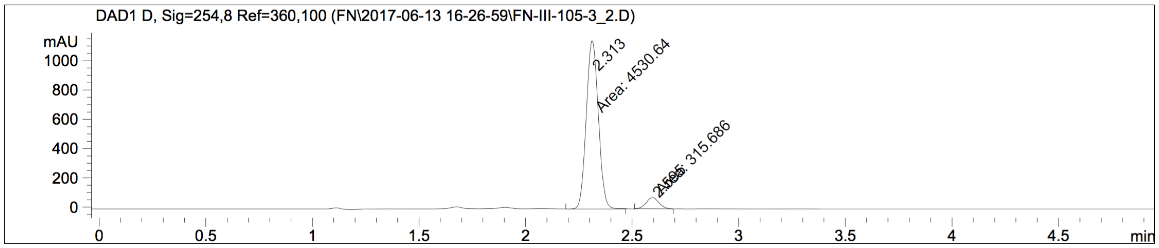

Signal 2: DAD1 D, Sig=254,8 Ref=360,100

Peak #	RetTime [min]	Type	Width [min]	Area [mAU*s]	Height [mAU]	Area %
1	2.313	MM	0.0652	4530.63818	1157.30896	93.4861
2	2.595	MM	0.0684	315.68573	76.92510	6.5139

Racemic **3ag**

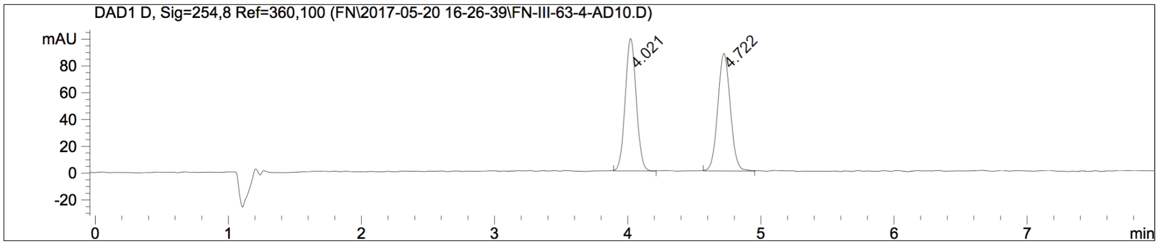

Signal 2: DAD1 D, Sig=254,8 Ref=360,100

Peak #	RetTime [min]	Type	Width [min]	Area [mAU*s]	Height [mAU]	Area %
1	4.021	BB	0.0891	569.60089	98.99100	49.7169
2	4.722	BB	0.1025	576.08771	87.84515	50.2831

Enantioenriched **3ag**

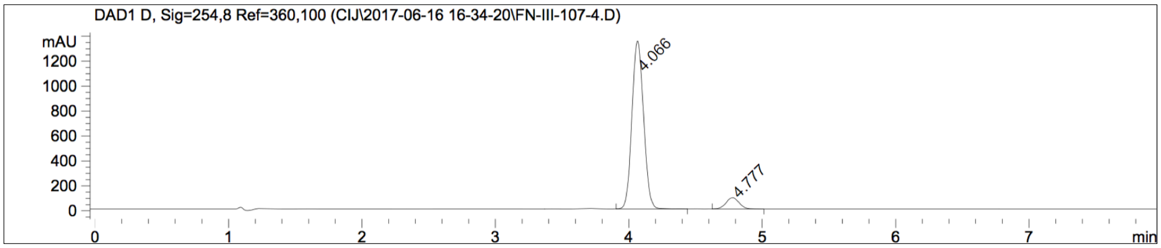

Signal 2: DAD1 D, Sig=254,8 Ref=360,100

Peak #	RetTime [min]	Type	Width [min]	Area [mAU*s]	Height [mAU]	Area %
1	4.066	BB	0.0988	8166.38477	1346.01123	92.9903
2	4.777	BB	0.1070	615.58563	90.86684	7.0097

Racemic **3ah**

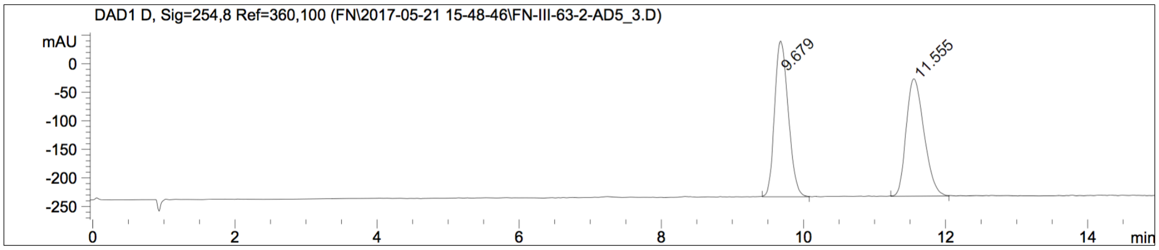

Signal 2: DAD1 D, Sig=254,8 Ref=360,100

Peak #	RetTime [min]	Type	Width [min]	Area [mAU*s]	Height [mAU]	Area %
1	9.679	BB	0.2081	3600.74146	272.66794	50.1264
2	11.555	BB	0.2713	3582.58228	205.54137	49.8736

Enantioenriched **3ah**

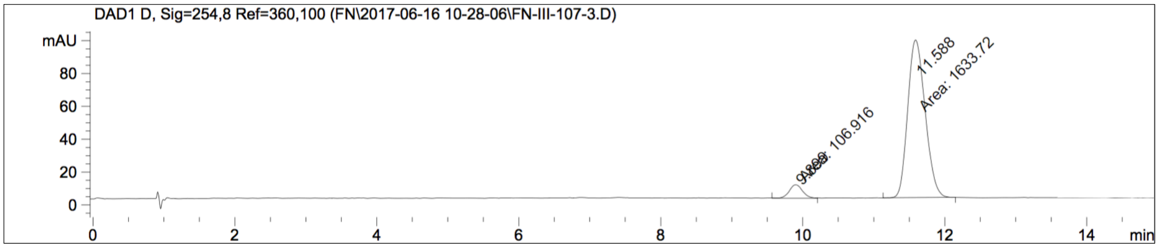

Signal 2: DAD1 D, Sig=254,8 Ref=360,100

Peak #	RetTime [min]	Type	Width [min]	Area [mAU*s]	Height [mAU]	Area %
1	9.899	MM	0.2158	106.91570	8.25906	6.1423
2	11.588	MM	0.2838	1633.72107	95.94908	93.8577

Racemic **3ai**

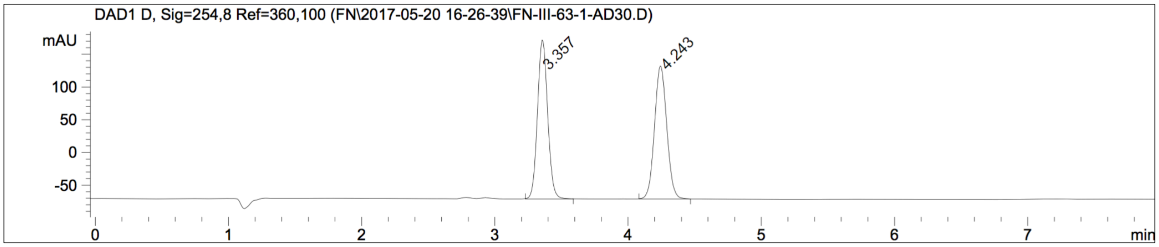

Signal 2: DAD1 D, Sig=254,8 Ref=360,100

Peak #	RetTime [min]	Type	Width [min]	Area [mAU*s]	Height [mAU]	Area %
1	3.357	BB	0.0821	1292.65442	242.93283	49.9831
2	4.243	BB	0.0982	1293.52759	203.45856	50.0169

Enantioenriched **3ai**

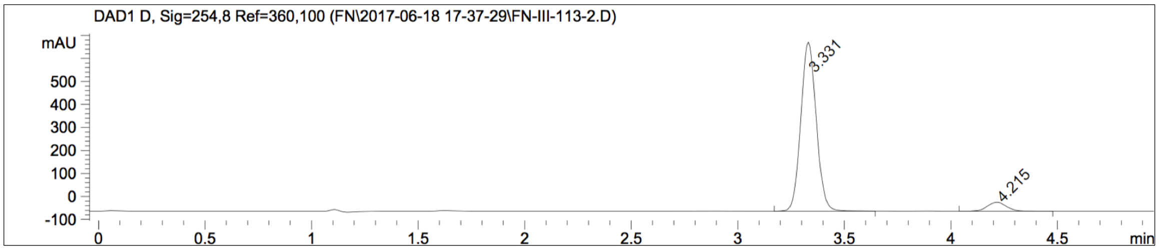

Signal 2: DAD1 D, Sig=254,8 Ref=360,100

Peak #	RetTime [min]	Type	Width [min]	Area [mAU*s]	Height [mAU]	Area %
1	3.331	BB	0.0769	3705.67261	734.24329	93.9213
2	4.215	VB	0.0957	239.83379	39.01456	6.0787

Racemic **3aj**

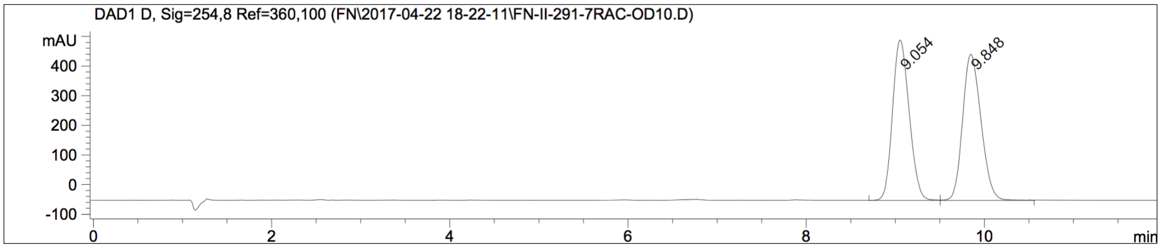

Signal 2: DAD1 D, Sig=254,8 Ref=360,100

Peak #	RetTime [min]	Type	Width [min]	Area [mAU*s]	Height [mAU]	Area %
1	9.054	VV	0.2039	7046.79688	541.37317	49.8581
2	9.848	VV	0.2277	7086.89697	493.04605	50.1419

Enantioenriched **3aj**

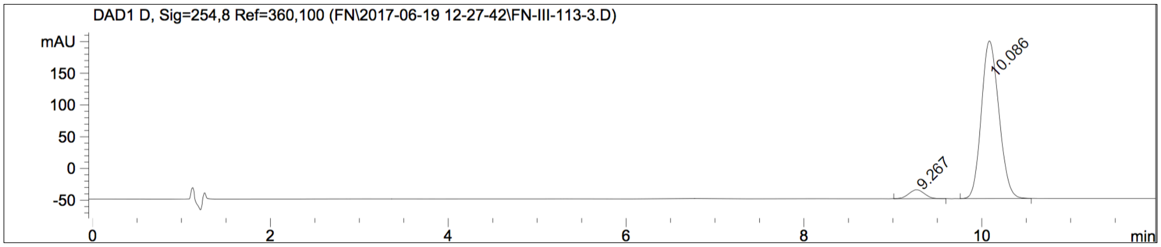

Signal 2: DAD1 D, Sig=254,8 Ref=360,100

Peak #	RetTime [min]	Type	Width [min]	Area [mAU*s]	Height [mAU]	Area %
1	9.267	BB	0.1900	173.35948	14.24575	4.8279
2	10.086	BB	0.2145	3417.41455	248.43091	95.1721

Racemic **3ak**

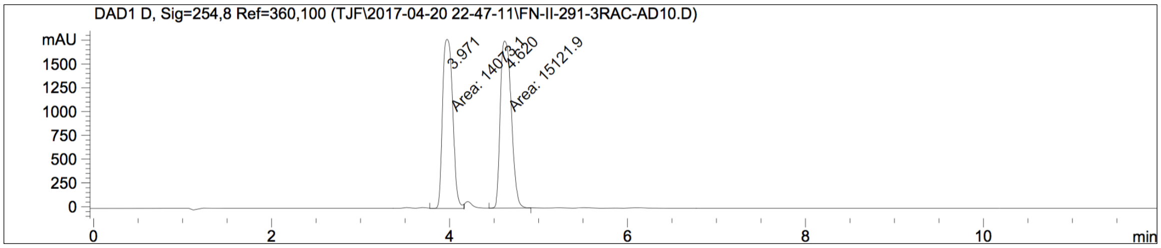

Signal 2: DAD1 D, Sig=254,8 Ref=360,100

Peak #	RetTime [min]	Type	Width [min]	Area [mAU*s]	Height [mAU]	Area %
1	3.971	MM	0.1320	1.40731e4	1777.38208	48.2039
2	4.620	MM	0.1436	1.51219e4	1755.34912	51.7961

Enantioenriched **3ak**

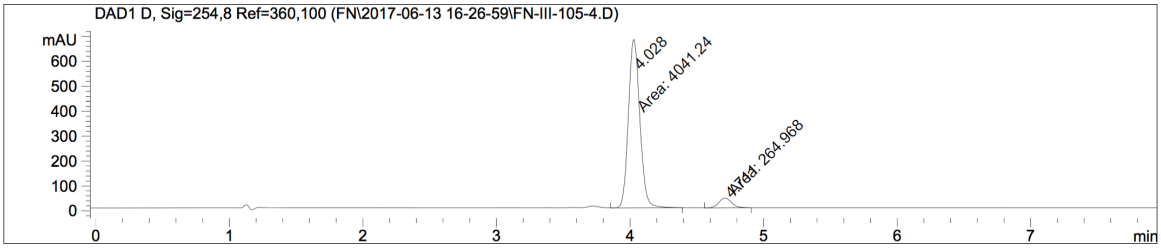

Signal 2: DAD1 D, Sig=254,8 Ref=360,100

Peak #	RetTime [min]	Type	Width [min]	Area [mAU*s]	Height [mAU]	Area %
1	4.028	MM	0.0993	4041.23779	678.18256	93.8468
2	4.711	MM	0.1113	264.96799	39.69333	6.1532

Racemic **3al**

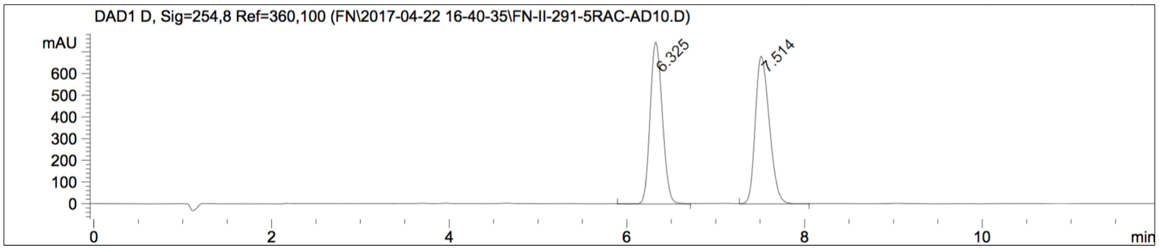

Signal 2: DAD1 D, Sig=254,8 Ref=360,100

Peak #	RetTime [min]	Type	Width [min]	Area [mAU*s]	Height [mAU]	Area %
1	6.325	VV	0.1518	7137.40479	745.04968	48.7423
2	7.514	VV	0.1727	7505.74561	680.44904	51.2577

Enantioenriched **3al**

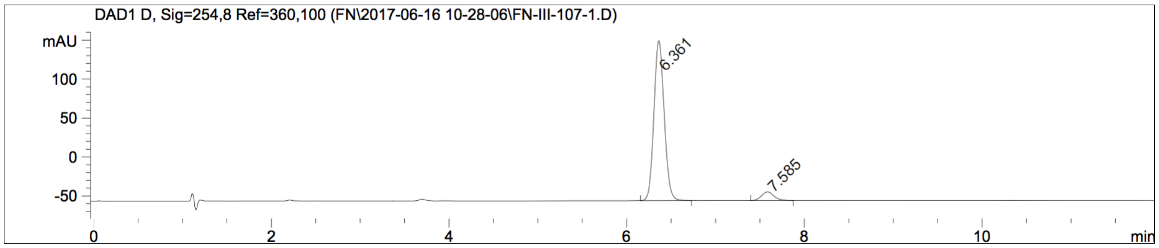

Signal 2: DAD1 D, Sig=254,8 Ref=360,100

Peak #	RetTime [min]	Type	Width [min]	Area [mAU*s]	Height [mAU]	Area %
1	6.361	BB	0.1236	1648.06433	205.36577	93.8136
2	7.585	BB	0.1500	108.67855	11.12690	6.1864

Racemic **3am**

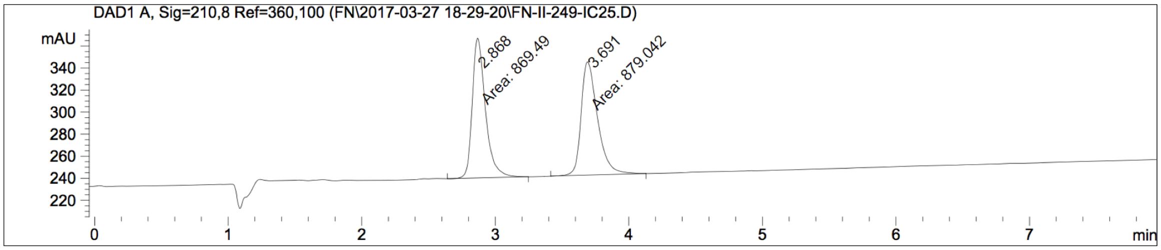

Signal 1: DAD1 A, Sig=210,8 Ref=360,100

Peak #	RetTime [min]	Type	Width [min]	Area [mAU*s]	Height [mAU]	Area %
1	2.868	MM	0.1139	869.48956	127.17487	49.7268
2	3.691	MM	0.1420	879.04242	103.21030	50.2732

Enantioenriched **3am**

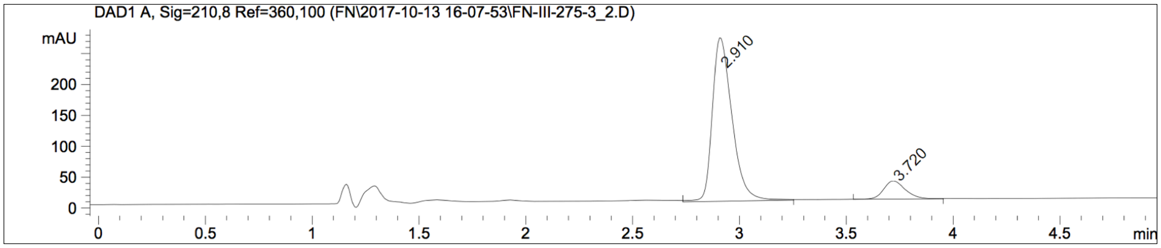

Signal 1: DAD1 A, Sig=210,8 Ref=360,100

Peak #	RetTime [min]	Type	Width [min]	Area [mAU*s]	Height [mAU]	Area %
1	2.910	VB	0.0964	1693.62781	265.69073	88.9254
2	3.720	BB	0.1088	210.92073	29.70895	11.0746

Racemic **3an**

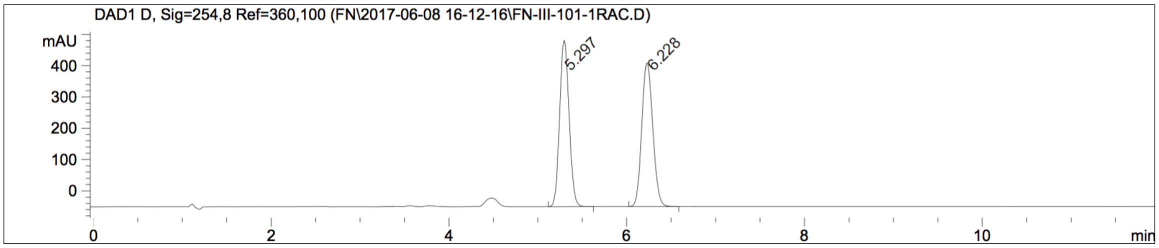

Signal 2: DAD1 D, Sig=254,8 Ref=360,100

Peak #	RetTime [min]	Type	Width [min]	Area [mAU*s]	Height [mAU]	Area %
1	5.297	BB	0.1182	3917.92212	530.28558	49.9952
2	6.228	BB	0.1357	3918.68042	458.49170	50.0048

Enantioenriched **3an**

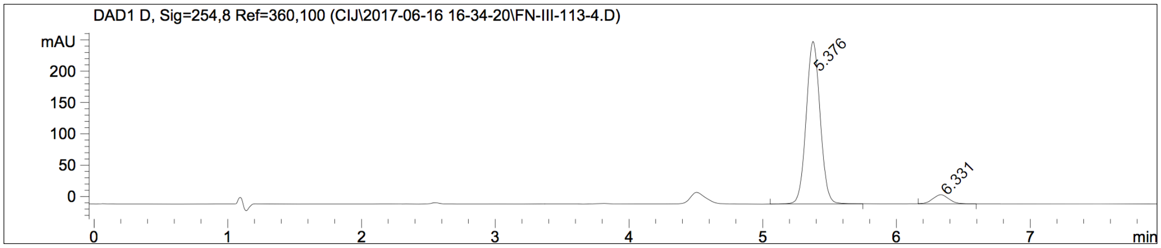

Signal 2: DAD1 D, Sig=254,8 Ref=360,100

Peak #	RetTime [min]	Type	Width [min]	Area [mAU*s]	Height [mAU]	Area %
1	5.376	BB	0.1128	1884.45520	259.40671	94.0390
2	6.331	BB	0.1286	119.45214	14.42992	5.9610

Racemic **5aa**

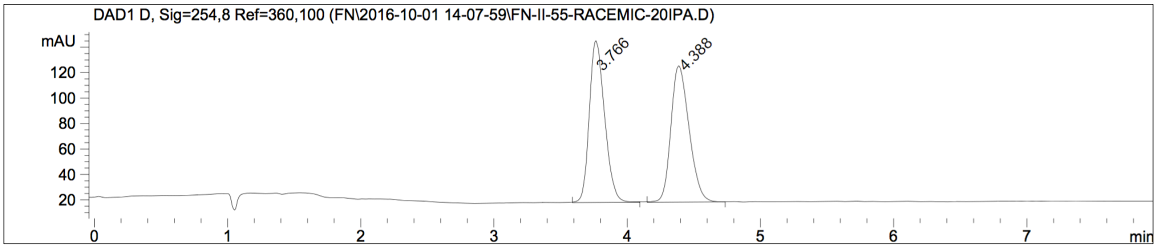

Signal 2: DAD1 D, Sig=254,8 Ref=360,100

Peak #	RetTime [min]	Type	Width [min]	Area [mAU*s]	Height [mAU]	Area %
1	3.766	BB	0.1253	1016.56195	127.06444	50.0580
2	4.388	BB	0.1466	1014.20660	107.02486	49.9420

Enantioenriched **5aa**

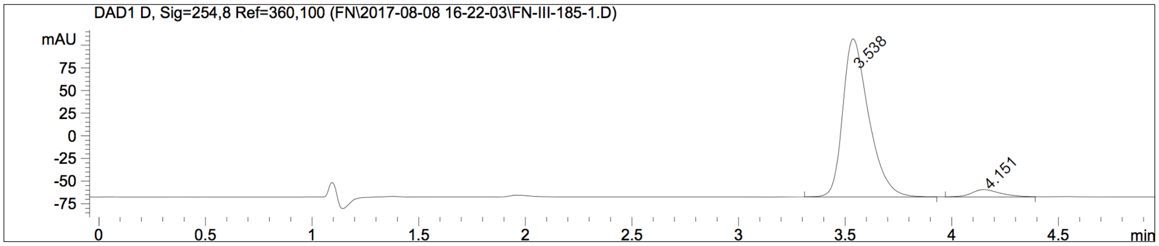

Signal 2: DAD1 D, Sig=254,8 Ref=360,100

Peak #	RetTime [min]	Type	Width [min]	Area [mAU*s]	Height [mAU]	Area %
1	3.538	BB	0.1271	1485.68469	174.90120	95.0771
2	4.151	BV	0.1400	76.92561	8.16185	4.9229

Racemic **5ba**

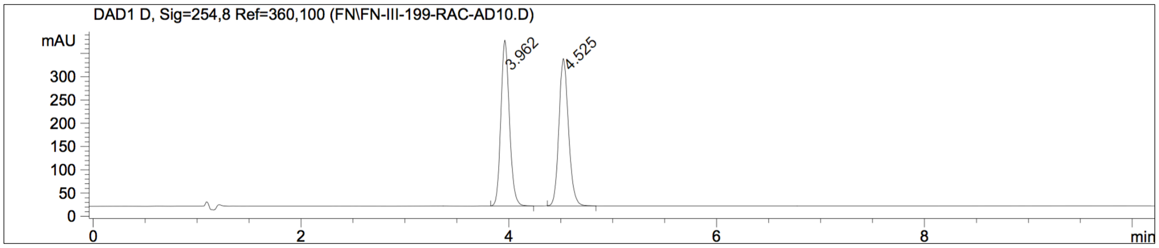

Signal 2: DAD1 D, Sig=254,8 Ref=360,100

Peak #	RetTime [min]	Type	Width [min]	Area [mAU*s]	Height [mAU]	Area %
1	3.962	BB	0.0911	2040.92883	354.94333	49.9794
2	4.525	BB	0.0996	2042.61084	315.37271	50.0206

Enantioenriched **5ba**

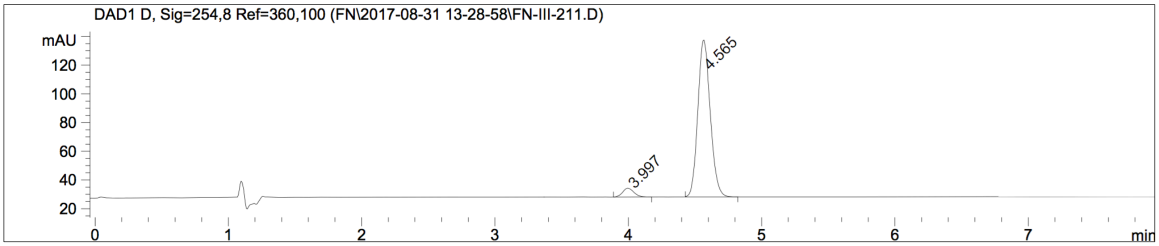

Signal 2: DAD1 D, Sig=254,8 Ref=360,100

Peak #	RetTime [min]	Type	Width [min]	Area [mAU*s]	Height [mAU]	Area %
1	3.997	BB	0.0906	36.39927	6.19094	4.8796
2	4.565	BB	0.0996	709.55328	109.53050	95.1204

Racemic **5ab**

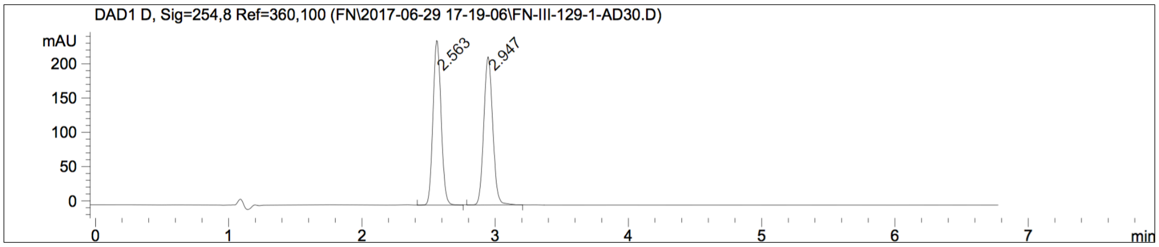

Signal 2: DAD1 D, Sig=254,8 Ref=360,100

Peak #	RetTime [min]	Type	Width [min]	Area [mAU*s]	Height [mAU]	Area %
1	2.563	VB	0.0650	1011.37903	241.02779	49.9815
2	2.947	BB	0.0726	1012.12671	216.18948	50.0185

Enantioenriched **5ab**

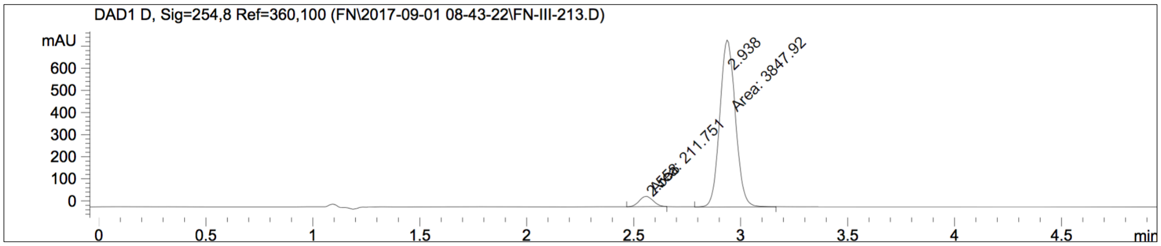

Signal 2: DAD1 D, Sig=254,8 Ref=360,100

Peak #	RetTime [min]	Type	Width [min]	Area [mAU*s]	Height [mAU]	Area %
1	2.558	MM	0.0750	211.75116	47.03239	5.2160
2	2.938	MM	0.0847	3847.92383	757.05585	94.7840

Racemic **7**

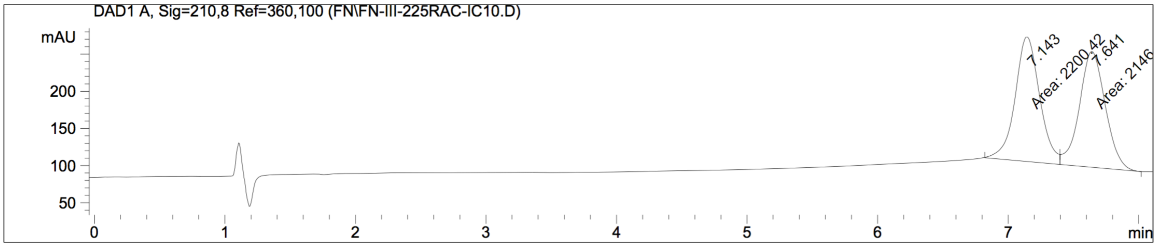

Signal 1: DAD1 A, Sig=210,8 Ref=360,100

Peak #	RetTime [min]	Type	Width [min]	Area [mAU*s]	Height [mAU]	Area %
1	7.143	MF	0.2183	2200.42358	168.01707	50.6247
2	7.641	FM	0.2295	2146.11572	155.82289	49.3753

Enantioenriched **7**

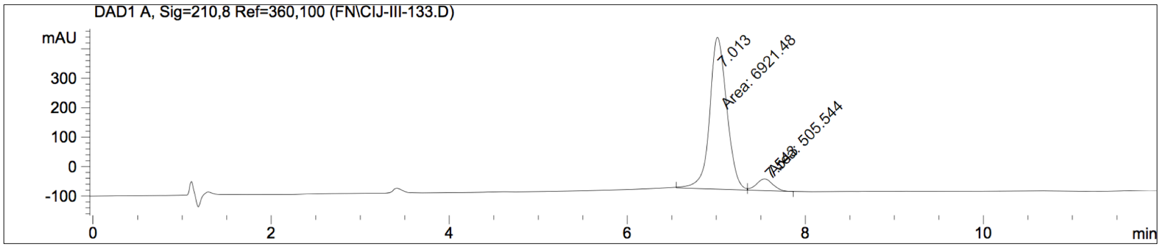

Signal 1: DAD1 A, Sig=210,8 Ref=360,100

Peak #	RetTime [min]	Type	Width [min]	Area [mAU*s]	Height [mAU]	Area %
1	7.013	MF	0.2234	6921.48047	516.47284	93.1932
2	7.543	FM	0.2113	505.54355	39.87366	6.8068
